# Conversion of waste poly(vinyl chloride) to branched polyethylene mediated by silylium ions†

**DOI:** 10.1039/d4sc00130c

**Published:** 2024-05-14

**Authors:** Zachary A. Wood, Eunice C. Castro, Angelyn N. Nguyen, Megan E. Fieser

**Affiliations:** a Department of Chemistry, University of Southern California Los Angeles CA 91706 USA; b Wrigley Institute for Environment and Sustainability University of Southern California Los Angeles CA 91706 USA fieser@usc.edu

## Abstract

Full dechlorination of poly(vinyl chloride) (PVC) in a controlled manner to yield useful polymeric and chlorinated products is of great interest for the processing of PVC waste. Forming polyethylene (PE) without corrosive by-products would allow for a pre-treatment of PE wastes that are often contaminated with PVC. Herein, full dechlorination of PVC has been achieved *via* generation of silylium ions *in situ*, to furnish PE products. Complete dechlorination of PVC can be achieved in 2 hours, yielding organic polymer that has similar spectroscopic and thermal signatures of branched PE, with no observable chlorine. The degree of branching can be tuned between 31 and 57 branches per 1000 carbons, with melting temperatures ranging from 51 to 93 °C. This method is applicable to not only pure PVC, but also commercial PVC products. Depending on if the PVC products are separated from plasticizers, different melting points of the resulting PE are observed. PVC dechlorination in the presence of PE waste is also shown. This is the first report of being able to cleanly convert PVC waste to PE in high yields and tune the thermal properties of the PE product, highlighting the remarkable control that silylium ion mediated transformations enables compared to past chemical methods.

## Abstract 2

Lewis acidic silylium ions are used to completely remove Cl from poly(vinyl chloride) (PVC) in clean and commercial products through hydrodechlorination. Reaction conditions were found to impact the branching in the resulting polyethylene (PE).

## Graphical abstract

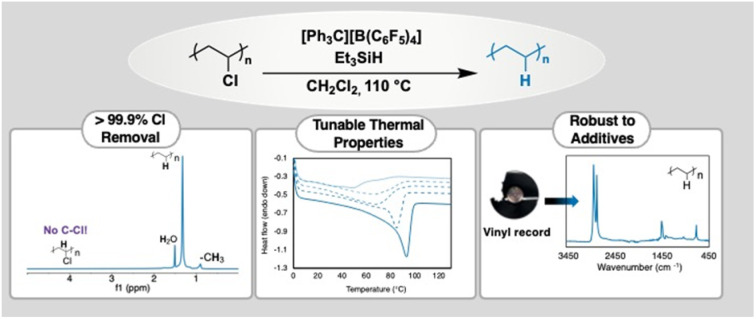

## Introduction

As the third-most produced plastic worldwide, the end-of-life of poly(vinyl chloride) (PVC) has garnered particular interest in addressing the plastic waste crisis due to its toxicity and the barriers it presents to recycling of mixed plastic streams.^1^ For example, mechanical recycling of PVC contaminated polyethylene (PE) waste results in release of HCl and Cl_2_, corroding recycling plants.^2^ Moreover, waxes and fuels from pyrolyzed polymers containing just 10 ppm Cl are unusable.^2^ Methods for recycling or upcycling PVC, as well as its compatibilization with other plastic waste recycling, are therefore vital.

Because polyolefin waste commonly has PVC (and other plastic) contamination, it is imperative to find multiple ways to process this plastic waste that can yield a variety of useful products. While processing of polyolefin waste to a distributions of small molecules and oligomers (*e.g.*, waxes and fuels) can add more value to contaminated waste,^3,4^ researching ways to convert such waste to practical materials that can be used for other applications would also be of importance. Converting PVC to PE, without the generation of corrosive byproducts, would be valuable as the product would promote more selective pyrolysis with longer-lived pyrolysis catalysts. An added benefit of producing PE compared to waxes and fuels is that the products can also be mechanically recycled.

**Fig. 1:**
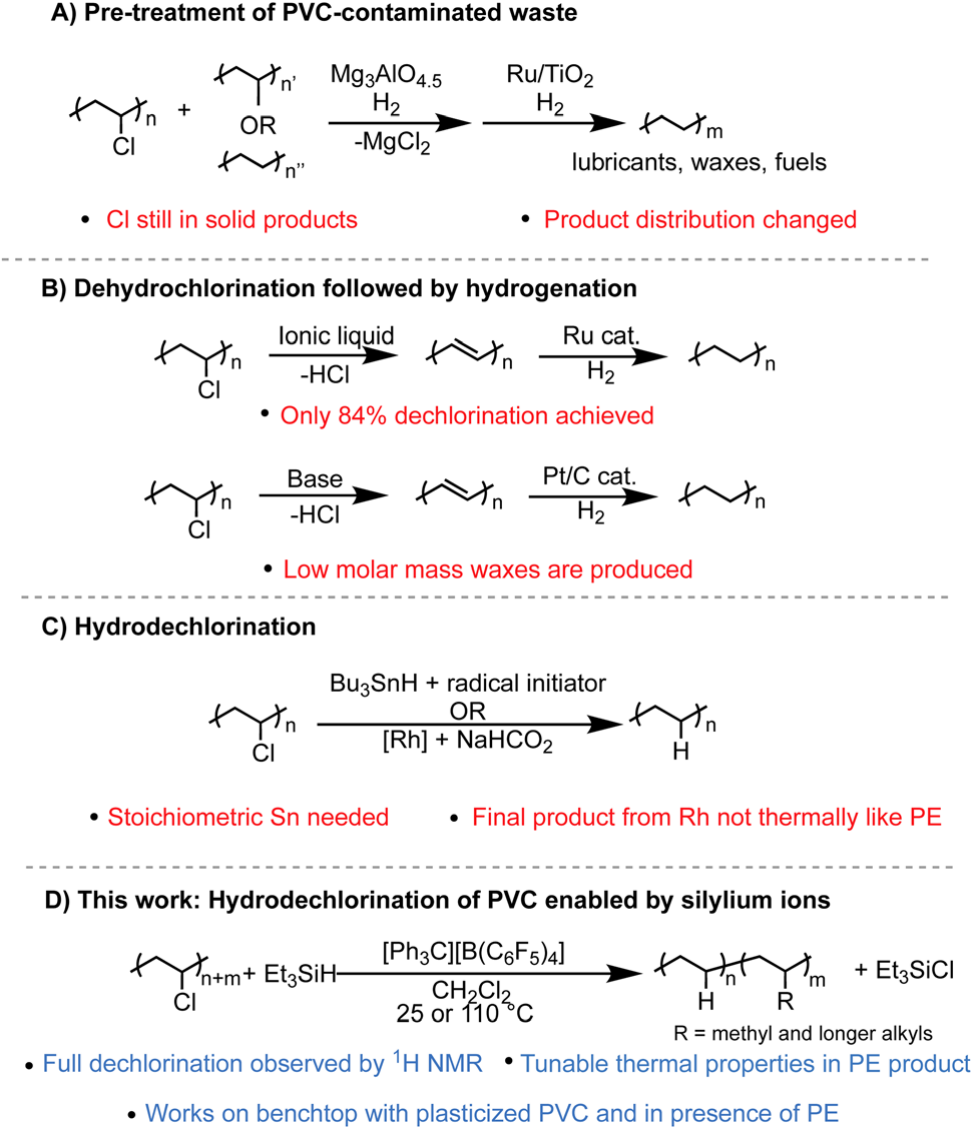
Summary of different methods (A–D) to dechlorinate PVC to PE-like materials.

There are many strategies that have been reported to dechlorinate PVC waste, yet two problems persist: full dechlorination is rarely achieved, and even if high dechlorination is achieved, the C–C backbone left behind is often not clean or selective, severely limiting its applications. A few emerging strategies include productive addition of the chlorine to other organic molecules,^5^ tandem dehydrochlorination^3,6^ and π-bond metathesis to achieve smaller molecules and oligomers,^7^ or tandem substitution of the Cl for H and arenes to form upcycled copolymers.^8^ While important in developing more ways to process PVC waste, these methods can't be used to convert PE/PVC waste into PE that can be mechanically recycled or pyrolyzed. Methods to process PVC contaminated PE waste can be separated into three categories. One method is the dechlorination of PVC-PE waste streams over solid material, such as Mg_3_AlO_4.5_, before pyrolysis is performed with a catalyst, often with Ru-based catalysts.^4,9–17^ However, in a recent example, the product selectivity of the Ru catalyst changes in the presence of chlorinated contamination.^4^ Additionally, the solid pyrolysis product still has Cl content (∼10 wt%) while the liquid fraction has <0.1 wt% (Fig. 1A). A second method is to perform sequential dehydrochlorination/hydrogenation to yield PE-like polymers. Only partial dechlorination (<90% dechlorination) is achieved in one case,^6^ and another case reports full dechlorination by Fourier-transform infrared spectroscopy (FT-IR) but product selectivity and yield are poor compared to other methods (Fig. 1B).^3^ The third method is to dechlorinate PVC selectively through reduction or hydrodechlorination to form PE-like materials using catalytic methods or stoichiometric reagents such as Bu_3_SnH.^18–20^ However, these reports either don't achieve full dechlorination, or if full dechlorination is achieved the polymer product does not have the necessary purity and thermal properties to enter the PE mechanical recycling stream (Fig. 1C).

We therefore sought to develop a system in which PVC can be dechlorinated fully to PE-like products, while also being purified of any reactants and matching the thermal properties of PE. Our recent report on using silylium ions generated *in situ* to dechlorinate PVC rapidly (5 minute reaction time) was a new strategy to convert PVC waste to polymers (in this case poly(ethylene-co-styrene) copolymers) with the potential for higher value applications.^8^ To form these copolymers, tandem hydrodechlorination/Friedel–Crafts reactivity is hypothesized. While full dechlorination is observed by ^1^H nuclear magnetic resonance spectroscopy (NMR), the resulting polymers cannot be recycled with PE due to the poly(styrene) (PS) moiety. Considering silylium ions were able to rapidly dechlorinate PVC in arene solvents, we questioned if the reaction conditions could be modified to fully dechlorinate PVC to PE as a proof-of-concept in mixed waste treatment before recycling. Not only would this furnish fully dechlorinated products, but Et_3_SiCl is also generated, which is much more benign than HCl and is a valuable chemical and a convenient starting point for many useful silicon containing compounds.^21^ However, the hydrodechlorination/Friedel–Crafts strategy was hypothesized to work so well due to the polymer products being soluble in the aromatic solvent, allowing for all the chlorine to be reached.^8^ Molecular analogues often use neat substrate, which would not be an option for PVC. With a very distinct solubility difference between PVC and PE, it was unclear if a method could be optimized to easily reach all chlorine in the polymer.

In regard to this goal, a recent report was published by Carta *et al.* that utilizes Lewis-acidic cations (Si^+^ and Zr^+^) to hydrodechlorination PVC up to 91%.^22^ By using a bromobenzene as the solvent, Friedel–Crafts reactivity is supressed and hydrodechlorination is the favored reaction. However, the polymer product is ill-defined, as many unwanted side reactions such as dehydrochlorination, chain scission, and cross linking are proposed to occur. Additionally, this report highlights how difficult it is to achieve >99% hydrodechlorination of PVC without the presence of deleterious side reactions.

Herein, we present the formation of branched PE products generated from the complete or near complete dechlorination of PVC in as little as two hours and as mild as room temperature (Fig. 1D). Adjusting reaction conditions was found to alter the production of PE with tunable degrees of branching, and therefore thermal properties, suggesting this process could work in the presence of different PE waste streams. Branched PE polymers have been of great interest, especially for use as thermoplastic elastomers, and are traditionally synthesized through metal catalyzed polymerization of ethylene and/or other olefins. This work represents a new pathway to achieve branched PE products from waste PVC material. Additionally, this method with silylium ions is remarkably robust to common plasticizers such as phthalate esters. Finally, the dechlorination of PVC in the presence of other plastics is also shown as a proof-of-concept of repurposing of PVC-contaminated waste streams.

## Results and discussion

**Scheme 1:**
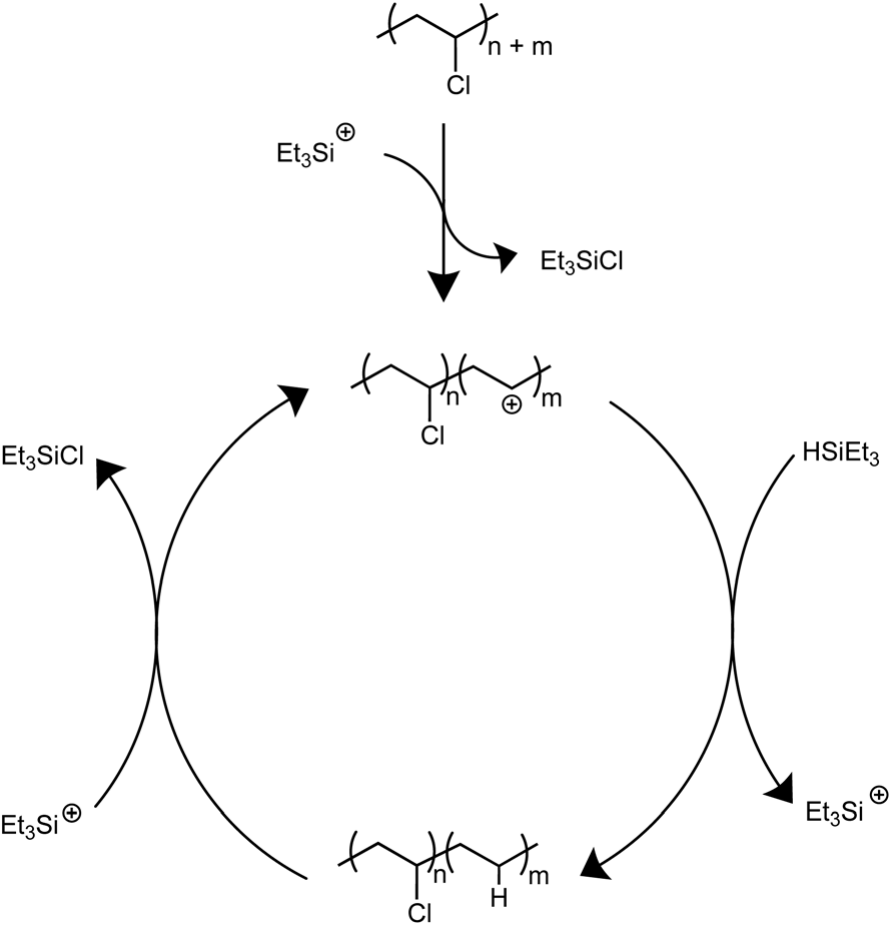
Proposed mechanism for dechlorination of PVC mediated by silylium ions.

Solvents were first screened that would enable hydrodechlorination without the presence of Friedel–Crafts reactivity (Scheme 1). Initial experiments were performed with 0.5 mol% of an initiator ([Ph_3_C][B(C_6_F_5_)_4_]) and 1.1 equivalence of Et_3_SiH at 110 °C for 18 h. Tetrahydrofuran (THF), dimethylacetamide (DMA), and cyclopentyl methyl ether (CpOMe) were all chosen first due to precedence for their ability to solubilize PVC. THF did not show any conversion of PVC to PE, which is unsurprising due to [Ph_3_C][B(C_6_F_5_)_4_] being able to ring-open polymerize THF.^23^ DMA and CpOMe also did not show any dechlorination, which is hypothesized to be due to the heteroatoms present forming strong interactions with silylium ions, rendering the catalyst in an unreactive dormant state.^24,25^ Attempts in non-polar, weakly coordinating solvents, such as hexanes and methylcyclohexane, showed little-to-no conversion observed by FT-IR spectroscopy even after 18 h at 110 °C. This can likely be due to the observation that no PVC dissolved in the solvent during the reaction time.

Dechlorination in the presence of dichloromethane (CH_2_Cl_2_),^26^ at 110 °C, showed >99% dechlorination after 18 h (by ^1^H NMR spectroscopy), see ESI† for details.^27–33^Scheme 1 represents the proposed mechanism for the hydrodechlorination of PVC mediated by silylium ions, which is based on the work of Ozerov and coworkers for the dechlorination of small molecule substrates.^34^ Gratifyingly, this reaction was performed on the benchtop with no drying needed of the PVC, Et_3_SiH, or CH_2_Cl_2_ used for the reaction. The [Ph_3_C][B(C_6_F_5_)_4_] can initiate the reaction even when stored in a desiccator on the benchtop for days, and the Et_3_SiH is still an active hydride source even when stored exposed to normal atmosphere. This is surprising given the highly Lewis-acidic nature of the presumed silylium ions being generated, and past researchers noting the necessity to rigorously dry all solvents and reagents in order to achieve high degrees of dechlorination.^24,25,34^ Even more surprising is the stability of the reaction in CH_2_Cl_2_, as past methods have mentioned activation of the C–Cl bond when generating silylium ions. CH_2_Cl_2_ activation in prior literature occurs with more activated (*e.g.*, more Lewis acidic) silylium ions that are sterically hindered, while our system likely never forms large concentrations of bare silylium ions due to excess Si–H present that forms [Si–H–Si]^+^ dimers, or silylium ions coordinated to CH_2_Cl_2_, both of which are less Lewis acidic. To test if the CH_2_Cl_2_ is dechlorinated to form CH_3_Cl or CH_4_, control reactions were performed in a Parr reactor, in which pressure build-up could be monitored. These reactions confirmed no evidence for CH_2_Cl_2_ activation (see ESI for more details, Table S7†).

**Fig. 2:**
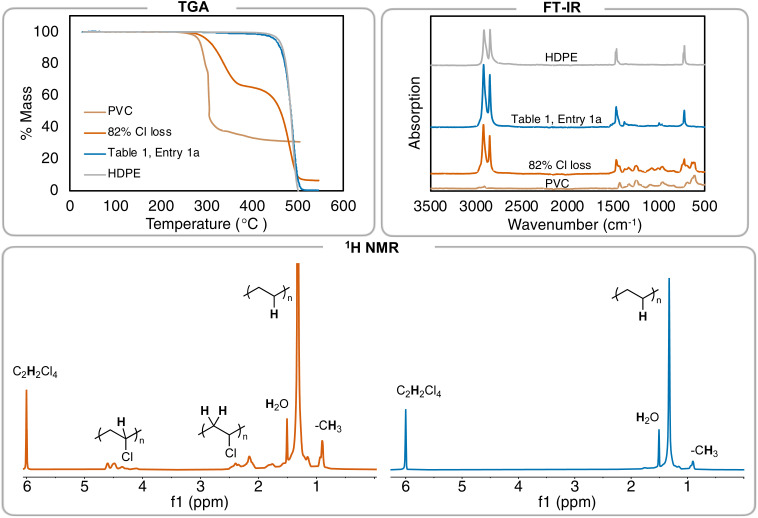
Summary of different methods to analyze branched PE formed from PVC. TGA of the resulting products (performed with a scan rate of 10 °C min^−1^). FT-IR spectra of varying amounts of PVC, PE, and the resulting 82% and >99% dechlorinated products. ^1^H NMR spectra of 82% dechlorinated PVC (left) *vs.*Table 1, entry 1 (right). Peak at 6.0 ppm is tetrachloroethane-d_2_.

Further investigation of the ^1^H NMR of the polymer product shows the C–H resonance of the C–Cl bond in PVC is insignificant and integrating it (relative to the CH_2_ repeat unit of the PE repeat unit) gives a >99% dechlorination value. Elemental analysis was also performed on the resulting PE product, in addition to pure high-density polyethylene (HDPE) from Sigma Aldrich, but even experimental data from pure PE was not in agreement with theoretical (Table S6†). Therefore, ^1^H NMR spectroscopy was primarily used to determine percent dechlorination in the resulting PE products from PVC. For this study, full dechorination is defined as >99%, which is when no C–H associated with the C–Cl of PVC can be observed in the ^1^H NMR and FT-IR spectra.^18^ Combination of ^1^H NMR spectroscopy, FT-IR spectroscopy, and thermogravimetric analysis (TGA) can all be used to distinguish chlorine still remaining in the polymer product (Fig. 2).

To ensure full dechlorination, the reaction was performed for 2 hours in slight excess of Et_3_SiH (1.2 equivalence to PVC) and a higher initiator loading (0.8 mol% *vs.* 0.5 mol%) to achieve no observable ^1^H NMR resonance ∼4.00 ppm (in d_2_-tetrachloroethane) (Table 1, entries 1a–b). FT-IR spectroscopy confirms near full dechlorination, as the vibration ∼600 cm^−1^ disappears.^35,36^^29^Si and ^19^F NMR spectroscopy were performed to confirm no contamination of the initiator and any by-products of the reaction (Fig. S61b and c†). A near quantitative isolated yield is achieved for this conversion of PVC to PE, which is a significant increase from other reports only able to achieve up to 69% yield of dechlorinated oils and waxes that are rid of Cl.^4^

**Table 1:** Conversion of poly(vinyl chloride) to branched polyethylene products

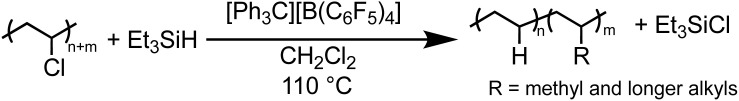										
Entry^a^	Time (h)	Init.^b^ (mol%)	Et_3_SiH^c^ (equiv.)	% Cl loss (^1^H NMR)^d^	B/1000 C^e^	*M*_n_ f (kDa)	*Đ* f	*T*_m_ g (°C)	*T*_d,5%_ h (°C)	Isolated yield (%)
1a	2	0.8	1.2	>99	49	13	2.4	56	444	>99
1b					57	12	3.1	50	450	81
2a	2	0.8	2.4	>99	47	21.8	3.1	67	443	69
2b					40	18.7	2.2	70	441	96
3a	2	0.8	3.6	>99	47	18	2.9	62	450	87
3b					46	27.1	2.6	68	445	84
4a	2	0.8	6	>99	41	24.3	2.7	81	453	89
4b					37	36.1	2.4	85	442	58
5a	2	0.8	12	>99	31	51.1	2.3	93	450	94
5b					36	40.1	2.7	90	444	34
6a	2	0.8	6	>99	48	13.3	2.3	67	449	>99
6b					39	21	2.5	82	446	69

a Entries 1a–2b and performed in 3 mL of CH_2_Cl_2_. Entries 2a–6b were performed in 9 mL of CH_2_Cl_2_.

b Initiator mol% relative to moles of PVC.

c Et_3_SiH equivalence relative to PVC.

d NMR performed in tetrachloroethane-d_2_ at 80 °C.

e Calculated by ^1^H NMR at 80 °C in tetrachlorethane-d_2_.

f Performed at 140 °C in trichlorobenzene. Mark–Houwink constants of HDPE (*k* = 3.23 × 10^2^ mL g^−1^, *a* = 0.735) were applied as a correction. Instrument calibrated with polystyrene standards using RI detector.^42^

g DSC performed at a scan rate of 10 °C min^−1^. *T*_m_ measured in second heating cycle.

h TGA performed and *T*_d,5%_ calculated from a heating rate of 10 °C min^−1^.

**Scheme 2:**
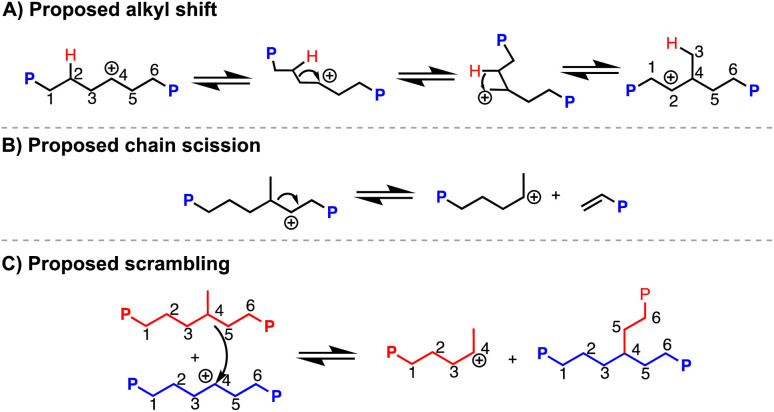
Proposed mechanisms of carbocation rearrangement (alkyl shift (A), chain scission (B), scrambling (C)) along polymer backbone in dechlorination of PVC mediated by silylium ions.

^1^H NMR analysis indicates 49 branches per 1000 carbons (49/1000 C),^37,38^ which is much higher than what PVC is suggested to have (∼2–4/1000 C) based on prior reports.^18,39^ This additional branching can be due to carbocation rearrangement of the PE chain, which would produce methyl (and other alkyl) branches along the polymer backbone (Scheme 2A).^40,41^^13^C NMR analysis of a resulting PE product (dechlorination = 94%) reveals 81% of branches are methyls, 14% are ethyls, and the remaining 5% are propyl and longer (Fig. S169†). Vibrations of branching in PE are also consistent with those reported in past literature.^43^ Hydrodechlorination using silylium ions on small molecules have exhibited carbocation rearrangement, supporting this can happen on larger molecules, such as organic polymers.^44^^1^H NMR spectroscopy also indicates a very minor presence of aromatic and alkene environments <1% compared to the CH_2_ repeat unit of PE. Alkene resonances can appear through chain scission events caused by carbocation rearrangements (Scheme 2B), and aromatic resonances can appear through further degradation involving the double bonds.^6,32,45^ Weak C=C frequencies ∼1500 cm^−1^ are also present in the FT-IR spectrum, supporting the chemical shifts seen in ^1^H NMR spectrum.^46^ Chain scission is further supported by the final polymer molar mass (*M*_n_) of 13.0 kDa, acquired through high temperature gel permeation chromatography (GPC), which is lower than the theoretical molar mass of 20.4 kDa if dechlorination without chain scission of PVC occurred. Chain scission is also consistent with the increase in dispersity (*Đ*) (2.4 from the original, 1.7 from the PVC starting material). Importantly, the molar mass numbers were calculated in the GPC software with Mark–Houwink correction factors that have been used in studies for the synthesis of branched PE. Scrambling of the carbocation between polymer chains is also possible (Scheme 2C).^47^ To test if chain scission was consistent between runs, a duplicate reaction was performed (Table 1, entry 1a *vs.* 1b). Only slight variation in the molar mass and dispersity of the resulting product can be seen. These results suggest that while carbonation rearrangements occur quickly, reasonable repeatability can be obtained.

**Fig. 3:**
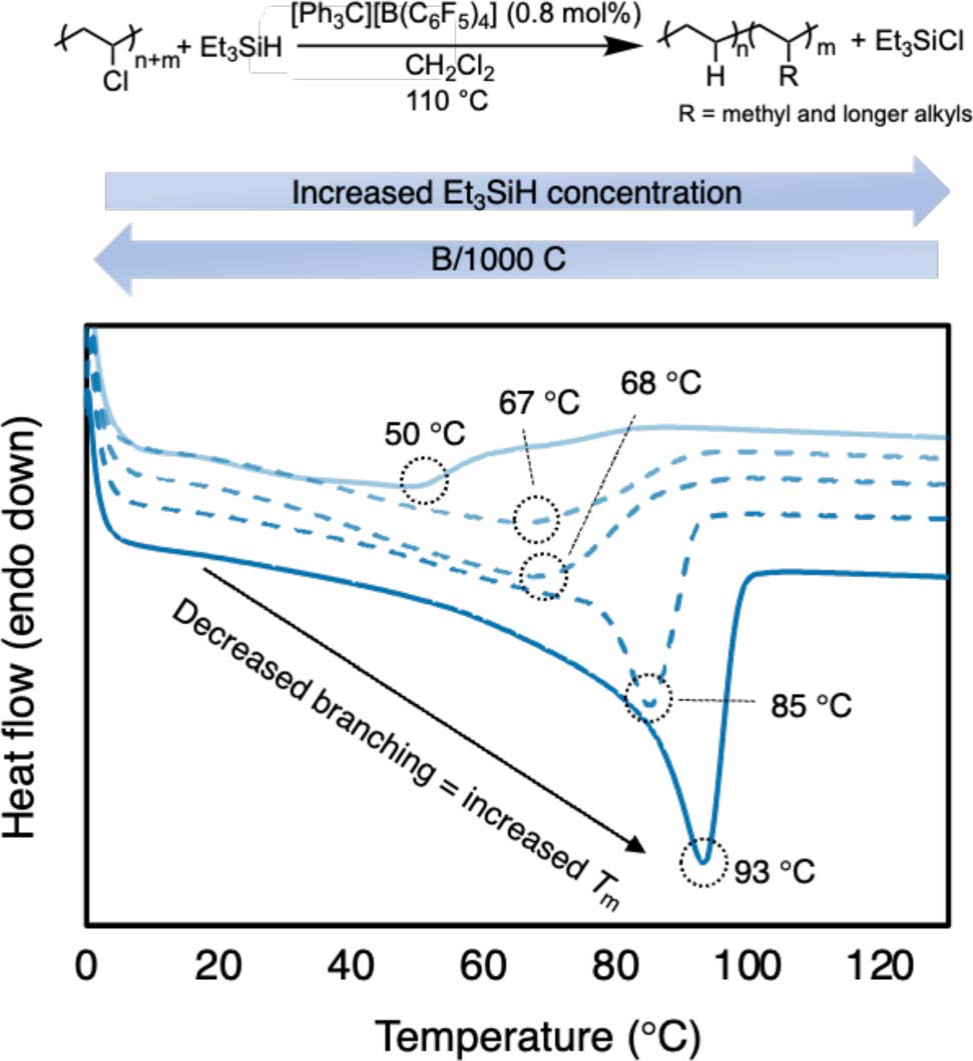
Plot of second heating curve of resulting branched PE products from Table 1 (entries 1b, 2a, 3b, 4b, and 5b). DSC traces offset on *y*-axis for clarity.

To confirm the branching content, differential scanning calorimetry (DSC) analysis was performed to measure the melting point of the PE product (Table 1 and Fig. 3). The resulting PE product has a melting point (*T*_m_) = 56 °C, which agrees with a relatively high branching content calculated by ^1^H NMR spectroscopy. For comparison, HDPE (Sigma Aldrich) has a *T*_m_ = 128 °C (Fig. S75†). The melting point depression is due to the short chain branches (which are likely placed randomly in this case) causing the PE to become less crystalline and more amorphous.^37,38,48^ When compared to reports with primarily methyl branching on the PE backbone, the *T*_m_ is lower than expected, further supporting the presence of longer alkyl branches (see ESI† for details). Regarding a duplicate run, the similar number of branches is achieved with the same conditions (57 B/1000 C) and a similar *T*_m_ as well (50 °C *vs.* 56 °C).

Additionally, the resulting PE product displays a *T*_d,5%_ = 444 °C, which is near virgin HDPE from Sigma Aldrich (*T*_d,5%_ = 457 °C) and matches that from other literature reports for PE polymers (Fig. 2A). A sample with 82% dechlorination by ^1^H NMR gives a *T*_d,5%_ = 302 °C, which is noticeably lower than the sample with >99% dechlorination. These data indicate that the *T*_d,5%_ of PE from PVC is a useful secondary measure measurement that can be used to see if Cl content is still in the polymer. TGA is secondary to NMR in quantifying the amount of Cl left, as a duplicate reaction (Table 1, entry 1b) that reached full dechlorination by ^1^H NMR has a *T*_d,5%_ of 450 °C, suggesting there is variability in the TGA of individual samples.

To tune the degree of branching in the resulting PE, it was hypothesized that performing the reaction in excess Et_3_SiH would favor hydride transfer from Et_3_SiH rather than carbocation rearrangement (Scheme 2). Indeed, as the Et_3_SiH loading was increased (Table 1, entries 2–5), the degree of branching was decreased. Satisfyingly, the branch content can be lowered to 31/1000 C (Table 1, entry 5a). The resulting PE displays a *T*_m_ of 93 °C. The increase in *T*_m_ with subtle decreases in branching content can likely be attributed to the presence of more methyl branches and fewer longer chain branches, which are known to impact the melt temperature more (Fig. 3).^49^ However, even at 12 equivalence of Et_3_SiH to PVC, carbocation rearrangement is still difficult to inhibit. This is likely due to carbocation rearrangement existing in a rapid equilibrium. Any reagents that can trap carbocations (*e.g.*, nucleophiles) will also likely trap the highly Lewis-acidic silylium ions, rendering the reaction dormant.^24,25^ It should be noted that the PVC materials were less soluble in conditions with higher Et_3_SiH content, requiring additional solvent (Table 1, entries 3–5).

Lowering the temperature of the reaction to 25 °C was also tried under more dilute conditions of Table 1, entries 1a–b (*e.g.*, 9 mL of CH_2_Cl_2_ was used instead of 3 mL). However, complete dechlorination was difficult to achieve, with only up to 87% being achieved even at longer reaction times of 72 hours (ESI Table S6,† entries 2a–b). Using the concentrated conditions, directly analogous to Table 1, entries 1a–b, and run for 18 hours at 25 °C, only one reaction reach full dechlorination. While full dechlorination was achieved in one run, only 92% dechlorination was observed in the second and third runs (Table S4,† entries 1a–c). This inconsistent conversion is hypothesized to be due to the insolubility of PVC and PE in CH_2_Cl_2_ at lower temperatures, and also being below the melting point of the resulting polymer products, making it difficult for silylium ions to reach all Cl atoms in the polymer backbone.

The initiator loading was also increased from 0.8 mol% to 1.6 mol%, and the branching content was subsequently increased from 31 to 40 B/1000 C (Table 1, entry 6). This increase in branching could be due to more cations being generated in solution, leading to more rearrangement.

Across duplicate reactions, the branching content, *T*_m_, and *T*_d,5%_ all stayed quite close to each other. The molar mass of the polymer did have variability between runs, which could indicate that chain scission reactions are not entirely controllable.

Dilution of the reaction from Table 1, entries 4a–b, did not change the branching in the product and therefore the *T*_m_ was the same (Table S3,† entries 5a–b). However, The ^1^H NMR spectrum of the dilute conditions indicates no arene or alkene environments, suggesting little chain scission happens with excess Et_3_SiH and dilute conditions.

Since Cl accounts for 57 wt% of PVC, it is important to determine its fate in these reactions. When the reaction from Table 1, entries 2a–b is characterized by ^1^H NMR spectroscopy before quenching, it can be seen that there is a resulting 50 : 50 mixture of Et_3_SiCl : Et_3_SiH in the reaction, with a minor presence of hydrolyzed products (Fig. S171†).^50^ This is exciting given that near quantitative conversion of Et_3_SiH to Et_3_SiCl is seen, which means that the Cl atoms are going to a valuable source in addition to the PVC backbone forming a valued product.

**Fig. 4:**
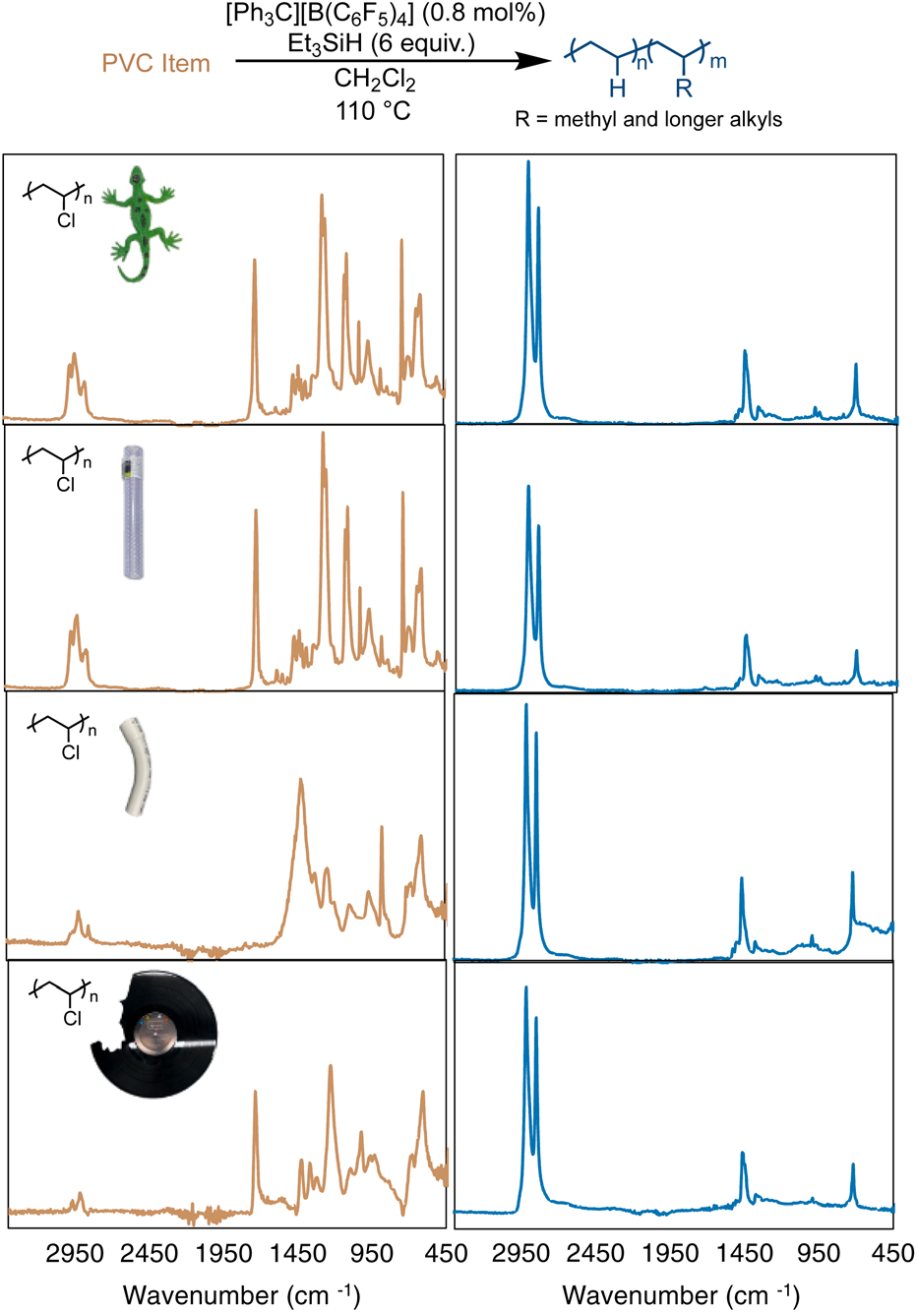
Dechlorination of a toy lizard, flexible PVC pipe, rigid PVC pipe, and used vinyl record (top-to-bottom), as measured by FT-IR spectroscopy.

In most cases, additives are considered a concern for the lifetime or stability of a catalyst. PVC is rarely used in its pure form commercially, as plasticizers (sometimes >50 wt% of the product) are added to give PVC properties for different applications.^51–54^ In particular, many additives have polar functional groups that could react with the silylium cations, triethyl silane or the trityl initial catalyst. Therefore, reactions were performed on four commercial items that varied in appearance and flexibility (including a colorful toy lizard, rigid PVC plumbing pipe, flexible and colorless PVC tubing, and a vinyl record, Fig. 4). Reactions were performed with the four items used without purification, other than to break the items into smaller pieces in a coffee grinder or with scissors. Using the same optimized conditions from Table 1, entries 4a–b (assuming the items were 100% PVC), near complete hydrodechlorination was realized for all four items (Table 2 and Fig. 4).

**Table 2:** Conversion of commercial PVC items to branched PE

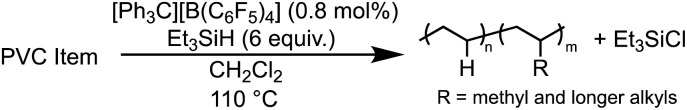					
PVC item	% Cl loss^a^ (^1^H NMR)	B/1000 C^b^	*M*_n_ c (*Đ*)	*T*_m_ d (°C)	*T*_d,5%_ e (°C)
Toy lizard	>99	53	9.5 (2.1)	65	431
Extracted toy lizard	>99	34	26.7 (4.1)	83 102	451
Rigid PVC pipe	98	42	26.9 (2.4)	82	434
Soft PVC pipe	>99	58	4.6 (3.2)	42	386
Vinyl record	>99	55	4.6 (2.1)	69	437
Extracted vinyl record	>99	55	4.1 (2.3)	76	441
Pure PVC^f^	>99	41	24.3 (2.7)	81	453

a NMR performed in tetrachloroethane-d_2_ at 80 °C.

b Calculated by ^1^H NMR spectroscopy at 80 °C in tetrachlorethane-d_2_.

c Performed at 140 °C in trichlorobenzene. Mark–Houwink constants of HDPE (*k* = 3.23 × 10^2^ mL g^−1^, *a* = 0.735) were applied as a correction. Instrument calibrated with polystyrene standards using RI detector.

d DSC performed at a scan rate of 10 °C min^−1^. *T*_m_ measured in second heating cycle.

e TGA performed and *T*_d,5%_ calculated from a heating rate of 10 °C min^−1^.

f Pure PVC from Table 1, entry 4a.

As seen in Fig. 4, all 4 PVC items were converted to PE-like products, based on FT-IR spectroscopy, with all items reaching >98% dechlorination by ^1^H NMR. This result was particularly exciting, as many of the PVC items showed a vibration at 1715 cm^−1^, which is indicative of a carbonyl stretching mode of an ester. For example, DEHP (di(2-ethylhexyl)phthalate) exhibits a carbonyl absorption also at 1725 cm^−1^.^55^^1^H NMR analysis (of the soluble fraction of the flexible PVC pipe) reveals one aromatic environment, indicative of a plasticizer more like bis(2-ethylhexyl) terephthalate (DEHT). As mentioned before, highly Lewis-acidic silylium ions are likely being formed in this reaction, so the observation that the reaction is not halted by the interaction of carbonyl and ester moieties of any plasticizers is unexpected. This is even more surprising given the fact that all the PVC items used in Fig. 4 were not purified or dried before the dechlorination, and likely retained some moisture. Surprisingly, the vinyl record showed no remaining Cl in NMR or FT-IR studies. Although rigid PVC pipe showed the least dechlorination (98.3%), the ability to achieve that high of dechlorination was unexpected, due to the large amount of additives and low solubility of this material. These results show that silylium ions are surprisingly robust to plasticizers and additives in commercial PVC items.

While near full dechlorination of almost all PVC items in Fig. 4 was achieved, the resulting polyethylene products differed quite greatly. Depending on the item selected, there were different degrees of calculated branching, with all items leading to more branching in the PE product than that of the pure PVC. Both the toy lizard and vinyl record produced PE materials with >50 branches per 1000 C, resulting in *T*_m_'s of 65 and 69, respectively. Rigid PVC pipe produced a PE product with 42 branches per 1000 C, leading to a higher *T*_m_ of 82 °C. A hypothesis for this observation is that there are a lot of additives by weight in rigid PVC pipe, making the Et_3_SiH loading higher than 6 equivalence, which was shown previously to decrease the branches in Fig. 3.

For comparison, PVC was extracted from the toy lizard and vinyl record, with additives separated, to identify if the additives influence the reaction. Upon using the same reaction conditions as Table 1, entries 4a–b (still assuming the materials are 100% PVC), near full dechlorination was observed for both purified PVC items. Notably, the vinyl record showed a slightly raised *T*_m_ measured by DSC (76 °C *vs.* 69 °C), yet the PVC extracted from the lizard showed a signficantly higher *T*_m_ (83 °C *vs.* 65 °C), when compared to the product from the item directly (Fig. S64 and 65†). The product from the reaction with the extracted PVC from the lizard also showed a second thermal transition at 102 °C, which could be due to insolubility of the PVC until a few hours into the reaction. These data suggest that depending on the PVC item being dechlorinated, the final properties of the polymer can be greatly impacted by additives/plasticizers present.

**Fig. 5:**
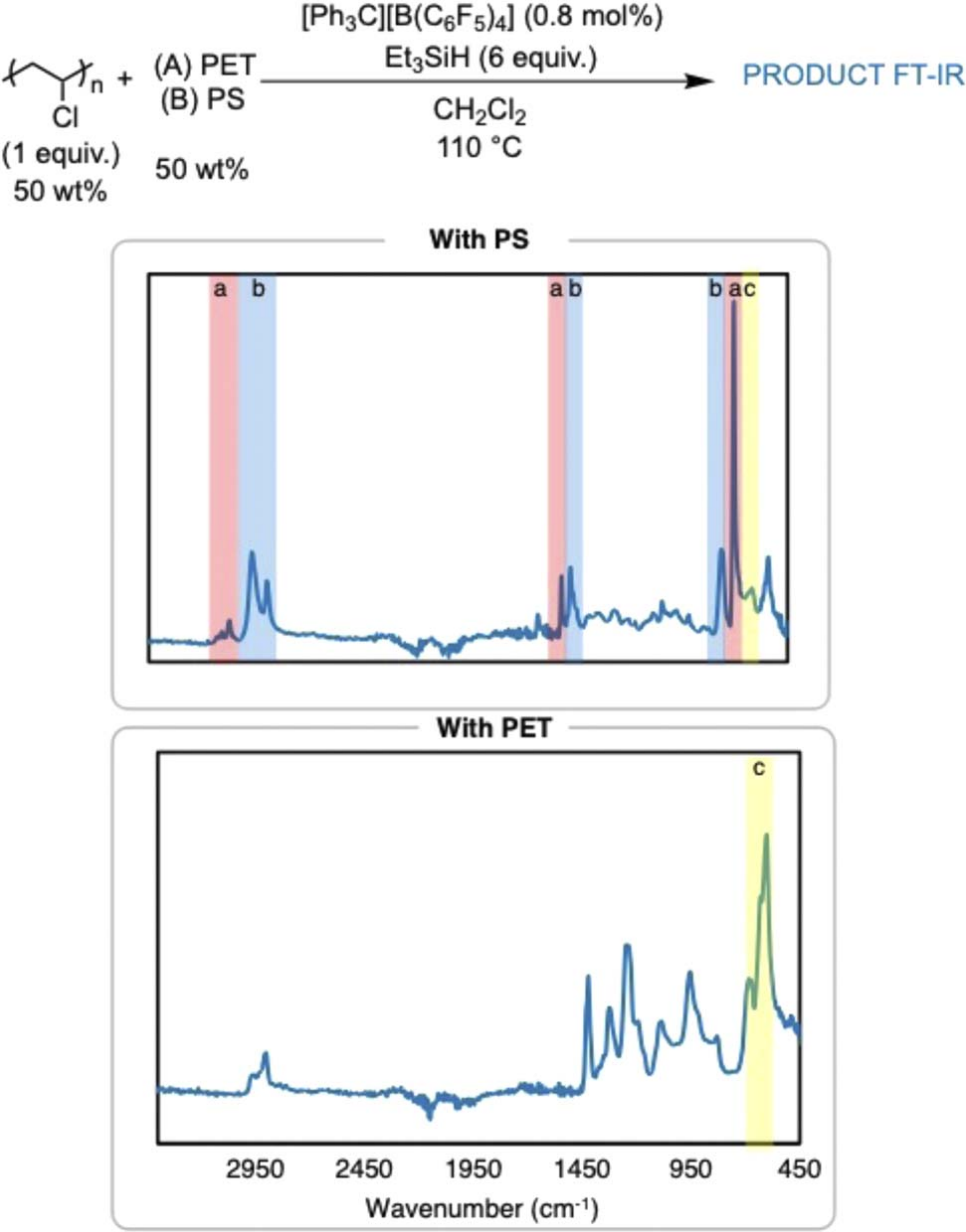
FT-IR spectra of PVC dechlorination in the presence of 50 wt% PET, and 50% PS (top-to-bottom): highlights are used to label (a) (red highlight) PS stretches, (b) (blue high-light) PE stretches, and (c) (yellow highlight) C–Cl stretch.

The dechlorination of PVC to PE in the presence of other plastics was also pursued in efforts to simulate what would happen in a plastic waste stream (Fig. 5). Dechlorination of PVC was attempted in the presence of poly(ethylene terephthalate) (PET) or PS to first test the compatibility of the reaction conditions with other plastics. The reaction in the presence of PET did not yield any dechlorination by FT-IR spectroscopy, which is hypothesized to be due to the high concentration of ester bonds in PET. This is surprising, as the method seemed to be robust to the ester-containing plasticizers in the commercial items. Dechlorination of PVC in the presence of PS yielded a very insoluble polymer product. FT-IR and ^1^H NMR analysis indicate presence of arenes in the polymer product, which supports cross linking between the C–C backbone of the PVC chain and the phenyl rings on PS, yielding cross-linked PS. This can be possible due to Friedel–Crafts alkylation of the carbocations generated on the PVC backbone, which is supported by past silylium ion chemistry on small molecules and PVC.^8^ Nonetheless, FT-IR and ^1^H NMR spectroscopy identify a significant loss of Cl in the presence of PS.

**Fig. 6:**
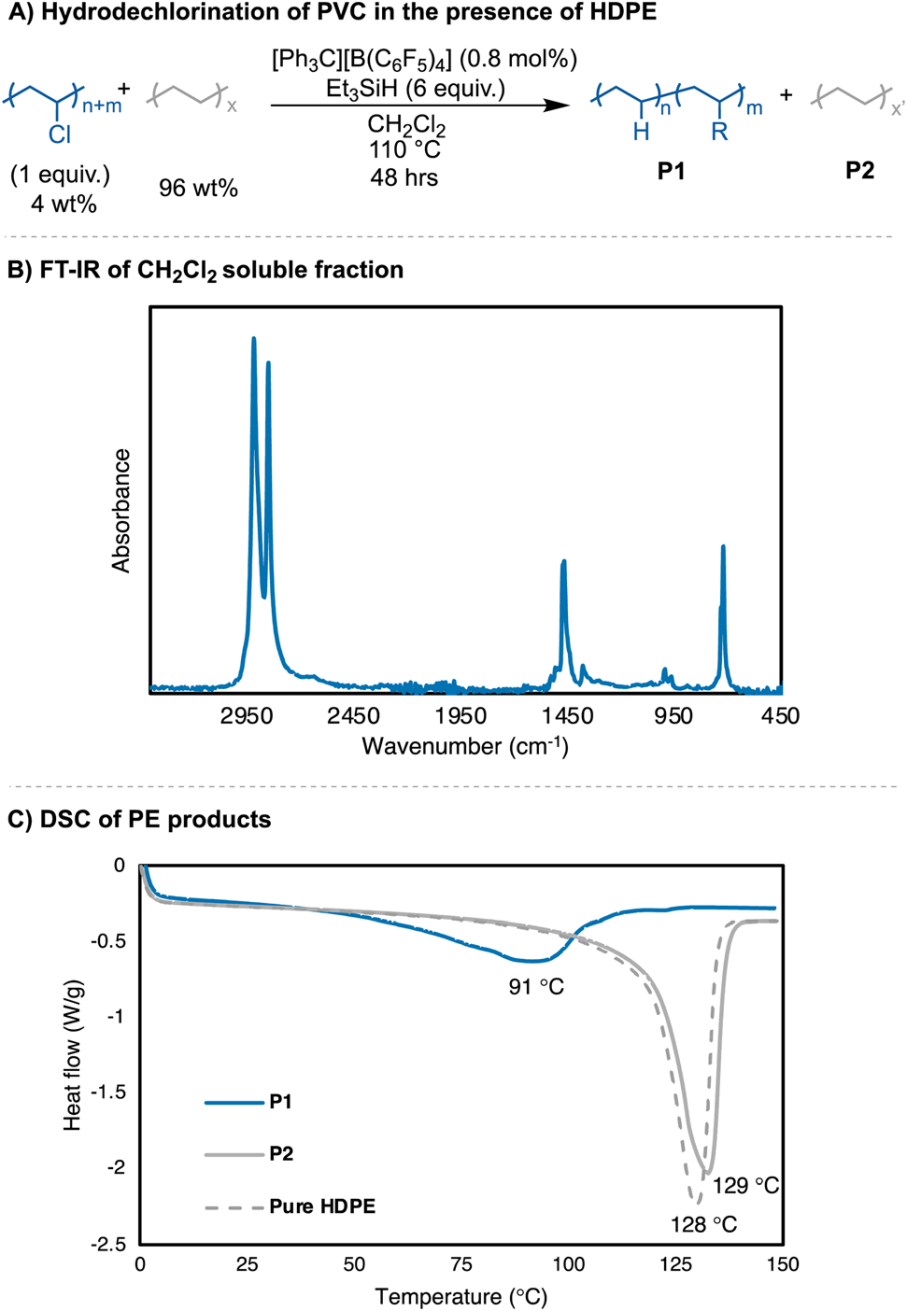
(A) Reaction conditions for PVC hydrodechlorination in the presence of HDPE; (B) FT-IR spectra of PE product; (C) DSC traces of PE products from mixed PVC/PE waste. CH_2_Cl_2_ soluble fraction has a *T*_m_ = 91 °C, with very little phase separation.

When dechlorination of PVC was attempted in the presence of PE (4 wt% of PVC, 96 wt% PE) to mimic the common impurity of PVC in PE waste, the reaction yielded near fully dechlorinated PVC (>99% Cl loss by ^1^H NMR) (Fig. 6). The reaction was performed in CH_2_Cl_2_, and the CH_2_Cl_2_ fraction was separated from the insoluble PE beads. The resulting CH_2_Cl_2_ fraction was purified (see ESI† for details), and the PE product was isolated in a 108% yield. In the reaction with pure PVC, a branching degree of 31 B/1000 C was observed and a *T*_m_ = 84 °C (Table 1, entry 4a). The PE achieved from the dechlorination in the presence of excess PE had a branching content of 32 B/1000 C, but a *T*_m_ = 91 °C. A very minor thermal transition at 123 °C is also observed in this sample. When the reaction conditions from Fig. 6 were replicated without the presence of HDPE beads, a similar molar mass and dispersity was achieved compared to Table 1, entries 4a–b. To probe whether the PE product and HDPE phase separates, PE from reaction Table 1, entry 4a was analyzed by DSC in the presence of HDPE (83 wt% *vs.* 17 wt%, respectively). The DSC trace shows two distinct melting points, with a *T*_m_ = 84 °C and *T*_m_ = 126 °C (Fig. S79†). These data show that the products obtained from mixed PVC/PE waste are separable, leading to pure PE products with different thermal properties. More importantly, the silylium ions generated *in situ* are able to dechlorinate PVC in reaction conditions that are dilute, which are like what recycling streams would see with small PVC impurities.

## Conclusions

We have demonstrated the ability to fully dechlorinate PVC to PE within two hours products by generating silylium ions *in situ*. By varying Et_3_SiH concentration, the degree of branching, and therefore thermal properties, can be tuned of the resulting PE product. No corrosive products are formed, with useful Et_3_SiCl being the chlorine-containing product, which is sold at a higher price than the silane precursor. Although not considered a great solvent for PVC or PE, dichloromethane was found to be the only solvent that could support dechlorination above 99%. Branched polyethylene products can be characterized fully, with thermal properties providing key insight into the degree and selectivity of hydrodechlorination. Importantly, this reaction is robust to common plasticizers and additives found in commercial PVC items, and full dechlorination can even occur in the presence of PE waste. This dechlorination strategy offers a controlled way of converting PVC contaminated PE waste to useful products that can be used for upcycled applications or also passed on to pyrolysis without the worry of corroding reactors, decomposing catalysts or altering the product distribution.

## Data availability

All relevant datasets supporting this article including general considerations for chemicals, solvents, reaction conditions and results, characterization data (including ^1^H, ^13^C, ^19^F, and ^29^Si NMR spectra, FT-IR spectra, DSC curves, and TGA curves) are available in the ESI.†

## Author contributions

Z. A. W., E. C. C., and A. N. N. contributed to experimental work, devising experiments, and writing the manuscript. M. E. F. directed the project, and helped write the manuscript. All authors discussed results.

## Conflicts of interest

The authors declare no competing financial interest.

## Supplementary Material

SC-015-D4SC00130C-s001
