# Supplementary material for: Conversion of waste poly(vinyl chloride) to branched polyethylene mediated by silylium ions
Source: Chem Sci. 2024 May 14;15(23):8766–74. doi: 10.1039/d4sc00130c (PMC11168076; doi:10.1039/d4sc00130c)
Supplement: SC-015-D4SC00130C-s001 [file SC-015-D4SC00130C-s001.pdf]

Electronic Supplementary Material (ESI) for Chemical Science.
This journal is © The Royal Society of Chemistry 2024

Electronic Supplementary Information for

Conversion of waste poly(vinyl chloride) to branched polyethylene mediated by silylium ions

Zachary A. Wood,[a] Eunice C. Castro,[a] Angelyn N. Nguyen,[a] and Megan E. Fieser*[a,b]

[a]Department of Chemistry, University of Southern California, Los Angeles, California 90089

[b]Wrigley Institute for Environmental Studies, University of Southern California, Los Angeles, California, 90089

**1.**	**General Considerations**	S7–S8
**2.**	**Experimental Procedures for PVC Dechlorination Results**	S9–15
**2.1**	**Synthesis of poly(ethylene) from low molecular weight PVC (Table 1, Entries 1a-b)**	S9
**2.2**	**Synthesis of poly(ethylene) from low molecular weight PVC (Table 1, Entries 2a-5b)**	S9
**2.3**	**Synthesis of poly(ethylene) from low molecular weight PVC (Table 1, Entries 6a-b)**	S9
**2.4**	**Synthesis of poly(ethylene) from PVC items (Table 2, Entries 1, 3-5)**	S9–S10
**2.5**	**Synthesis of poly(ethylene) from extracted PVC items (Table 2, Entries 2 and 6)**	S10
**2.6**	**Synthesis of poly(ethylene) from mixture of PVC and PET (Fig. 5)**	S10
**2.7**	**Synthesis of poly(ethylene) from mixture of PVC and PS (Fig. 5)**	S10–S11
**2.8**	**Synthesis of poly(ethylene) from mixture of PVC and HDPE (Fig. 5)**	S12
**2.9**	**Synthesis of poly(ethylene) from low molecular weight PVC under dilute conditions**	S12
**2.10**	**Extraction of PVC from PVC items**	S12
**2.11**	**Synthesis of partially chlorinated poly(ethylene) from low molecular weight PVC (Table S1, Entry 1)**	S11–S12
**2.12**	**Synthesis of poly(ethylene) from low molecular weight PVC at 25 °C (Table S4, Entry 1-3)**	S12
**2.13**	**Synthesis of poly(ethylene) from low molecular weight PVC at 25 °C (Table S4, Entry 4-5)**	S12
**2.14**	**Synthesis of poly(ethylene) from low molecular weight PVC at 110 °C (For ^{13}C analysis)**	S12–S13
**2.15**	**Safety Note**	S13
	**Table S1** Dechlorination of low molar mass PVC with 0.5 mol% $[CPh_3][B(C_6F_5)_4]$ loading	S13
	**Table S2** Partial dechlorination of PVC with substoichiometric Et_3SiH	S13
	**Table S3** Additional triplicate runs of PVC to branched PE	S14
	**Table S4** Conversion of PVC to PE at different temperatures	S14
	**Table S5** Screening for the conversion of PVC to PE in different solvents	S15
	**Table S6** Summary of Elemental Analysis Results	S15
**3.**	**FT-IR Spectroscopy**	S16–S35
	**Fig. S1** FT-IR spectrum of low molecular weight PVC	S16
	**Fig. S2** FT-IR spectrum of HDPE pellets	S16
	**Fig. S3** FT-IR spectrum of Table 1, Entry 1a	S17
	**Fig. S4** FT-IR spectrum of Table 1, Entry 1b	S17
	**Fig. S5** FT-IR spectrum of Table 1, Entry 2a	S18

Fig. S6 FT-IR spectrum of Table 1, Entry 2b S18
Fig. S7 FT-IR spectrum of Table 1, Entry 3a S19
Fig. S8 FT-IR spectrum of Table 1, Entry 3b S19
Fig. S9 FT-IR spectrum of Table 1, Entry 4a S20
Fig. S10 FT-IR spectrum of Table 1, Entry 4b S20
Fig. S11 FT-IR spectrum of Table 1, Entry 5a S21
Fig. S12 FT-IR spectrum of Table 1, Entry 5b S21
Fig. S13 FT-IR spectrum of Table 1, Entry 6a S22
Fig. S14 FT-IR spectrum of Table 1, Entry 6b S22
Fig. S15 FT-IR spectrum of PVC toy lizard item S23
Fig. S16 FT-IR spectrum of soft PVC pipe item S23
Fig. S17 FT-IR spectrum of rigid PVC pipe item S24
Fig. S18 FT-IR spectrum of vinyl record item S24
Fig. S19 FT-IR spectrum of Table 2, Entry 1 (PVC toy lizard item) S25
Fig. S20 FT-IR spectrum of Table 2, Entry 2 (extracted PVC toy lizard) S25
Fig. S21 FT-IR spectrum of Table 2, Entry 3 (rigid PVC pipe item) S26
Fig. S22 FT-IR spectrum of Table 2, Entry 4 (soft PVC pipe item) S26
Fig. S23 FT-IR spectrum of Table 2, Entry 5 (vinyl record item) S27
Fig. S24 FT-IR spectrum of Table 2, Entry 6 (extracted vinyl record item) S27
Fig. S25 FT-IR spectrum of extracted PVC toy lizard item S28
Fig. S26 FT-IR spectrum of product from PS-PVC mixture S28
Fig. S27 FT-IR spectrum of product from PET-PVC mixture S29
Fig. S28 FT-IR spectrum of Table S1, Entry 1 S29
Fig. S29 FT-IR spectrum of Table S2, Entry 1 S30
Fig. S30 FT-IR spectrum of Table S3, Entry 1 S30
Fig. S31 FT-IR spectrum of Table S3, Entry 2 S31
Fig. S32 FT-IR spectrum of Table S3, Entry 3 S31
Fig. S33 FT-IR spectrum of Table S3, Entry 4 S32
Fig. S34 FT-IR spectrum of Table S3, Entry 5a S32
Fig. S35 FT-IR spectrum of Table S3, Entry 5b S33
Fig. S36 FT-IR spectrum of Table S4, Entry 1a S33
Fig. S37 FT-IR spectrum of Table S4, Entry 1b S34
Fig. S38 FT-IR spectrum of Table S4, Entry 1c S34
Fig. S39 Representative FT-IR spectrum of Table S5, Entries 2-7 S35
4 **^{1}H NMR Spectroscopy** S36–S104
Fig. S40 ^{1}H NMR spectrum, in tetrachloroethane-d_2 (80 °C), of low molecular weight PVC S36
Fig. S41 ^{1}H NMR spectrum, in tetrachloroethane-d_2 (80 °C), of HDPE pellets S37
Fig. S41a Zoomed in ^{1}H NMR spectrum, in tetrachloroethane-d_2 (80 °C), of HDPE pellets S38
Fig. S42 ^{1}H NMR spectrum of tetrachloroethane-d_2 (25 °C) S39
Fig. S43 ^{1}H NMR spectrum, in tetrachloroethane-d_2 (80 °C), of polyethylene product in Table 1, Entry 1a S40
Fig. S43a Zoomed in ^{1}H NMR spectrum, in tetrachloroethane-d_2 (80 °C), of polyethylene product in Table 1, Entry 1a S41
Fig. S44 ^{1}H NMR spectrum, in tetrachloroethane-d_2 (80 °C), of polyethylene product in Table 1, Entry 1b S42
Fig. S44a Zoomed in ^{1}H NMR spectrum, in tetrachloroethane-d_2 (80 °C), of polyethylene product in Table 1, Entry 1b S43
Fig. S45 ^{1}H NMR spectrum, in tetrachloroethane-d_2 (80 °C), of polyethylene product in Table 1, Entry 2a S44
Fig. S45a Zoomed in ^{1}H NMR spectrum, in tetrachloroethane-d_2 (80 °C), of polyethylene product in Table 1, Entry 2a S45
Fig. S46 ^{1}H NMR spectrum, in tetrachloroethane-d_2 (80 °C), of polyethylene product in Table 1, Entry 2b S46
Fig. S46a Zoomed in ^{1}H NMR spectrum, in tetrachloroethane-d_2 (80 °C), of polyethylene product in Table 1, Entry 2b S47

Fig. S47 ^{1}H NMR spectrum, in tetrachloroethane-d_2 (80 °C), of polyethylene product in Table 1, Entry 3a S48
Fig. S47a Zoomed in ^{1}H NMR spectrum, in tetrachloroethane-d_2 (80 °C), of polyethylene product in Table 1, Entry 3a S49
Fig. S48 ^{1}H NMR spectrum, in tetrachloroethane-d_2 (80 °C), of polyethylene product in Table 1, Entry 3b S50
Fig. S48a Zoomed in ^{11}H NMR spectrum, in tetrachloroethane-d_2 (80 °C), of polyethylene product in Table 1, Entry 3b S51
Fig. S49 ^{1}H NMR spectrum, in tetrachloroethane-d_2 (80 °C), of polyethylene product in Table 1, Entry 4a S52
Fig. S49a Zoomed in ^{1}H NMR spectrum, in tetrachloroethane-d_2 (80 °C), of polyethylene product in Table 1, Entry 4a S53
Fig. S50 ^{1}H NMR spectrum, in tetrachloroethane-d_2 (80 °C), of polyethylene product in Table 1, Entry 4b S54
Fig. S50a Zoomed in ^{1}H NMR spectrum, in tetrachloroethane-d_2 (80 °C), of polyethylene product in Table 1, Entry 4b S55
Fig. S51 ^{11}H NMR spectrum, in tetrachloroethane-d_2 (80 °C), of polyethylene product in Table 1, Entry 5a S56
Fig. S51a Zoomed in ^{1}H NMR spectrum, in tetrachloroethane-d_2 (80 °C), of polyethylene product in Table 1, Entry 5a S57
Fig. S52 ^{1}H NMR spectrum, in tetrachloroethane-d_2 (80 °C), of polyethylene product in Table 1, Entry 5b S58
Fig. S52a Zoomed in ^{1}H NMR spectrum, in tetrachloroethane-d_2 (80 °C), of polyethylene product in Table 1, Entry 5b S59
Fig. S53 ^{1}H NMR spectrum, in tetrachloroethane-d_2 (80 °C), of polyethylene product in Table 1, Entry 6a S60
Fig. S53a Zoomed in ^{1}H NMR spectrum, in tetrachloroethane-d_2 (80 °C), of polyethylene product in Table 1, Entry 6a S61
Fig. S54 ^{1}H NMR spectrum, in tetrachloroethane-d_2 (80 °C), of polyethylene product in Table 1, Entry 6b S62
Fig. S54a Zoomed in ^{1}H NMR spectrum, in tetrachloroethane-d_2 (80 °C), of polyethylene product in Table 1, Entry 6b S63
Fig. S55 ^{1}H NMR spectrum, in tetrachloroethane-d_2 (80 °C), of polyethylene product in Table 2, Entry 1 (toy lizard) S64
Fig. S55a Zoomed in ^{1}H NMR spectrum, in tetrachloroethane-d_2 (80 °C), of polyethylene product in Table 2, Entry 1 (toy lizard) S65
Fig. S56 ^{11}H NMR spectrum, in tetrachloroethane-d_2 (80 °C), of polyethylene product in Table 2, Entry 2 (extracted toy lizard) S66
Fig. S56a Zoomed in ^{1}H NMR spectrum, in tetrachloroethane-d_2 (80 °C), of polyethylene product in Table 2, Entry 2 (extracted toy lizard) S67
Fig. S57 ^{1}H NMR spectrum, in tetrachloroethane-d_2 (80 °C), of polyethylene product in Table 2, Entry 3 (rigid PVC pipe) S68
Fig. S57a Zoomed in ^{1}H NMR spectrum, in tetrachloroethane-d_2 (80 °C), of polyethylene product in Table 2, Entry 3 (rigid PVC pipe) S69
Fig. S58 ^{1}H NMR spectrum, in tetrachloroethane-d_2 (80 °C), of polyethylene product in Table 2, Entry 4 (soft PVC pipe) S70
Fig. S58a Zoomed in ^{1}H NMR spectrum, in tetrachloroethane-d_2 (80 °C), of polyethylene product in Table 2, Entry 4 (soft PVC pipe) S71
Fig. S59 ^{1}H NMR spectrum, in tetrachloroethane-d_2 (80 °C), of polyethylene product in Table 2, Entry 5 (vinyl record) S72
Fig. S59a Zoomed in ^{1}H NMR spectrum, in tetrachloroethane-d_2 (80 °C), of polyethylene product in Table 2, Entry 5 (vinyl record) S73
Fig. S60 ^{1}H NMR spectrum, in tetrachloroethane-d_2 (80 °C), of polyethylene product in Table 2, Entry 6 (extracted vinyl record) S74
Fig. S60a Zoomed in ^{1}H NMR spectrum, in tetrachloroethane-d_2 (80 °C), of polyethylene product in Table 2, Entry 6 (extracted vinyl record) S75
Fig. S61 ^{1}H NMR spectrum, in $CDCl_3$ (25 °C), of polyethylene product in Table S1, Entry 1 S76
Fig. S61a Zoomed in ^{1}H NMR spectrum, in $CDCl_3$ (25 °C), of polyethylene product in Table S1, Entry 1 S77

Fig. S61b $^{19}F\{^1H\}$ NMR spectrum, in $CDCl_3$ (25 °C), of polyethylene product in Table S1, Entry 1 S78
Fig. S61c ^{29}Si NMR spectrum, in $CDCl_3$ (25 °C), of polyethylene product in Table S1, Entry 1 S79
Fig. S62 ^1H NMR spectrum, in tetrachloroethane-d_2 (80 °C), of partially dechlorinated polyethylene product in Table S2, Entry 1 S80
Fig. S63 ^1H NMR spectrum, in tetrachloroethane-d_2 (80 °C), of polyethylene product in Table S3, Entry 1 S81
Fig. S63a Zoomed in ^1H NMR spectrum, in tetrachloroethane-d_2 (80 °C), of polyethylene product in Table S3, Entry 1 S82
Fig. S64 ^1H NMR spectrum, in tetrachloroethane-d_2 (80 °C), of polyethylene product in Table S3, Entry 2 S83
Fig. S64a Zoomed in ^1H NMR spectrum, in tetrachloroethane-d_2 (80 °C), of polyethylene product in Table S3, Entry 2 S84
Fig. S65 ^1H NMR spectrum, in tetrachloroethane-d_2 (80 °C), of polyethylene product in Table S3, Entry 3 S85
Fig. S65a Zoomed in^1H NMR spectrum, in tetrachloroethane-d_2 (80 °C), of polyethylene product in Table S3, Entry 3 S86
Fig. S66 ^1H NMR spectrum, in tetrachloroethane-d_2 (80 °C), of polyethylene product in Table S3, Entry 4 S87
Fig. S66a Zoomed in ^1H NMR spectrum, in tetrachloroethane-d_2 (80 °C), of polyethylene product in Table S3, Entry 4 S88
Fig. S67 ^1H NMR spectrum, in tetrachloroethane-d_2 (80 °C), of polyethylene product in Table S3, Entry 5a S89
Fig. S67a Zoomed in ^1H NMR spectrum, in tetrachloroethane-d_2 (80 °C), of polyethylene product in Table S3, Entry 5a S90
Fig. S68 ^1H NMR spectrum, in tetrachloroethane-d_2 (80 °C), of polyethylene product in Table S3, Entry 5b S91
Fig. S68a Zoomed in ^1H NMR spectrum, in tetrachloroethane-d_2 (80 °C), of polyethylene product in Table S3, Entry 5b S92
Fig. S69 ^1H NMR spectrum, in tetrachloroethane-d_2 (80 °C), of polyethylene product in Table S4, Entry 1a S93
Fig. S69a Zoomed in ^1H NMR spectrum, in tetrachloroethane-d_2 (80 °C), of polyethylene product in Table S4, Entry 1a S94
Fig. S70 ^1H NMR spectrum, in tetrachloroethane-d_2 (80 °C), of polyethylene product in Table S4, Entry 1b S95
Fig. S70a Zoomed in ^1H NMR spectrum, in tetrachloroethane-d_2 (80 °C), of polyethylene product in Table S4, Entry 1b S96
Fig. S71 ^1H NMR spectrum, in tetrachloroethane-d_2 (80 °C), of polyethylene product in Table S4, Entry 1c S97
Fig. S71a Zoomed in ^1H NMR spectrum, in tetrachloroethane-d_2 (80 °C), of polyethylene product in Table S4, Entry 1c S98
Fig. S72 ^1H NMR spectrum, in tetrachloroethane-d_2 (80 °C), of polyethylene product in Table S4, Entry 2a S99
Fig. S72a Zoomed in ^1H NMR spectrum, in tetrachloroethane-d_2 (80 °C), of polyethylene product in Table S4, Entry 2a S100
Fig. S73 ^1H NMR spectrum, in tetrachloroethane-d_2 (80 °C), of polyethylene product in Table S4, Entry 2b S101
Fig. S73a Zoomed in ^1H NMR spectrum, in tetrachloroethane-d_2 (80 °C), of polyethylene product in Table S4, Entry 2b S102
Fig. S74 ^1H NMR spectrum, in $CDCl_3$ (25 °C), of chloroform soluble fraction of flexible PVC tubing S103
Fig. S75 ^1H NMR spectrum, in tetrachloroethane-d_2 (80 °C), of product from PS-PVC mixture S104
5 Differential Scanning Calorimetry (DSC) S105-S122
Fig. S76 DSC (2nd heating curve) of Table 1, Entry 1a S105
Fig. S77 DSC (2nd heating curve) of Table 1, Entry 1b S105
Fig. S78 DSC (2nd heating curve) of Table 1, Entry 2a S106
Fig. S79 DSC (2nd heating curve) of Table 1, Entry 2b S106
Fig. S80 DSC (2nd heating curve) of Table 1, Entry 3a S107

Fig. S81 DSC (2nd heating curve) of Table 1, Entry 3b S107
Fig. S82 DSC (2nd heating curve) of Table 1, Entry 4a S108
Fig. S83 DSC (2nd heating curve) of Table 1, Entry 4b S108
Fig. S84 DSC (2nd heating curve) of Table 1, Entry 5a S109
Fig. S85 DSC (2nd heating curve) of Table 1, Entry 5b S109
Fig. S86 DSC (2nd heating curve) of Table 1, Entry 6a S110
Fig. S87 DSC (2nd heating curve) of Table 1, Entry 6b S110
Fig. S88 DSC (2nd heating curve) of Table 2, toy lizard (product) S111
Fig. S89 DSC (2nd heating curve) of Table 2, extracted toy lizard (product) S111
Fig. S90 DSC (2nd heating curve) of Table 2, rigid PVC pipe (product) S112
Fig. S91 DSC (2nd heating curve) of Table 2, soft PVC pipe (product) S112
Fig. S92 DSC (2nd heating curve) of Table 2, vinyl record (product) S113
Fig. S93 DSC (2nd heating curve) of Table 2, extracted vinyl record (product) S113
Fig. S94 DSC (2nd heating curve) of HDPE pellets (CAS: 9002-88-4, Sigma Aldrich) S114
Fig. S95 DSC (2nd heating curve) of low molecular weight S114
Fig. S96 DSC (2nd heating curve) of CH_2Cl_2 soluble fraction from the mixed PE/PVC reaction S115
Fig. S97 DSC (2nd heating curve) of CH_2Cl_2 insoluble fraction from the mixed PE/PVC reaction S115
Fig. S98 DSC (2nd heating curve) of mixed Table 1, Entry 4a and HDPE (83/17 wt%) S116
Fig. S99 DSC (2nd heating curve) of Table S1, Entry 1 S116
Fig. S100 DSC (2nd heating curve) of Table S2, Entry 1 S117
Fig. S101 DSC (2nd heating curve) of Table S3, Entry 1 S117
Fig. S102 DSC (2nd heating curve) of Table S3, Entry 2 S118
Fig. S103 DSC (2nd heating curve) of Table S3, Entry 3 S118
Fig. S104 DSC (2nd heating curve) of Table S3, Entry 4 S119
Fig. S105 DSC (2nd heating curve) of Table S3, Entry 5a S119
Fig. S106 DSC (2nd heating curve) of Table S3, Entry 5b S120
Fig. S107 DSC (2nd heating curve) of Table S4, Entry 1a S120
Fig. S108 DSC (2nd heating curve) of Table S4, Entry 1b S121
Fig. S109 DSC (2nd heating curve) of Table S4, Entry 1c S121
Fig. S110 Summary of melting point temperatures for branched PE products with varied branching S122
6 Thermogravimetric Analysis (TGA) S123–S137
Fig. S111 TGA curve of low molecular weight PVC S123
Fig. S112 TGA curve of HDPE pellets S123
Fig. S113 TGA curve of Table 1, Entry 1a S124
Fig. S114 TGA curve of Table 1, Entry 1b S124
Fig. S115 TGA curve of Table 1, Entry 2a S125
Fig. S116 TGA curve of Table 1, Entry 2b S125
Fig. S117 TGA curve of Table 1, Entry 3a S126
Fig. S118 TGA curve of Table 1, Entry 3b S126
Fig. S119 TGA curve of Table 1, Entry 4a S127
Fig. S120 TGA curve of Table 1, Entry 4b S127
Fig. S121 TGA curve of Table 1, Entry 5a S128
Fig. S122 TGA curve of Table 1, Entry 5b S128
Fig. S123 TGA curve of Table 1, Entry 6a S129
Fig. S124 TGA curve of Table 1, Entry 6b S129
Fig. S125 TGA curve of Table 2, Entry 1 S130
Fig. S126 TGA curve of Table 2, Entry 2 S130
Fig. S127 TGA curve of Table 2, Entry 3 S131
Fig. S128 TGA curve of Table 2, Entry 4 S131
Fig. S129 TGA curve of Table 2, Entry 5 S132
Fig. S130 TGA curve of Table 2, Entry 6 S132
Fig. S131 TGA curve of Table S1, Entry 1 S133
Fig. S132 TGA curve of Table S2, Entry 1 S133

	**Fig. S133** TGA curve of Table S3, Entry 1	S134
	**Fig. S134** TGA curve of Table S3, Entry 2	S134
	**Fig. S135** TGA curve of Table S3, Entry 3	S135
	**Fig. S136** TGA curve of Table S3, Entry 4	S135
	**Fig. S137** TGA curve of Table S3, Entry 5a	S136
	**Fig. S138** TGA curve of Table S4, Entry 1a	S136
	**Fig. S139** TGA curve of Table S4, Entry 1b	S137
	**Fig. S140** TGA curve of Table S4, Entry 1c	S137
**7**	**Gel Permeation Chromatography (GPC)**	S138–S152
	**Table S7** Table of molar mass data for PVC items, analyzed via light scattering signal and eluted with THF	S138
	**Fig. S141** GPC traces corresponding to low molecular weight PVC in Table S4, Entry 1	S138
	**Fig. S142** GPC traces corresponding to PVC, M_n = 35 kDa in Table S4, Entry 2	S139
	**Fig. S143** GPC traces corresponding to PVC, M_n = 47 kDa in Table S4, Entry 3	S139
	**Fig. S144** GPC traces corresponding to PVC, M_n = 99 kDa in Table S4, Entry 4	S140
	**Fig. S145** GPC traces corresponding to PVC toy lizard item in Table S4, Entry 5	S140
	**Fig. S146** GPC traces corresponding to PVC vinyl record item in Table S4, Entry 6	S141
	**Fig. S147** GPC traces corresponding to rigid PVC pipe item in Table S4, Entry 7	S141
	**Fig. S148** GPC traces corresponding to flexible PVC pipe item in Table S4, Entry 8	S142
	**Fig. S149** GPC trace corresponding to Table 1, Entry 1a	S142
	**Fig. S150** GPC trace corresponding to Table 1, Entry 1b	S143
	**Fig. S151** GPC trace corresponding to Table 1, Entry 2a	S143
	**Fig. S152** GPC trace corresponding to Table 1, Entry 2b	S144
	**Fig. S153** GPC trace corresponding to Table 1, Entry 3a	S144
	**Fig. S154** GPC trace corresponding to Table 1, Entry 3b	S145
	**Fig. S155** GPC trace corresponding to Table 1, Entry 4a	S145
	**Fig. S156** GPC trace corresponding to Table 1, Entry 4b	S146
	**Fig. S157** GPC trace corresponding to Table 1, Entry 5a	S146
	**Fig. S158** GPC trace corresponding to Table 1, Entry 5b	S147
	**Fig. S159** GPC trace corresponding to Table 1, Entry 6a	S147
	**Fig. S160** GPC trace corresponding to Table 1, Entry 6b	S148
	**Fig. S161** GPC trace corresponding to Table 2, Entry 1	S148
	**Fig. S162** GPC trace corresponding to Table 2, Entry 2	S149
	**Fig. S163** GPC trace corresponding to Table 2, Entry 3	S149
	**Fig. S164** GPC trace corresponding to Table 2, Entry 4	S150
	**Fig. S165** GPC trace corresponding to Table 2, Entry 5	S150
	**Fig. S166** GPC trace corresponding to Table 2, Entry 6	S151
	**Fig. S167** GPC trace corresponding to Table S3, Entry 5b	S151
	**Fig. S168** GPC trace corresponding to Table S4, Entry 1	S152
**8**	**^{13}C and ^{1}H NMR Spectroscopy for calculating branching**	S153
	**Fig. S169** $^{13}C\{^{1}H\}$ NMR spectrum of PVC to PE sample (in tetrachlorethane-d_2) using conditions from Table S1, Entry 4a	S153
	**Fig. S170** ^{1}H NMR spectrum of PVC to PE sample (in tetrachlorethane-d_2) using conditions from Table S1, Entry 4a	S153
**9**	**Control reactions for activation of CH_2Cl_2 by silylium ions**	S154
	**Table S8** Pressure of Parr Reactor vessels before, during, and after reactions	S154
**10**	**Conversion of Et_3SiH to Et_3SiCl monitored by ^{1}H NMR**	S155
	**Fig. S171** ^{1}H NMR spectrum of aliquot for Et_3SiH to Et_3SiCl (in $CDCl_3$) using conditions from Table S1, Entry 2a-b.	S155
**11**	**References**	S156

1. General Considerations

All reactions were performed on the benchtop with no further purification of reagents unless otherwise noted. Poly(vinyl chloride) (PVC) (Aldrich, low molecular weight, Product #: 81388; M_n = 35 kDa, M_w = 62 kDa, Product #: 189588; M_n = 47 kDa, M_w = 80 kDa, Product #: 389232; M_n = 99 kDa, M_w= 233 kDa, Product #: 346764) were used as received. Triphenylmethylium terakis(pentaflouorophenyl)borate ($[Ph_3C][B(C_6F_5)_4]$) was purchased from Fischer Chemical (Fisher, Product #: T28635G) and anhydrous grade triethyl silane (Et_3SiH) (Aldrich. Product #: 230197) was purchased from Aldrich and used as received. ($[Ph_3C][B(C_6F_5)_4]$) stored in a glove box under an N_2 atmosphere, and transferred to a desiccator 2 hours – 5 days prior to use. Et_3SiH was stored in a glove box under N_2 atmosphere and transferred to a 20 mL vial XXXft h used within 5 days for all reactions. $CDCl_3$ was purchased from Cambridge Isotope Laboratories and tetrachloroethane-d_2 was purchased from Fischer Chemical. Toy PVC lizard was purchased from Amazon. Toy PVC lizard was ground to a powder using a blender and dried under reduced pressure at 110 °C for 24 hours prior to use. Rigid and soft PVC pipes were purchased from Home Depot. Vinyl record was purchased from a used goods store. Rigid PVC pipe, flexible PVC pipe, and vinyl record were cut into small pieces prior to reactions.

^1H and $^{13}C\{^1H\}$ NMR spectra were recorded on a Varian 500-MR 2-Channel NMR spectrometer, Varian VNMRS-600 3-Channel NMR spectrometer, or collected at a Bruker Instruments facility. All NMRs referenced against residual solvent resonances. FT-IR spectra were recorded on an Agilent Cary 630 FTIR spectrometer equipped with a single reflection diamond (Di) ATR module. Polymer molar masses and dispersities of PE products, were determined using an Agilent 1260 Infinity II High Temperature GPC instrument (equipped with 3 columns in series and a guard column) calibrated with monodisperse polystyrene standards at 140 °C (flow rate = 1.0 mL/min) in 1,2,4-trichlorobenzene. Mark-Houwink corrections based on HDPE ($k = 3.23 \times 10^2$ ml/g, α = 0.735). [1] Polymer molar masses and dispersities for PVC materials were determined using a SEC-MALS instrument equipped with an Agilent 1260 Infinity II HPLC System

and autosampler, 2 Agilent PolyPore columns (both 5 micron, 4.6 mm ID) in sequence, a Wyatt DAWN HELEOS-II light scattering detector, and a Wyatt Optilab TrEX refractive index detector. The columns were eluted with HPLC grade THF at 30 °C at a flow rate of 0.3 mL/min, and polymer samples were dissolved in this solvent and filtered through a 0.2 micron PTFE membrane before SEC-MALS (size exclusion chromatography multi-angle light scattering) analyses. Dn/dc values were calculated from the RI signal by using the 100% mass recovery method in the Astra software and a known sample concentration. Thermogravimetric analyses (TGA) were carried out on a Mettler Toledo TGA/DSC+ instrument at a heating rate of 10 °C/min and a dinitrogen flow rate of 40 ml/min. Elemental analyses were carried out on a Thermo Flash 2000 Elemental Analyzer. DSC traces were recorded using a TA Instruments Discovery DSC 250 Auto, equipped with a Discovery LN Pump, and processed with TRIOS software. The DSC measurements were made at a heating rate of 10 °C/min and N_2 flow rate of 20 ml/min, and T_m values were obtained from the peak XXXft he melting transition in the second heating curve. Parr reactions were performed in a Parr MRS 5000 Multireactor containing 75 mL total volume vessels with glass liners and PTFE-coated stir bars.

Yield of polyethylene products were calculated as follows in Equation 1:

$$(1)\ \%\ Yield = \frac{experimental\ mass}{mmol(PE) * (MW_{PE}) + mmol(PVC) * (MW_{PVC})} * 100$$

Branching calculated using Equation 2[1]:

$$(2)\ Branches\ per\ 1000\ C = \frac{\left(\frac{integration\ CH_3}{3}\right)}{\left(\frac{integration\ CH_3 +\ CH_2 +\ CH}{2}\right)} * 1000$$

%Cl loss calculated using Equation 3:

$$(3)\ \%\ Cl\ loss = 1 - \frac{integration\ 4 - 4.5\ ppm}{\left(\frac{integration\ 1 - 1.5\ ppm}{4}\right)} * 100$$

Experimental Procedures

2. General Procedures for PVC Dechlorination Results

2.1 Synthesis of poly(ethylene) from low molecular weight PVC (Table 1, Entry 1a-b): ($[Ph_3C][B(C_6F_5)_4]$) (16.8 mg, 0.018 mmol, 0.8 mol%), Et_3SiH (0.42 mL, 2.63 mmol, 1.2 equiv.) CH_2Cl_2 (3 mL) and a stir bar were charged to a 2 dram vial. The vial was then stirred for 5 minutes. PVC (136 mg, 2.176 mmol, 1 equiv.) was weighed into a 2 dram vial equipped with a Teflon-coated cap. The CH_2Cl_2 mixture was then added to the PVC, and the vial was sealed with Teflon tape along the inside of the cap, and electrical tape on the outside. The vial was then placed inside a Chemglass high-throughput tray that was preheated to 110 °C. After 2 hours, the reaction mixture was quenched with methanol (~ 15 mL), which resulted in the precipitation of white solids. The mixture was then filtered and washed with methanol (~5 mL) and $CHCl_3$ (~1 mL) three times. The polymer was then dried at 60 °C for 18 hours.

2.2 Synthesis of poly(ethylene) from low molecular weight PVC (Table 1, Entries 2a-5b): ($[Ph_3C][B(C_6F_5)_4]$) (16.8 mg, 0.018 mmol, 0.8 mol%), Et_3SiH (0.84 mL – 4.2 mL, 5.26 – 26.3 mmol, 2.4 – 12 equiv.) CH_2Cl_2 (9 mL) and a stir bar were charged to a 2 dram vial. The vial was then stirred for 5 minutes. PVC (136 mg, 2.176 mmol, 1 equiv.) was weighed into a 16 mL vial equipped with a Teflon-coated cap. The CH_2Cl_2 mixture was then added to the PVC, and the vial was sealed with Teflon tape along the inside of the cap, and electrical tape on the outside. The vial was then placed inside a Chemglass high-throughput tray that was preheated to 110 °C. After 2 hours, the reaction mixtures were quenched with methanol (~ 15 mL), which resulted in the precipitation of white solids. The mixture was then filtered and washed with methanol (~5 mL) and $CHCl_3$ (~1 mL) three times. The polymer was then dried at 60 °C for 18 hours.

2.3 Synthesis of poly(ethylene) from low molecular weight PVC (Table 1, Entry 6a-b): ($[Ph_3C][B(C_6F_5)_4]$) (33.6 mg, 0.036 mmol, 1.6 mol%), Et_3SiH (2.1 mL, 13.2 mmol, 6 equiv.) CH_2Cl_2 (9 mL) and a stir bar were charged to a 2 dram vial. The vial was then stirred for 5 minutes. PVC (136 mg, 2.176 mmol, 1 equiv.) was weighed into a 16 mL vial equipped with a Teflon-coated cap. The CH_2Cl_2 mixture was then added to the PVC, and the vial was sealed with Teflon tape along the inside of the cap, and electrical tape on the outside. The vial was then placed inside a Chemglass high-throughput tray that was preheated to 110 °C. After 4 hours, the reaction mixture was quenched with methanol (~ 15 mL), which resulted in the precipitation of white solids. The mixture was then filtered and washed with methanol (~5 mL) and $CHCl_3$ (~1 mL) three times. The polymer was then dried at 60 °C for 18 hours.

Synthesis of poly(ethylene) from PVC items (Table 2, Entries 1, 3-5): ($[Ph_3C][B(C_6F_5)_4]$) (16.8 mg, 0.018 mmol, 0.8 mol%), Et_3SiH (2.1 mL, 13.2 mmol, 6 equiv.) CH_2Cl_2 (6 mL) and a stir bar were charged to a 16 mL vial. The vial was then stirred for 5 minutes. A PVC item (136 mg, 2.176 mmol, 1 equiv.) was weighed into a 16 mL vial equipped with a Teflon-coated cap. The CH_2Cl_2 mixture was then added to the PVC, and the vial was sealed with Teflon tape along the inside of the cap, and electrical tape on the outside. The vial was then placed inside a Chemglass high-throughput tray that was preheated to 110 °C. After 24 hours, the reaction mixtures were quenched with methanol (~ 15 mL), which resulted in the precipitation of white solids. The mixture was then filtered and washed with methanol (~5 mL) three times. The polymer was then dried at 60 °C for 18 hours. Yields of product are calculated with the assumption the PVC item is 100 wt% PVC. **Table 2, Entry 1:** 60% yield; **Table 2, Entry 3:** 78% yield; **Table 2, Entry 4:** 51% yield; **Table 2, Entry 5:** 33% yield.

2.4 **Synthesis of poly(ethylene) from extracted PVC items (Table 2, Entries 2 and 6):** ($[Ph_3C][B(C_6F_5)_4]$) (16.8 mg, 0.018 mmol, 0.8 mol%), Et_3SiH (2.1 mL, 13.2 mmol, 6 equiv.) CH_2Cl_2 (6 mL) and a stir bar were charged to a 16 mL vial. The vial was then stirred for 5 minutes. PVC, extracted from a PVC item, (136 mg, 2.176 mmol, 1 equiv.) was weighed into a 16 mL vial equipped with a Teflon-coated cap. The CH_2Cl_2 mixture was then added to the PVC, and the vial was sealed with Teflon tape along the inside of the cap, and electrical tape on the outside. The vial was then placed inside a Chemglass high-throughput tray that was preheated to 110 °C. After 24 hours, the reaction mixture was quenched with methanol (~ 15 mL), which resulted in the precipitation of white solids. The mixture was then filtered and washed with methanol (~5 mL) three times. The polymer was then dried at 60 °C for 18 hours. Yields of product are calculated with the assumption the extracted material is 100 wt% PVC. **Table 2, Entry 2:** 89% yield; **Table 2, Entry 6:** 58% yield.

2.5 **Synthesis of poly(ethylene) from mixture of PVC and PET (Fig. 5):** ($[Ph_3C][B(C_6F_5)_4]$) (16.8 mg, 0.018 mmol, 0.8 mol%), Et_3SiH (2.1 mL, 13.2 mmol, 6 equiv.) CH_2Cl_2 (6 mL) and a stir bar were charged to a 16 mL vial. The vial was then stirred for 5 minutes. Low molecular weight PVC (137.8 mg, 2.176 mmol, 1 equiv.) and PET (137.8 mg, 1.0 mmol, 0.48 equiv.) was weighed into a 16 mL vial equipped with a Teflon-coated cap. The CH_2Cl_2 mixture was then added to the PVC, and the vial was sealed with Teflon tape along the inside of the cap, and electrical tape on the outside. The vial was then placed inside a Chemglass high-throughput tray that was preheated to 110 °C. After 24 hours, the reaction mixture was quenched with methanol (~ 15 mL), which resulted in the precipitation of white solids. The mixture was then filtered and washed with methanol (~5 mL) three times. The polymer was then dried at 60 °C for 18 hours.

2.6 **Synthesis of poly(ethylene) from mixture of PVC and PS (Fig. 5):** ($[Ph_3C][B(C_6F_5)_4]$) (16.8 mg, 0.018 mmol, 0.8 mol%), Et_3SiH (2.1 mL, 13.2 mmol,

6 equiv.) CH_2Cl_2 (6 mL) and a stir bar were charged to a 2 dram vial. The vial was then stirred for 5 minutes. PVC (136.3 mg, 2.176 mmol, 1 equiv.) and PS (136.3 mg, 1.3 mmol, 0.60 equiv.) was weighed into a 16 mL vial equipped with a Teflon-coated cap. The CH_2Cl_2 mixture was then added to the PVC, and the vial was sealed with Teflon tape along the inside of the cap, and electrical tape on the outside. The vial was then placed inside a Chemglass high-throughput tray that was preheated to 110 °C. After 2 hours, the reaction mixture was quenched with methanol (~ 15 mL), which resulted in the precipitation of white solids. The mixture was then filtered and washed with methanol (~5 mL) and $CHCl_3$ (~1 mL) three times. The polymer was then dried at 60 °C for 18 hours.

2.7 Synthesis of poly(ethylene) from mixture of PVC and HDPE (Fig. 5): ($[Ph_3C][B(C_6F_5)_4]$) (16.9 mg, 0.018 mmol, 0.8 mol%), Et_3SiH (2.1 mL, 13.2 mmol, 6 equiv.) CH_2Cl_2 (9 mL) and a stir bar were charged to a 2 dram vial. The vial was then stirred for 5 minutes. PVC (136.3 mg, 2.176 mmol, 1 equiv.) and HDPE (3.837 mg, 136.8 mmol, 62.9 equiv.) was weighed into a 30 mL Chemglass heavy wall pressure vessel equipped with a Teflon-coated cap. The CH_2Cl_2 mixture was then added to the PVC, and the vial was sealed with Teflon tape along the inside of the cap, and electrical tape on the outside. The vial was then placed inside an oil bath that was preheated to 110 °C. After 48 hours, the reaction mixture was quenched with methanol (~ 15 mL), which resulted in the precipitation of white solids. The DCM fraction was pipetted away from the insoluble HDPE beads, and the HDPE beads were washed with 5 mL methanol, and the methanol wash was added to the DCM fraction. Both the HDPE and DCM fraction were filtered separately and the solids were washed with 15 mL of methanol. The polymer was then dried at 60 °C for 18 hours.

2.8 Synthesis of poly(ethylene) from low molecular weight PVC under dilute conditions: ($[Ph_3C][B(C_6F_5)_4]$) (16.9 mg, 0.018 mmol, 0.8 mol%), Et_3SiH (2.1 mL, 13.2 mmol, 6 equiv.) CH_2Cl_2 (9 mL) and a stir bar were charged to a 2 dram vial. The vial was then stirred for 5 minutes. PVC (136.3 mg, 2.176 mmol, 1 equiv.) was weighed into a 30 mL Chemglass heavy wall pressure vessel. The CH_2Cl_2 mixture was then added to the PVC before the pressure vessel was placed inside an oil bath that was preheated to 110 °C. After 48 hours, the reaction mixture was quenched with methanol (~ 15 mL), which resulted in the precipitation of white solids. The mixture was then filtered and washed with methanol (~5 mL) three times. The polymer was then dried at 60 °C for 18 hours.

2.9 Extraction of PVC from PVC items: A PVC item (either a vinyl record or toy PVC lizard) was dissolved in a minimal amount of tetrahydrofuran, in a 20 mL scintillation vial and heated at 50 °C until dissolved. 15 mL of methanol was added to crash out a rubbery solid. The solid was then filtered and washed with methanol (~15 mL) and then placed back into the 20 mL scintillation vial. The dissolving and washing process was repeated a total of two more times. The resulting solid

material was then dried in a vacuum oven under reduced pressure at 60 °C for 36 hours.

2.10 Synthesis of partially chlorinated poly(ethylene) from low molecular weight PVC (Table S1, Entry 1): ($[Ph_3C][B(C_6F_5)_4]$) (10.4 mg, 0.011 mmol, 0.5 mol%), Et_3SiH (0.26 mL, 2.38 mmol, 0.75 equiv.) CH_2Cl_2 (3 mL) and a stir bar were charged to a 2 dram vial. The vial was then stirred for 5 minutes. PVC (136 mg, 1.632 mmol, 1 equiv.) was weighed into a 2 dram vial equipped with a Teflon-coated cap. The CH_2Cl_2 mixture was then added to the PVC, and the vial was sealed with Teflon tape along the inside of the cap, and electrical tape on the outside. The vial was then placed inside a Chemglass high-throughput tray that was preheated to 60 °C. After 18 hours, the reaction mixture was quenched with methanol (~ 15 mL), which resulted in the precipitation of white solids. The mixture was then filtered and washed with methanol (~5 mL) and $CHCl_3$ (~1 mL) three times. The polymer was then dried at 60 °C for 18 hours.

2.11 Synthesis of poly(ethylene) from low molecular weight PVC at 25 °C (Table S4, Entry 1-3): ($[Ph_3C][B(C_6F_5)_4]$) (16.8 mg, 0.018 mmol, 0.8 mol%), Et_3SiH (0.42 mL, 2.63 mmol, 1.2 equiv.) CH_2Cl_2 (3 mL) and a stir bar were charged to a 2 dram vial. The vial was then stirred for 5 minutes. PVC (136 mg, 2.176 mmol, 1 equiv.) was weighed into a 2 dram vial equipped with a Teflon-coated cap. The CH_2Cl_2 mixture was then added to the PVC, and the vial was sealed with Teflon tape along the inside of the cap, and electrical tape on the outside. The vial was then placed inside a Chemglass high-throughput tray that was preheated to 60 °C. After 5 minutes, the vial was stirred at room temperature for 18 hours. The reaction mixture was quenched with methanol (~ 15 mL), which resulted in the precipitation of white solids. The mixture was then filtered and washed with methanol (~5 mL) and $CHCl_3$ (~1 mL) three times. The polymer was then dried at 60 °C for 6 hours.

2.12 Synthesis of poly(ethylene) from low molecular weight PVC at 25 °C (Table S4, Entry 4-5): ($[Ph_3C][B(C_6F_5)_4]$) (16.8 mg, 0.018 mmol, 0.8 mol%), Et_3SiH (0.42 mL, 2.63 mmol, 1.2 equiv.) CH_2Cl_2 (9 mL) and a stir bar were charged to a 2 dram vial. The vial was then stirred for 5 minutes. PVC (136 mg, 2.176 mmol, 1 equiv.) was weighed into a 2 dram vial equipped with a Teflon-coated cap. The CH_2Cl_2 mixture was then added to the PVC, and the vial was sealed with Teflon tape along the inside of the cap, and electrical tape on the outside. The vial was then placed inside a Chemglass high-throughput tray that was preheated to 60 °C. After 5 minutes, the vial was stirred at room temperature for 72 hours. The reaction mixture was quenched with methanol (~ 15 mL), which resulted in the precipitation of white solids. The mixture was then filtered and washed with methanol (~5 mL) and $CHCl_3$ (~1 mL) three times. The polymer was then dried at 60 °C for 18 hours.

2.13 Synthesis of poly(ethylene) from low molecular weight PVC at 110 °C (For ^{13}C analysis): ($[Ph_3C][B(C_6F_5)_4]$) (33.6 mg, 0.036 mmol, 0.8 mol%), Et_3SiH (4.2 mL, 26.4 mmol, 6 equiv.) CH_2Cl_2 (9 mL) and a stir bar were charged to a 2

dram vial. The vial was then stirred for 5 minutes. PVC (272.1 mg, 4.352 mmol, 1 equiv.) was weighed into a 30 mL Chemglass heavy wall pressure vessel. The CH_2Cl_2 mixture was then added to the PVC before the pressure vessel was placed inside an oil bath that was preheated to 110 °C. After 48 hours, the reaction mixture was quenched with methanol (~ 15 mL), which resulted in the precipitation of white solids. The mixture was then filtered and washed with methanol (~5 mL) three times. The polymer was then dried at 60 °C for 18 hours.

2.14 Safety Note: Reactions were done well above the boiling point of CH_2Cl_2 either in a Chemglass pressure vessel that is rated for elevated pressure or in a 2 dram vial with a Teflon-coated cap. In the case of the vial, a Chemglas high throughput heating block was used with a lid locked over the top of the vials to ensure safety when these vials reach the temperature.

Table S1 Dechlorination of low molar mass PVC with 0.5 mol% $[CPh_3][B(C_6F_5)_4]$ loading.

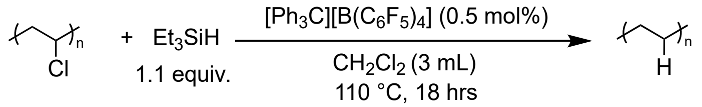

Entry	% Cl loss (^{1}H NMR)[a]	B/1000C[b]	T_m (°C)	$T_{d,5\%}$(°C)
1	>99	51	59	446

Table S2 Partial dechlorination of PVC with substoichiometric Et_3SiH.

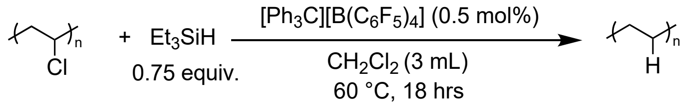

Entry	% Cl loss (^{1}H NMR)[a]	B/1000C[b]	T_m (°C)	$T_{d,5\%}$(°C)
1	82	-	-	-

Table S3 Additional triplicate runs of PVC to branched PE.

Entry	Time (h)	Init. Mol%	Et_3SiH equiv.	% Cl loss (^1H NMR)[a]	B/1000C[b]	T_m (°C)	$T_{d,5\%}$(°C)	Isolated yield (%)
1	2	0.8	1.2	>99	46	51	451	95
2	2	0.8	3.6	>99	43	76	451	37
3	2	0.8	6	>99	31	84	440	69
4	2	1.6	6	>99	40	73	450	79
5a<br>5b	48	0.8	6	>99	33<br>35	85<br>85	451<br>-	47<br>86

Table S4 Conversion of PVC to PE at room temperature (25 °C).

Entry	Temperature (Celsius)	Time (hours)	CH_2Cl_2 (mL)	% Cl loss (^1H NMR)[a]	B/1000C[b]	T_m (°C)	$T_{d,5\%}$(°C)	Isolated yield (%)
1a<br>1b<br>1c	25	18	3	>99<br>92<br>92	50<br>-<br>-	49<br>46<br>44	445<br>362<br>316	91<br>-<br>150
2a<br>2b	25	72	9	87<br>87	-	-	-	86<br>92

Table S5 Screening for the conversion of PVC to PE in different solvents.

Entry	Temperature (Celsius)	Time (hours)	Solvent (mL)	Conversion by IR? Y/N
1	110	18	CH_2Cl_2 (3)	Y
2	110	18	Hexanes (9)	N
3	110	1	Tetrahydrofuran (3)	N
4	110	2	1,2,4-trichlorobenzene (9)	N
5	110	2	Dimethylacetamide (3)	N
6	110	2	Cyclopentanemethylether (3)	N
7	110	18	Methylcyclohexane (6)	N

Table S6 Summary of Elemental Analysis Results.

Item	%C	%H	%N
Table S3, Entry 1	84.927	13.372	0
HDPE (Sigma)	85.266	13.908	0
Theoretical	85.630	14.370	0

Results and Discussion

1. FT-IR Spectroscopy

Note: noise present around 1950 cm^{-1} is instrument noise and is present in all samples.

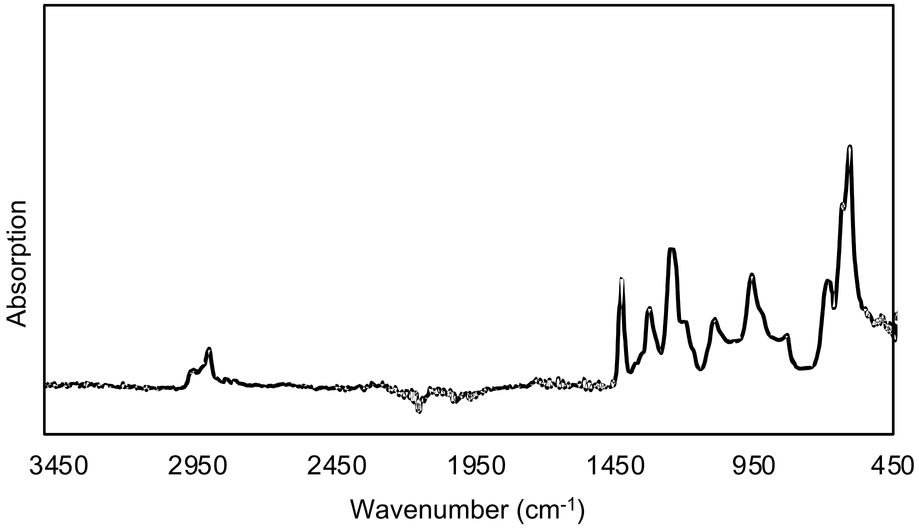

Fig. S1 FT-IR spectrum of low molecular weight PVC (CAS: 9002-86-2, Product #: 81388, Sigma Aldrich).

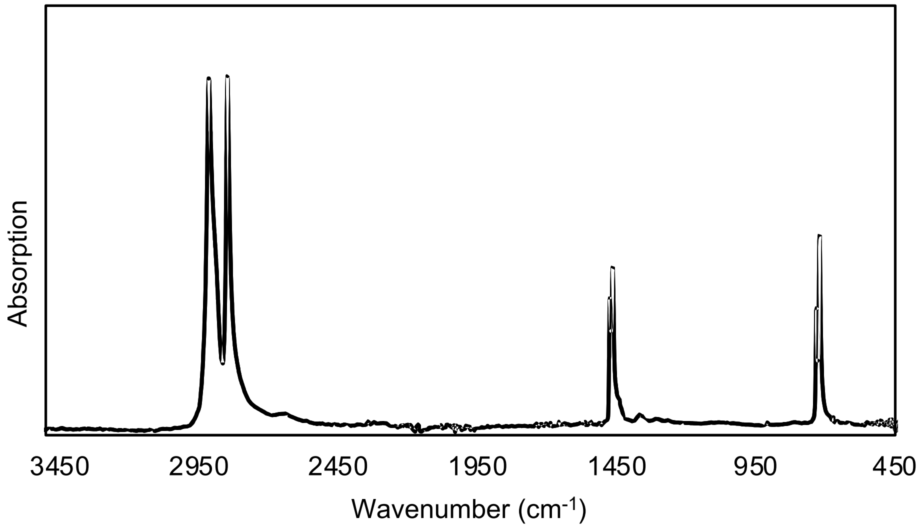

Fig. S2 FT-IR spectrum of HDPE pellets (CAS: 9002-88-4, Sigma Aldrich).

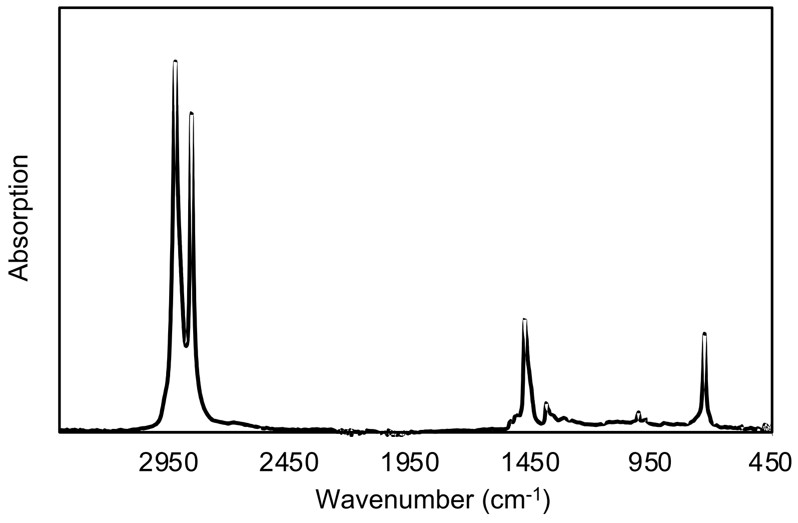

Fig. S3 FT-IR spectrum of Table 1, Entry 1a.

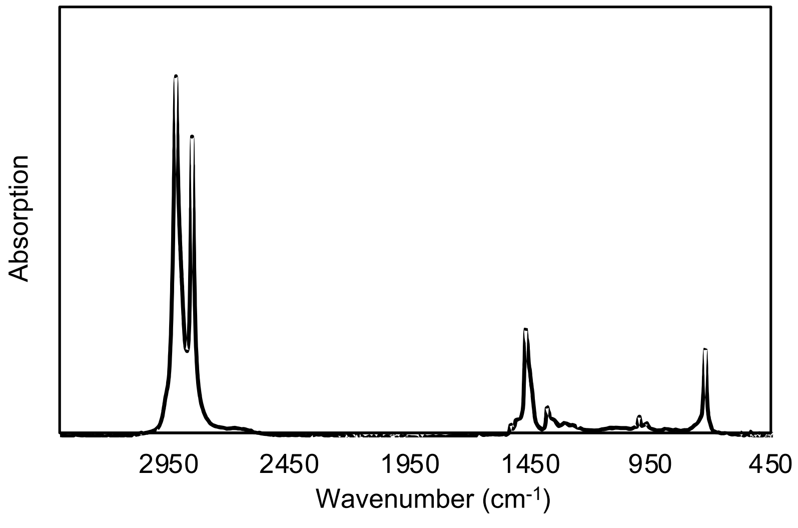

Fig. S4 FT-IR spectrum of Table 1, Entry 1b.

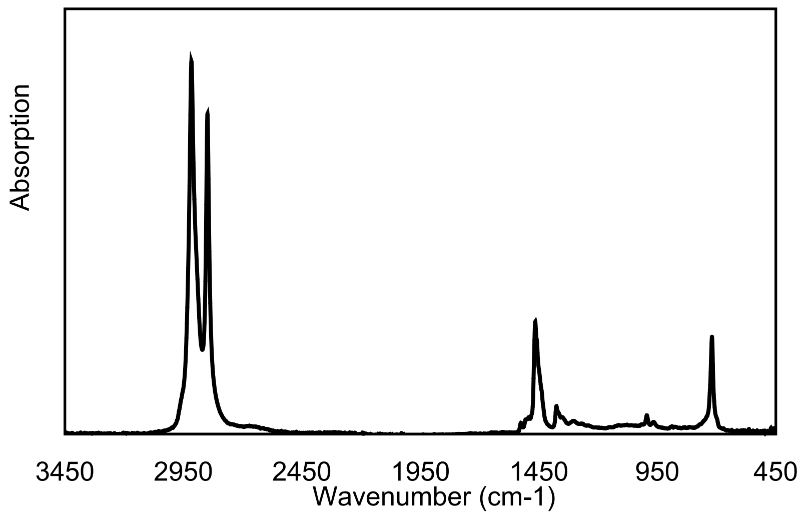

Fig. S5 FT-IR spectrum of Table 1, Entry 2a.

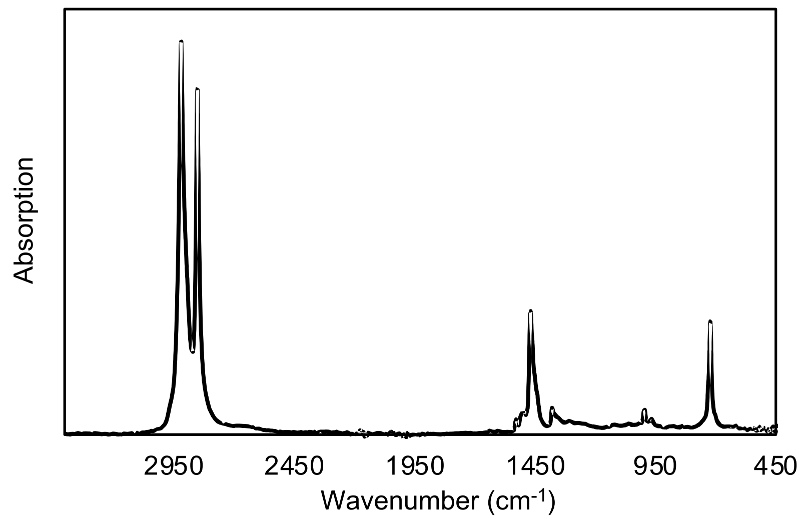

Fig. S6 FT-IR spectrum of Table 1, Entry 2b.

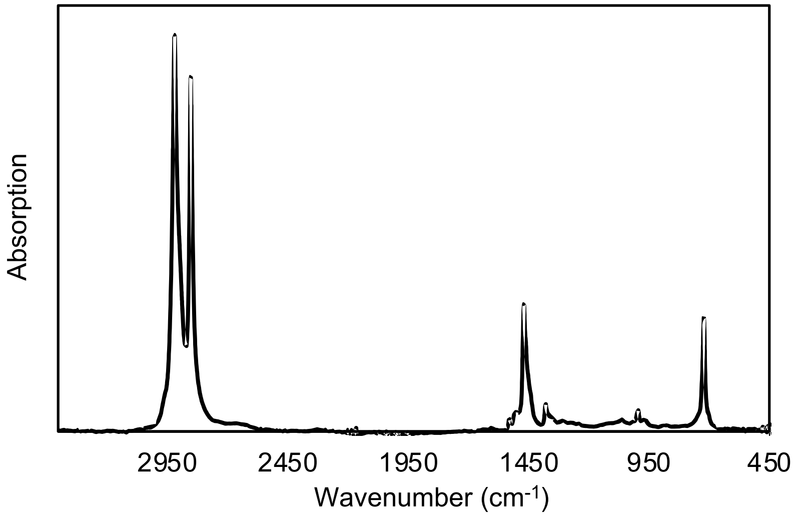

Fig. S7 FT-IR spectrum of Table 1, Entry 3a.

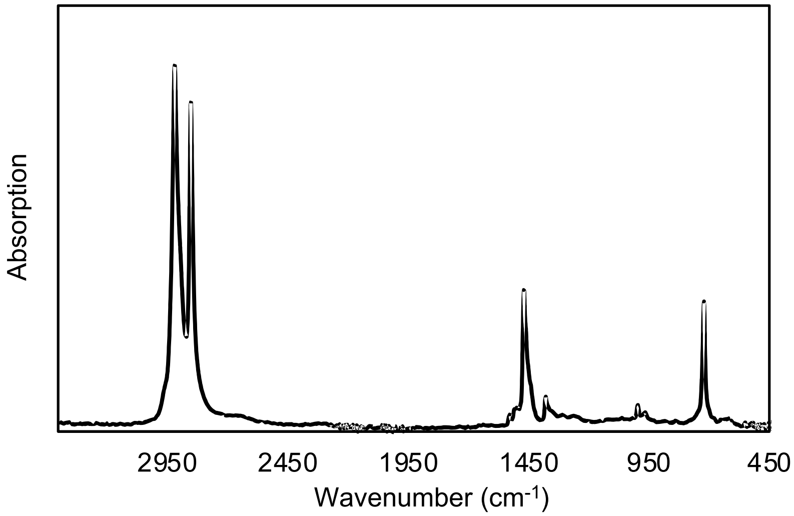

Fig. S8 FT-IR spectrum of Table 1, Entry 3b.

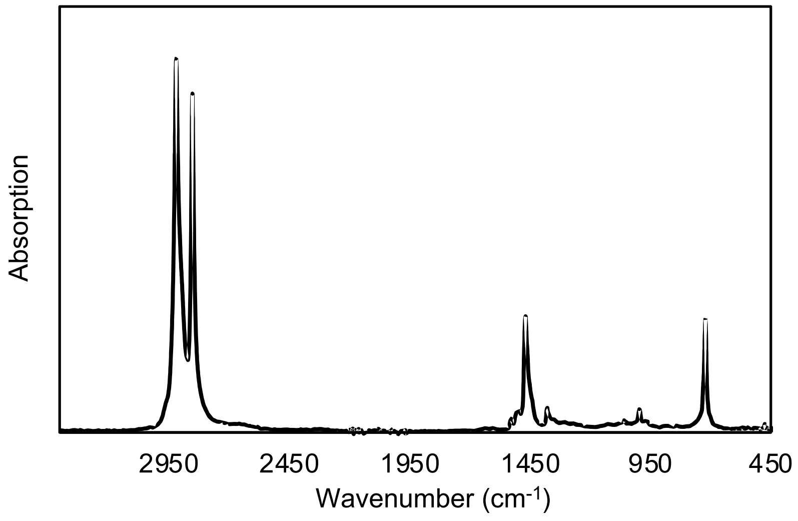

Fig. S9 FT-IR spectrum of Table 1, Entry 4a.

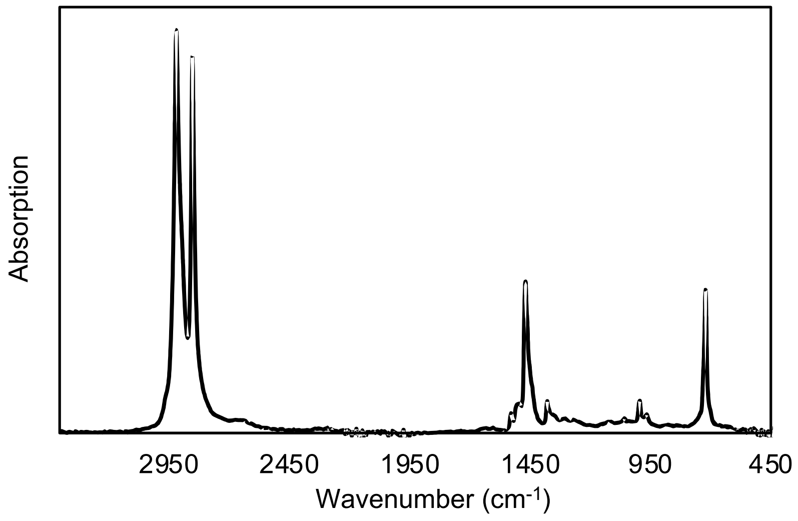

Fig. S10 FT-IR spectrum of Table 1, Entry 4b.

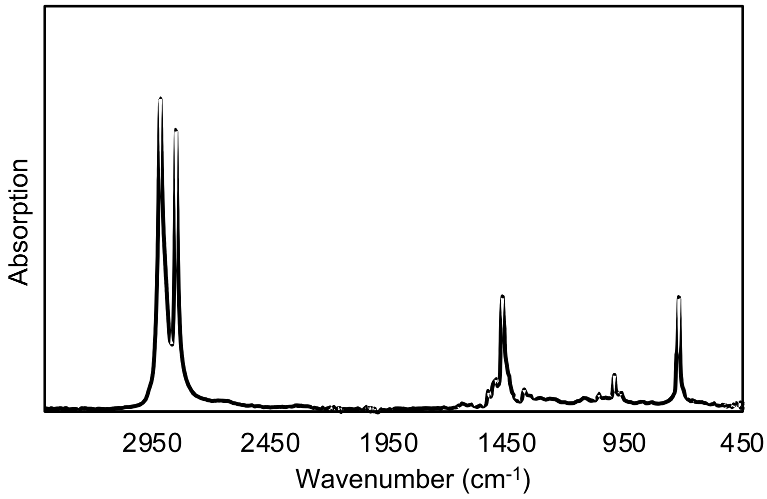

Fig. S11 FT-IR spectrum of Table 1, Entry 5a.

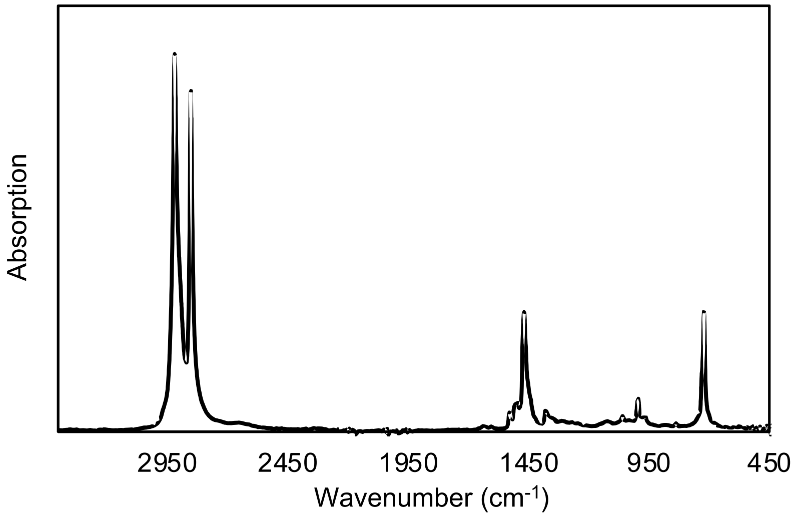

Fig. S12 FT-IR spectrum of Table 1, Entry 5b.

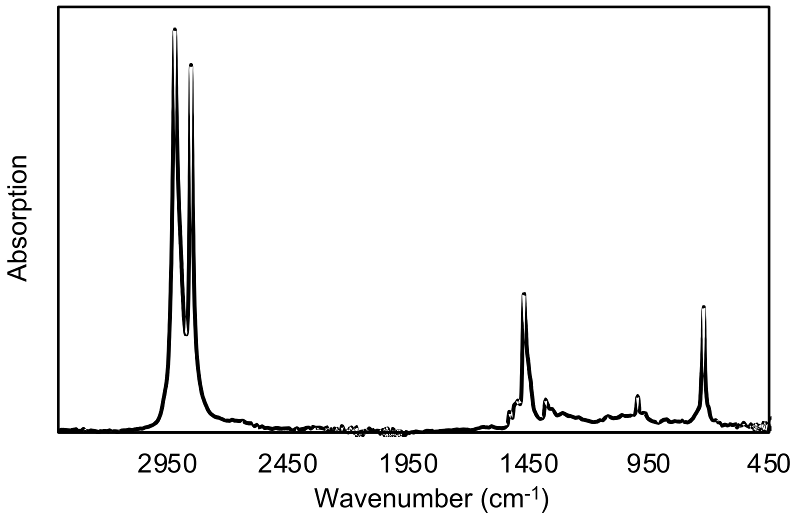

Fig. S13 FT-IR spectrum of Table 1, Entry 6a.

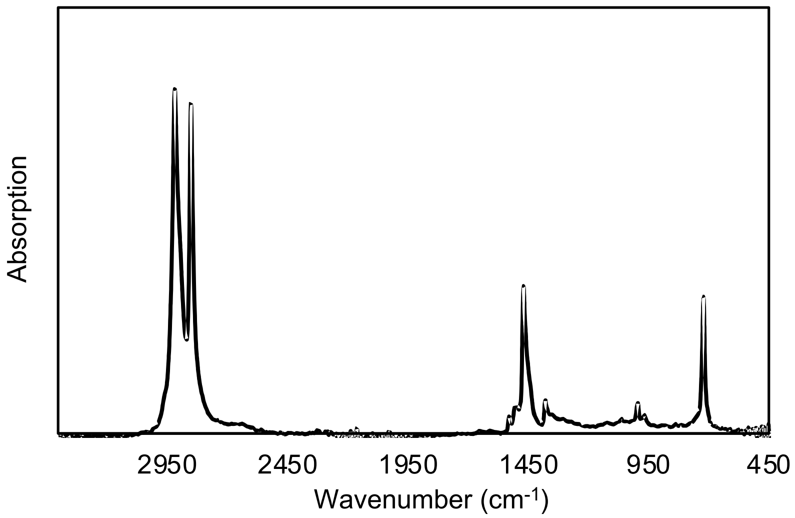

Fig. S14 FT-IR spectrum of Table 1, Entry 6b.

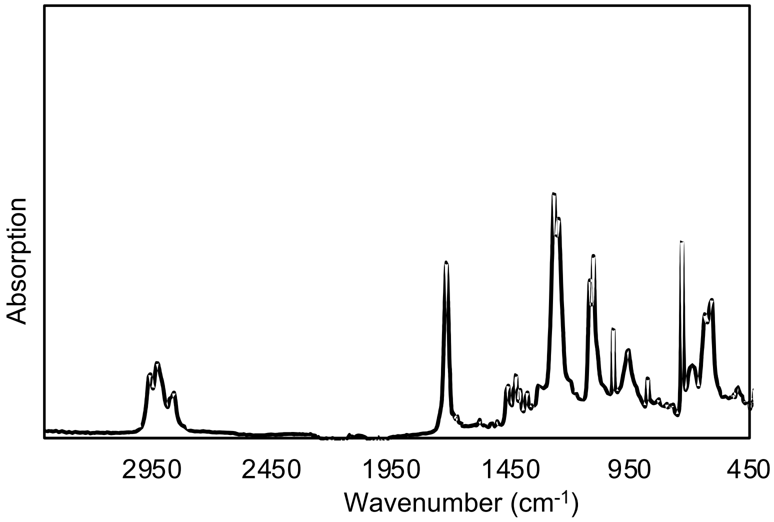

Fig. S15 FT-IR spectrum of PVC toy lizard item.

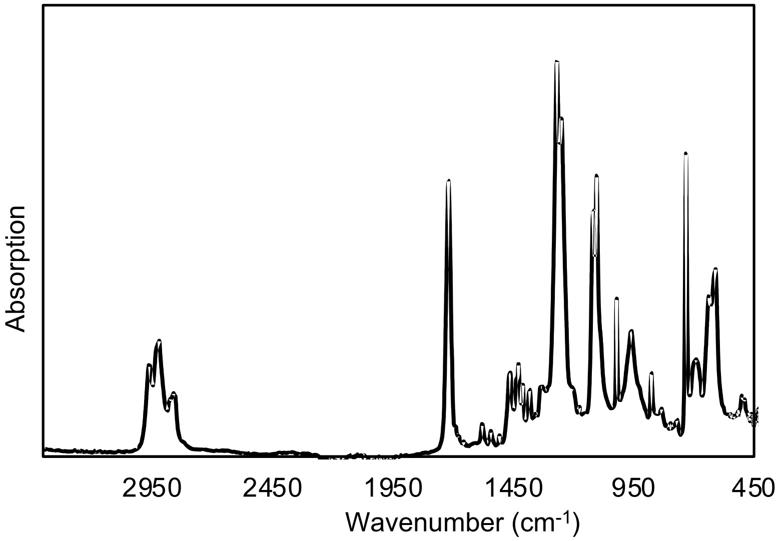

Fig. S16 FT-IR spectrum of soft PVC pipe item.

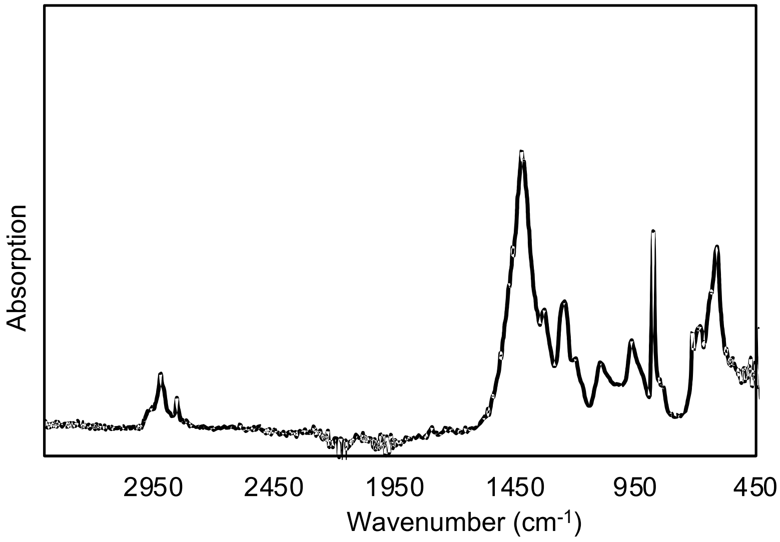

Fig. S17 FT-IR spectrum of rigid PVC pipe item.

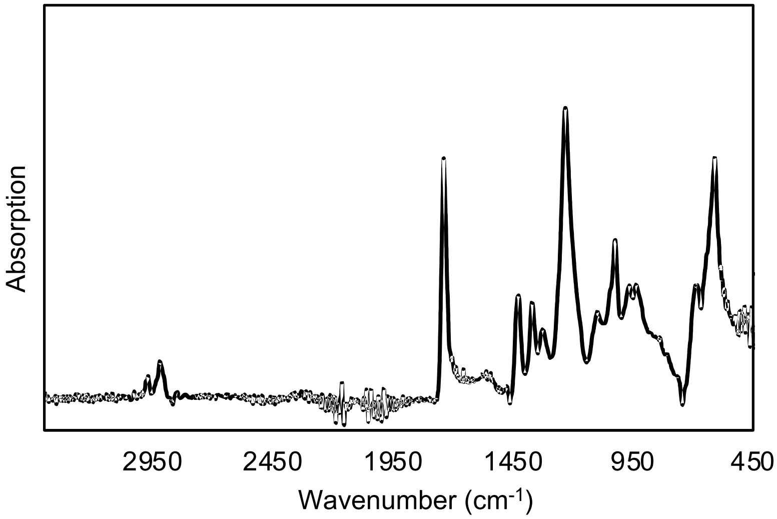

Fig. S18 FT-IR spectrum of vinyl record item.

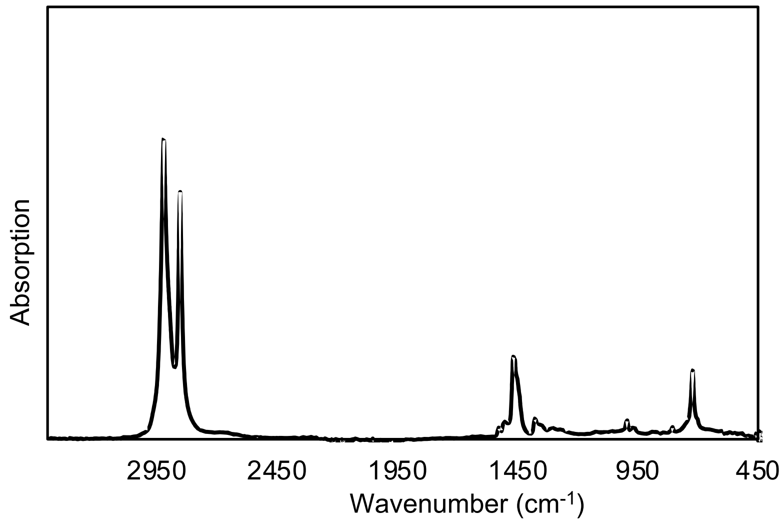

Fig. S19 FT-IR spectrum of Table 2, Entry 1 (PVC toy lizard item).

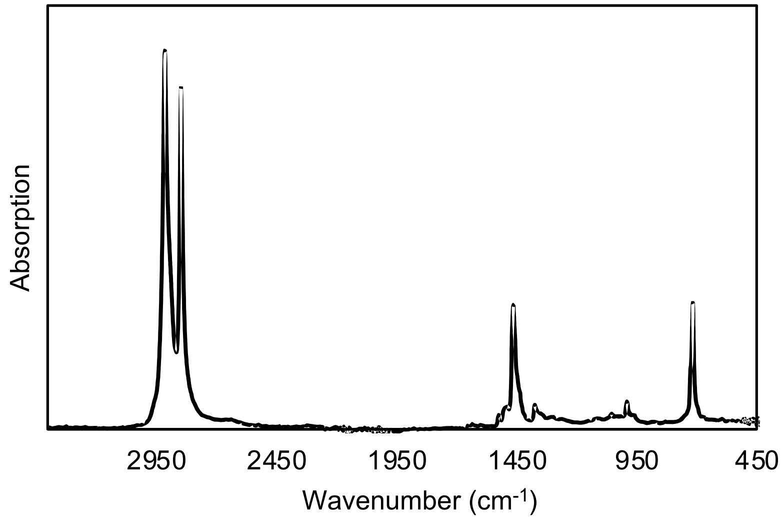

Fig. S20 FT-IR spectrum of Table 2, Entry 2 (extracted PVC toy lizard).

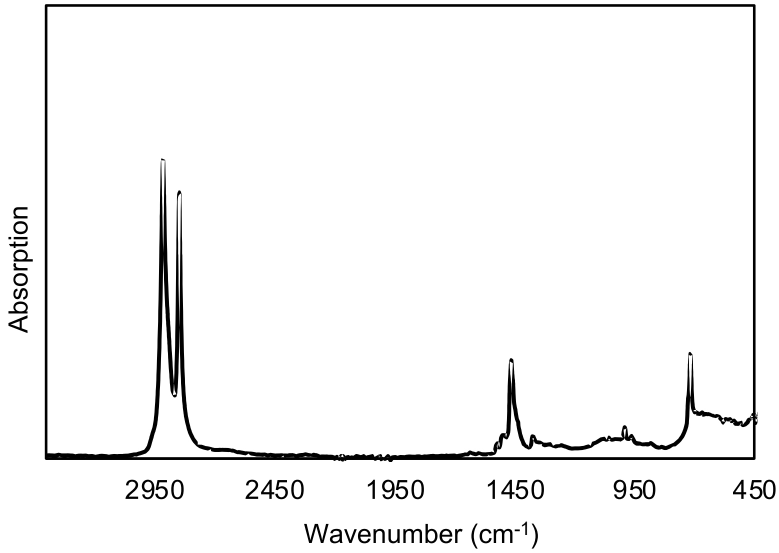

Fig. S21 FT-IR spectrum of Table 2, Entry 3 (rigid PVC pipe item).

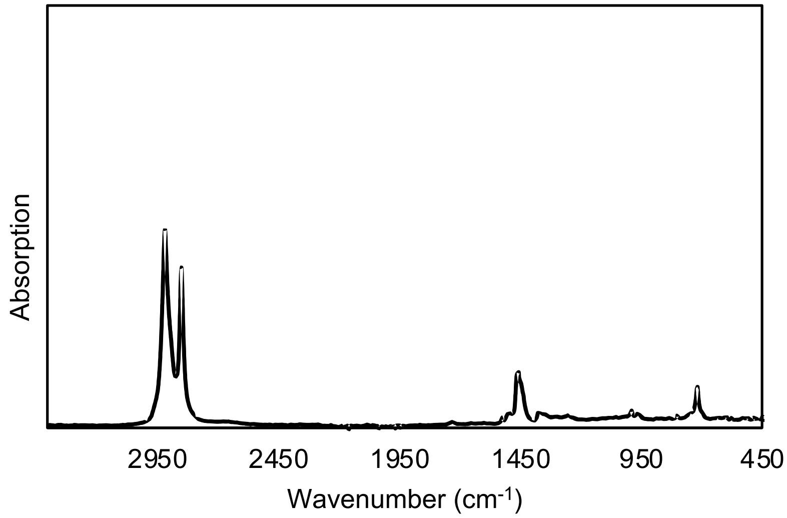

Fig. S22 FT-IR spectrum of Table 2, Entry 4 (soft PVC pipe item).

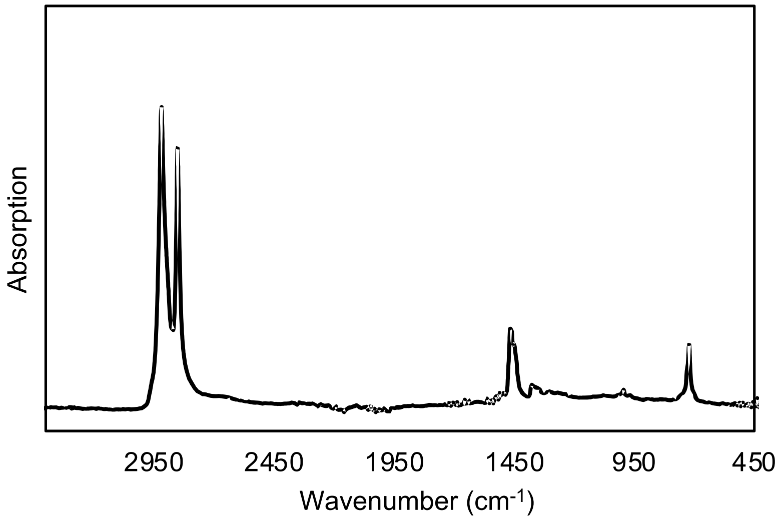

Fig. S23 FT-IR spectrum of Table 2, Entry 5 (vinyl record item).

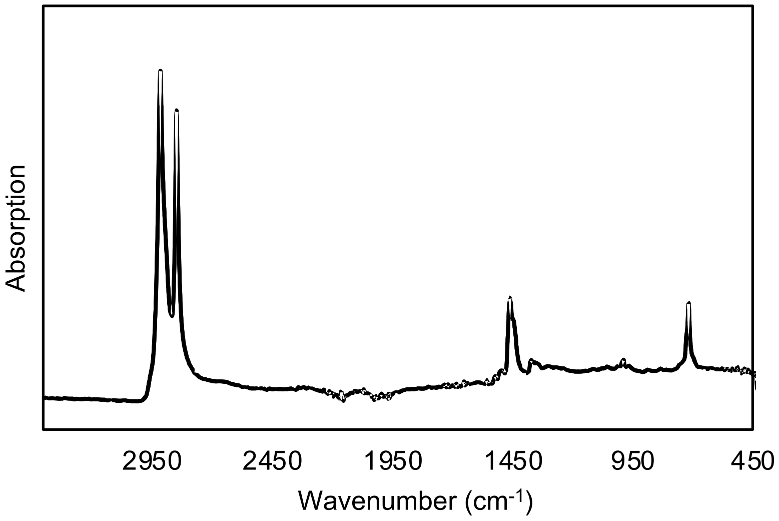

Fig. S24 FT-IR spectrum of Table 2, Entry 6 (extracted vinyl record item).

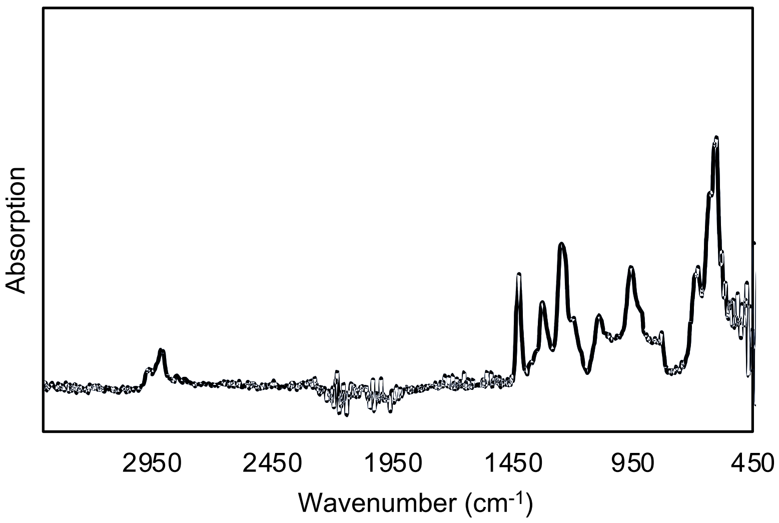

Fig. S25 FT-IR spectrum of extracted PVC toy lizard item.

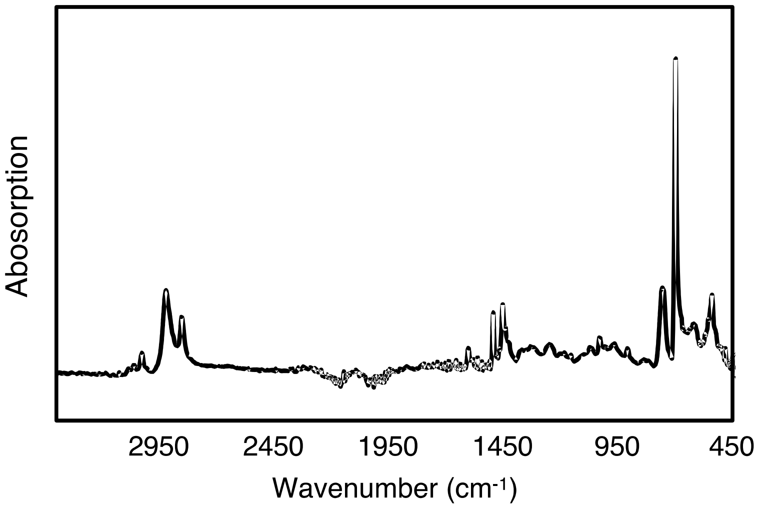

Fig. S26 FT-IR spectrum of product from PS-PVC mixture.

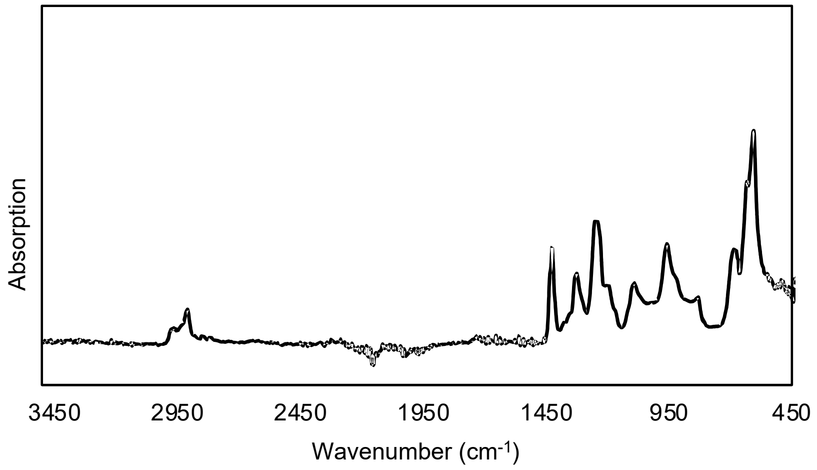

Fig. S27 FT-IR spectrum of product from PET-PVC mixture.

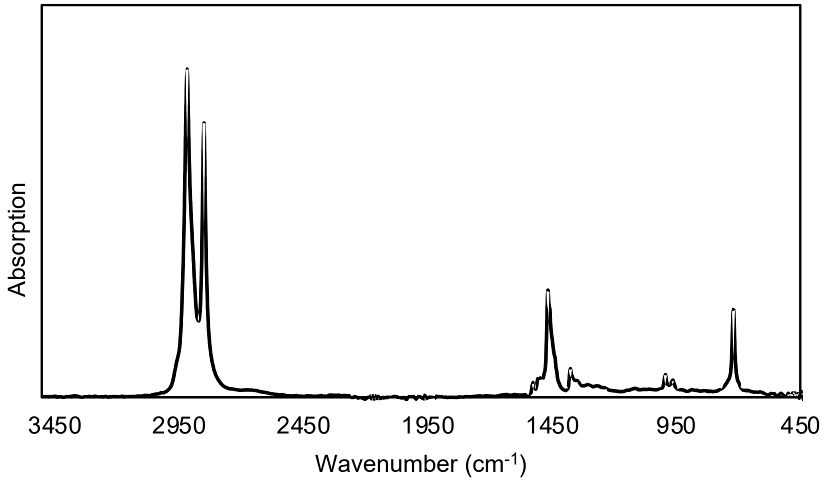

Fig. S28 FT-IR spectrum of Table S1, Entry 1.

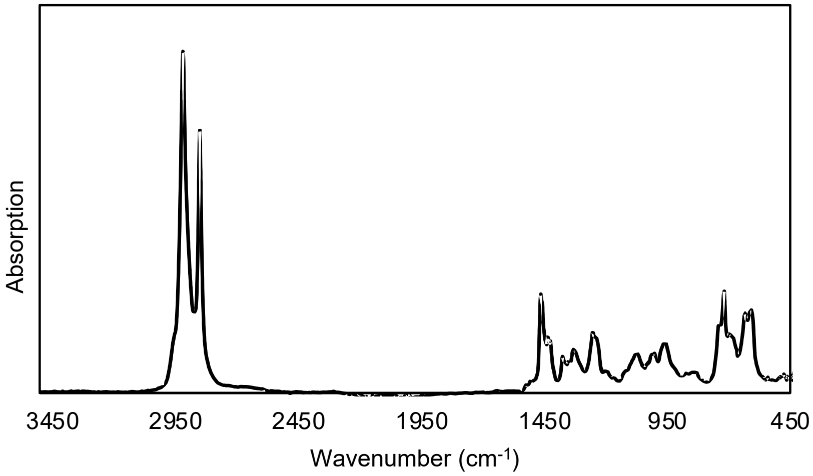

Fig. S29 FT-IR spectrum of Table S2, Entry 1.

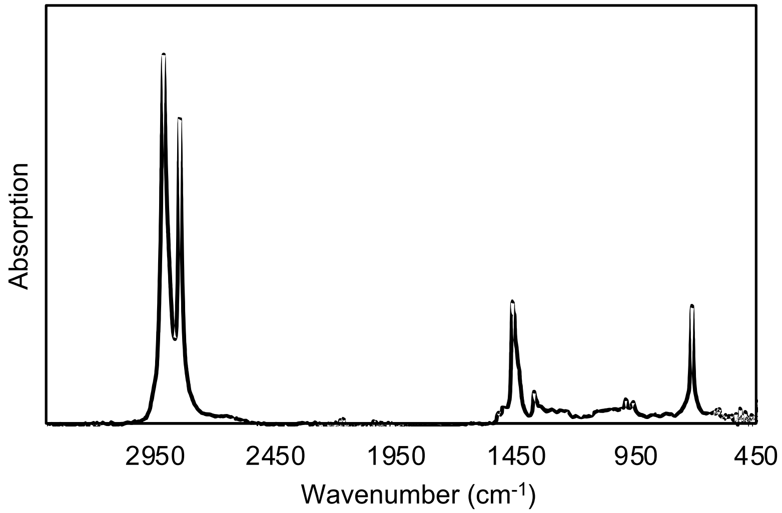

Fig. S30 FT-IR spectrum of Table S3, Entry 1.

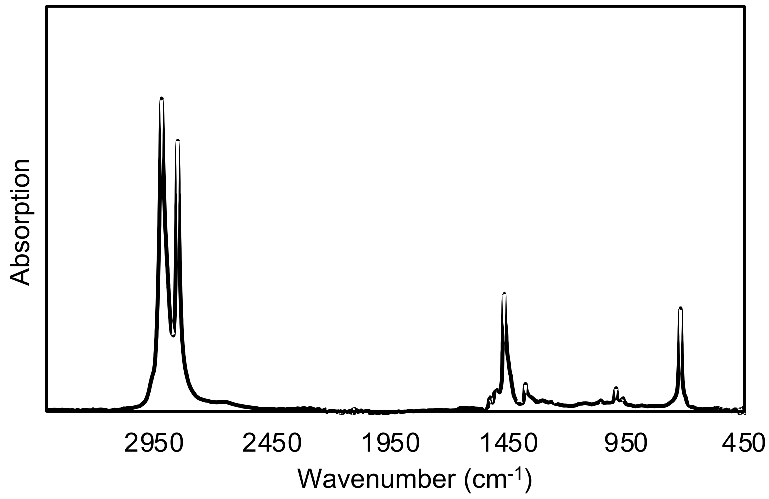

Fig. S31 FT-IR spectrum of Table S3, Entry 2.

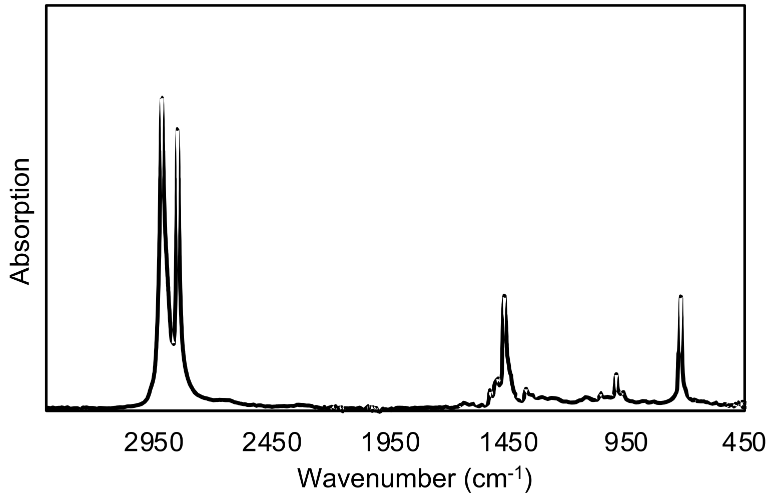

Fig. S32 FT-IR spectrum of Table S3, Entry 3.

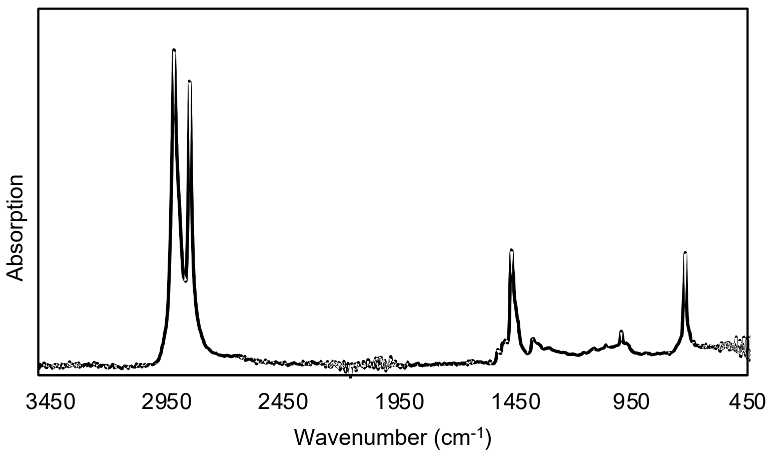

Fig. S33 FT-IR spectrum of Table S3, Entry 4.

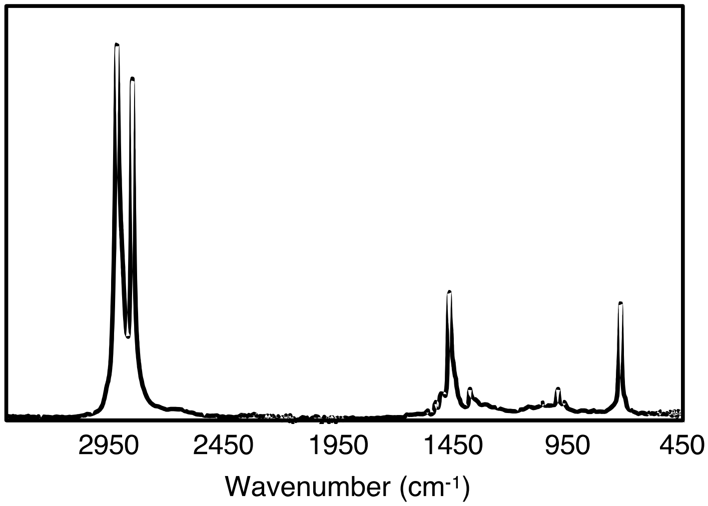

Fig. S34 FT-IR spectrum of Table S3, Entry 5a.

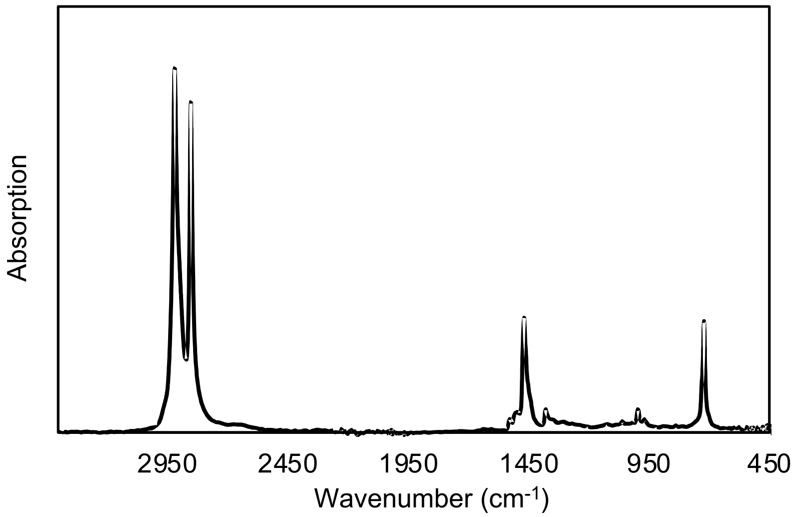

Fig. S35 FT-IR spectrum of Table S3, Entry 5b.

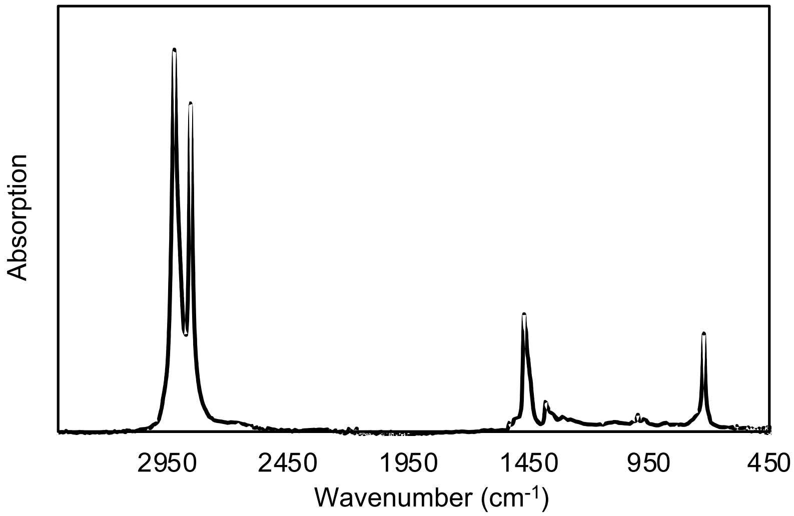

Fig. S36 FT-IR spectrum of Table S4, Entry 1a.

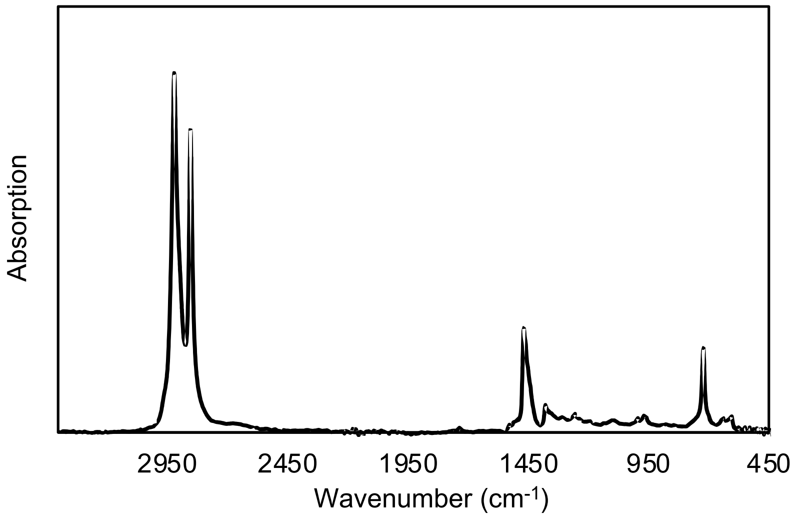

Fig. S37 FT-IR spectrum of Table S4, Entry 1b.

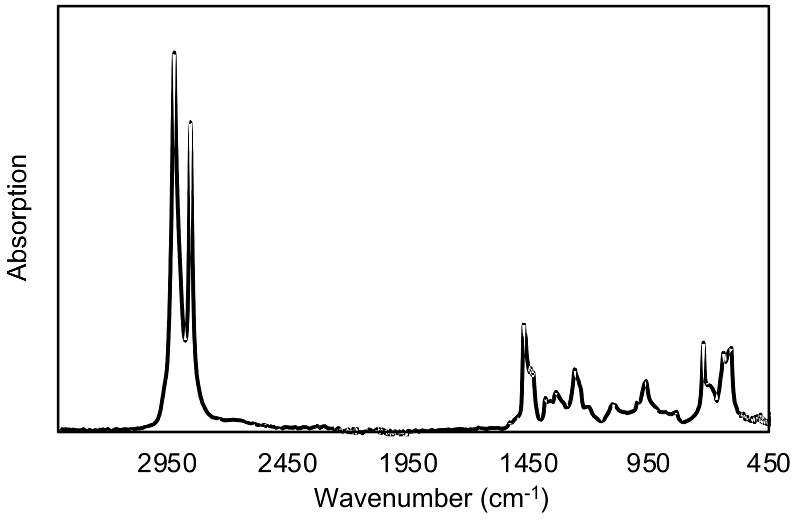

Fig. S38 FT-IR spectrum of Table S4, Entry 1c.

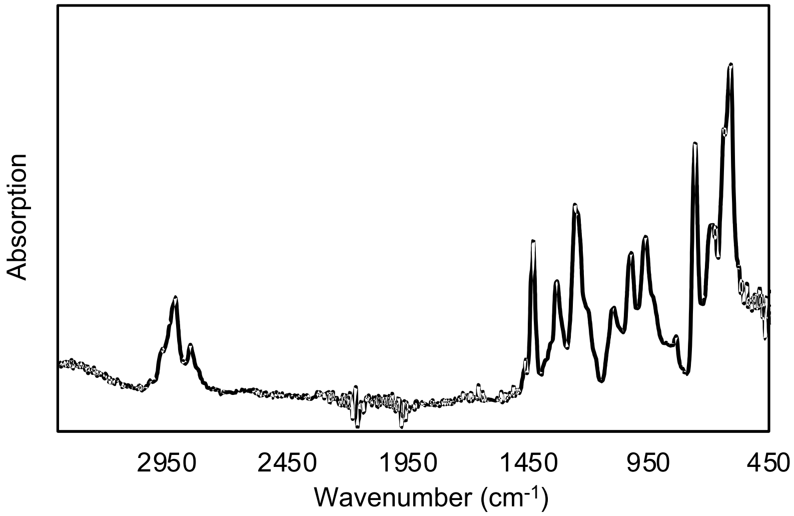

Fig. S39 Representative FT-IR spectrum of Table S5, Entries 2-7.

2. ¹H NMR Spectroscopy

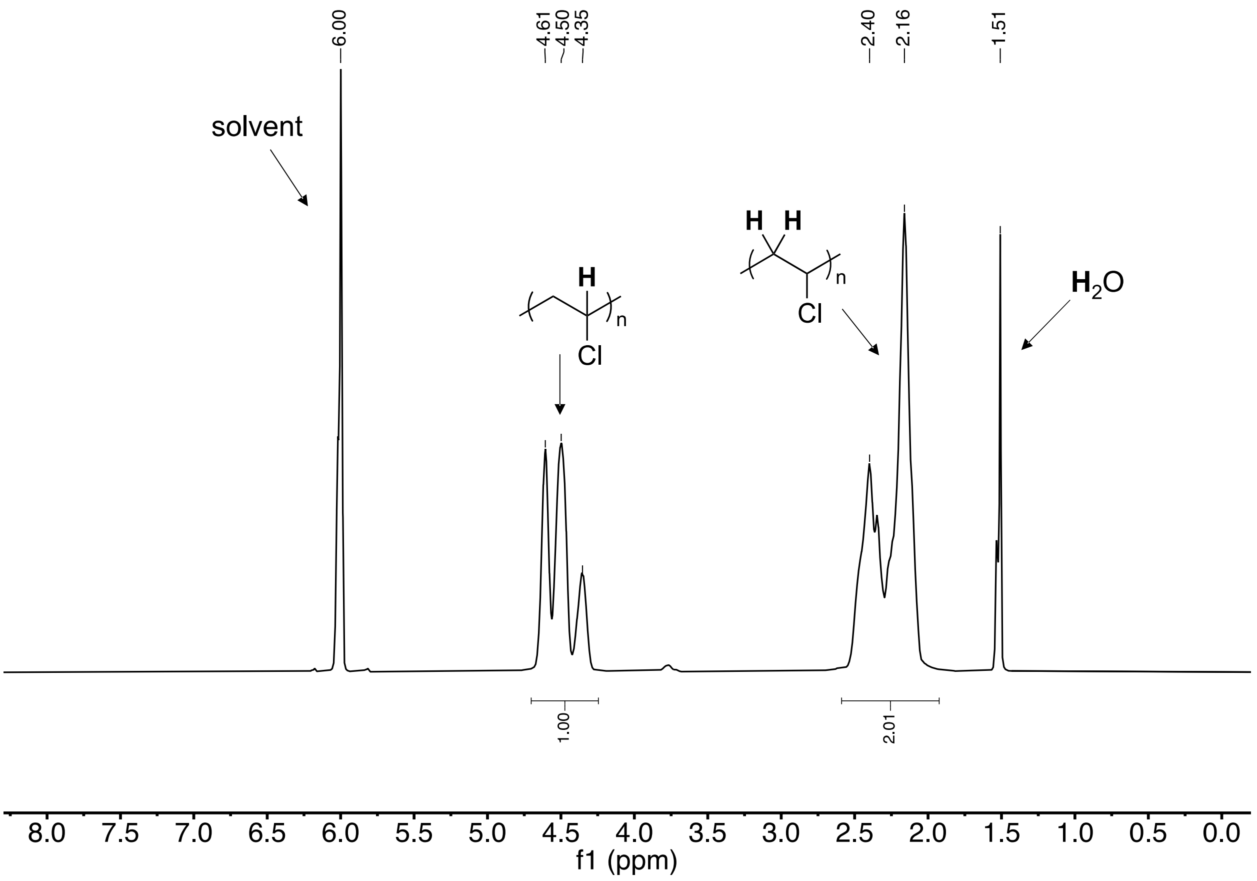

Fig. S40 ¹H NMR spectrum, in tetrachloroethane-d_2 (80 °C), of low molecular weight PVC (CAS: 9002-86-2, Product #: 81388, Sigma Aldrich).

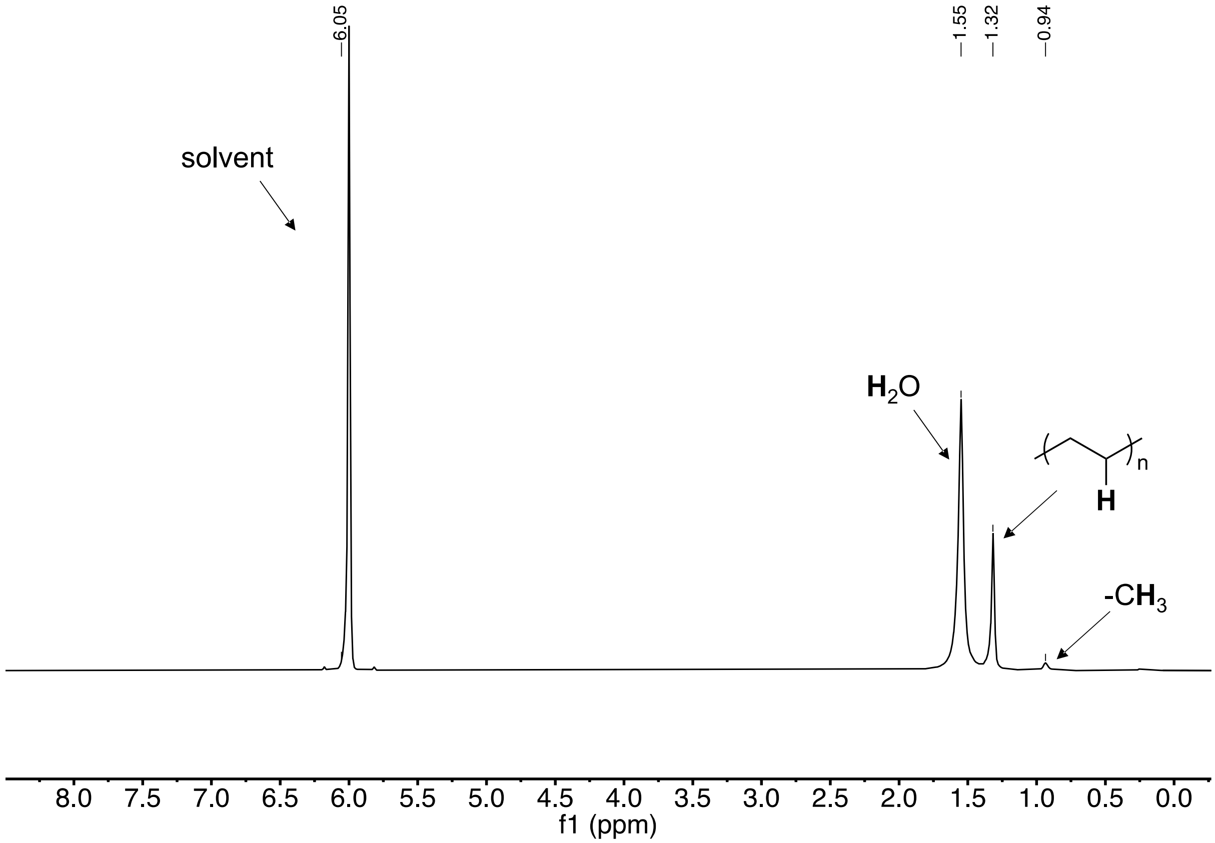

Fig. S41 ^{1}H NMR spectrum, in tetrachloroethane-d$_2$ (80 °C), of HDPE pellets (CAS: 9002-88-4, Sigma Aldrich).

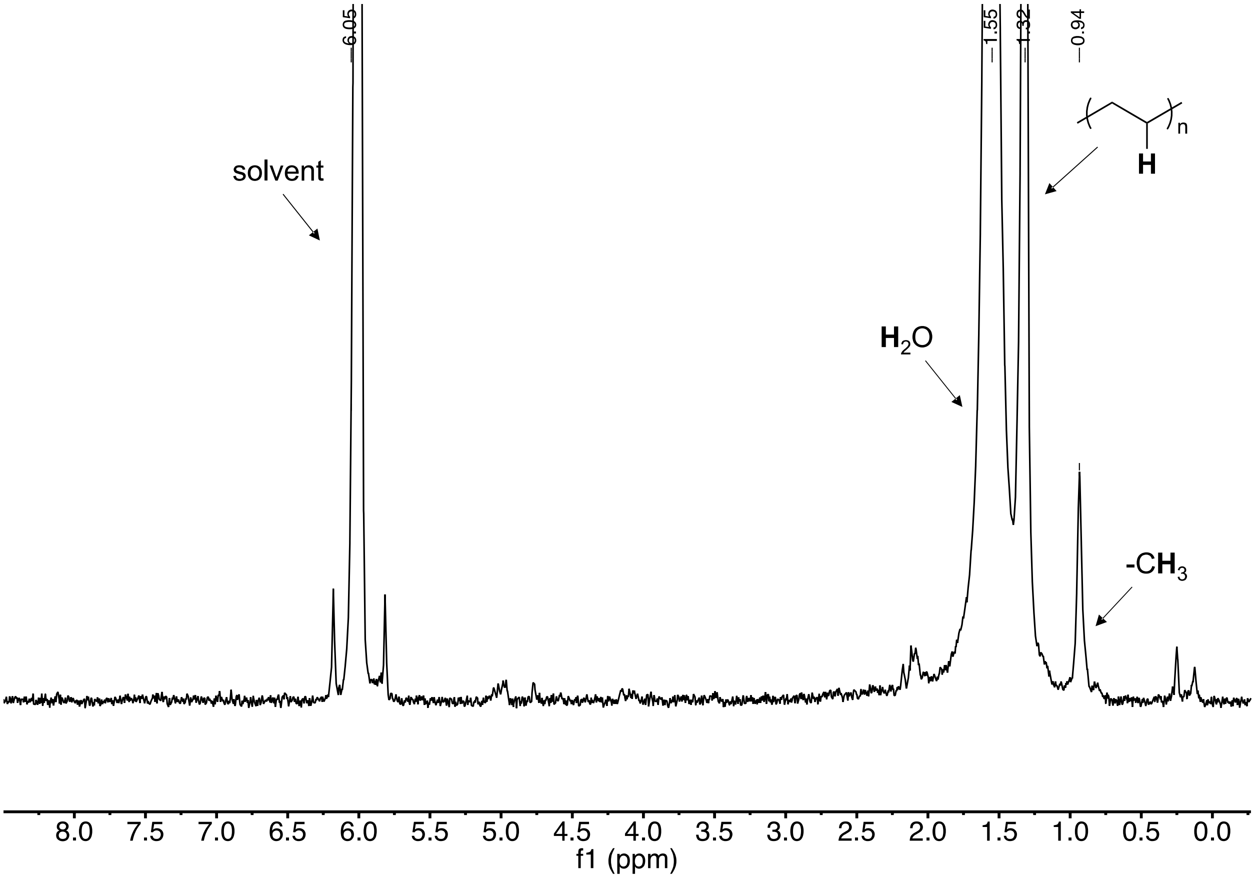

Fig. S41a Zoomed in ^{1}H NMR spectrum, in tetrachloroethane-d_2 (80 °C), of HDPE pellets (CAS: 9002-88-4, Sigma Aldrich).

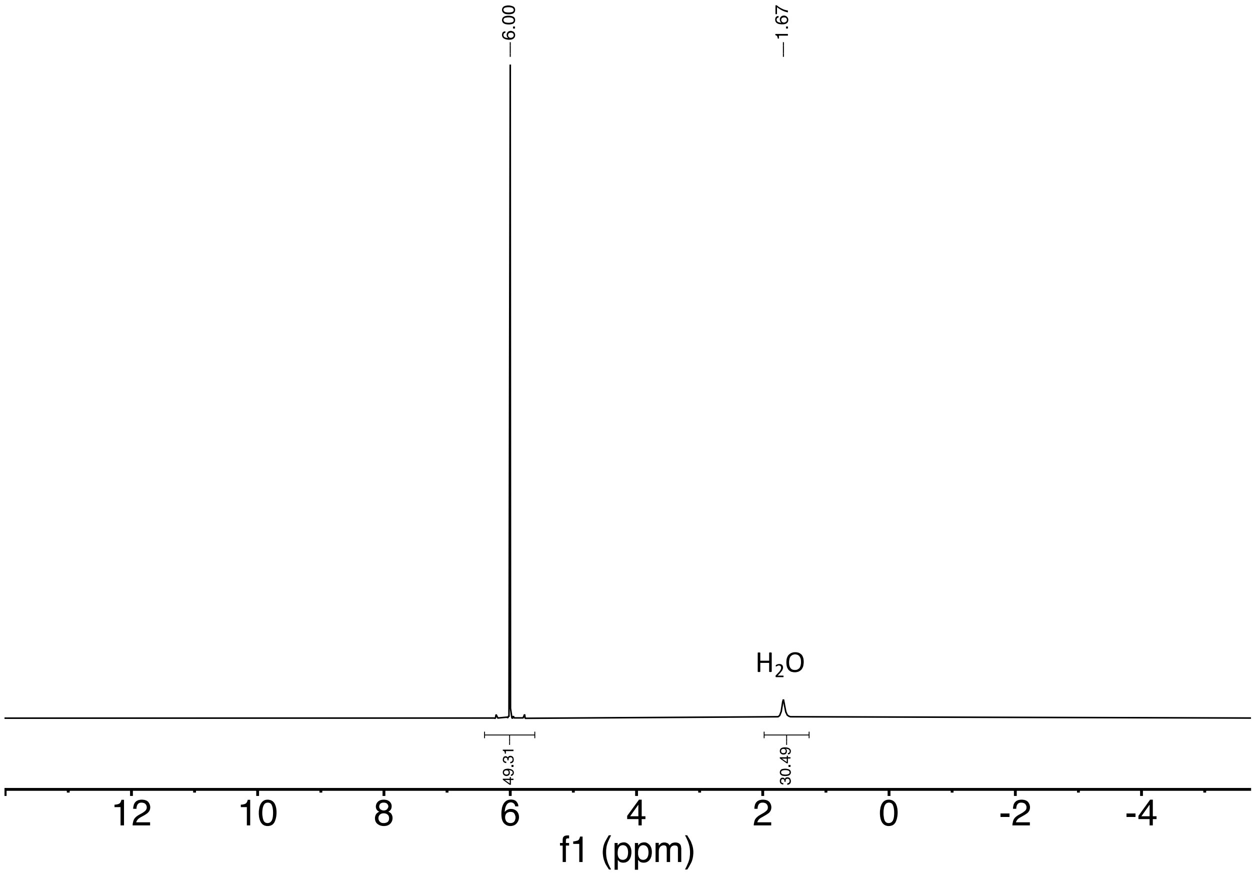

Fig. S42 ^{1}H NMR spectrum of tetrachloroethane-d_2 (25 °C).

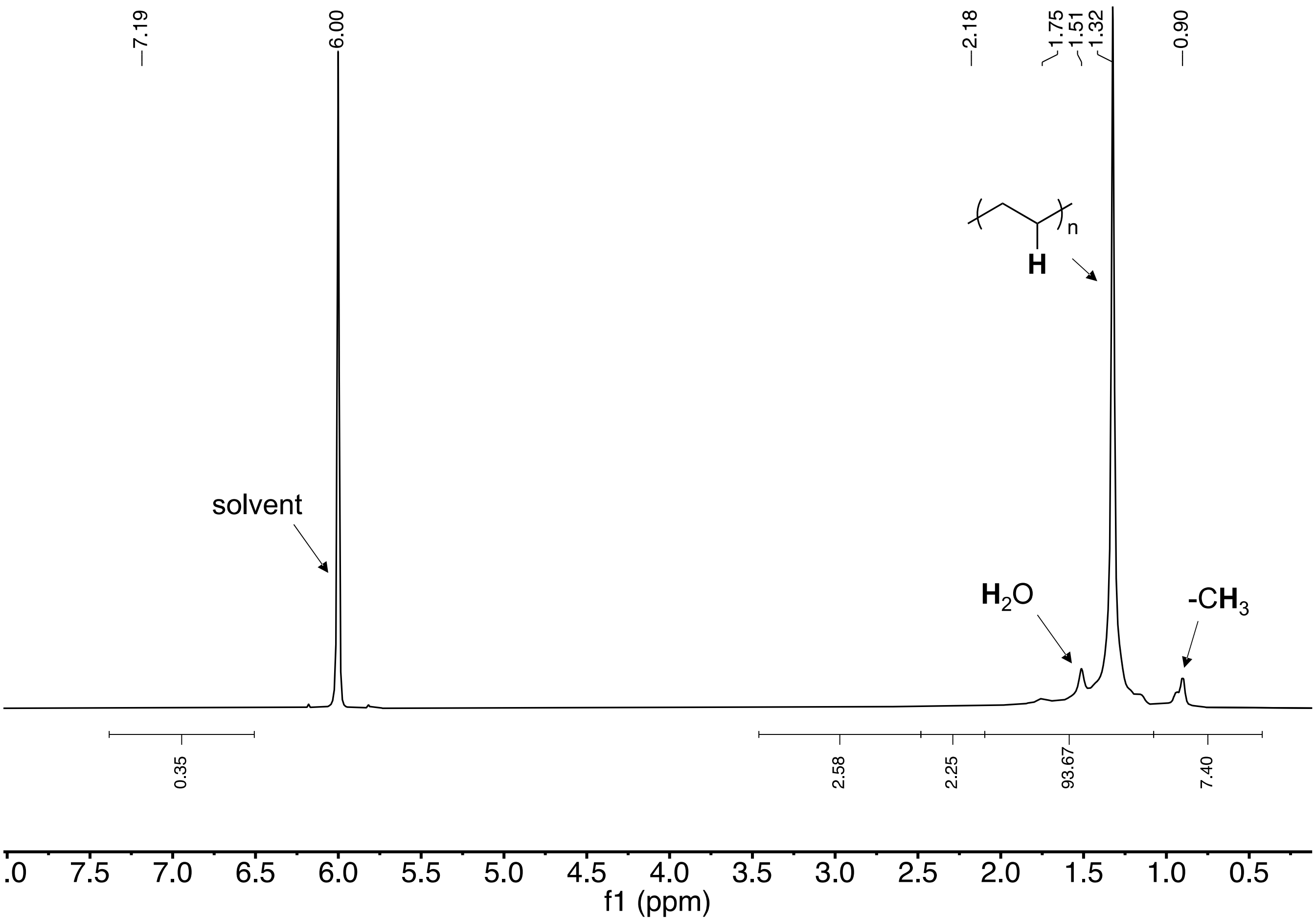

Fig. S43 ^{1}H NMR spectrum, in tetrachloroethane-d_2 (80 °C), of polyethylene product in Table 1, Entry 1a.

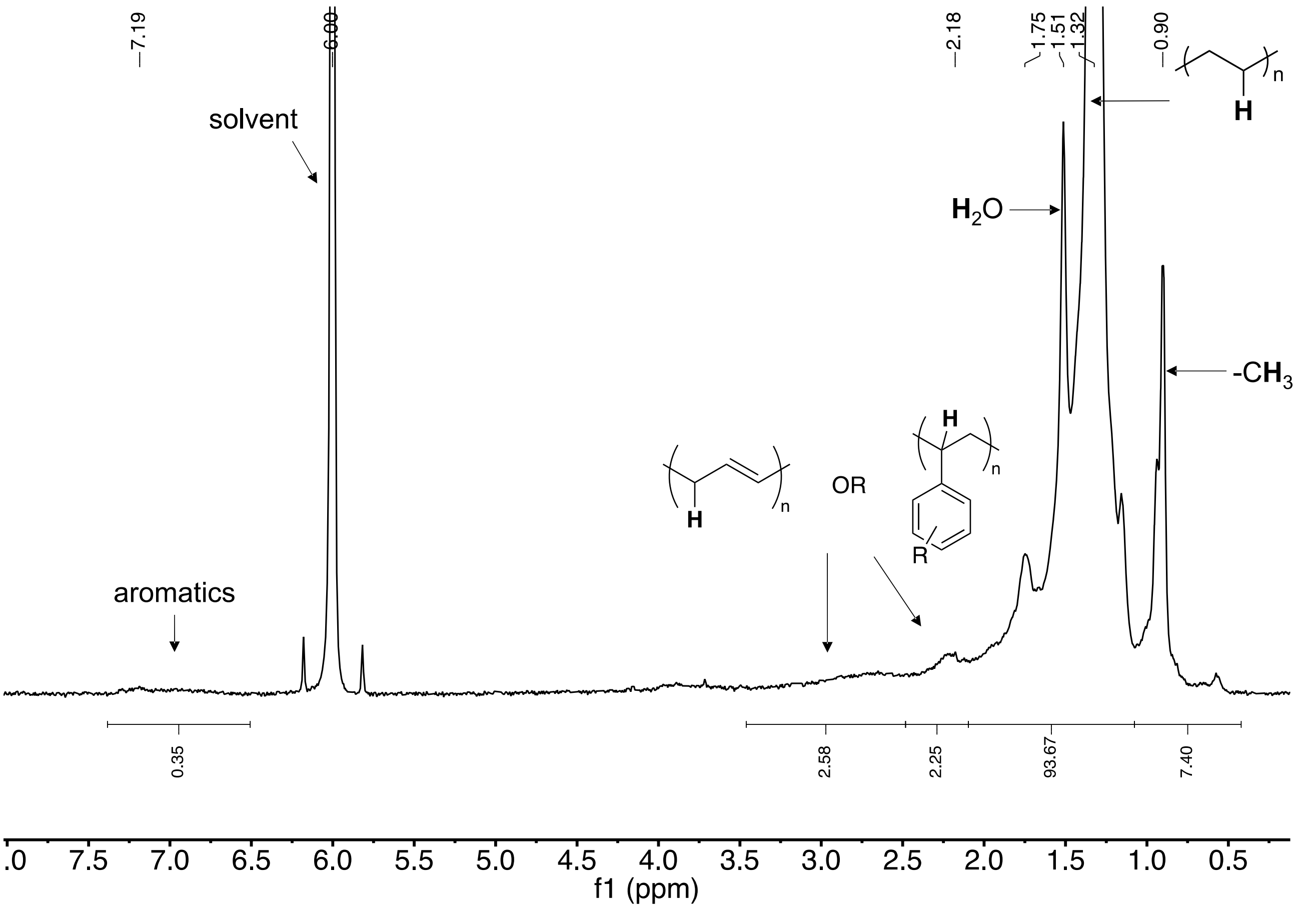

Fig. S43a Zoomed in ^{1}H NMR spectrum, in tetrachloroethane-d_2 (80 °C), of polyethylene product in Table 1, Entry 1a.

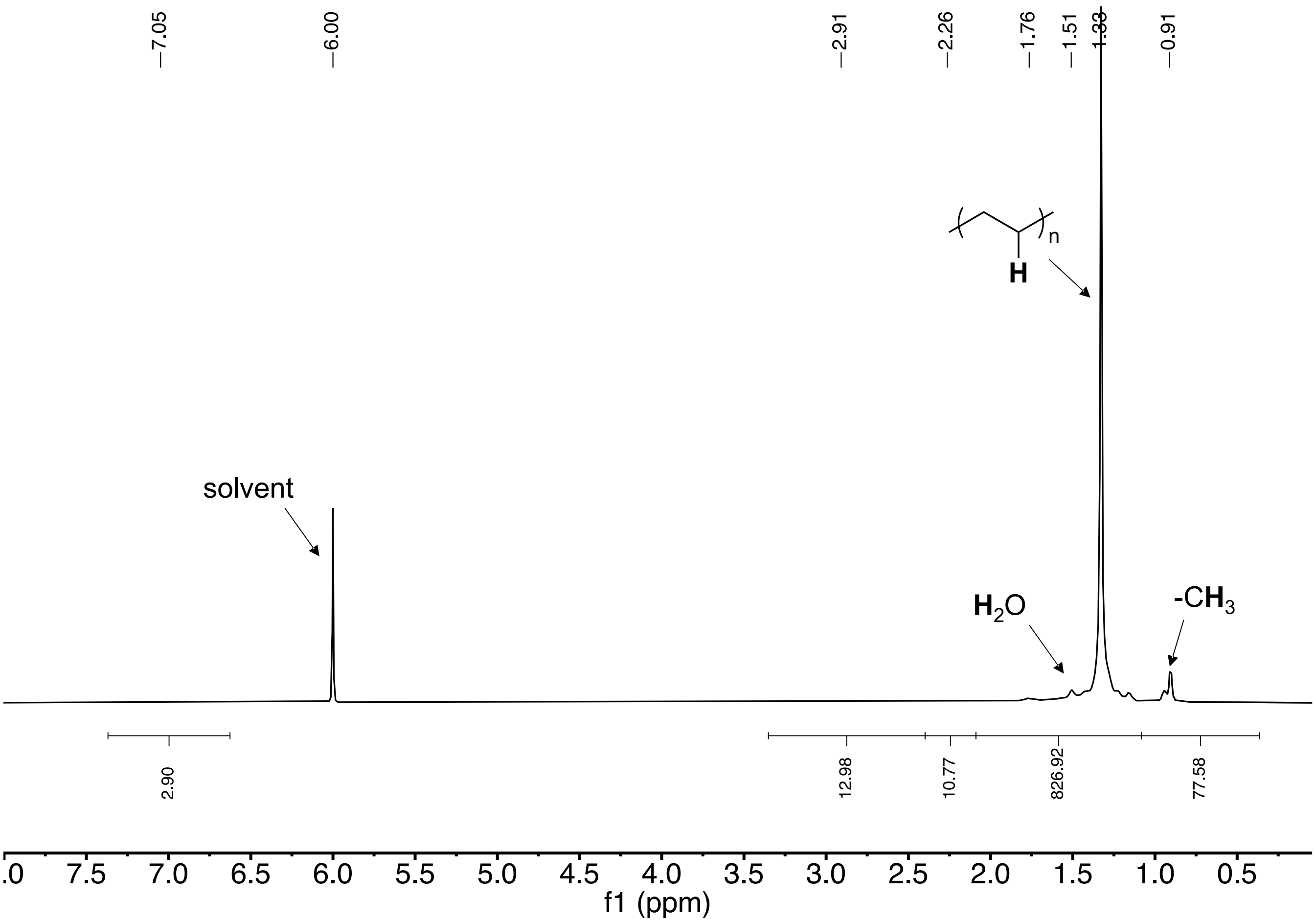

Fig. S44 ^{1}H NMR spectrum, in tetrachloroethane-d$_2$ (80 °C), of polyethylene product in Table 1, Entry 1b.

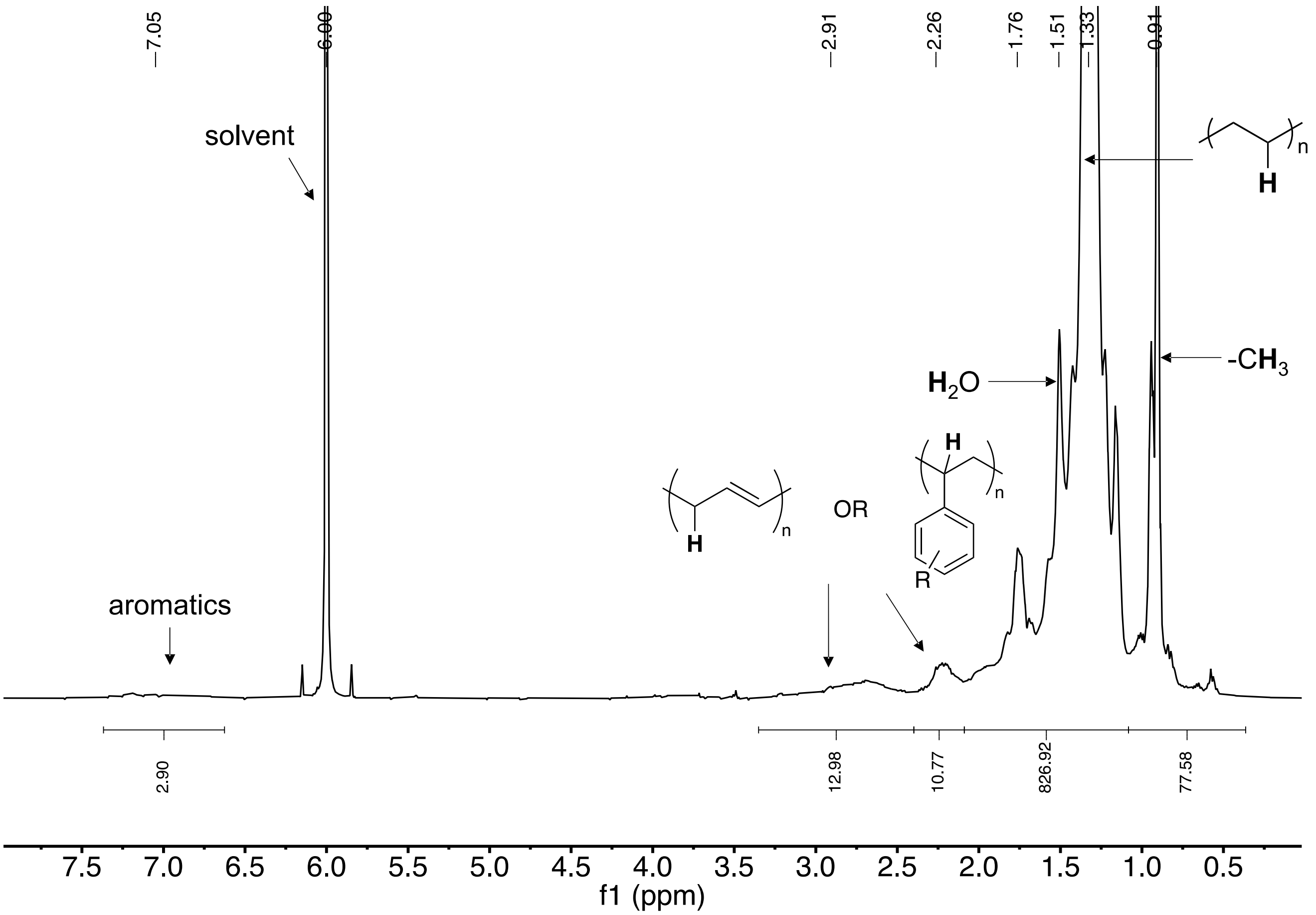

Fig. S44a Zoomed in ^{1}H NMR spectrum, in tetrachloroethane-d_2 (80 °C), of polyethylene product in Table 1, Entry 1b.

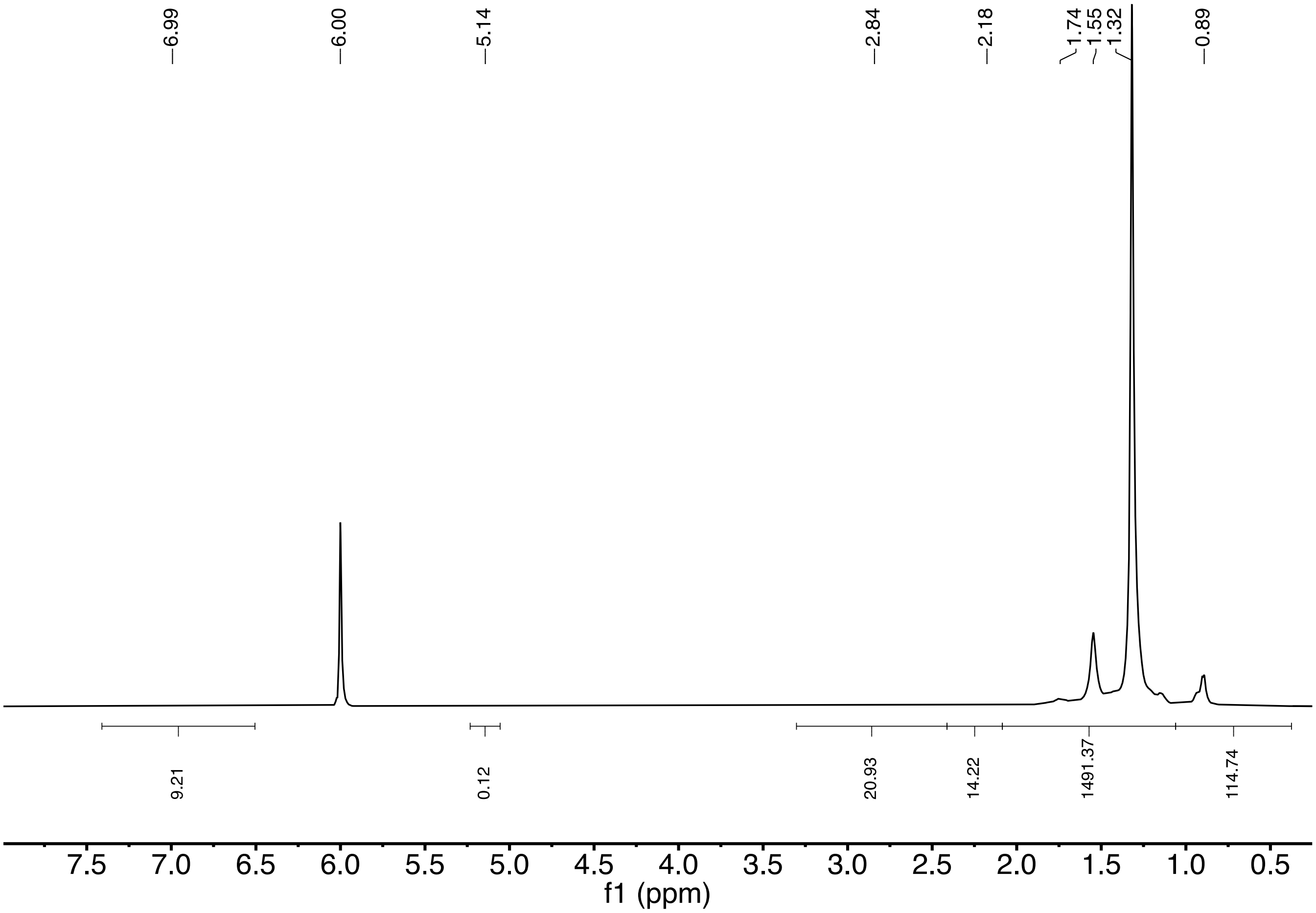

Fig. S45 ^{1}H NMR spectrum, in tetrachloroethane-d_2 (80 °C), of polyethylene product in Table 1, Entry 2a.

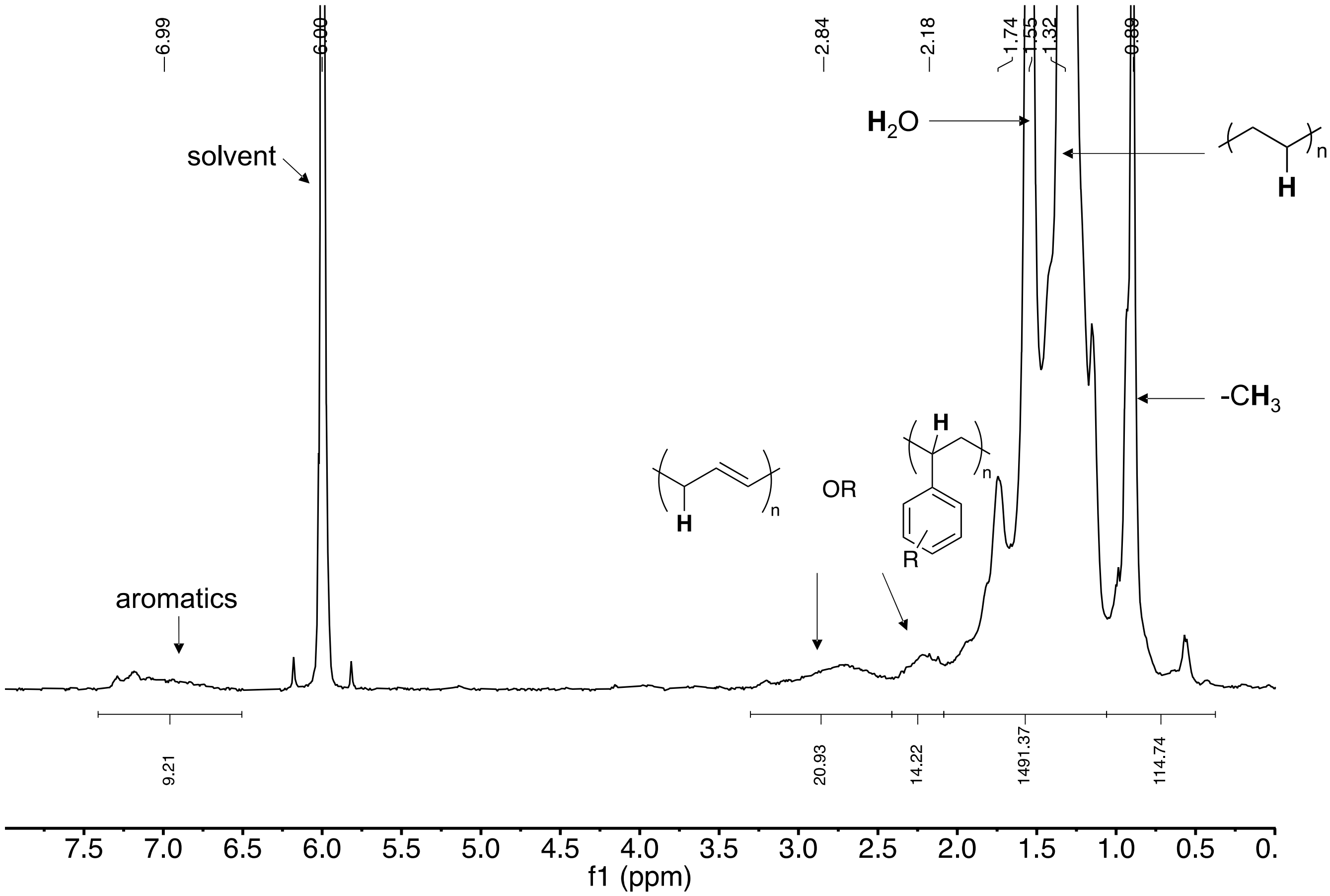

Fig. S45a Zoomed in ^{1}H NMR spectrum, in tetrachloroethane-d_2 (80 °C), of polyethylene product in Table 1, Entry 2a.

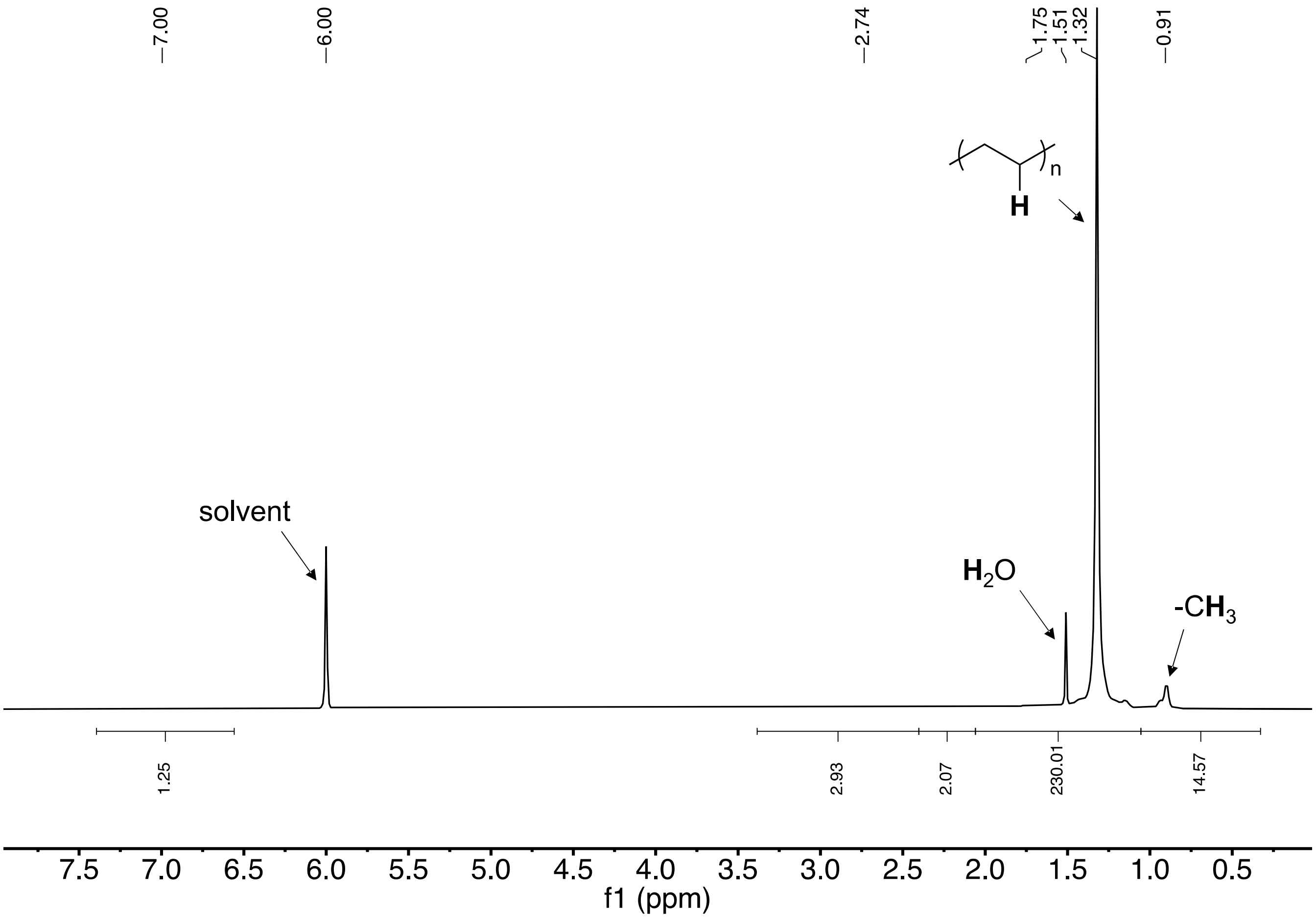

Fig. S46 ^{1}H NMR spectrum, in tetrachloroethane-d_2 (80 °C), of polyethylene product in Table 1, Entry 2b.

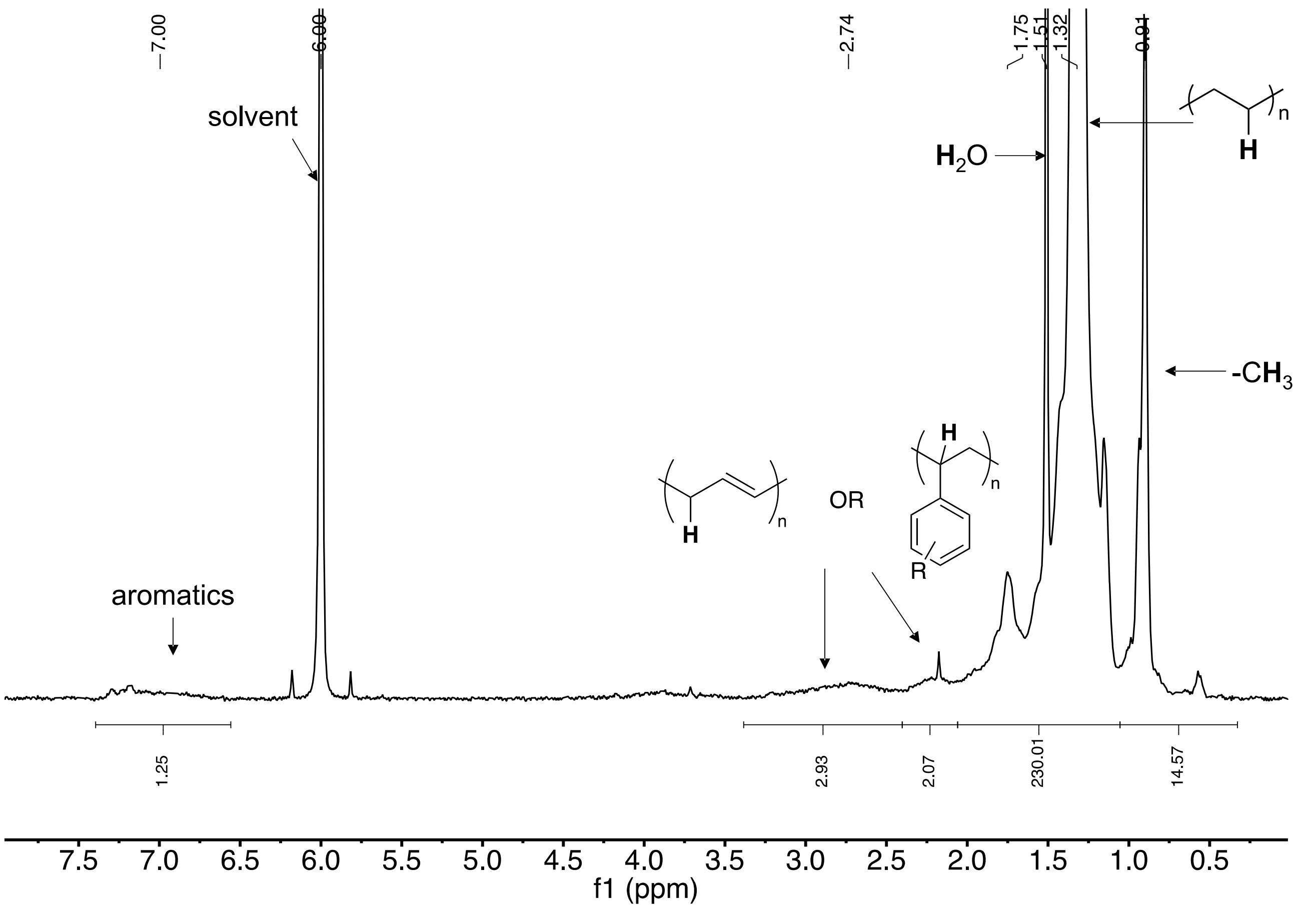

Fig. S46a Zoomed in ^{1}H NMR spectrum, in tetrachloroethane-d_2 (80 °C), of polyethylene product in Table 1, Entry 2b.

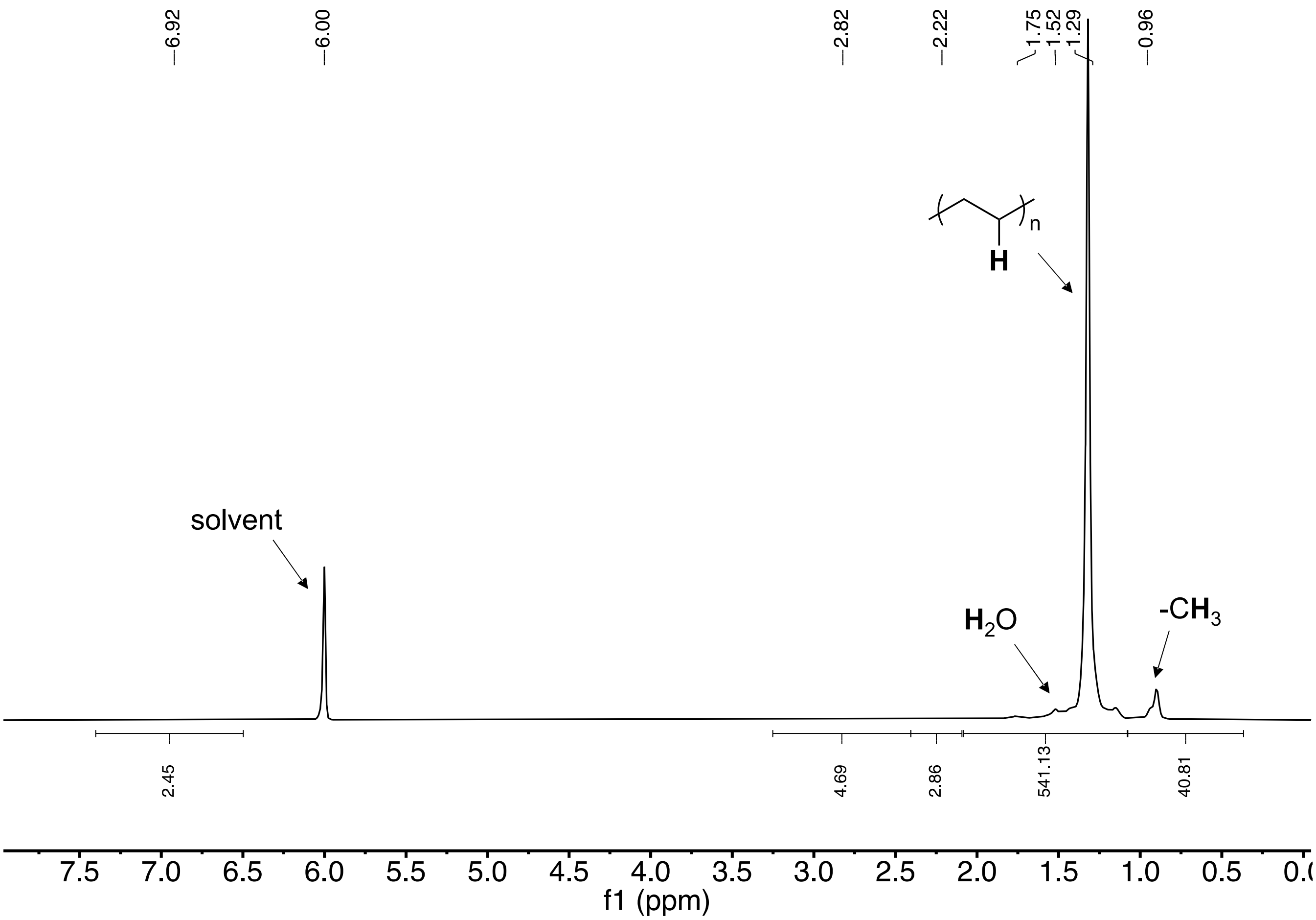

Fig. S47 ^{1}H NMR spectrum, in tetrachloroethane-d_2 (80 °C), of polyethylene product in Table 1, Entry 3a.

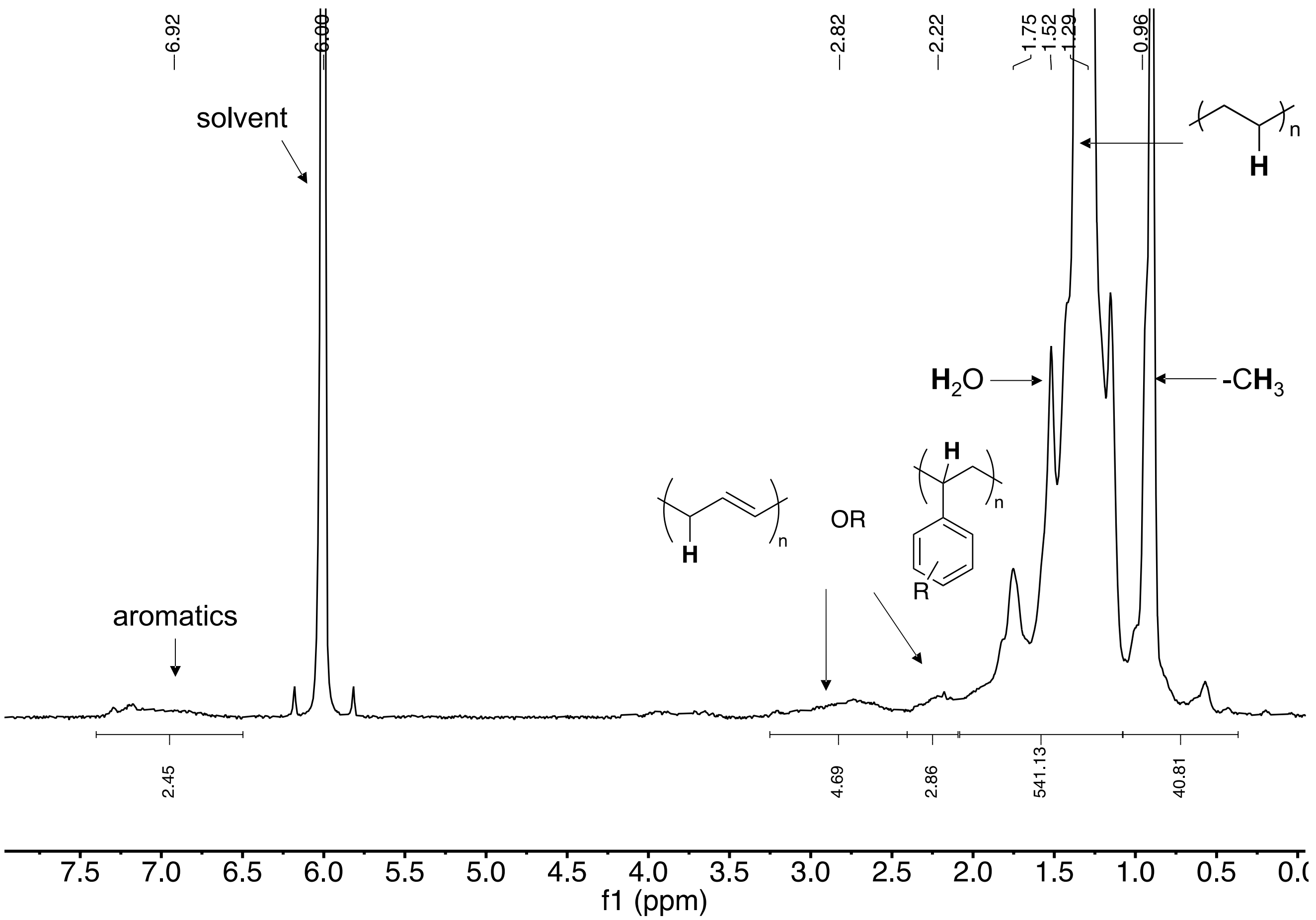

Fig. S47a Zoomed in ^{1}H NMR spectrum, in tetrachloroethane-d_2 (80 °C), of polyethylene product in Table 1, Entry 3a.

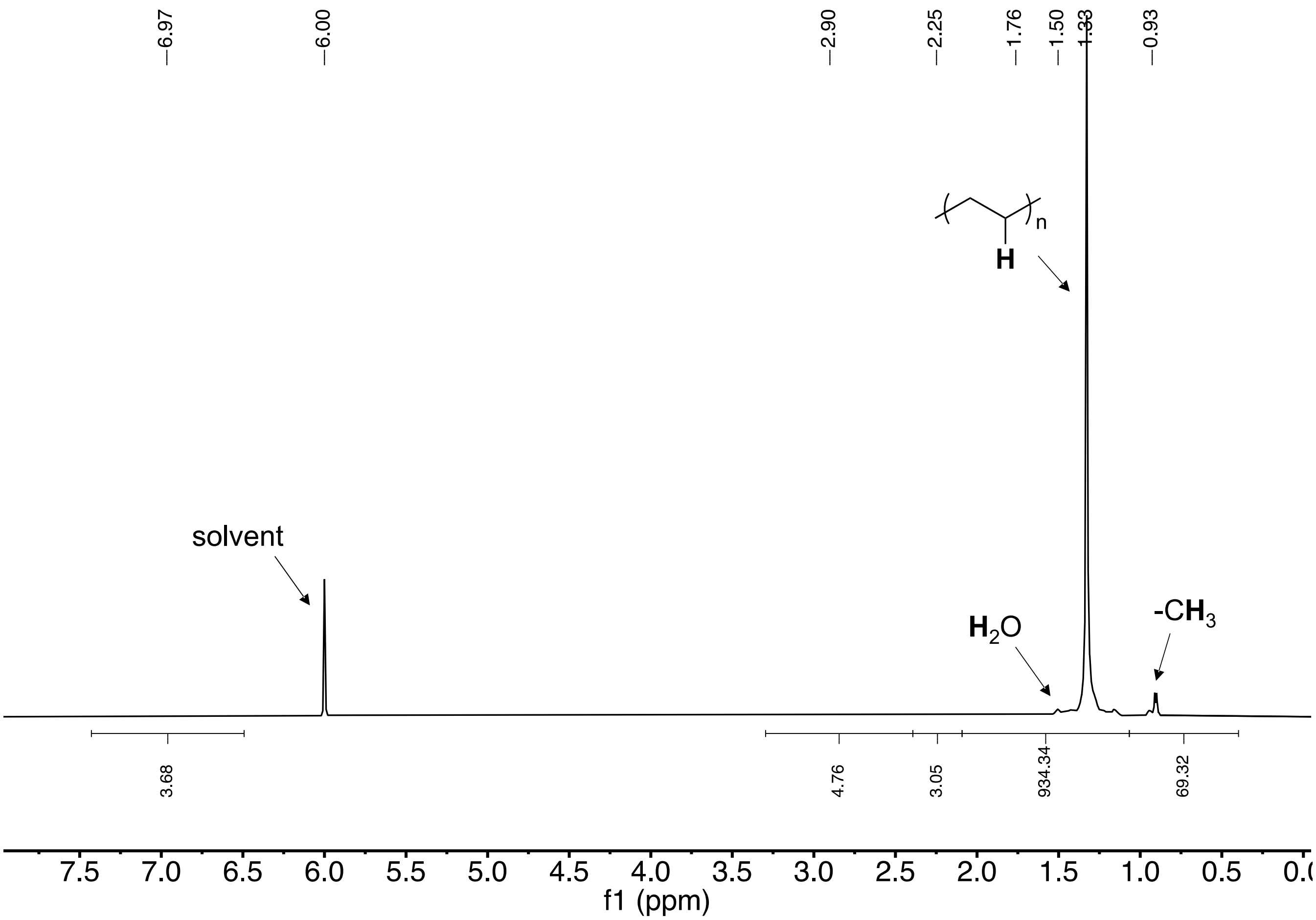

Fig. S48 ^{1}H NMR spectrum, in tetrachloroethane-d$_2$ (80 °C), of polyethylene product in Table 1, Entry 3b.

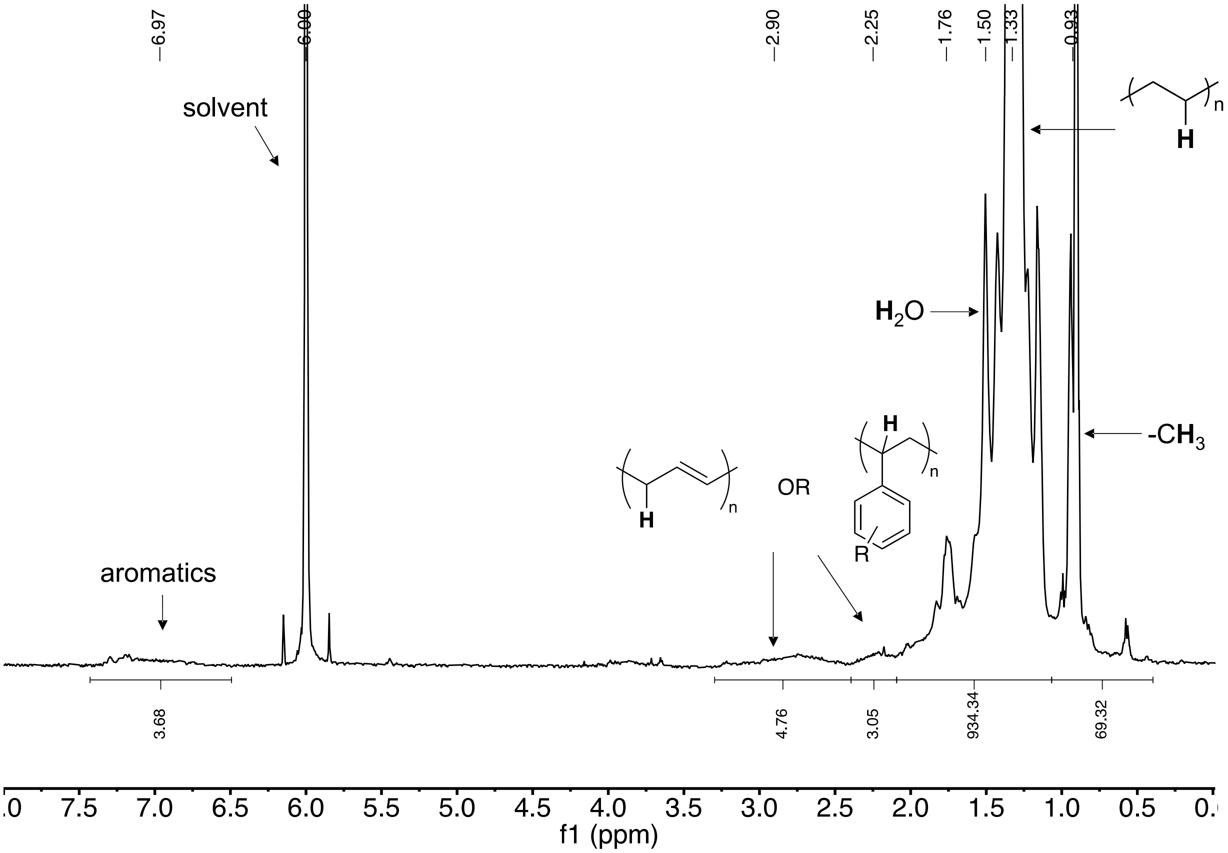

Fig. S48a Zoomed in ^{1}H NMR spectrum, in tetrachloroethane-d_2 (80 °C), of polyethylene product in Table 1, Entry 3b.

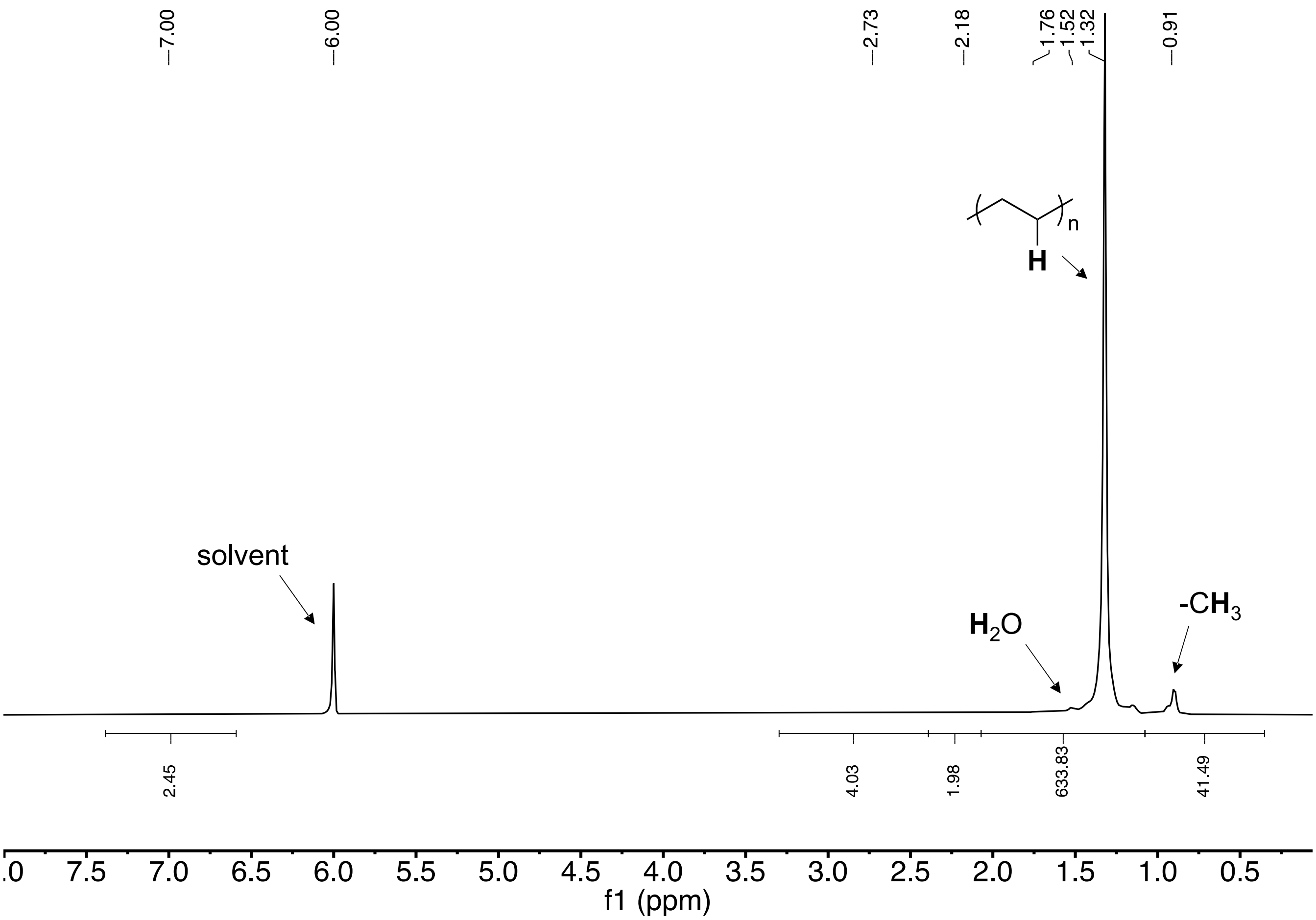

Fig. S49 ^{1}H NMR spectrum, in tetrachloroethane-d_2 (80 °C), of polyethylene product in Table 1, Entry 4a.

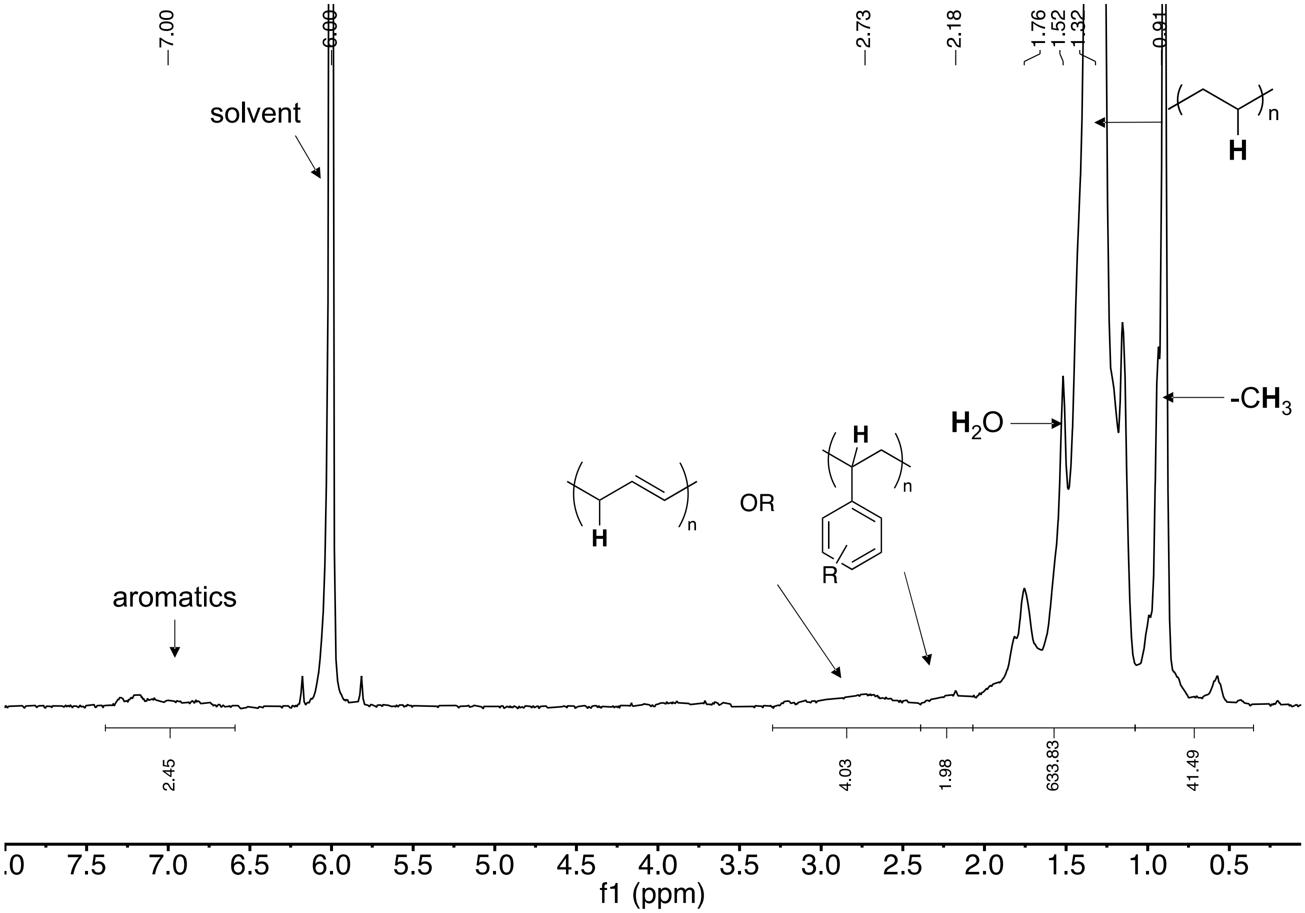

Fig. S49a Zoomed in ^{1}H NMR spectrum, in tetrachloroethane-d_2 (80 °C), of polyethylene product in Table 1, Entry 4a.

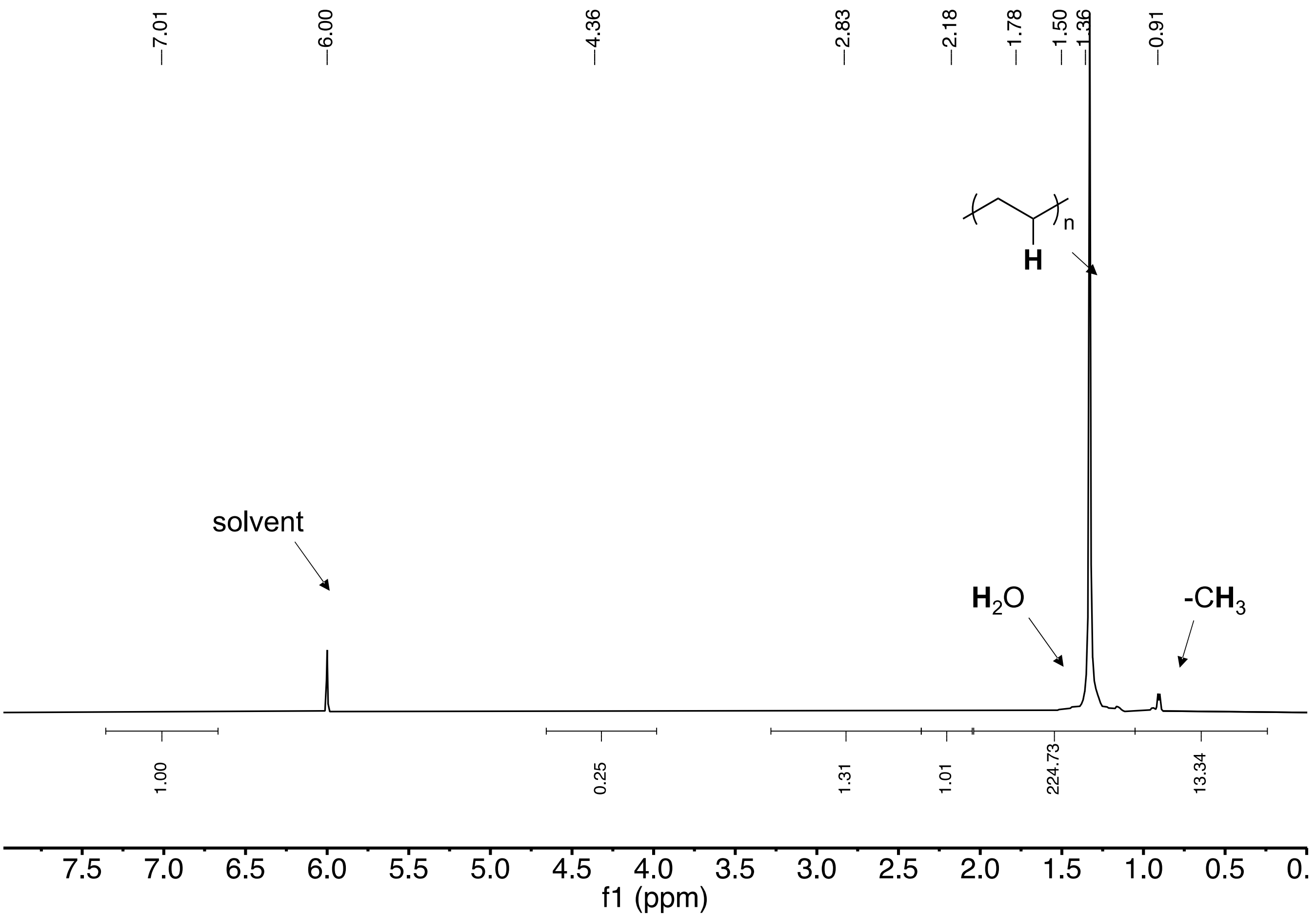

Fig. S50 ^{1}H NMR spectrum, in tetrachloroethane-d$_2$ (80 °C), of polyethylene product in Table 1, Entry 4b.

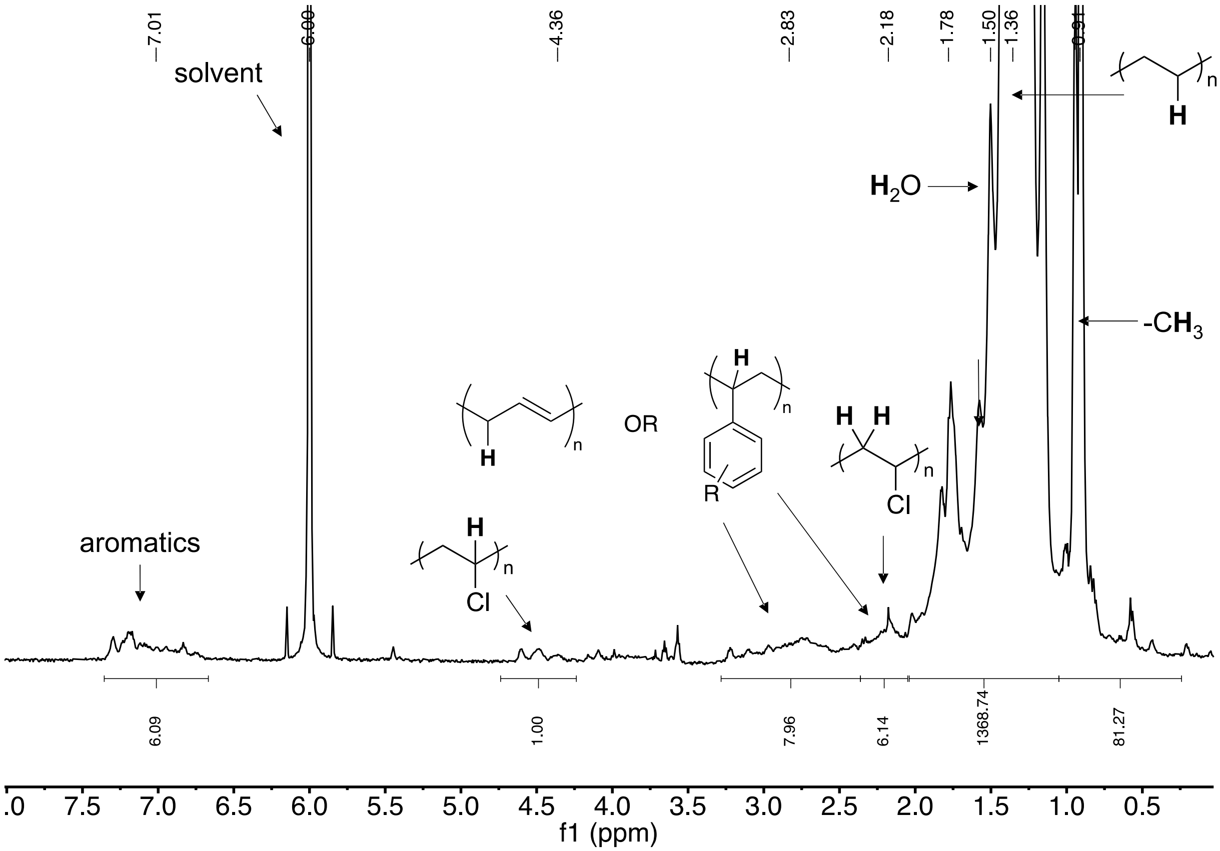

Fig. S50a Zoomed in ^{1}H NMR spectrum, in tetrachloroethane-d_2 (80 °C), of polyethylene product in Table 1, Entry 4b.

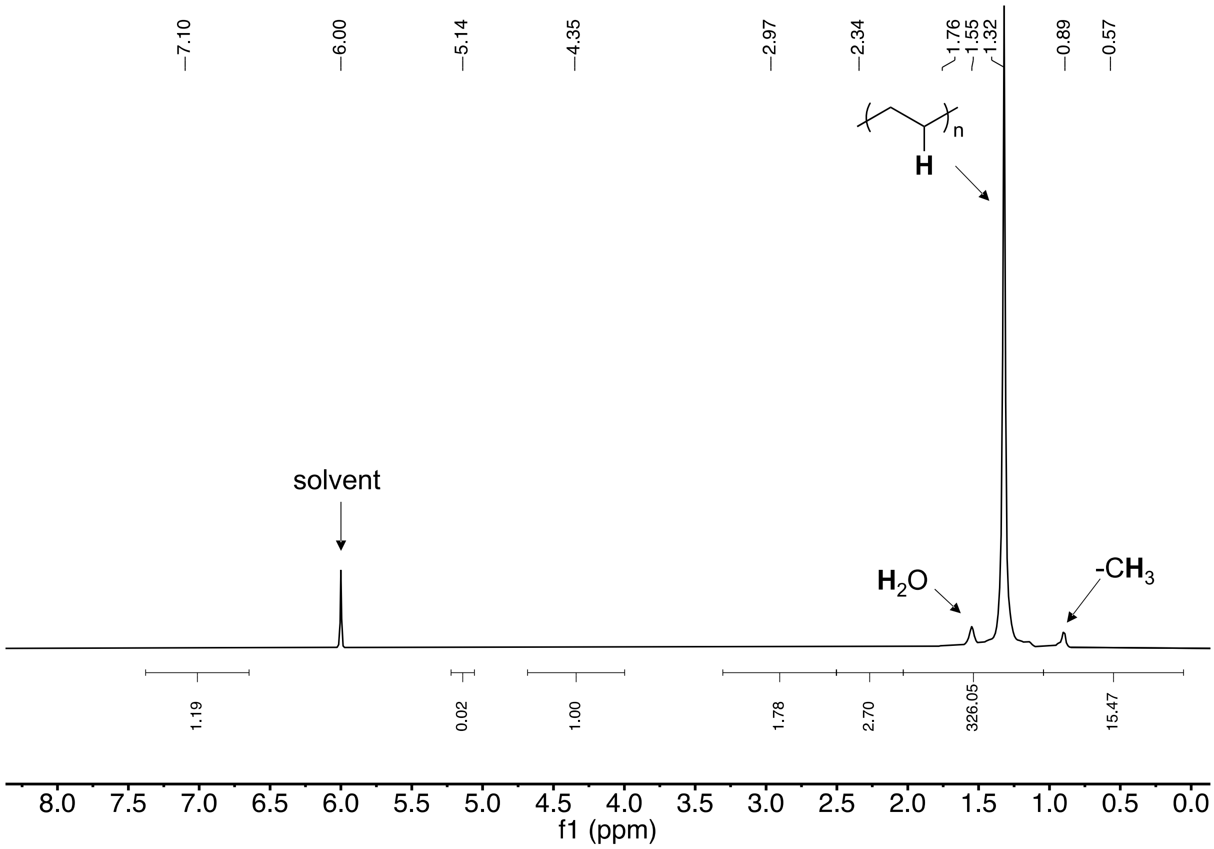

Fig. S51 ^{1}H NMR spectrum, in tetrachloroethane-d$_2$ (80 °C), of polyethylene product in Table 1, Entry 5a.

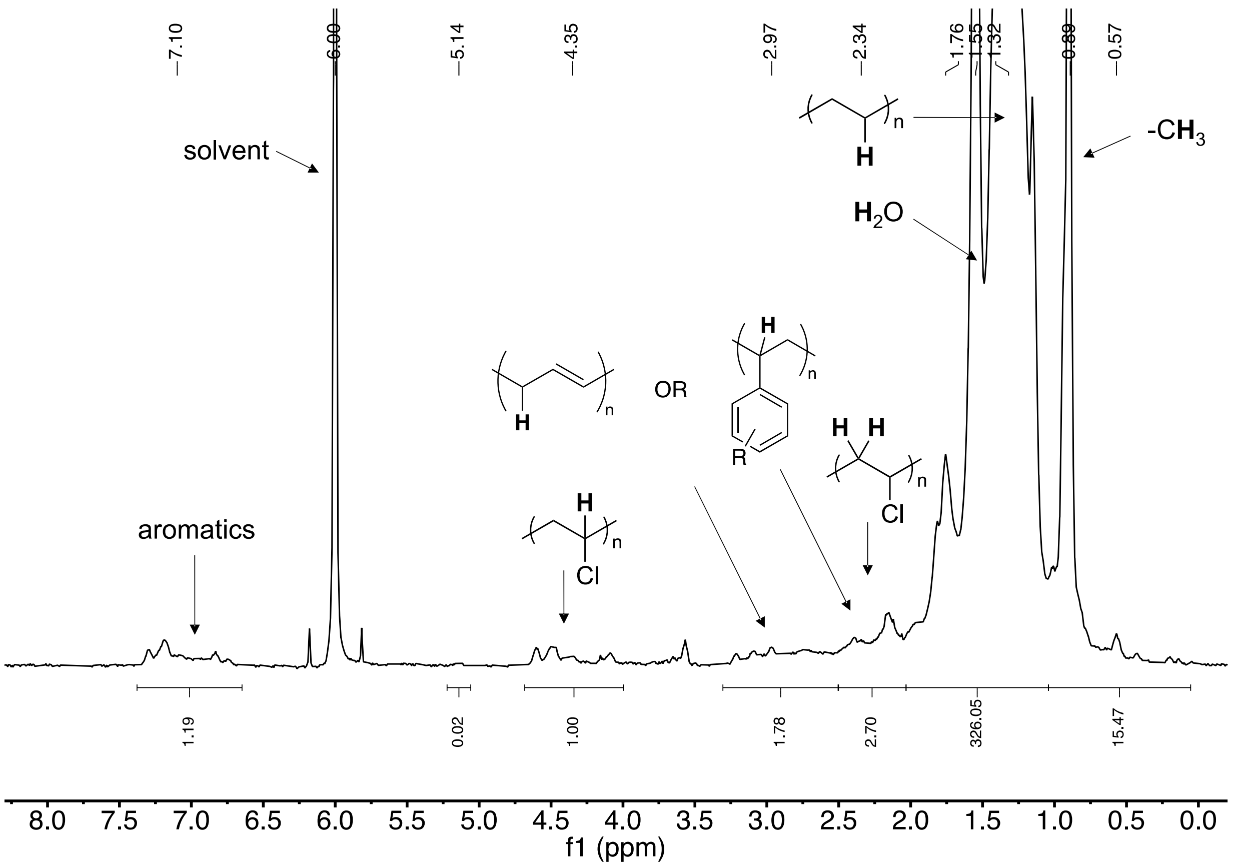

Fig. S51a Zoomed in ^{1}H NMR spectrum, in tetrachloroethane-d_2 (80 °C), of polyethylene product in Table 1, Entry 5a.

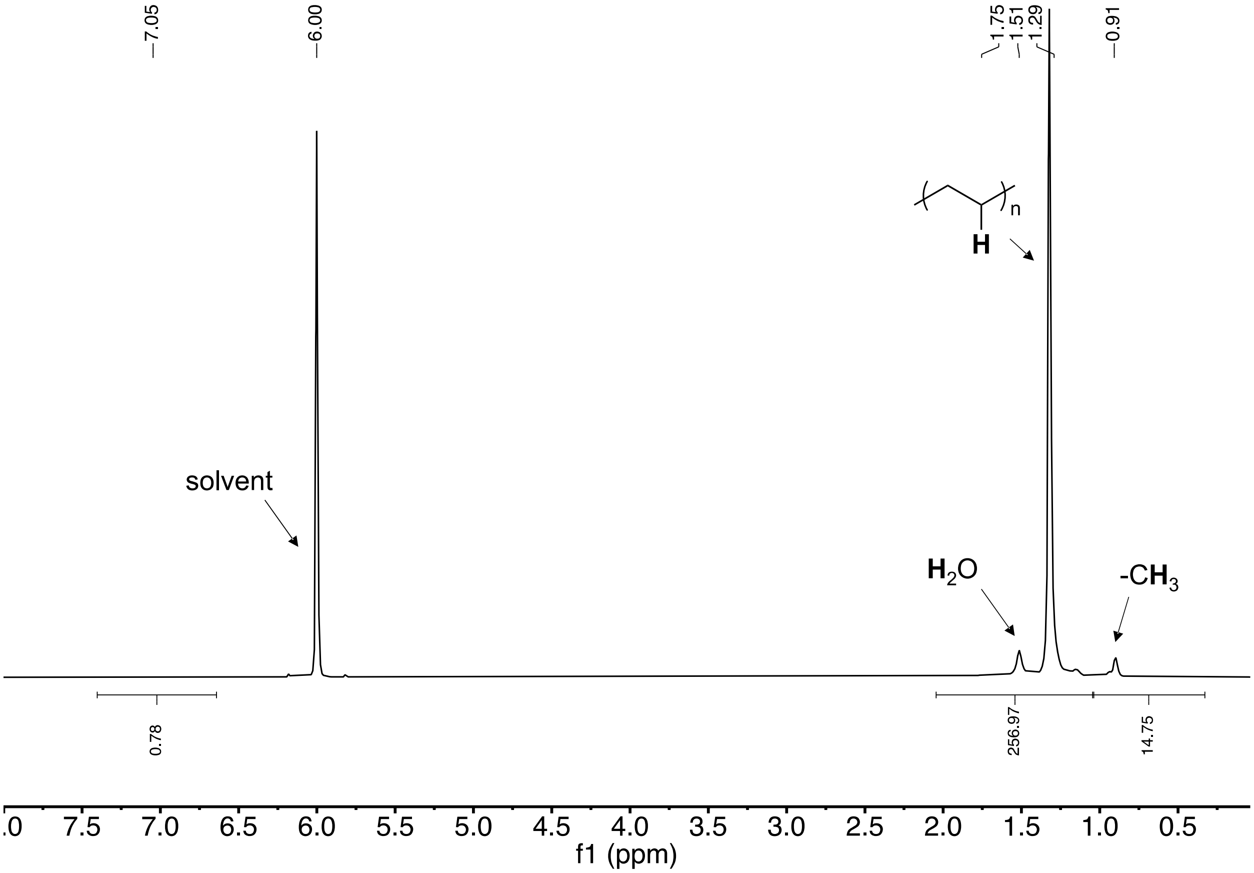

Fig. S52 ^{1}H NMR spectrum, in tetrachloroethane-d_2 (80 °C), of polyethylene product in Table 1, Entry 5b.

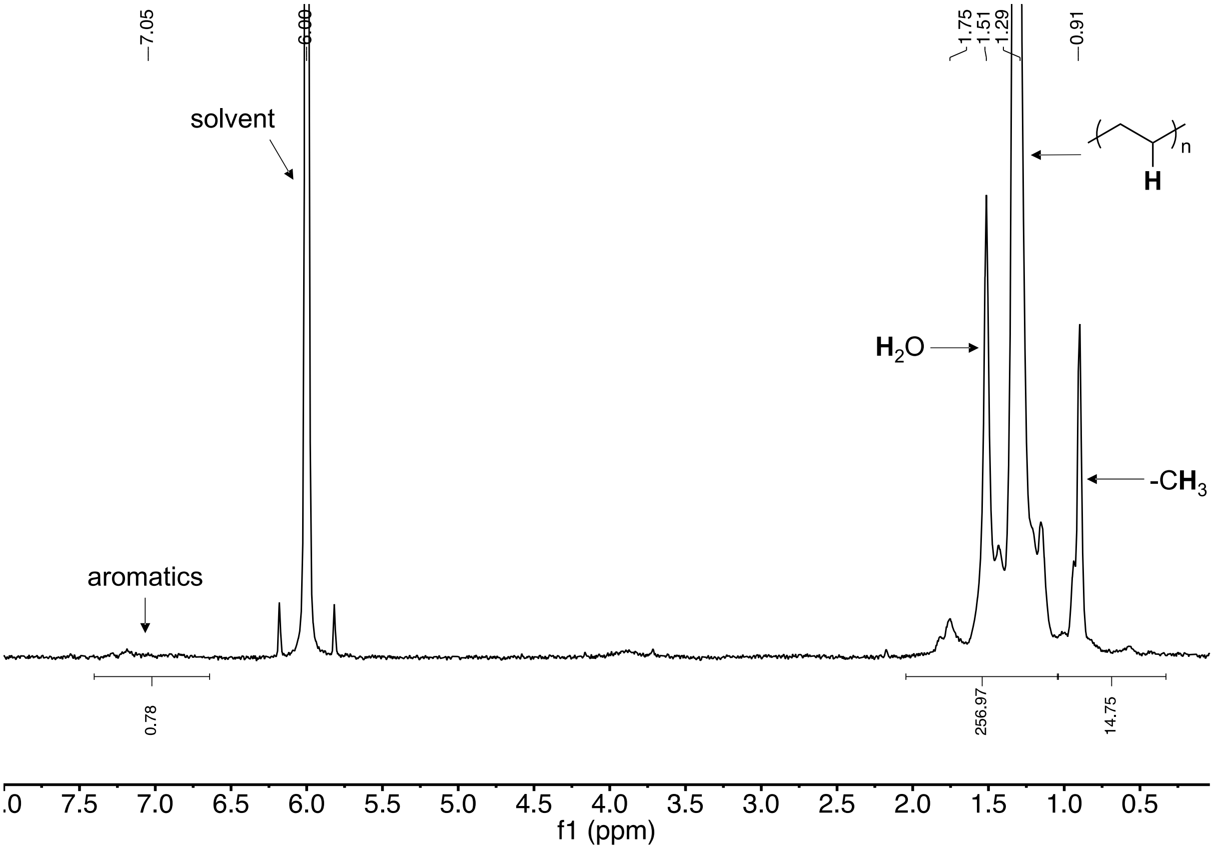

Fig. S52a Zoomed in ^{1}H NMR spectrum, in tetrachloroethane-d_2 (80 °C), of polyethylene product in Table 1, Entry 5b.

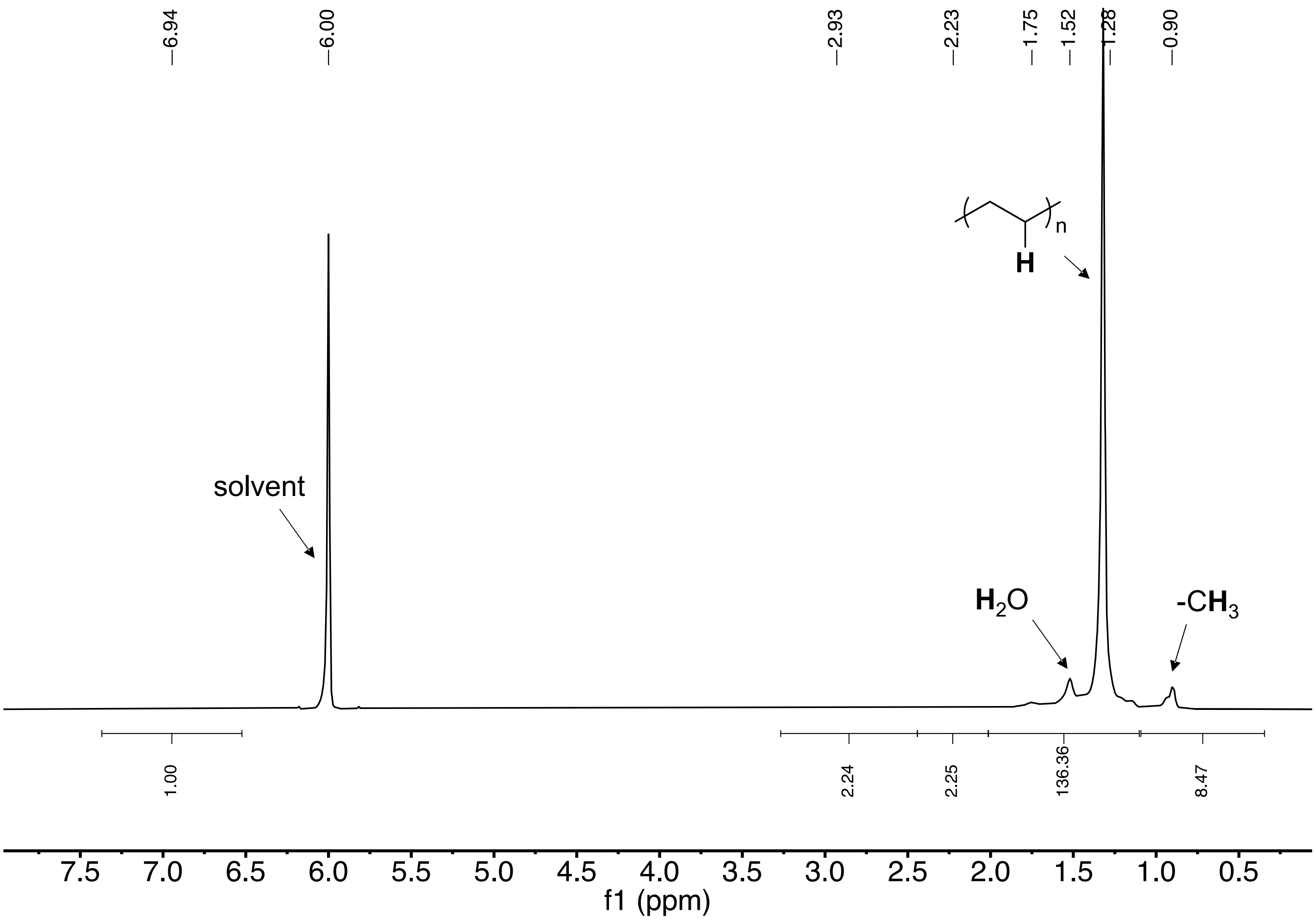

Fig. S53 ^{1}H NMR spectrum, in tetrachloroethane-d$_2$ (80 °C), of polyethylene product in Table 1, Entry 6a.

‘

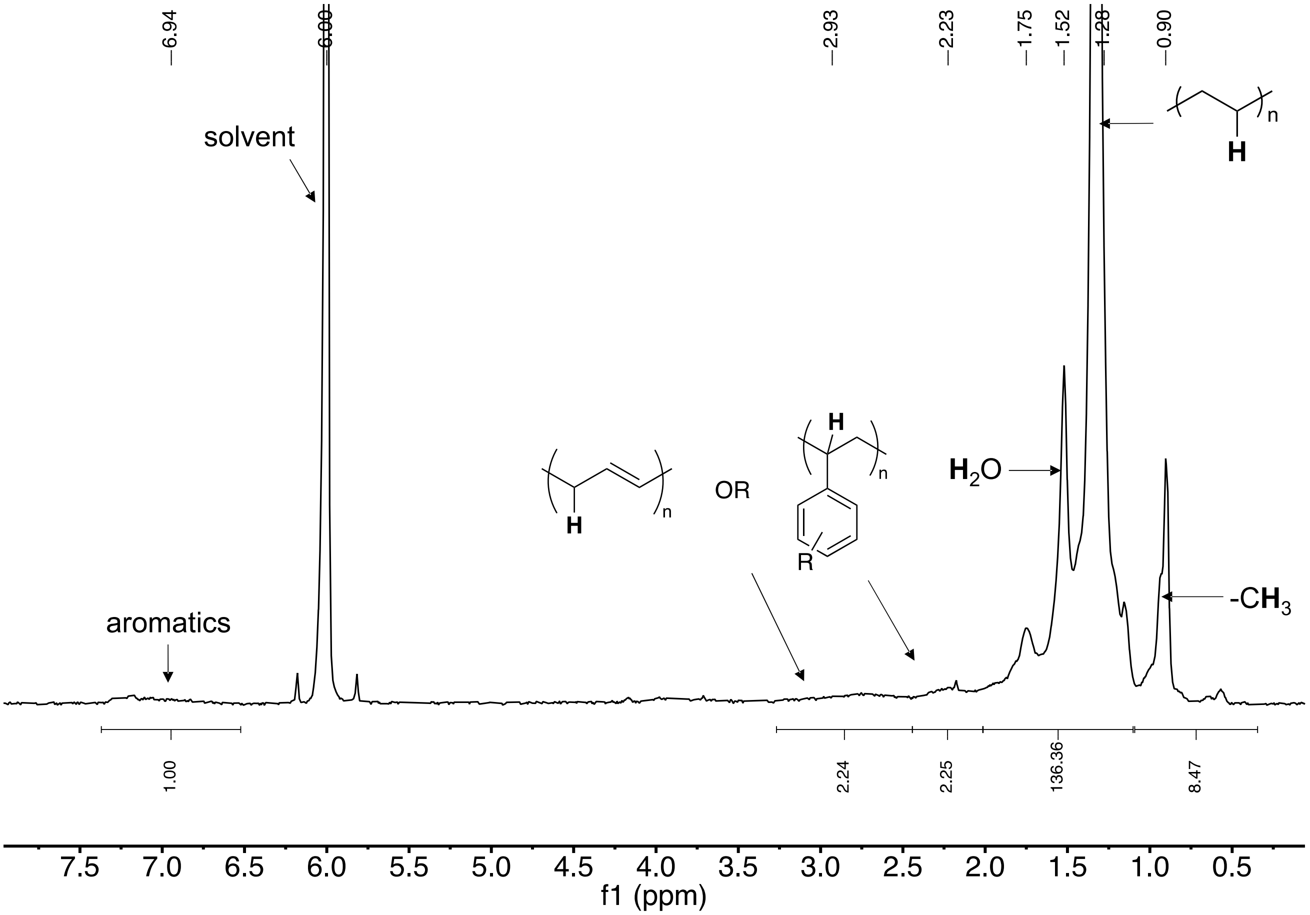

Fig. S53a Zoomed in ^{1}H NMR spectrum, in tetrachloroethane-d_2 (80 °C), of polyethylene product in Table 1, Entry 6a.

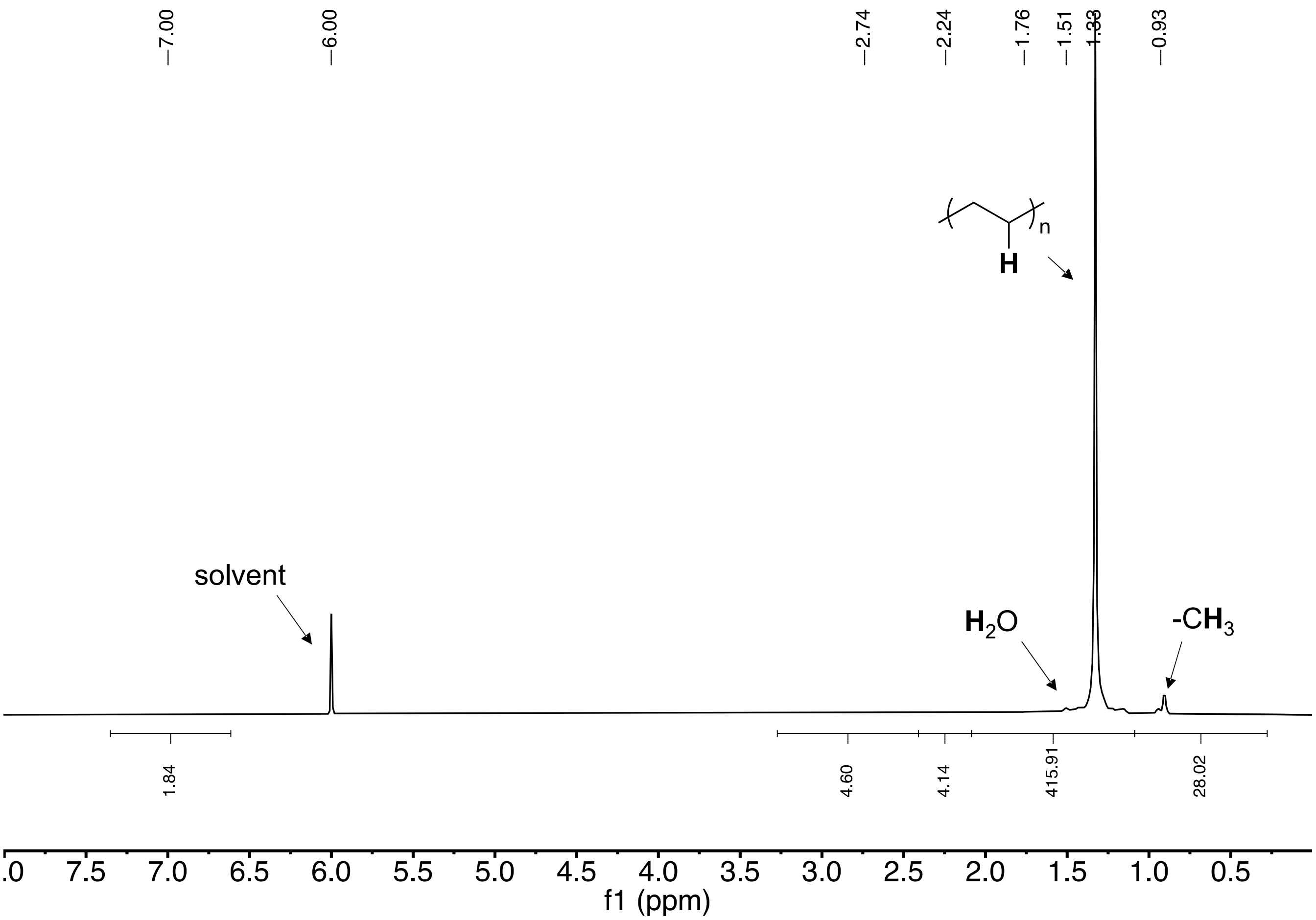

Fig. S54 ^{1}H NMR spectrum, in tetrachloroethane-d_2 (80 °C), of polyethylene product in Table 1, Entry 6b.

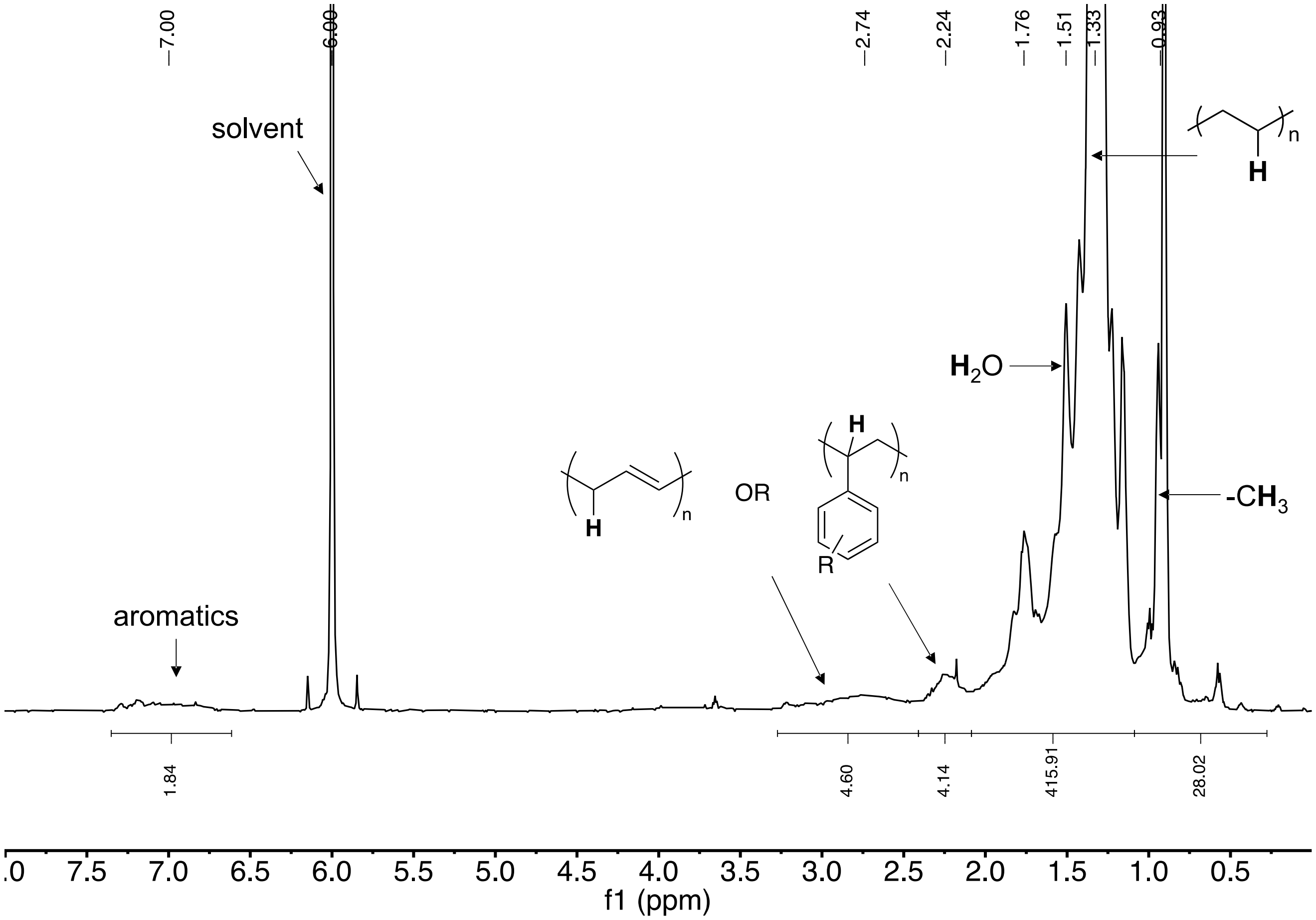

Fig. S54a Zoomed in ^{1}H NMR spectrum, in tetrachloroethane-d$_2$ (80 °C), of polyethylene product in Table 1, Entry 6b.

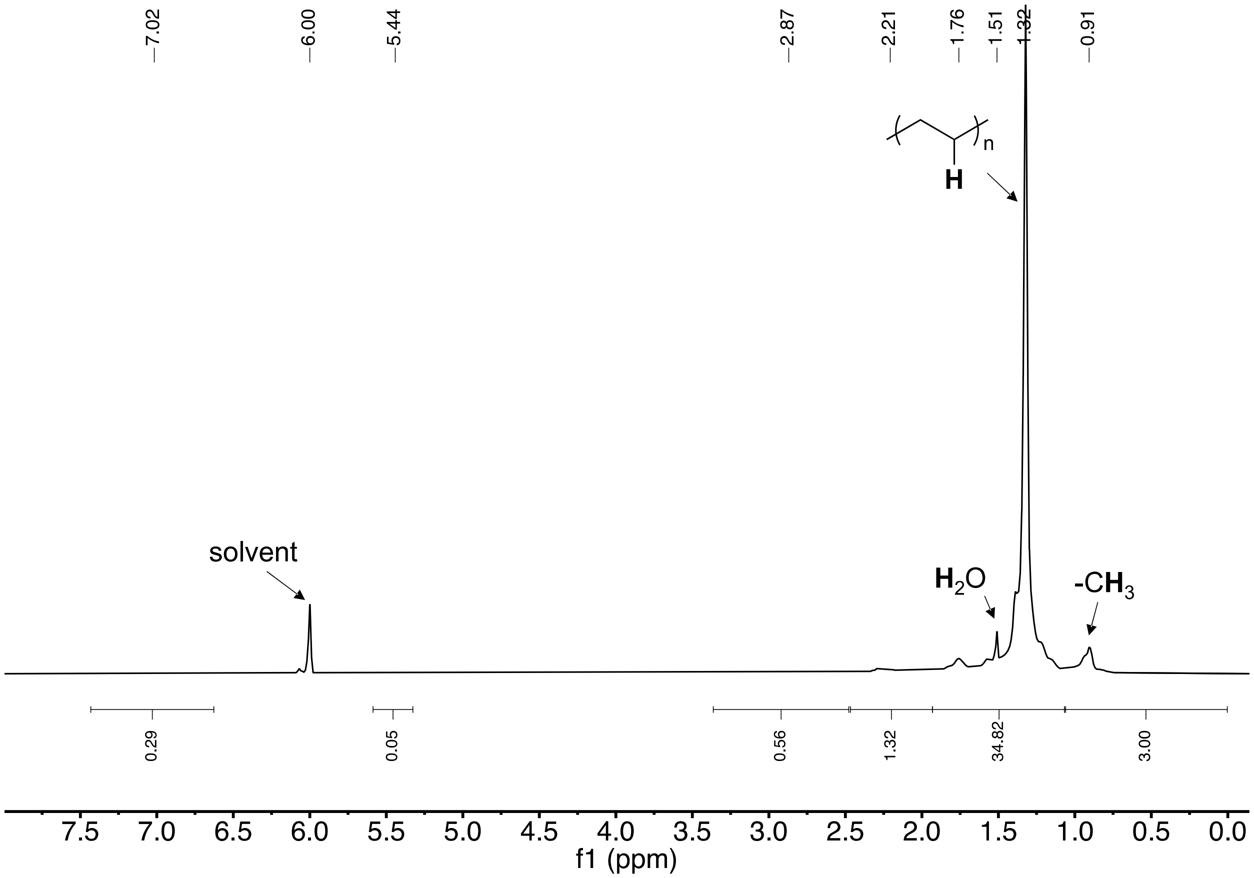

Fig. S55 ^{1}H NMR spectrum, in tetrachloroethane-d_2 (80 °C), of polyethylene product in Table 2, Entry 1 (toy lizard).

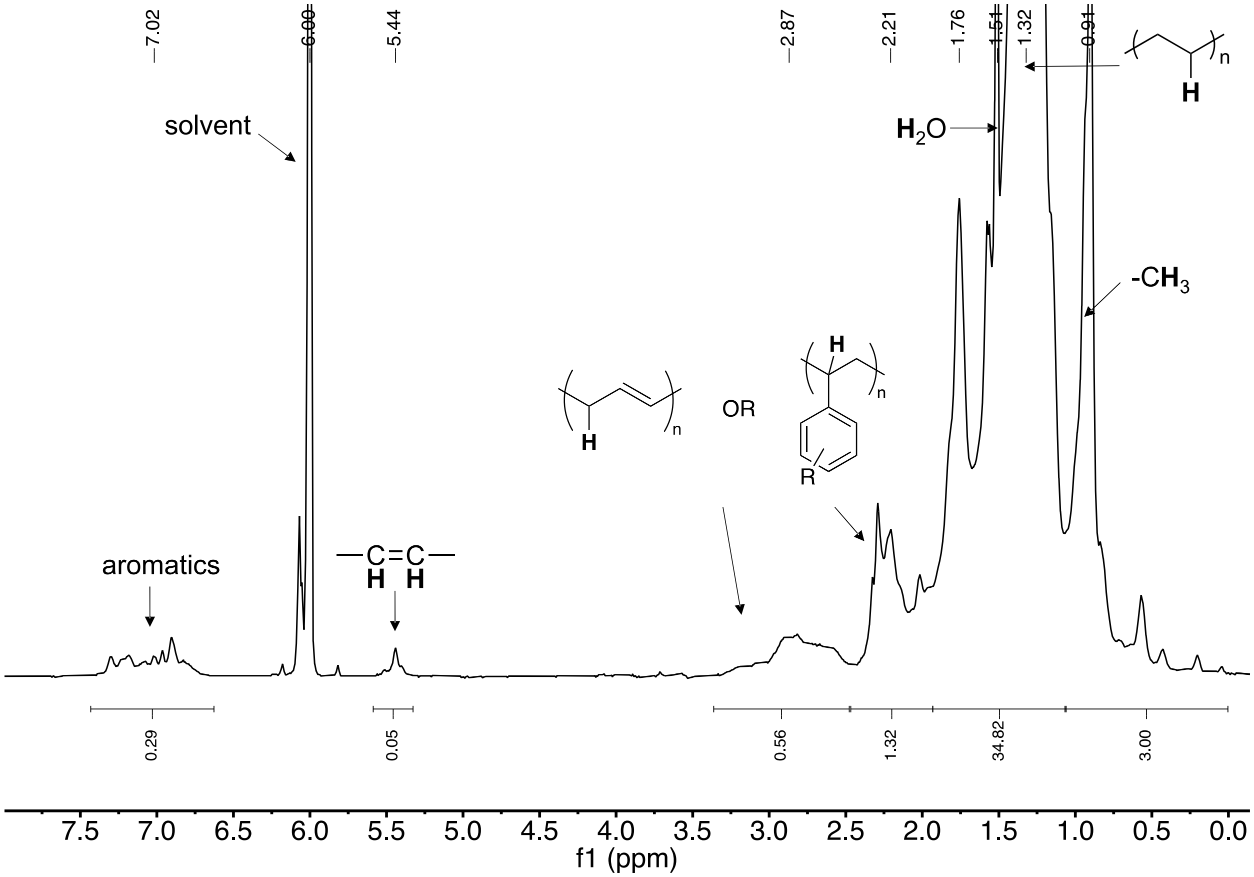

Fig. S55a Zoomed in ^{1}H NMR spectrum, in tetrachloroethane-d_2 (80 °C), of polyethylene product in Table 2, Entry 1 (toy lizard).

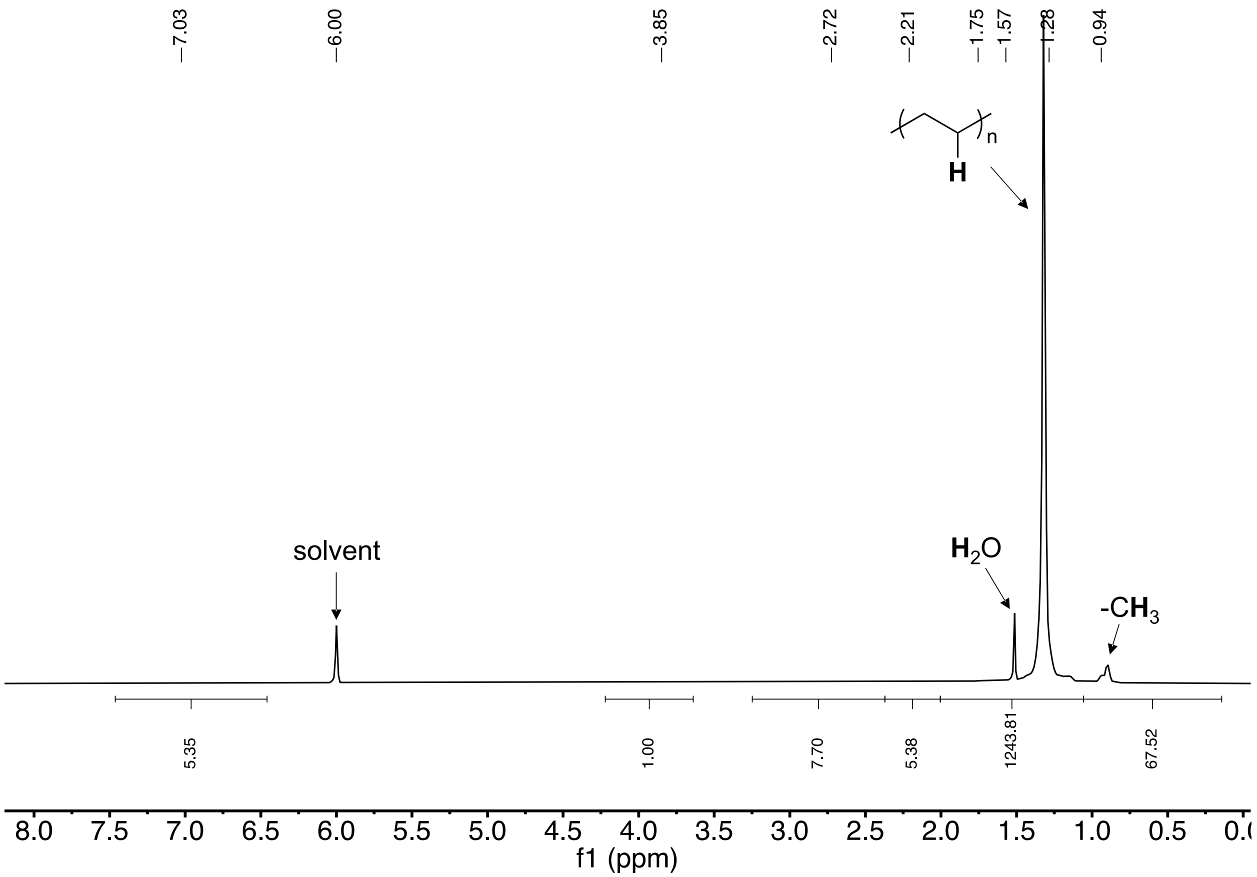

Fig. S56 ^{1}H NMR spectrum, in tetrachloroethane-d_2 (80 °C), of polyethylene product in Table 2, Entry 2 (extracted toy lizard).

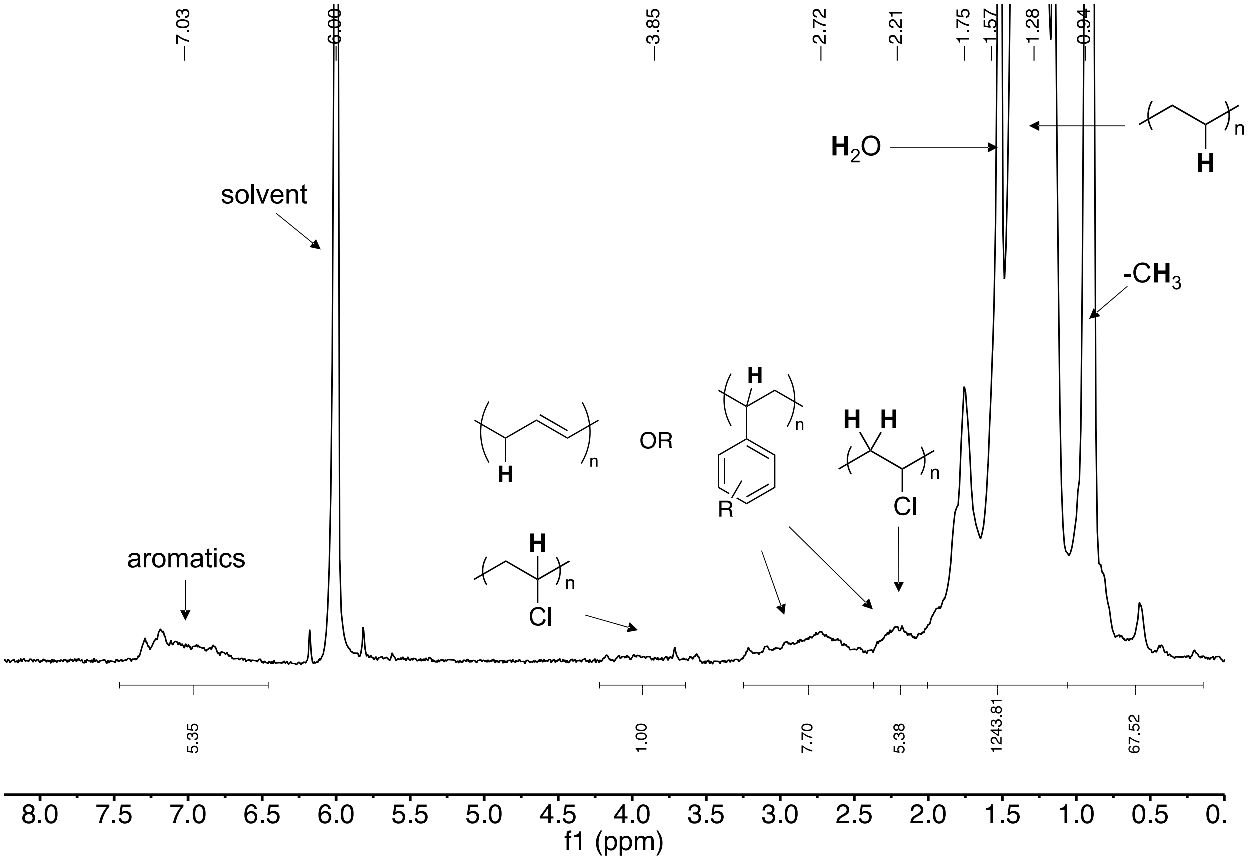

Fig. S56a Zoomed in ^{1}H NMR spectrum, in tetrachloroethane-d_2 (80 °C), of polyethylene product in Table 2, Entry 2 (extracted toy lizard).

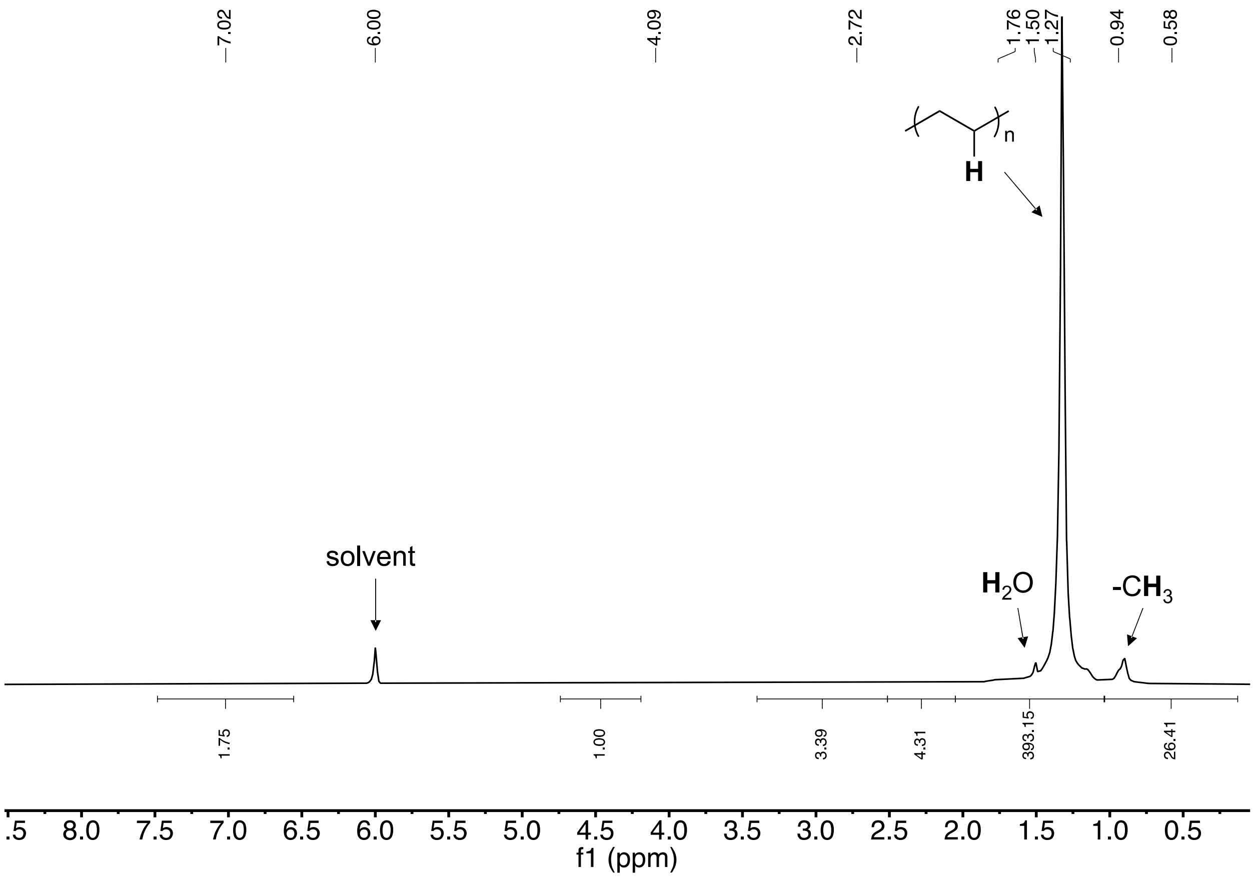

Fig. S57 ^1H NMR spectrum, in tetrachloroethane-d_2 (80 °C), of polyethylene product in Table 2, Entry 3 (rigid PVC pipe).

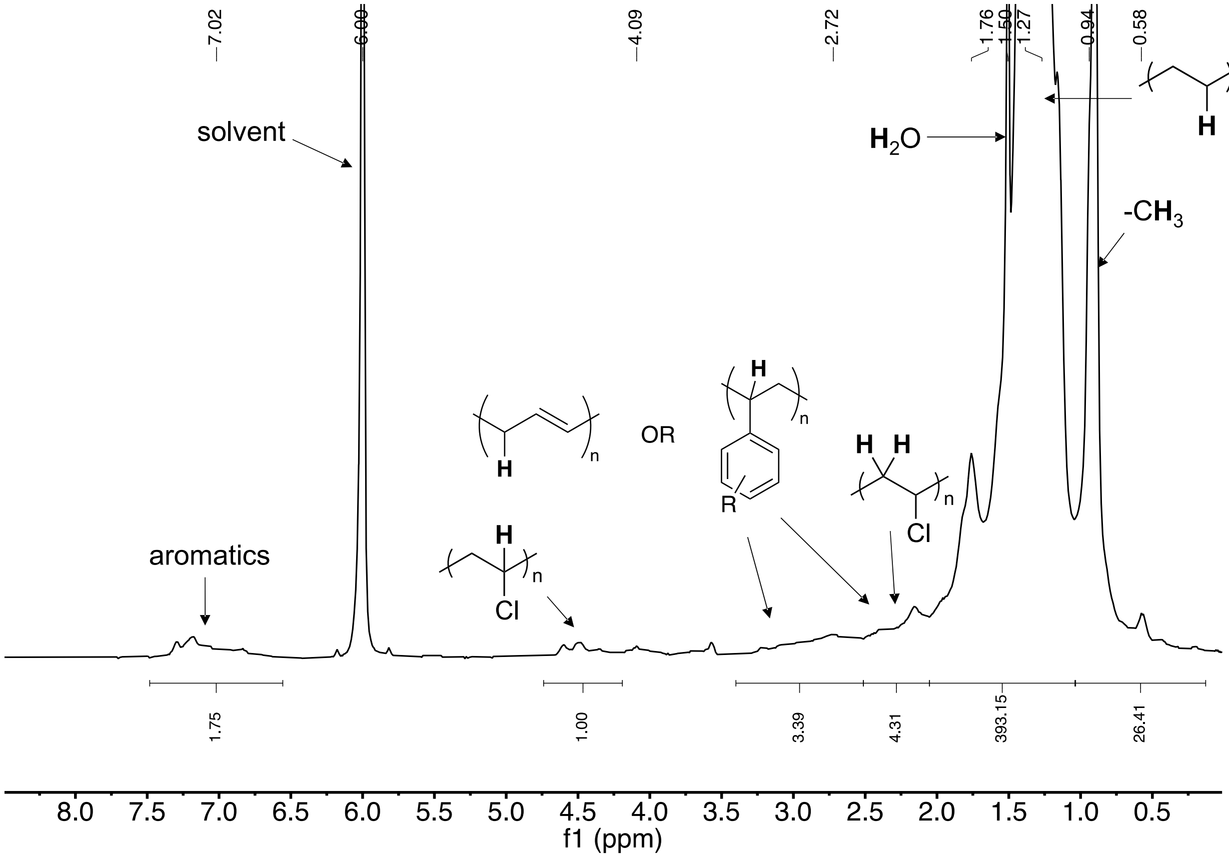

Fig. S57a Zoomed in ^{1}H NMR spectrum, in tetrachloroethane-d_2 (80 °C), of polyethylene product in Table 2, Entry 3 (rigid PVC pipe).

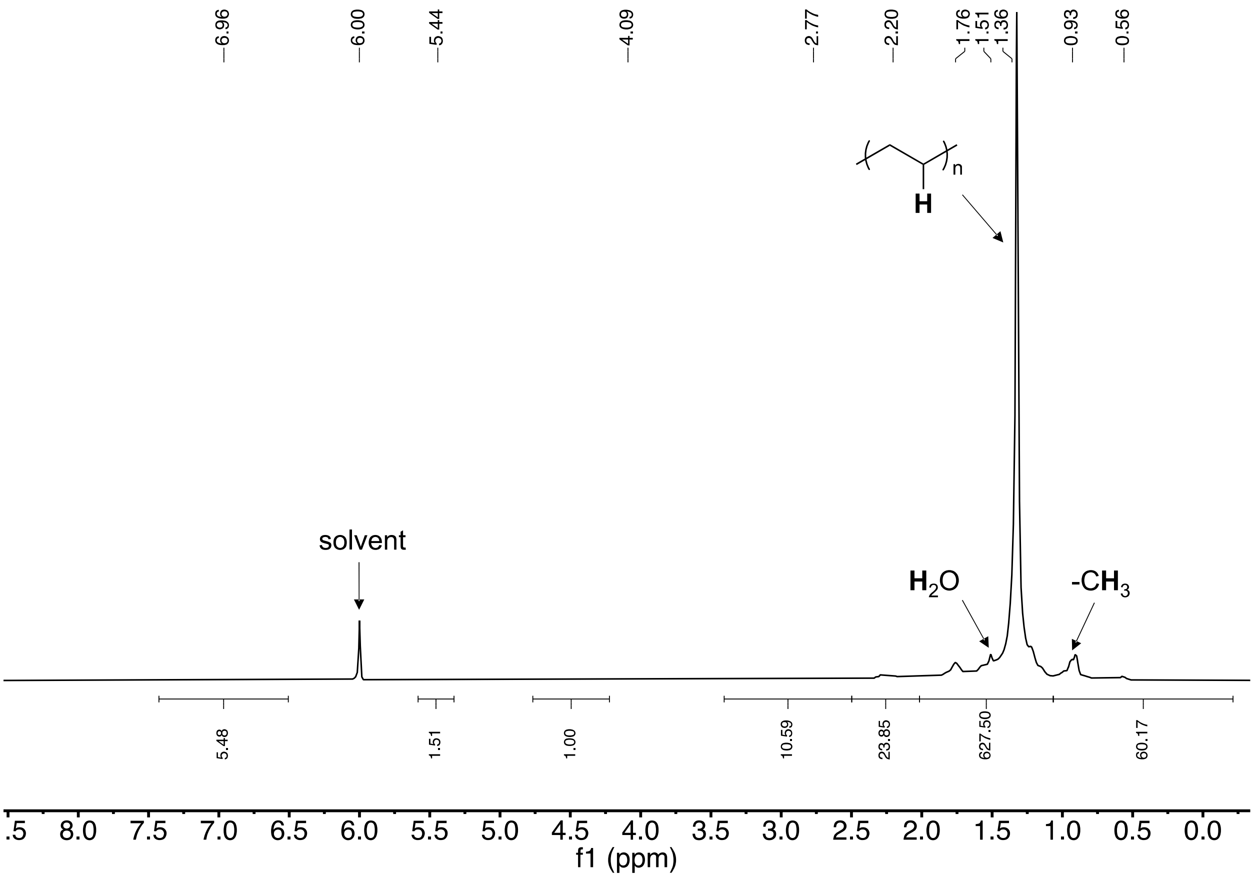

Fig. S58 ^{1}H NMR spectrum, in tetrachloroethane-d_2 (80 °C), of polyethylene product in Table 2, Entry 4 (soft PVC pipe).

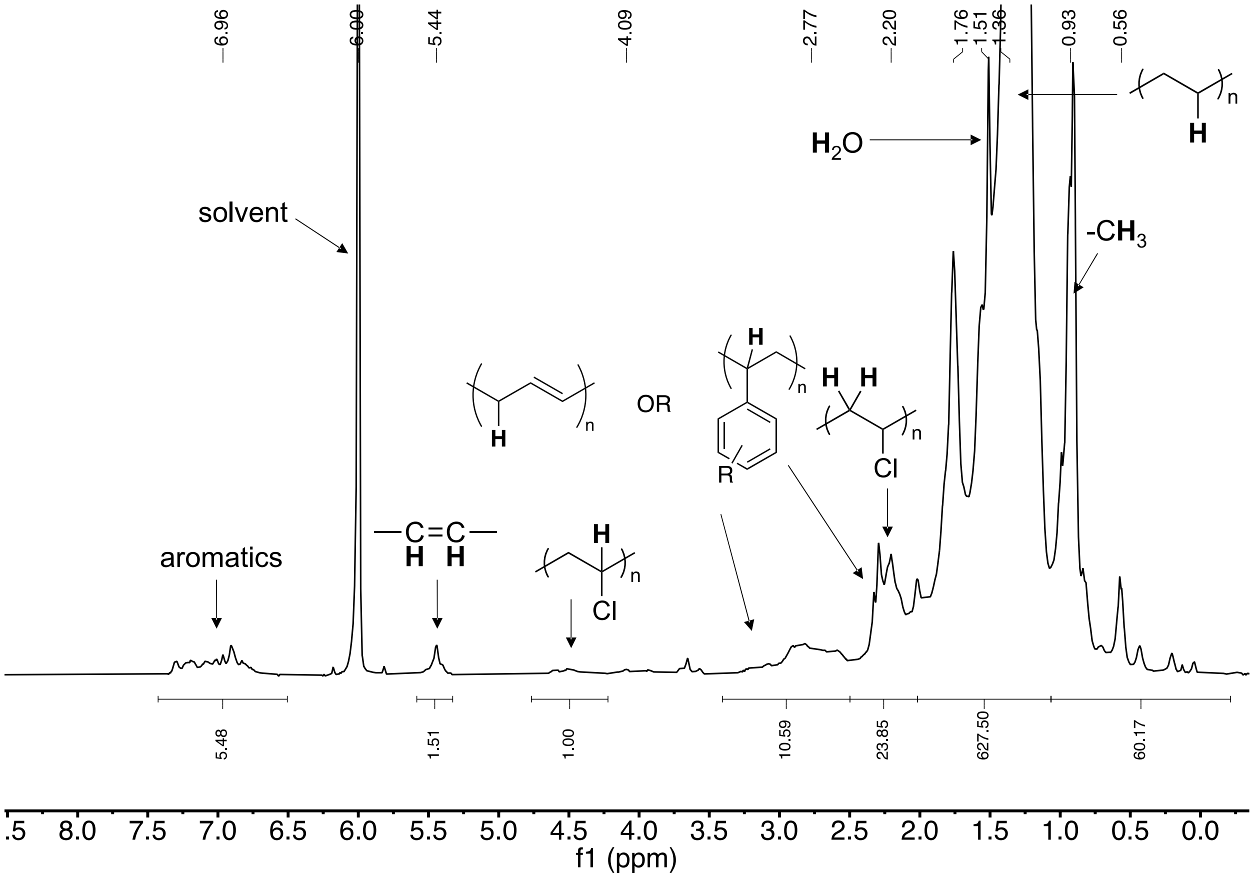

Fig. S58a Zoomed in ^{1}H NMR spectrum, in tetrachloroethane-d$_2$ (80 °C), of polyethylene product in Table 2, Entry 4 (soft PVC pipe).

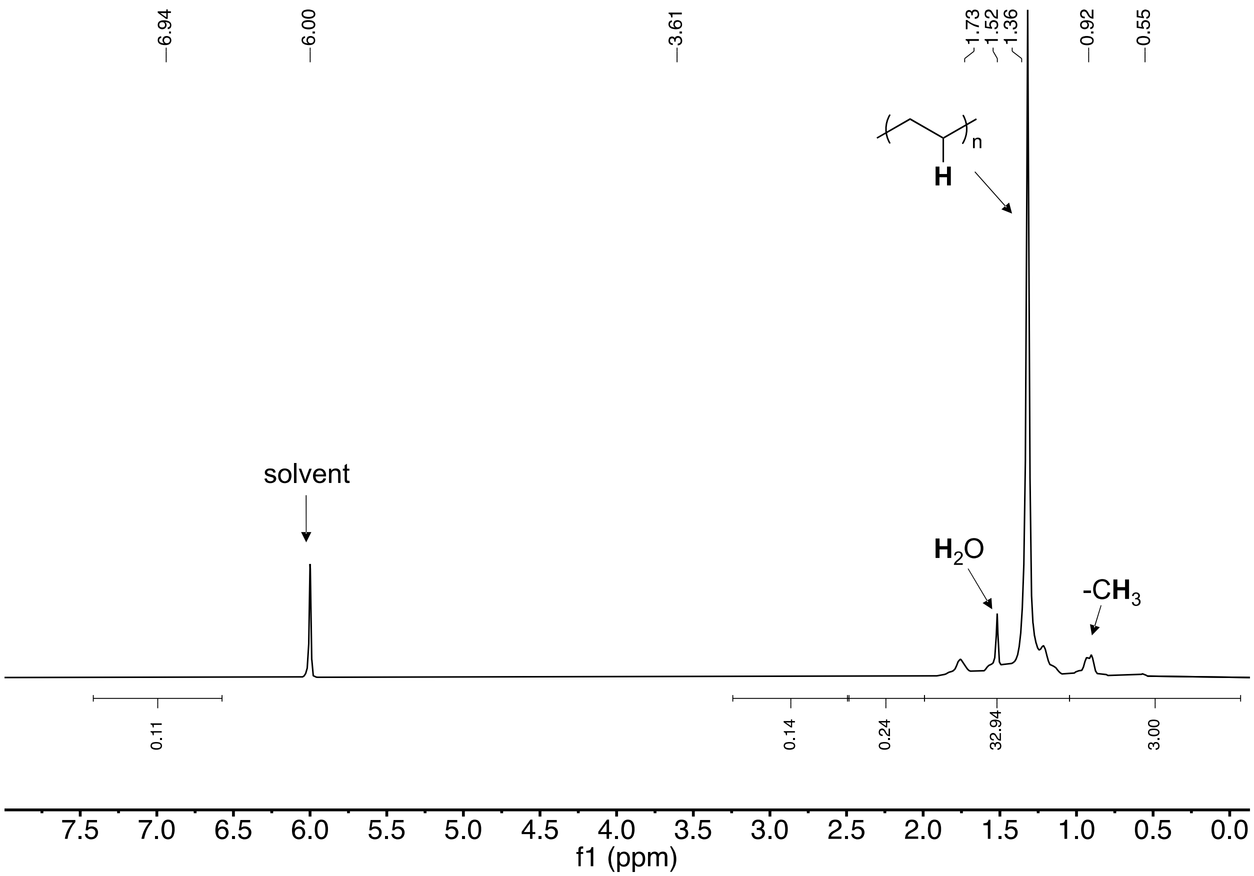

Fig. S59 ^{1}H NMR spectrum, in tetrachloroethane-d_2 (80 °C), of polyethylene product in Table 2, Entry 5 (vinyl record).

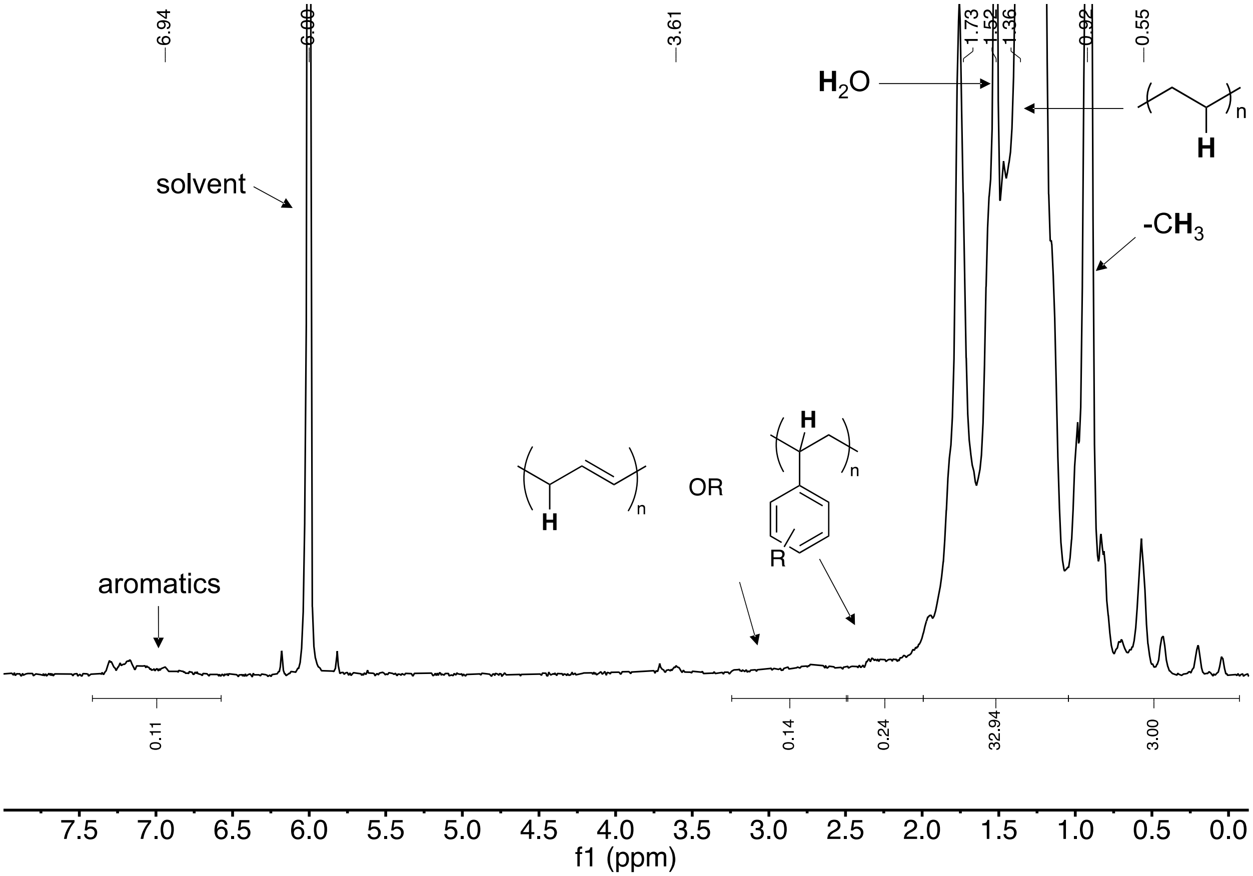

Fig. S59a Zoomed in ^{1}H NMR spectrum, in tetrachloroethane-d_2 (80 °C), of polyethylene product in Table 2, Entry 5 (vinyl record).

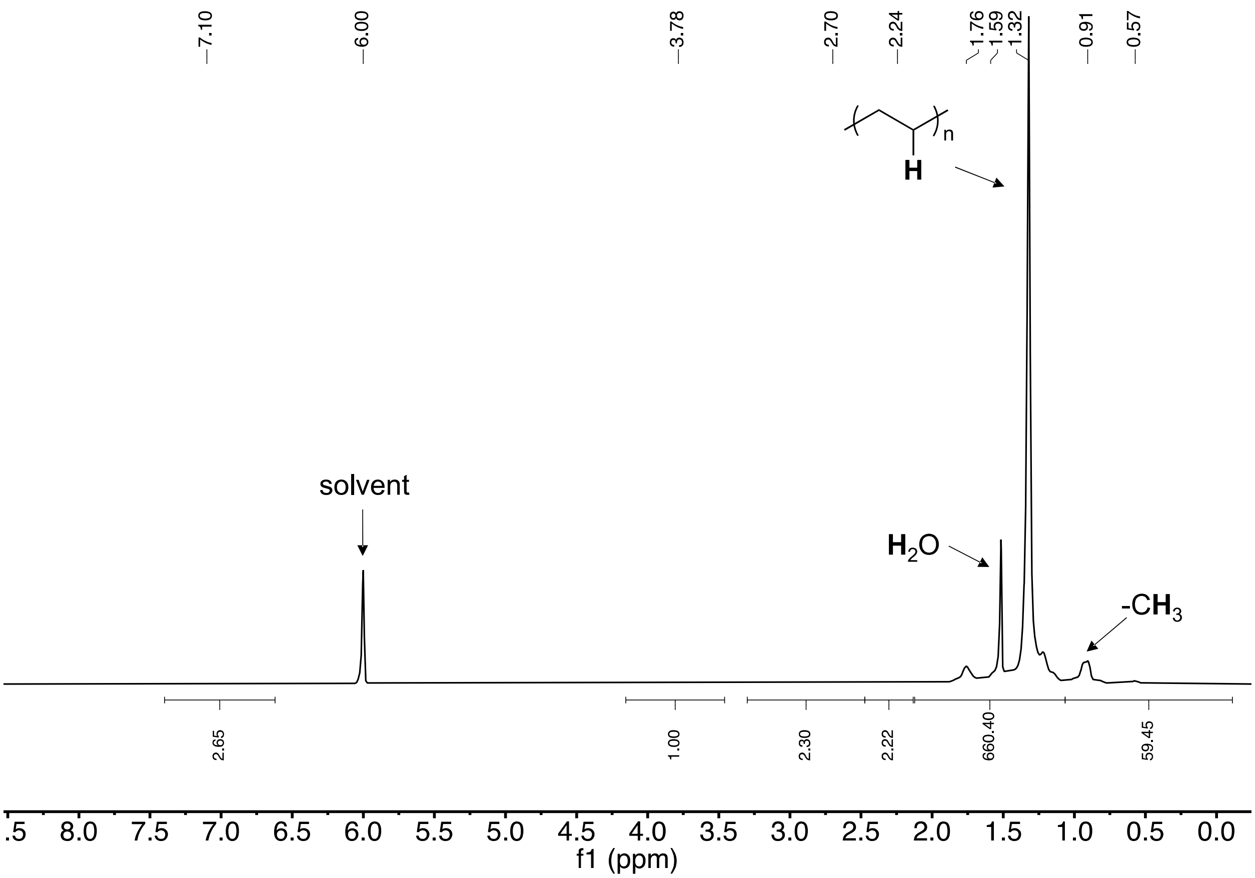

Fig. S60 ^{1}H NMR spectrum, in tetrachloroethane-d_2 (80 °C), of polyethylene product in Table 2, Entry 6 (extracted vinyl record).

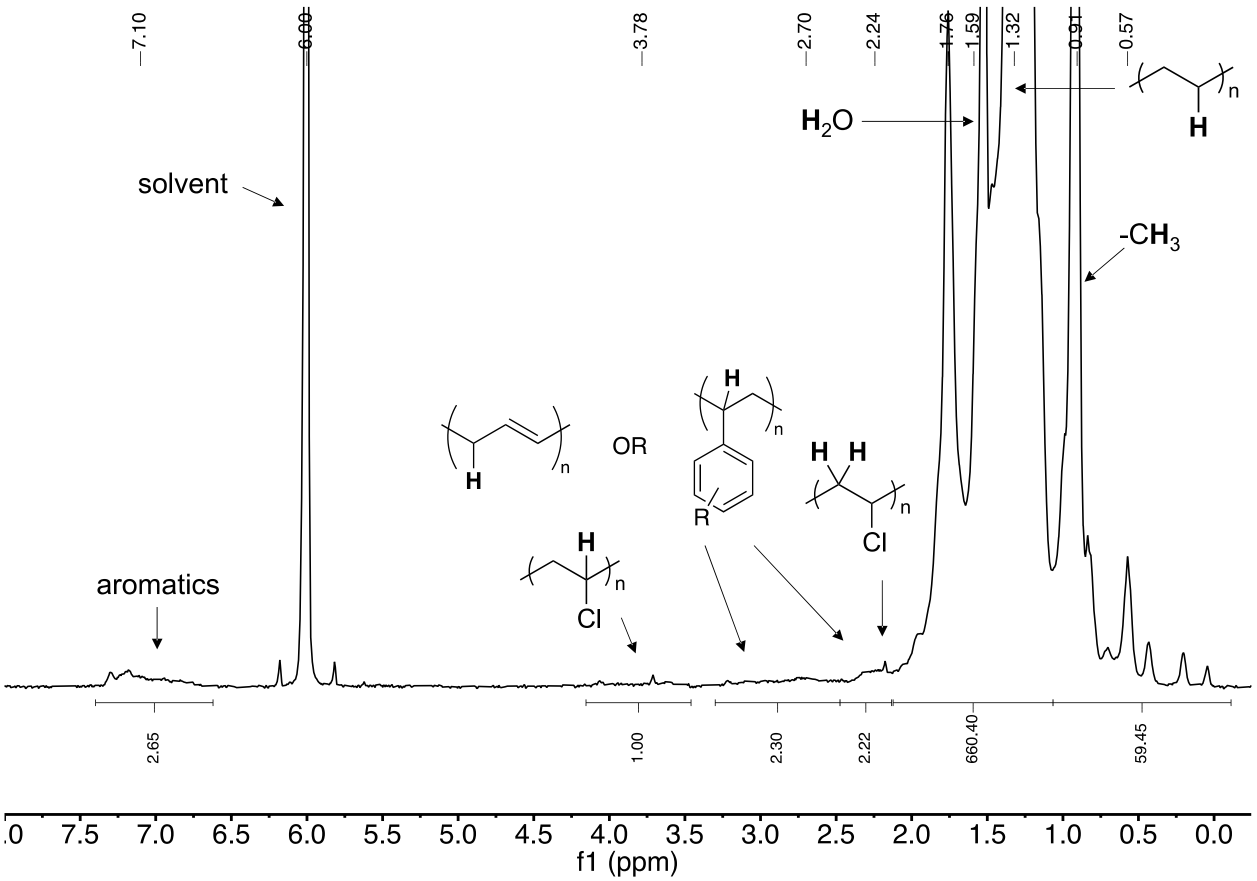

Fig. S60a Zoomed in ^{1}H NMR spectrum, in tetrachloroethane-d_2 (80 °C), of polyethylene product in Table 2, Entry 6 (extracted vinyl record).

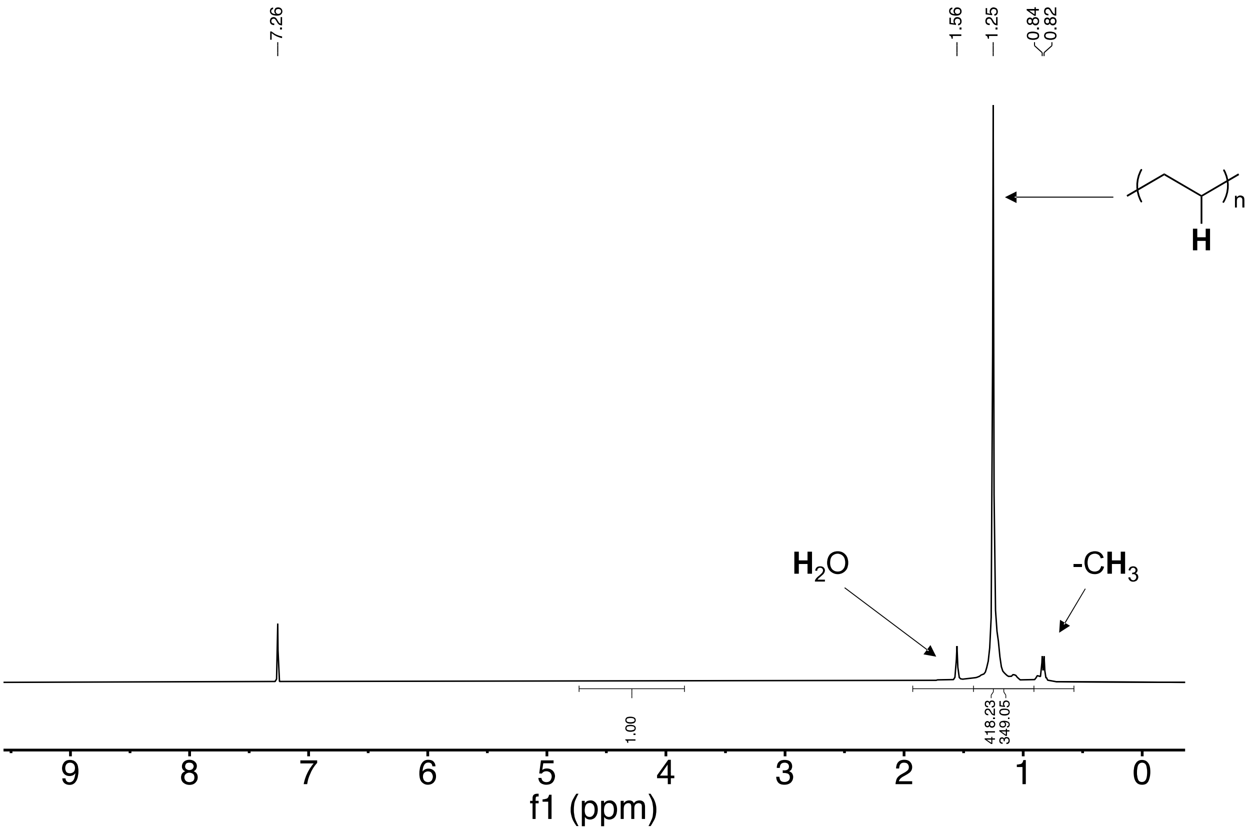

Fig. S61 ^{1}H NMR spectrum, in $CDCl_3$ (25 °C), of polyethylene product in Table S1, Entry 1.

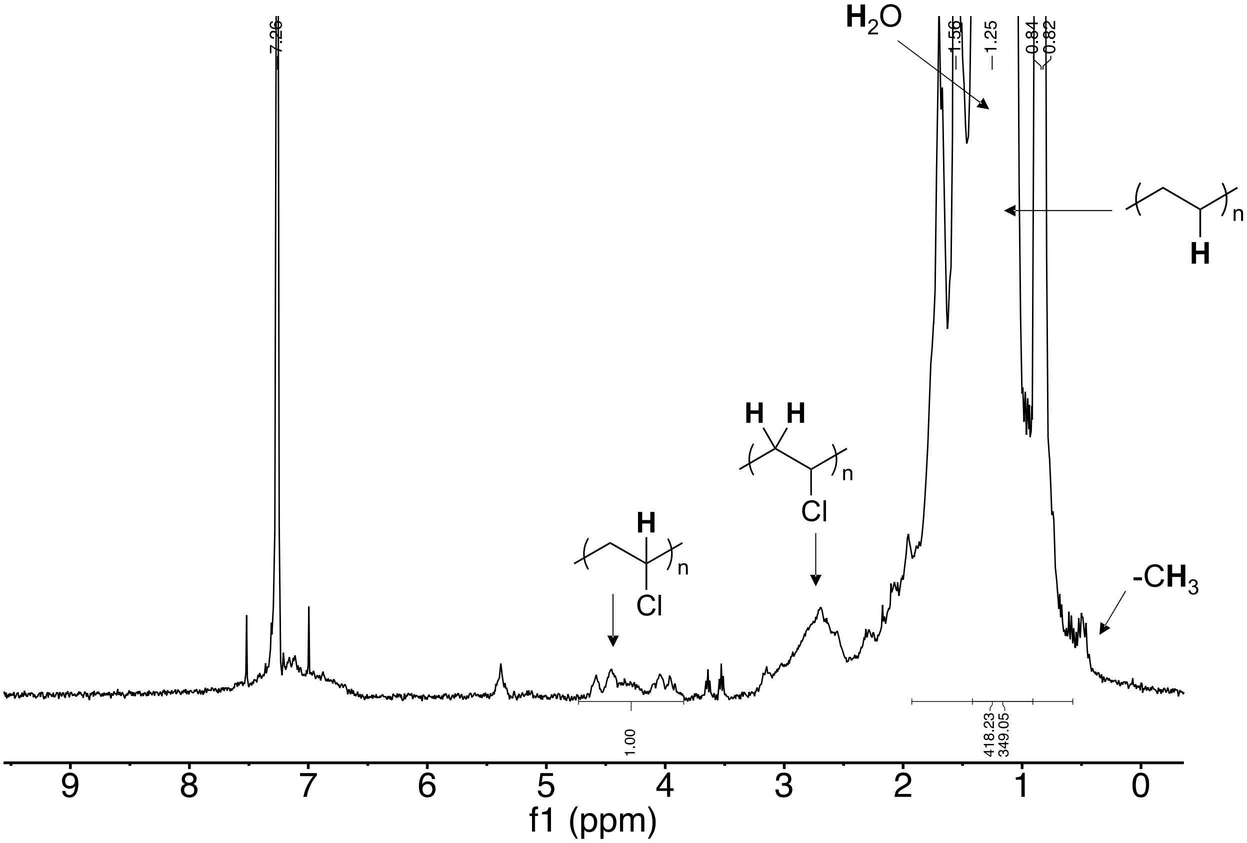

Fig. S61a Zoomed in ^{1}H NMR spectrum, in $CDCl_3$ (25 °C), of polyethylene product in Table S1, Entry 1.

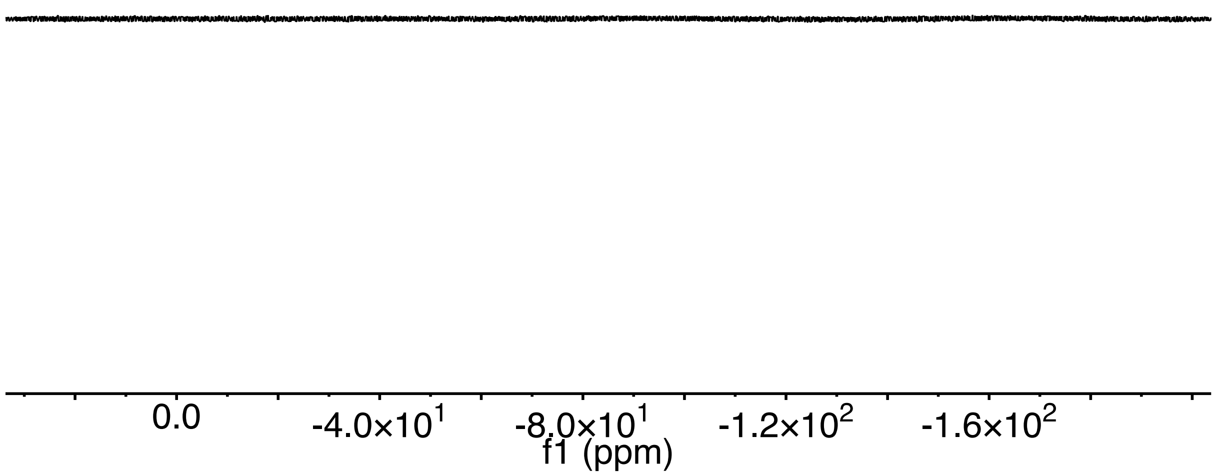

Fig. S61b $^{19}F\{^{1}H\}$ NMR spectrum, in $CDCl_3$ (25 °C), of polyethylene product in Table S1, Entry 1.

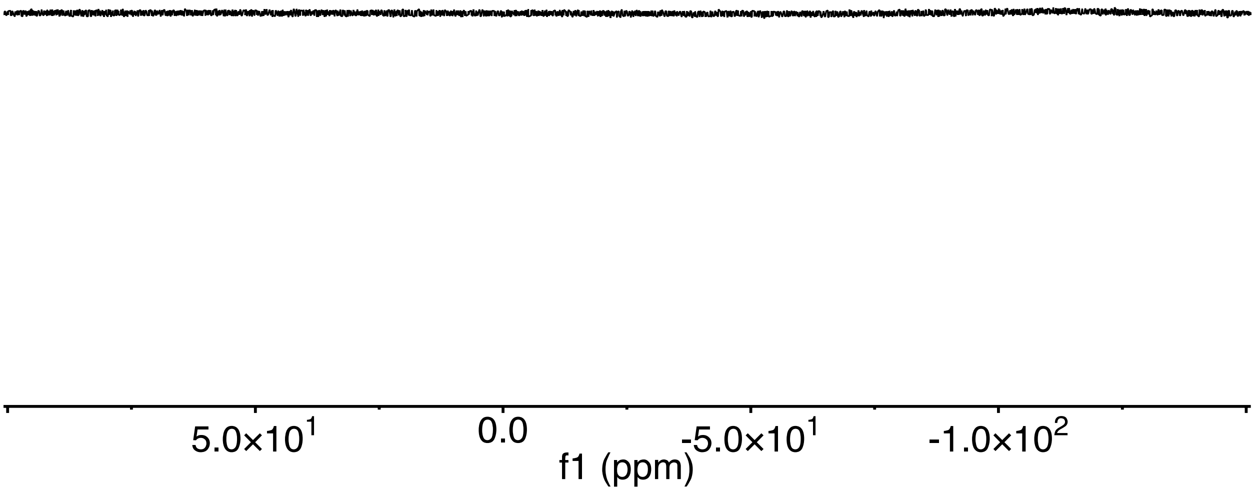

Fig. S61c ^{29}Si NMR spectrum, in $CDCl_3$ (25 °C), of polyethylene product in Table S1, Entry 1.

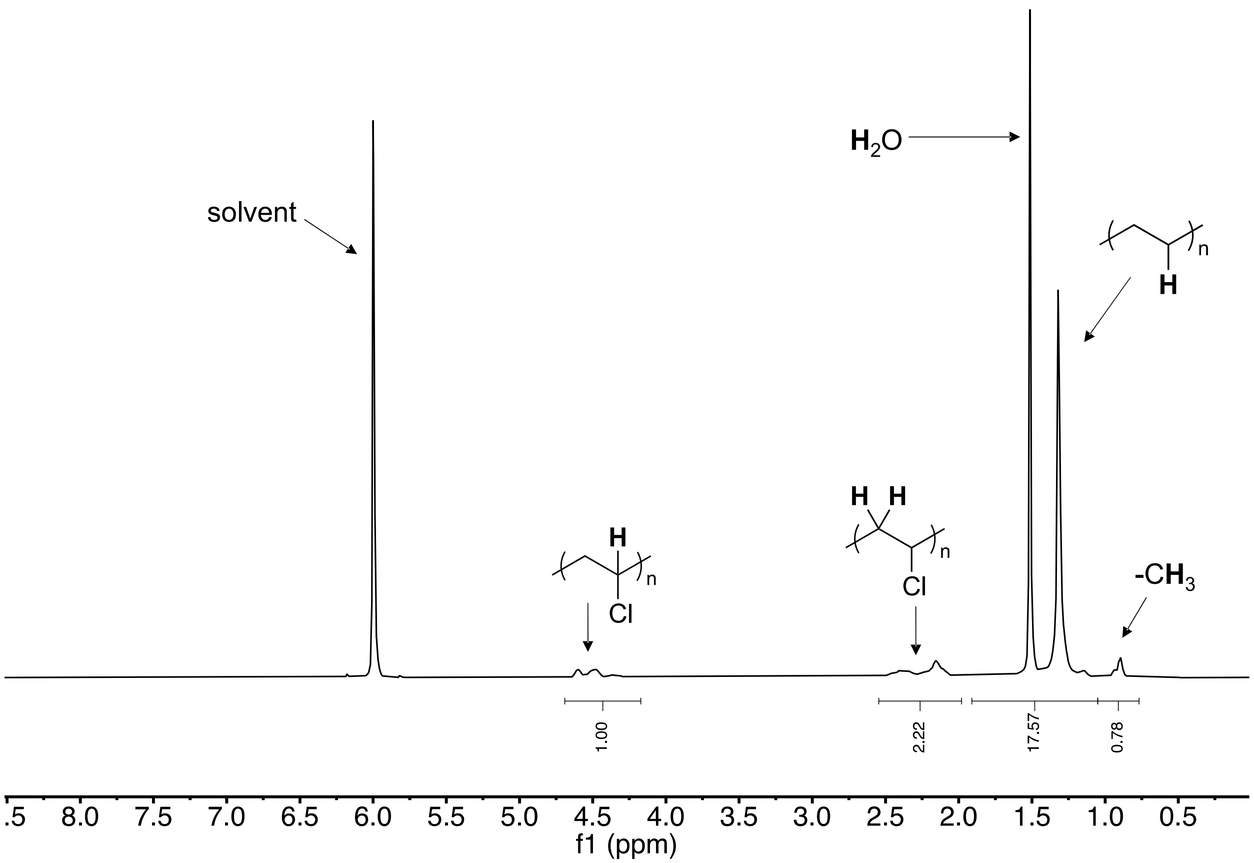

Fig. S62 ^{1}H NMR spectrum, in tetrachloroethane-d_2 (80 °C), of partially dechlorinated polyethylene product in Table S2, Entry 1.

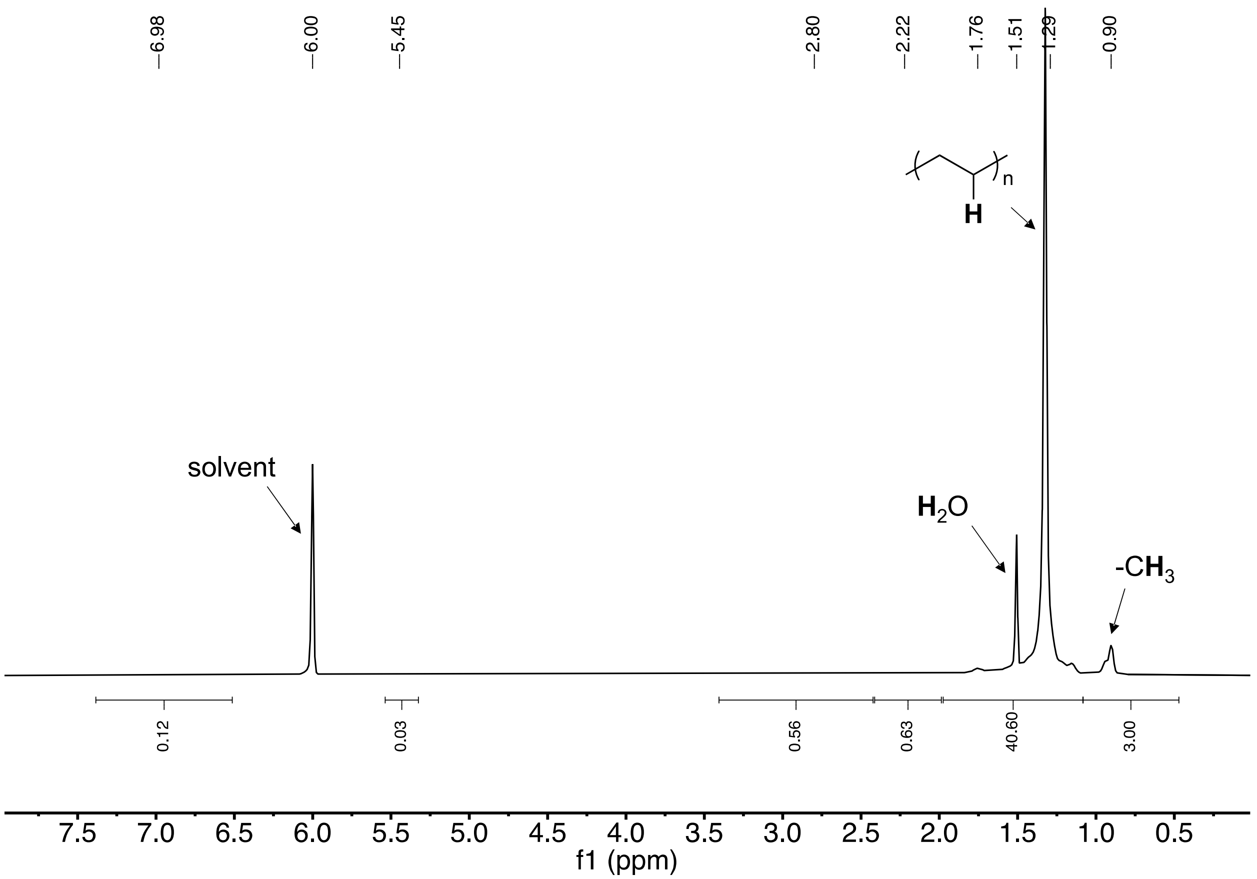

Fig. S63 ^{1}H NMR spectrum, in tetrachloroethane-d_2 (80 °C), of polyethylene product in Table S3, Entry 1.

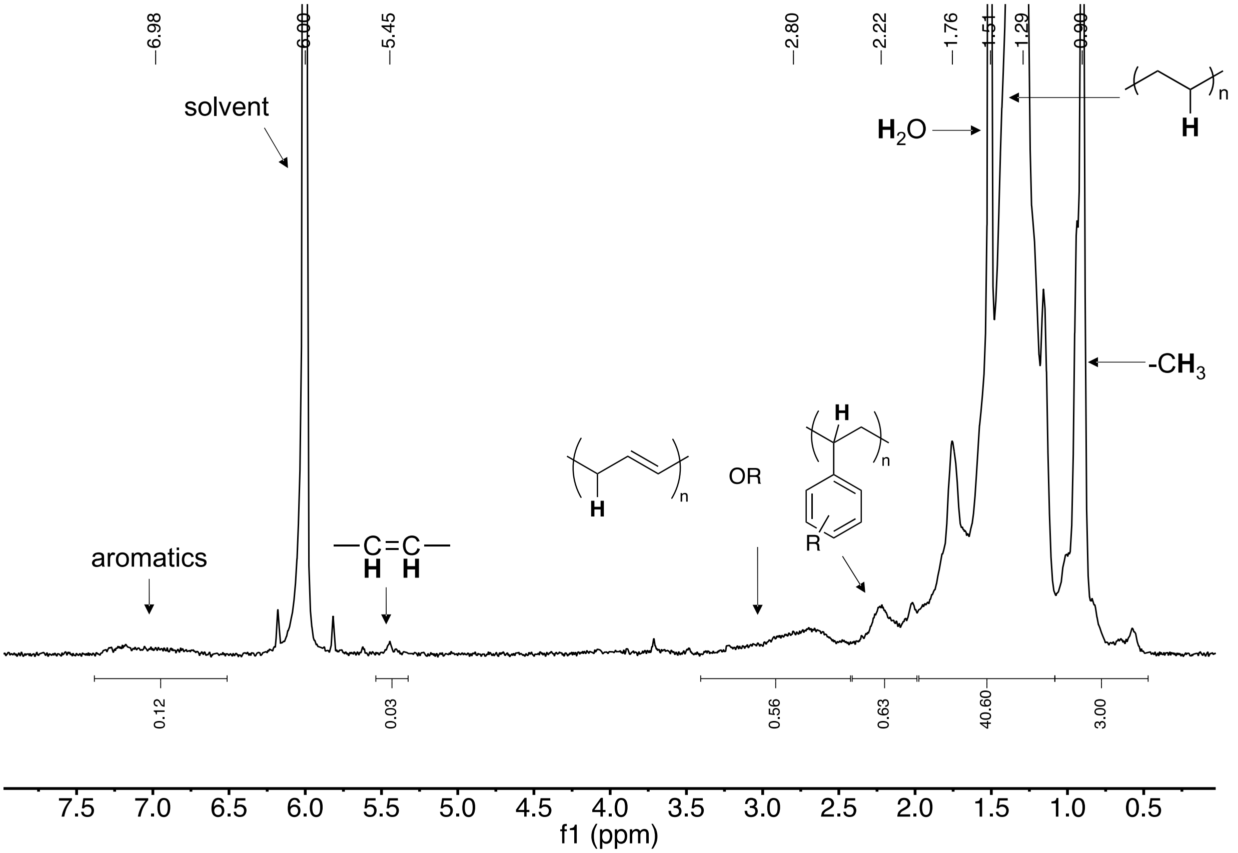

Fig. S63a Zoomed in ^{1}H NMR spectrum, in tetrachloroethane-d_2 (80 °C), of polyethylene product in Table S3, Entry 1.

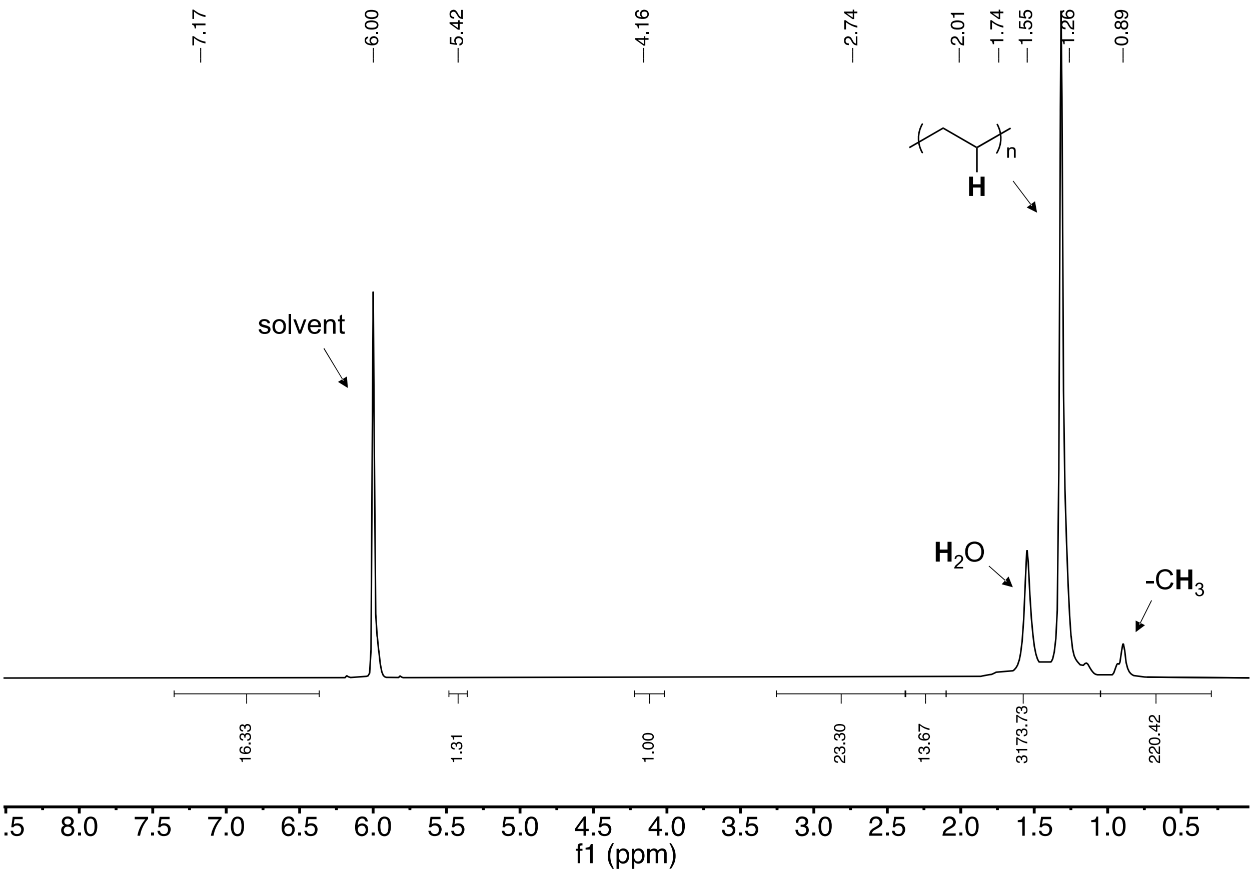

Fig. S64 ^{1}H NMR spectrum, in tetrachloroethane-d_2 (80 °C), of polyethylene product in Table S3, Entry 2.

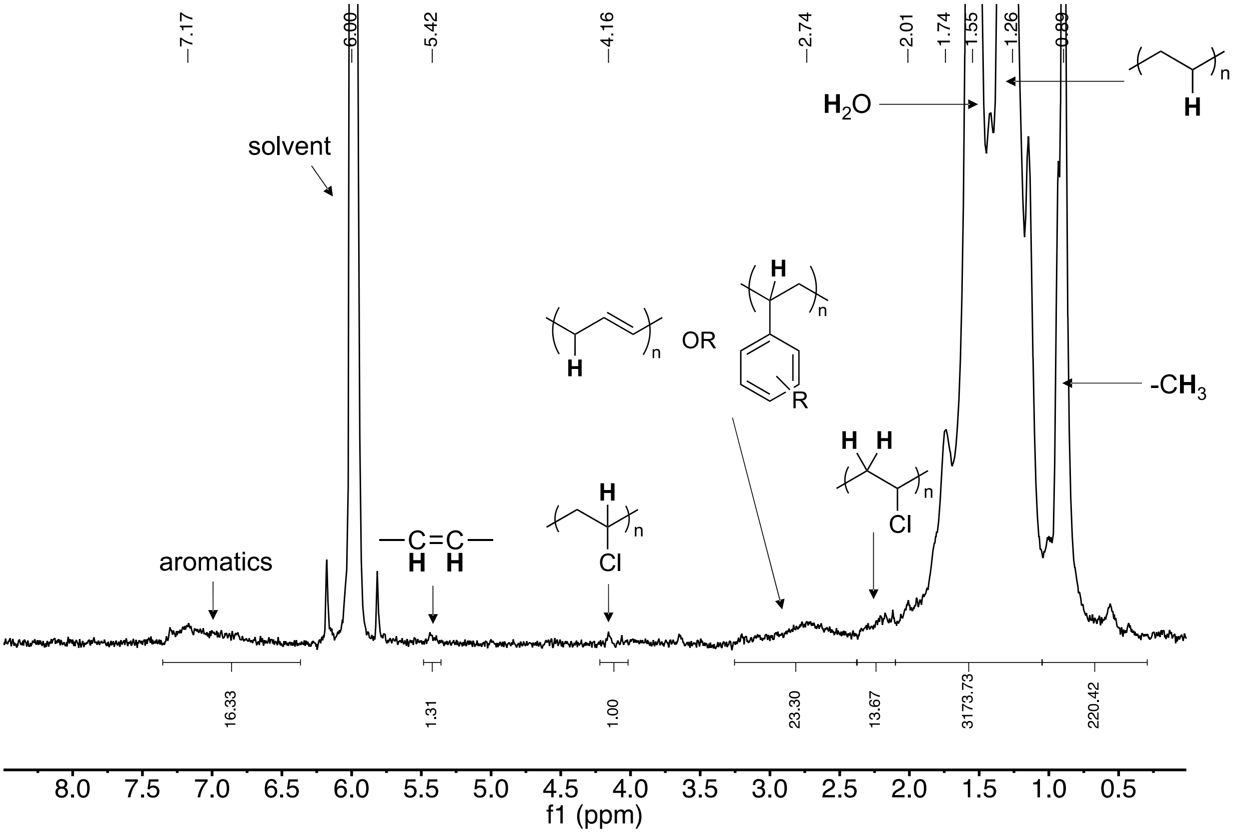

Fig. S64a Zoomed in ^{1}H NMR spectrum, in tetrachloroethane-d_2 (80 °C), of polyethylene product in Table S3, Entry 2.

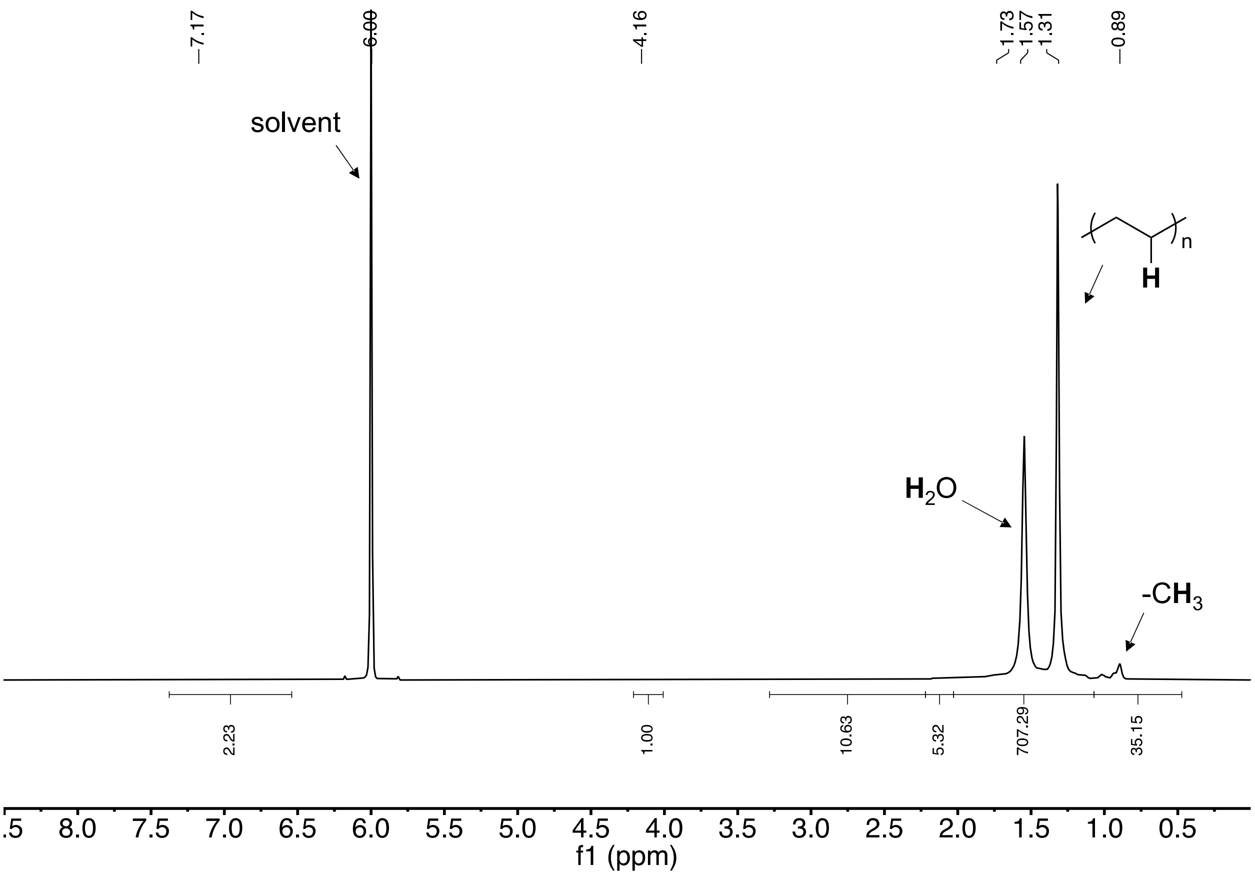

Fig. S65 ^{1}H NMR spectrum, in tetrachloroethane-d_2 (80 °C), of polyethylene product in Table S3, Entry 3.

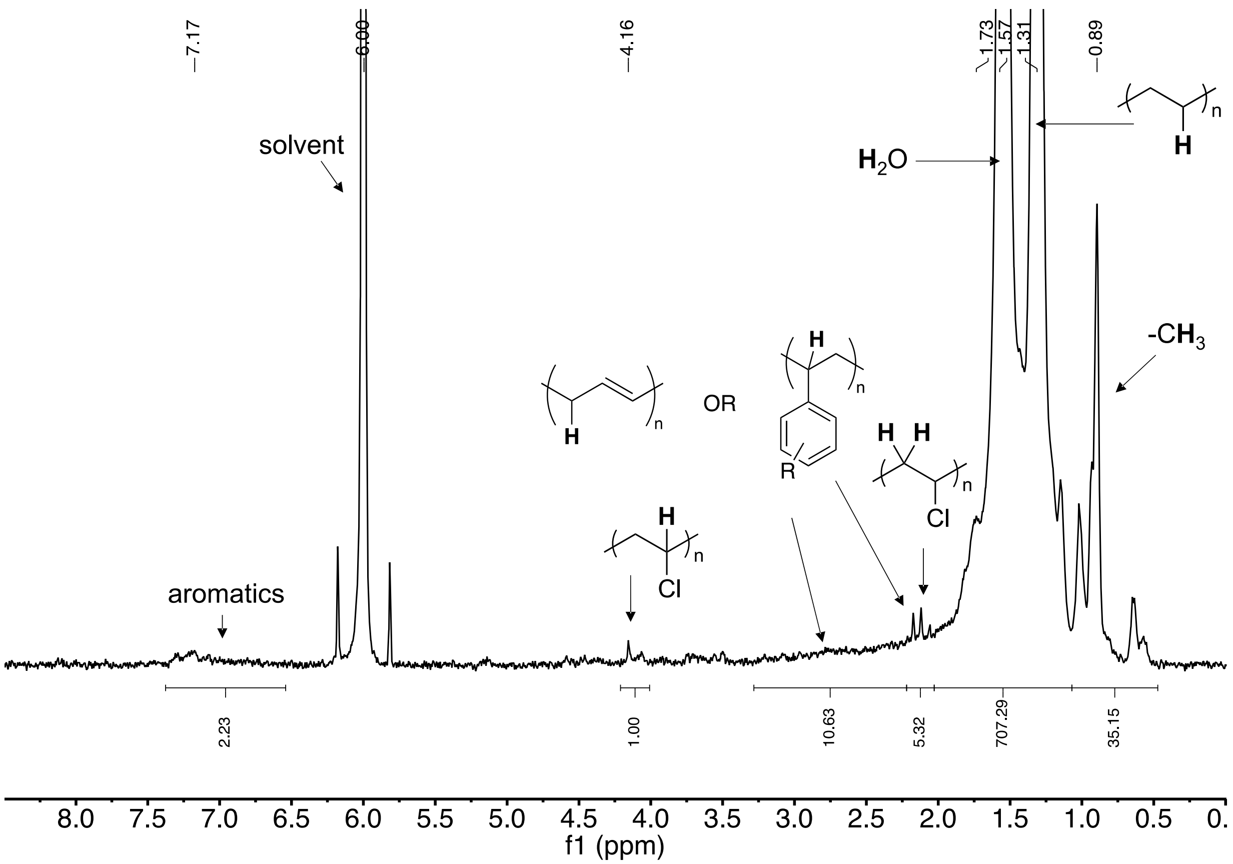

Fig. S65a Zoomed in ^{1}H NMR spectrum, in tetrachloroethane-d_2 (80 °C), of polyethylene product in Table S3, Entry 3.

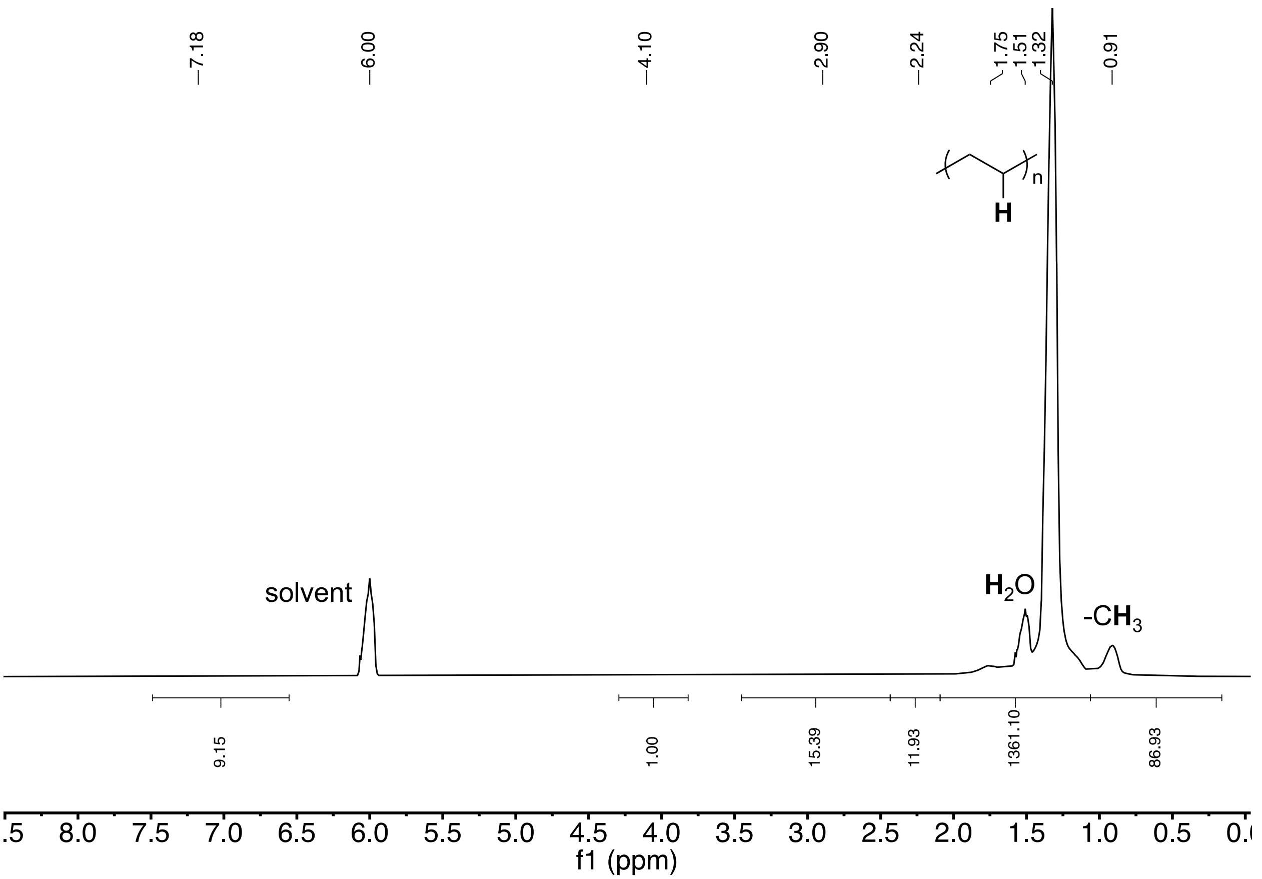

Fig. S66 ^{1}H NMR spectrum, in tetrachloroethane-d$_2$ (80 °C), of polyethylene product in Table S3, Entry 4.

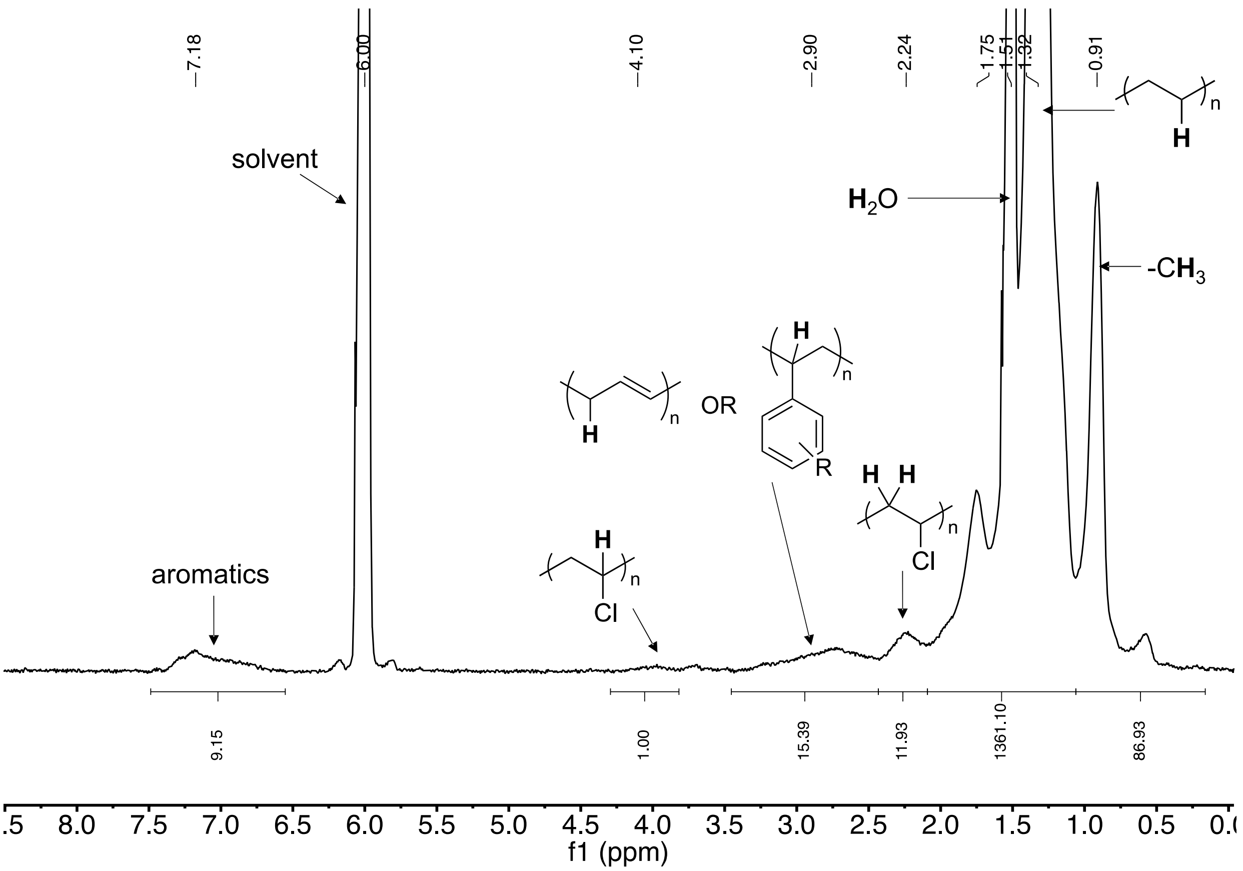

Fig. S66a Zoomed in ^{1}H NMR spectrum, in tetrachloroethane-d_2 (80 °C), of polyethylene product in Table S3, Entry 4.

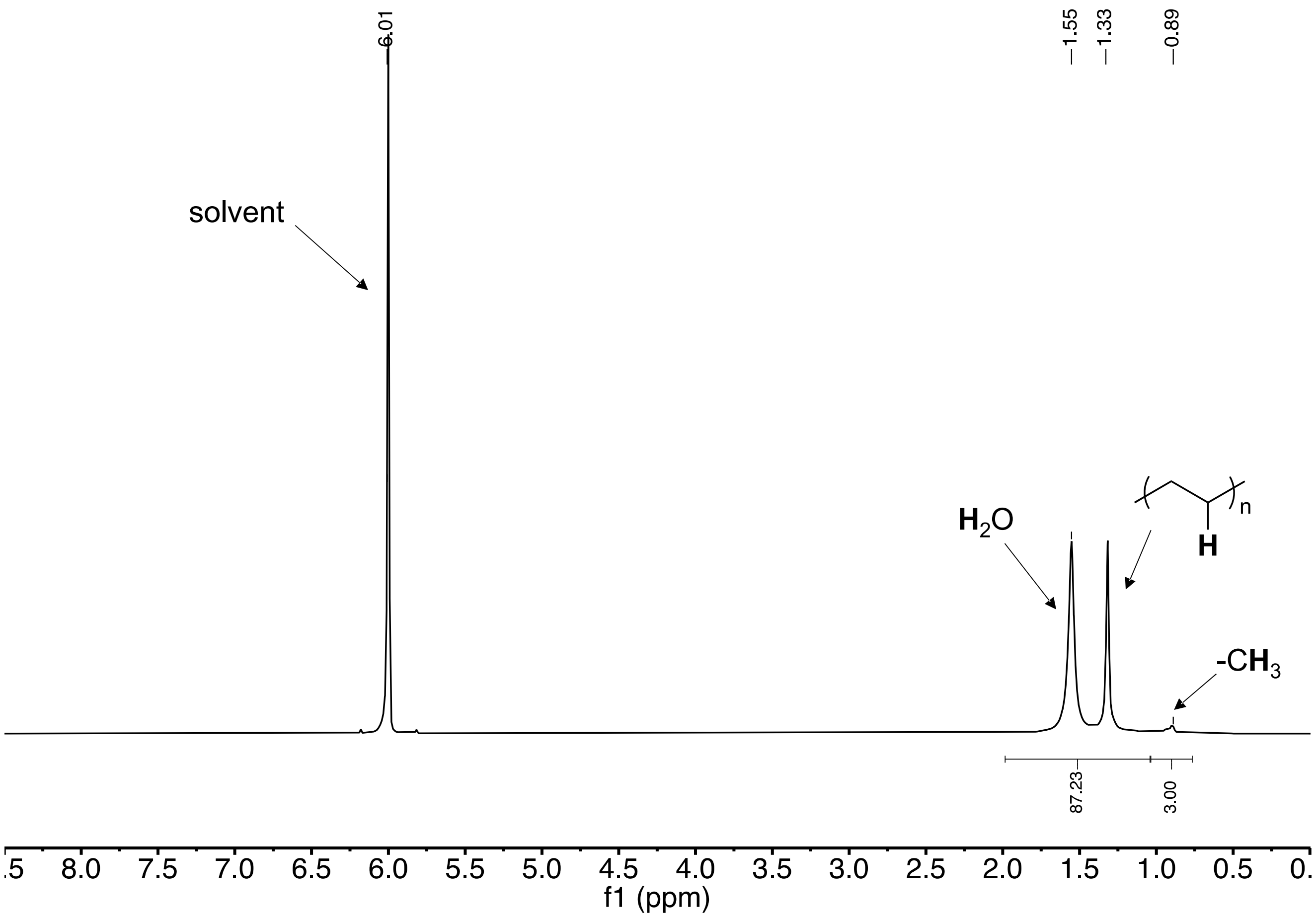

Fig. S67 ^{1}H NMR spectrum, in tetrachloroethane-d_2 (80 °C), of polyethylene product in Table S3, Entry 5a.

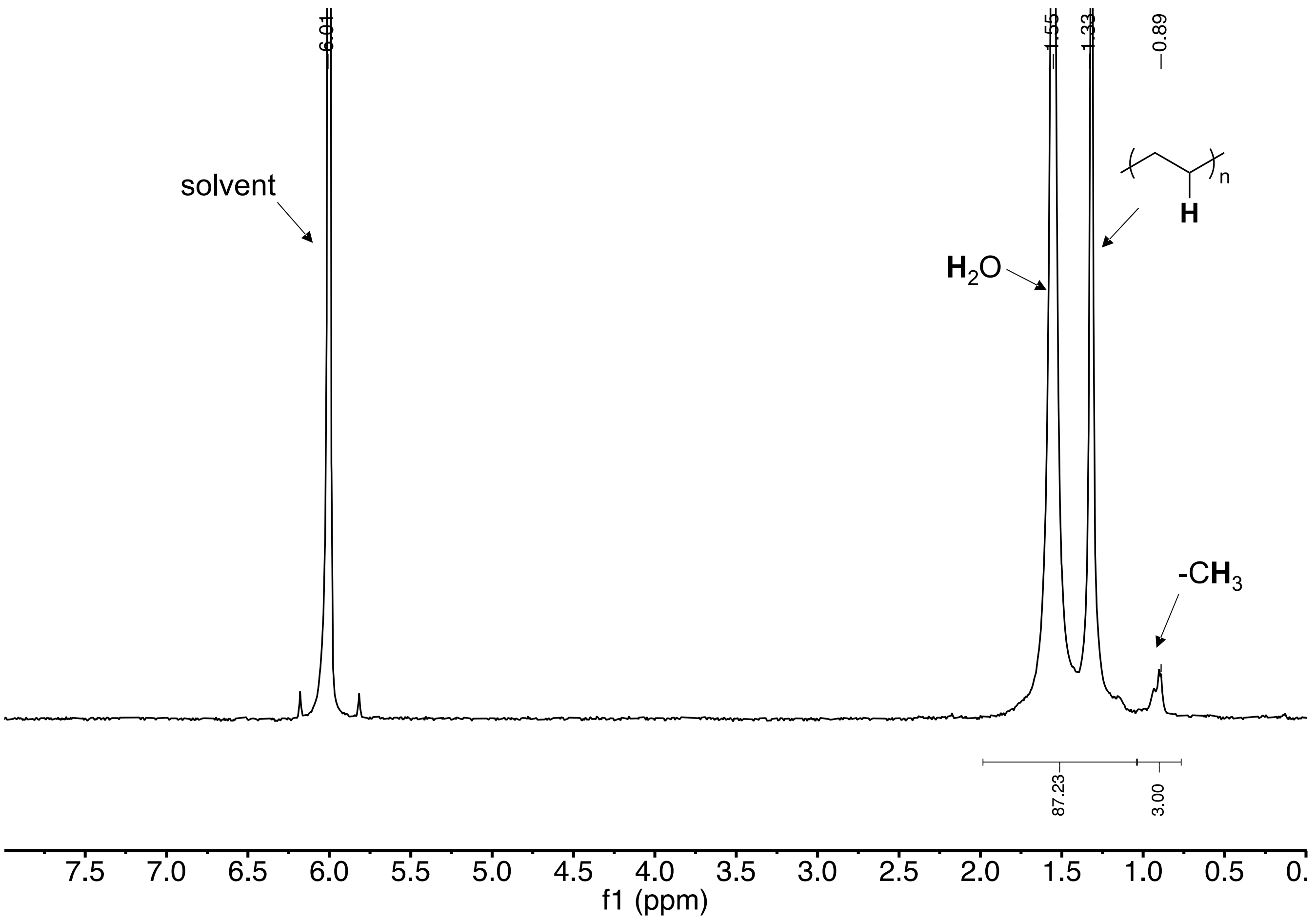

Fig. S67a Zoomed in ^{1}H NMR spectrum, in tetrachloroethane-d_2 (80 °C), of polyethylene product in Table S3, Entry 5a.

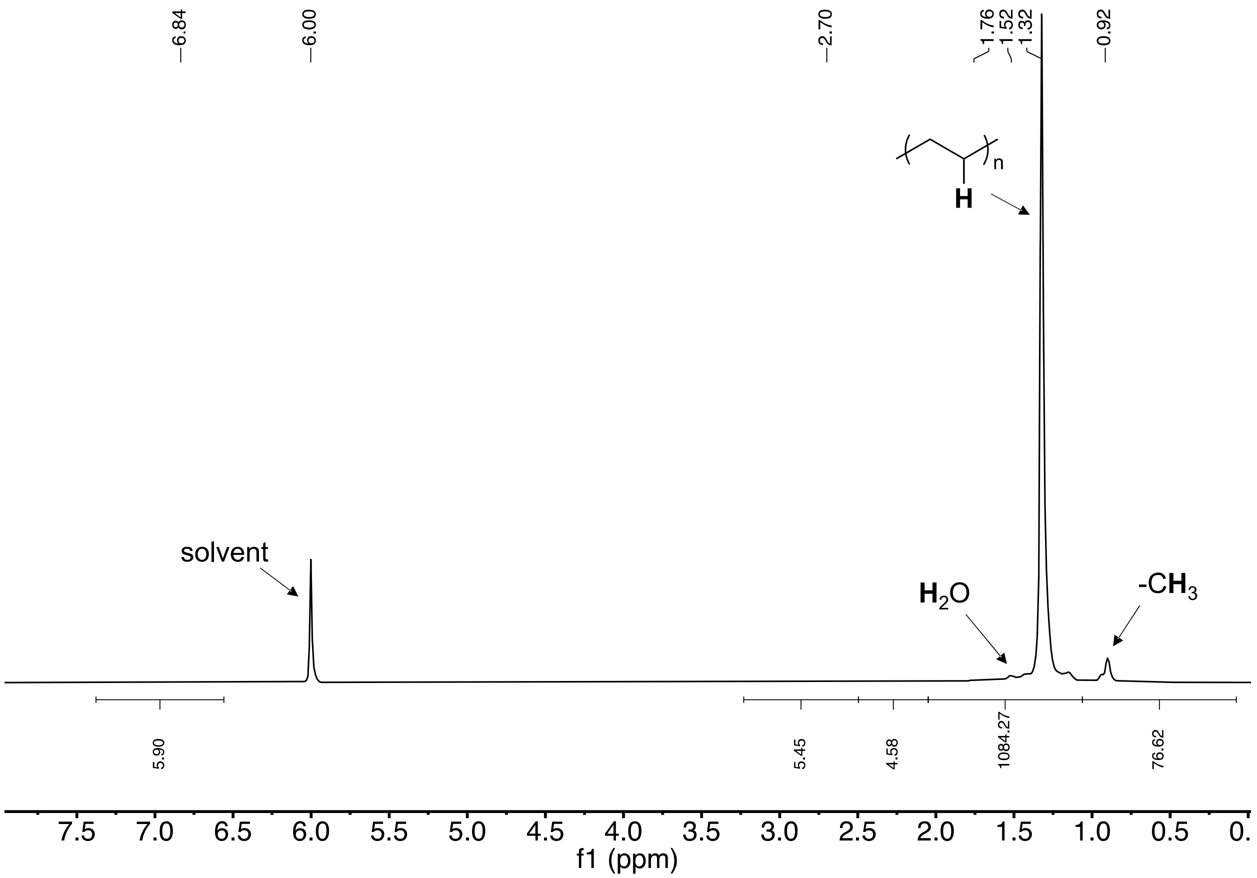

Fig. S68 ^1H NMR spectrum, in tetrachloroethane-d_2 (80 °C), of polyethylene product in Table S3, Entry 5b.

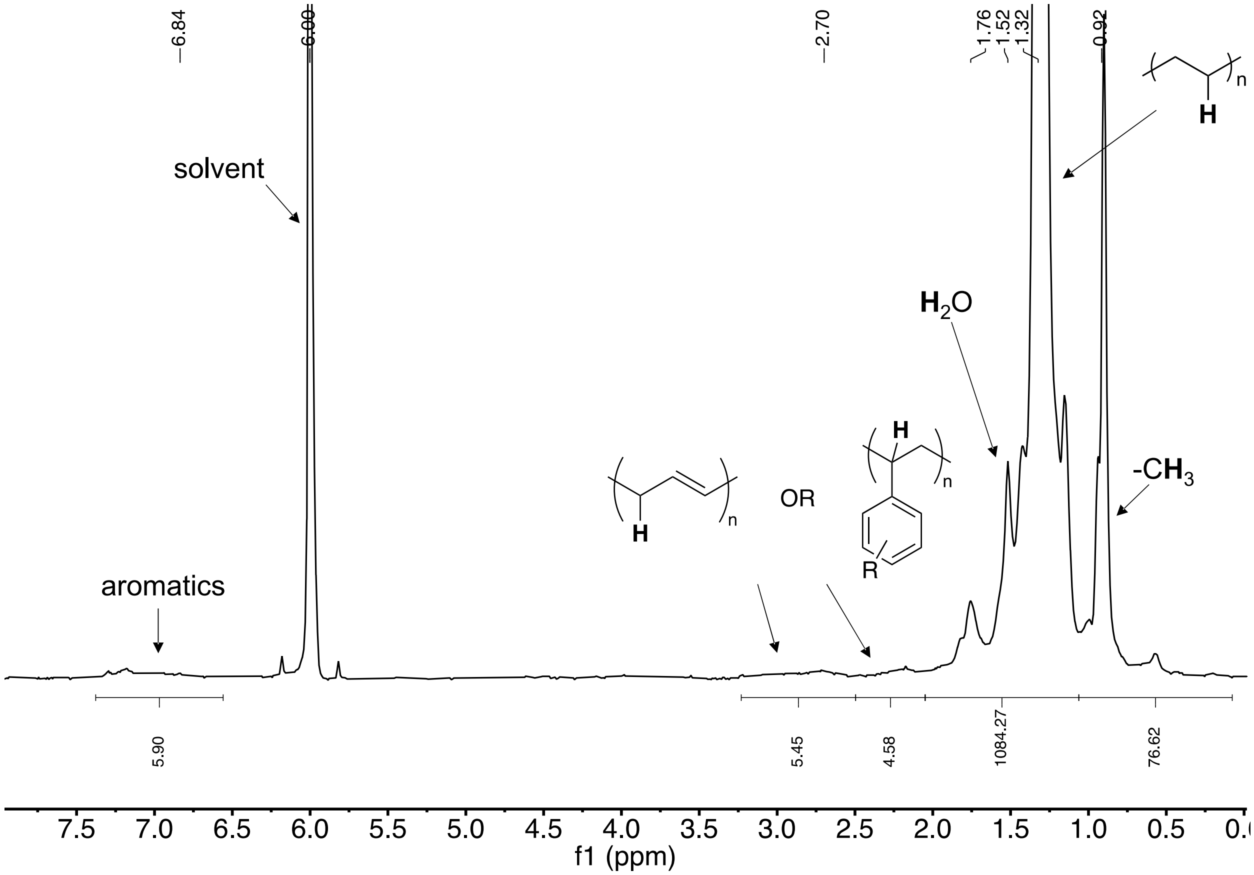

Fig. S68a Zoomed in ^{1}H NMR spectrum, in tetrachloroethane-d_2 (80 °C), of polyethylene product in Table S3, Entry 5b.

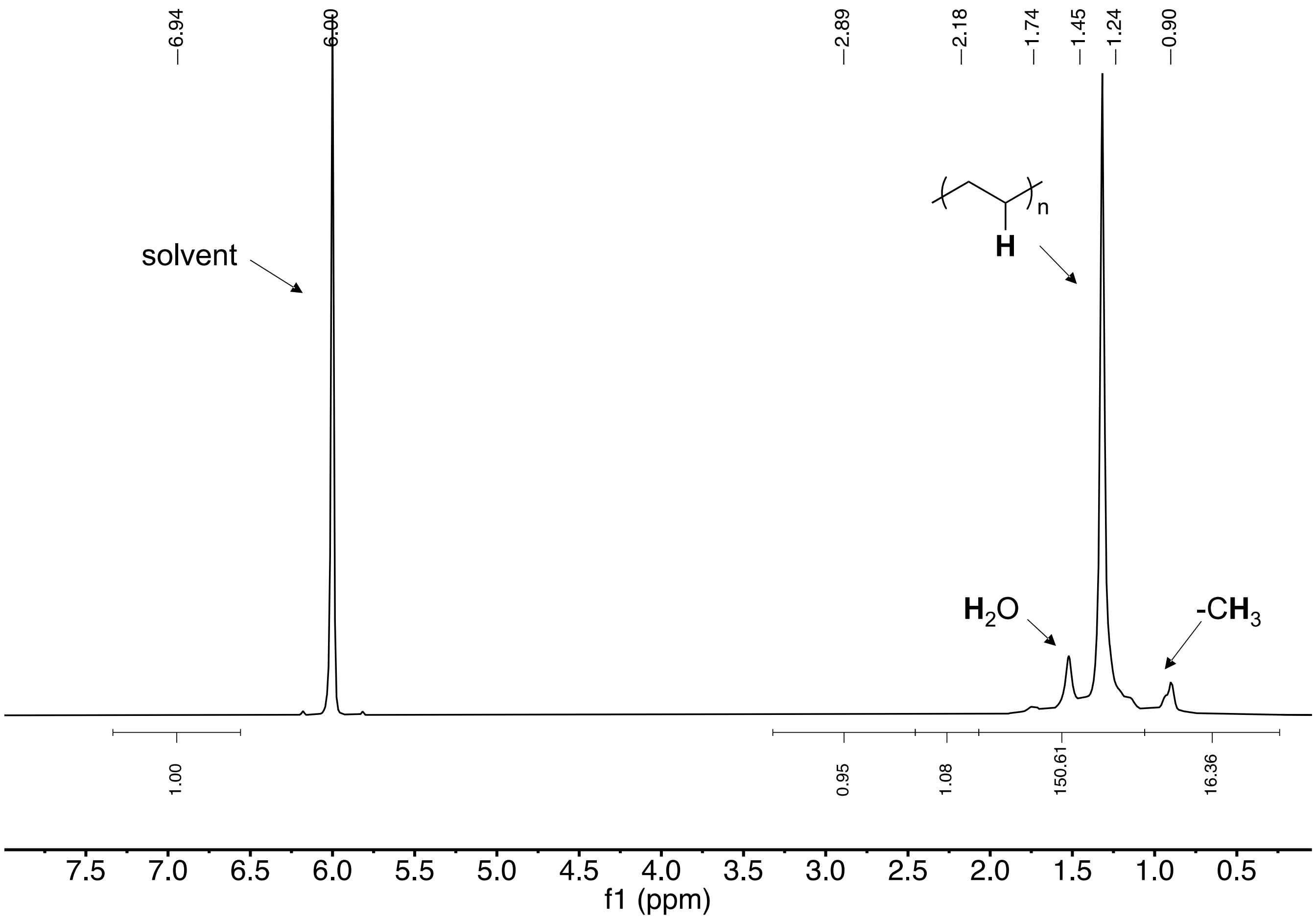

Fig. S69 ^{1}H NMR spectrum, in tetrachloroethane-d_2 (80 °C), of polyethylene product in Table S4, Entry 1a.

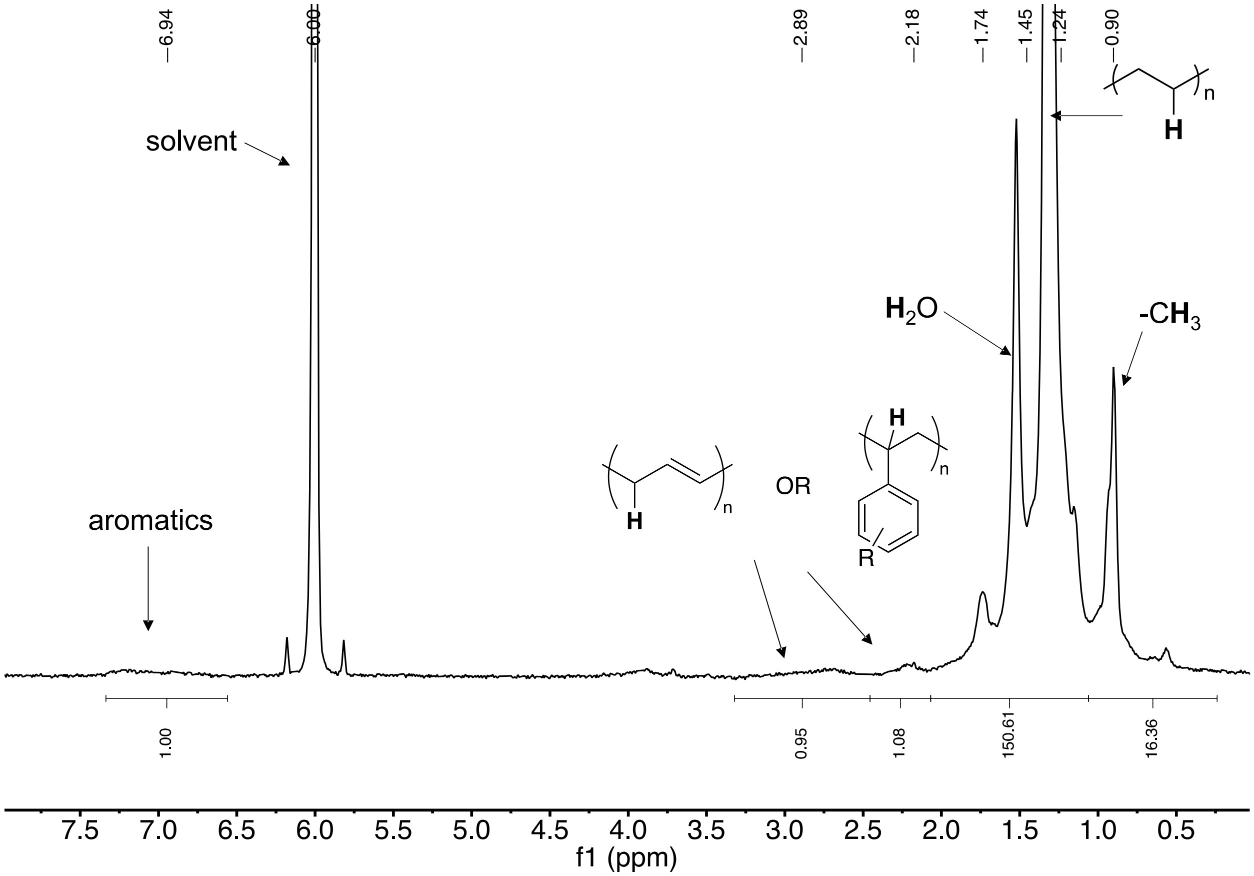

Fig. S69a Zoomed in ^{1}H NMR spectrum, in tetrachloroethane-d_2 (80 °C), of polyethylene product in Table S4, Entry 1a.

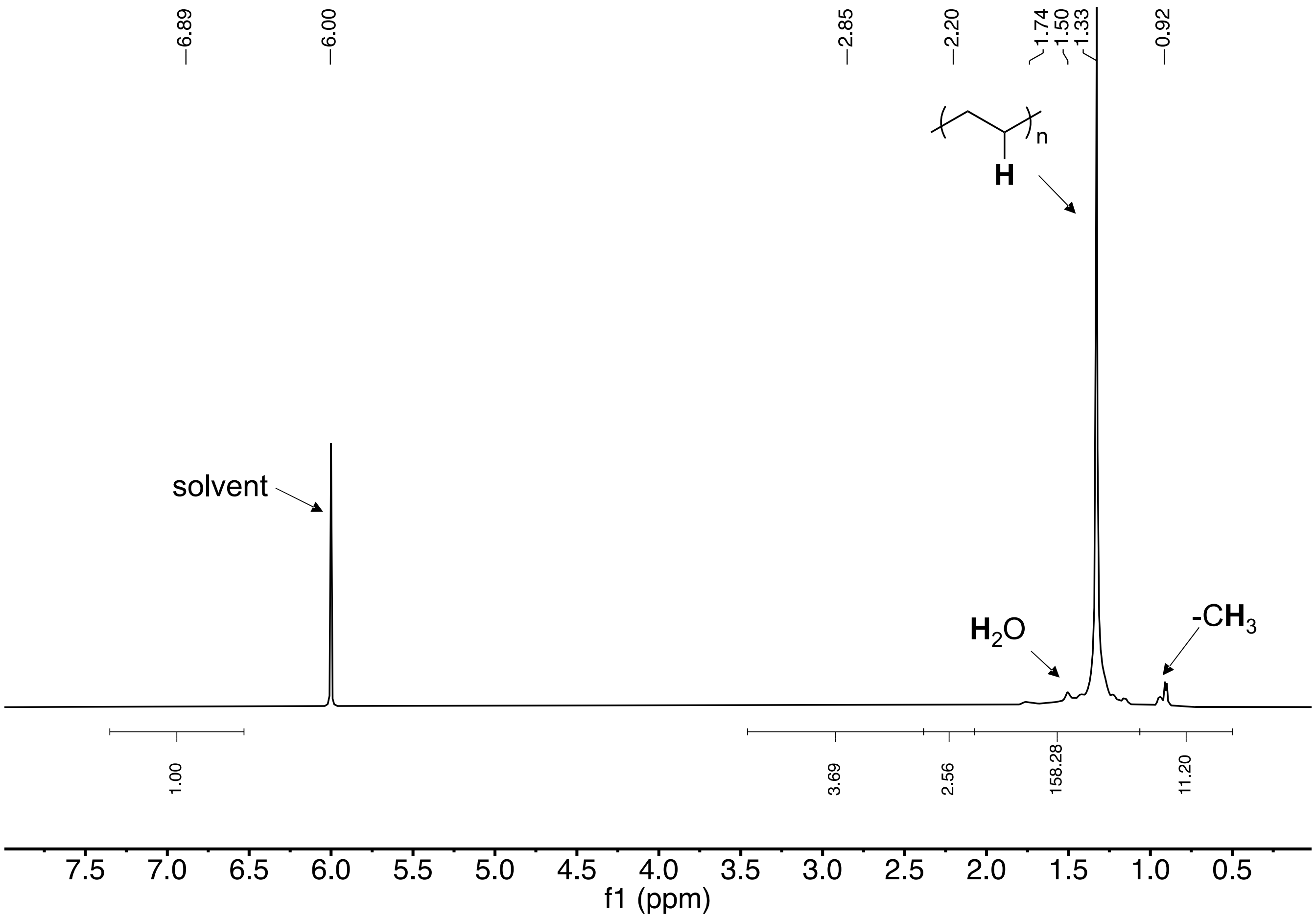

Fig. S70 ^{1}H NMR spectrum, in tetrachloroethane-d$_2$ (80 °C), of polyethylene product in Table S4, Entry 1b.

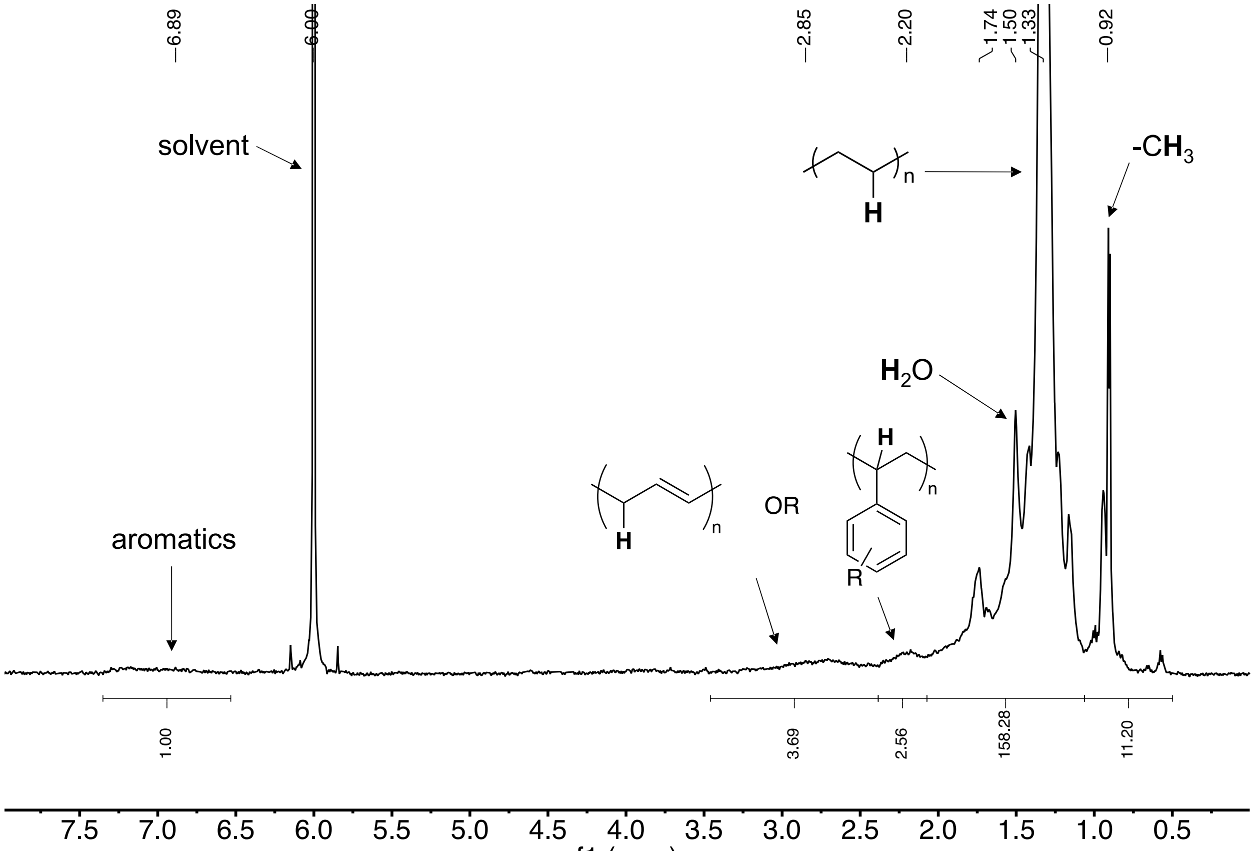

Fig. S70a Zoomed in ^{1}H NMR spectrum, in tetrachloroethane-d$_2$ (80 °C), of polyethylene product in Table S4, Entry 1b.

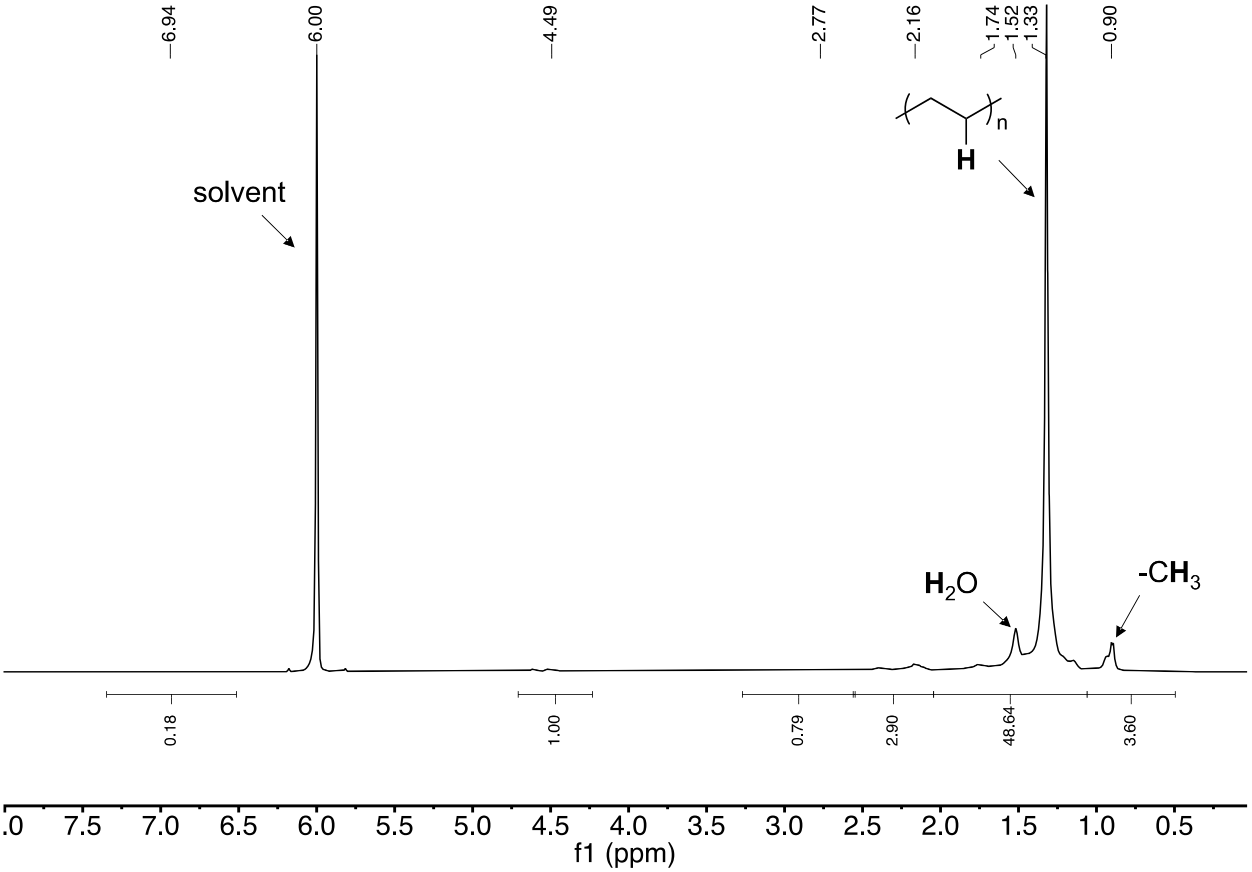

Fig. S71 ^{1}H NMR spectrum, in tetrachloroethane-d_2 (80 °C), of polyethylene product in Table S4, Entry 1c.

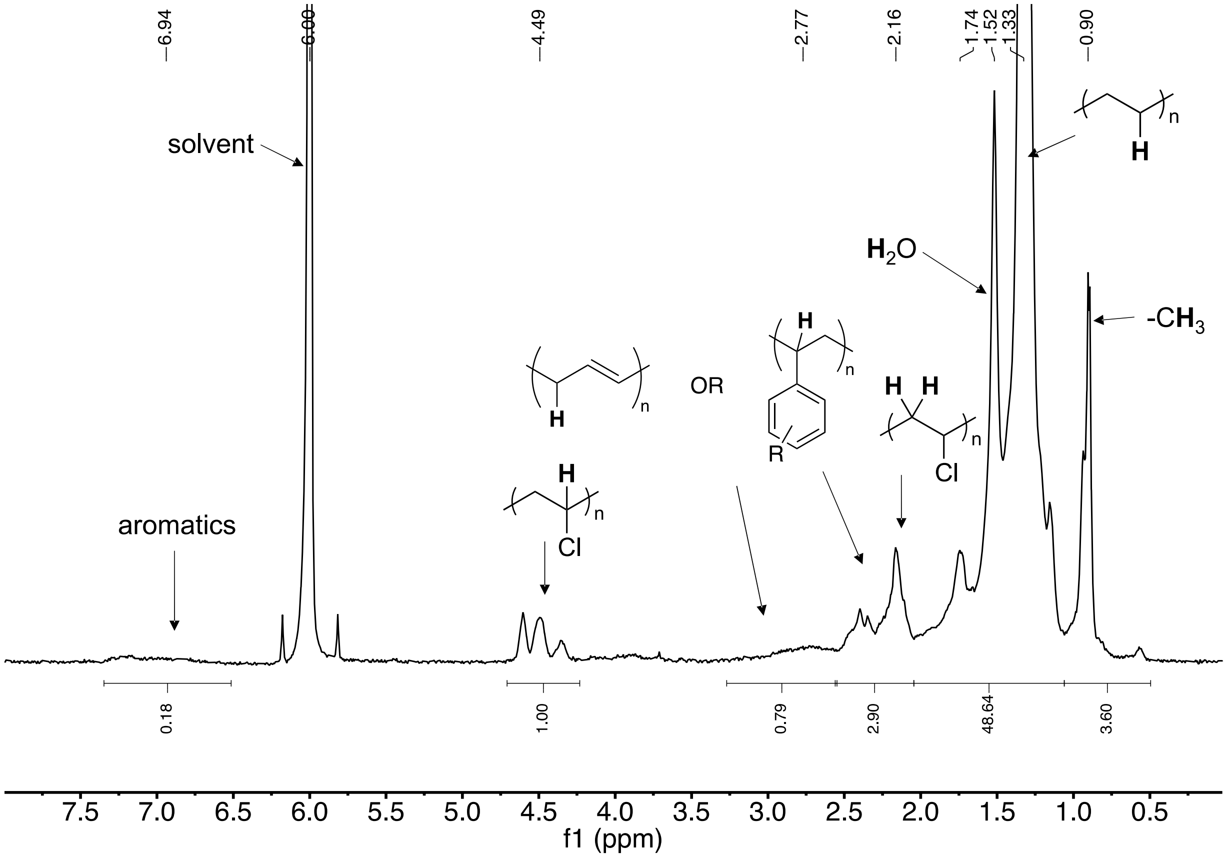

Fig. S71a Zoomed in ^{1}H NMR spectrum, in tetrachloroethane-d_2 (80 °C), of polyethylene product in Table S4, Entry 1c.

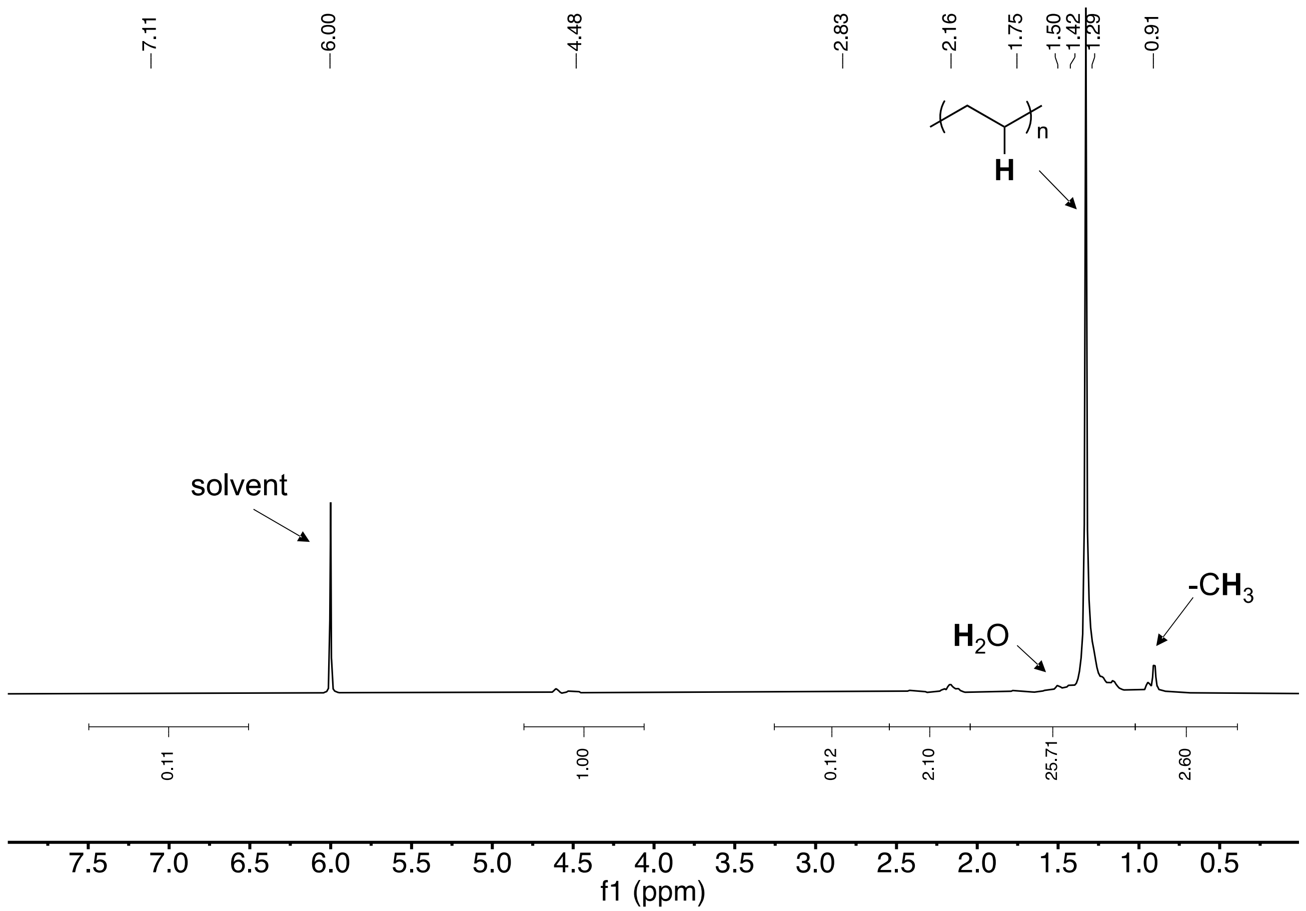

Fig. S72 ^{1}H NMR spectrum, in tetrachloroethane-d_2 (80 °C), of polyethylene product in Table S4, Entry 2a.

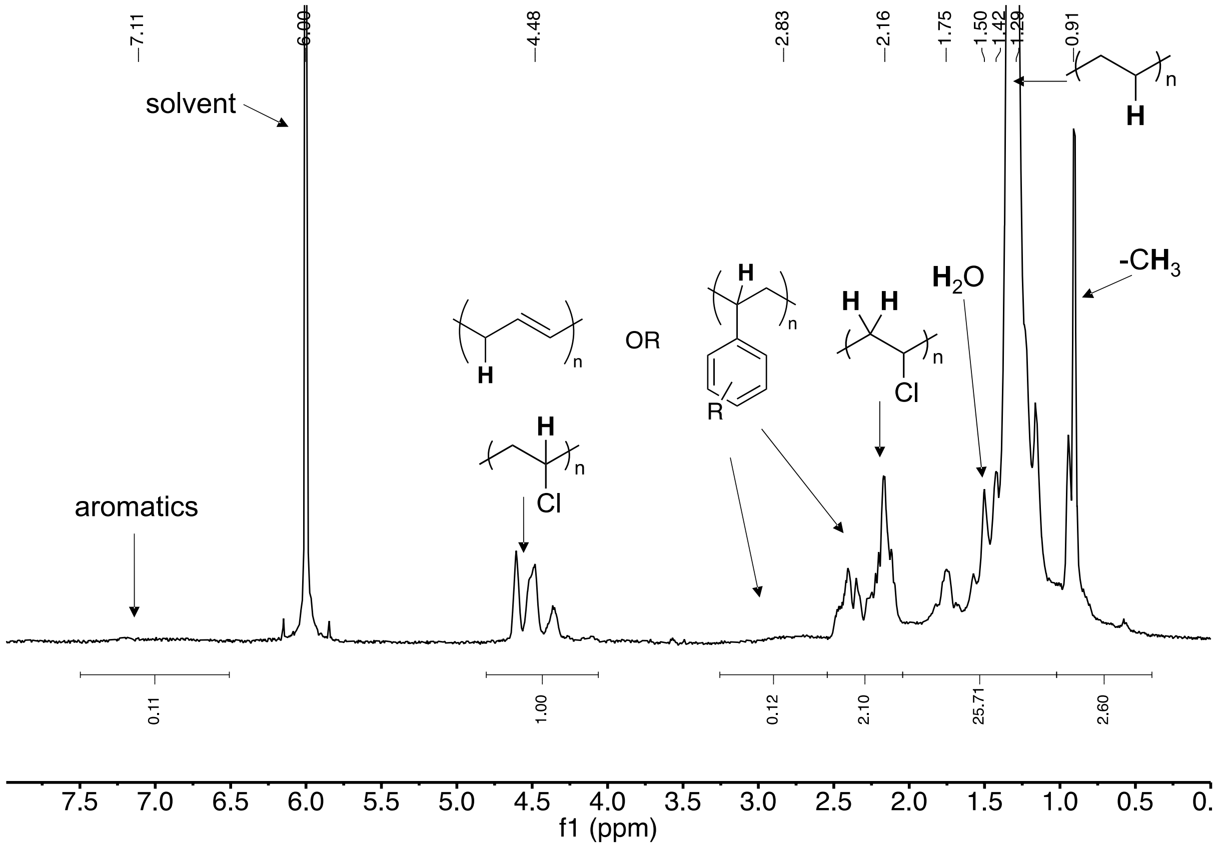

Fig. S72a Zoomed in ^{1}H NMR spectrum, in tetrachloroethane-d_2 (80 °C), of polyethylene product in Table S4, Entry 2a.

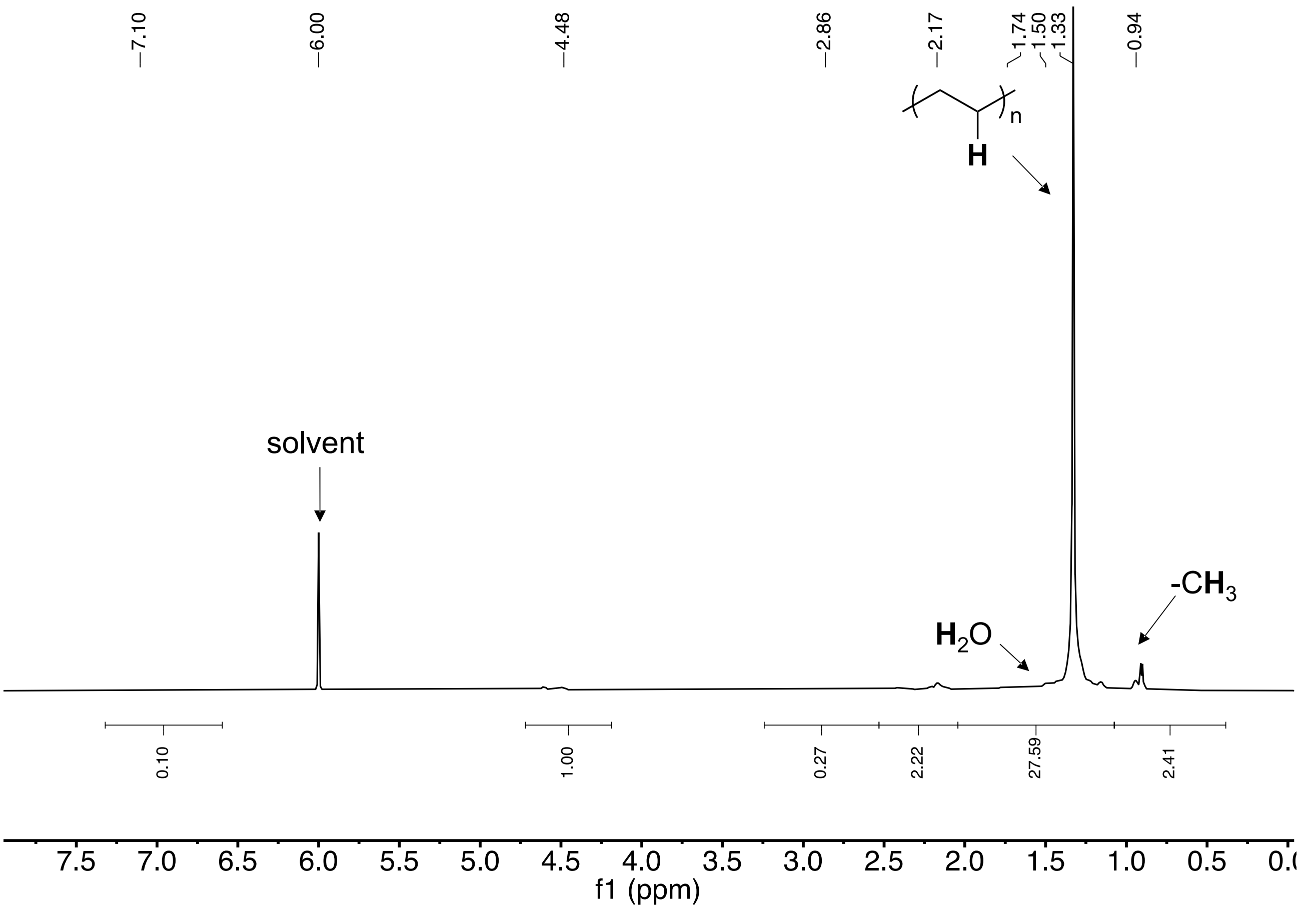

Fig. S73 ^{1}H NMR spectrum, in tetrachloroethane-d_2 (80 °C), of polyethylene product in Table S4, Entry 2b.

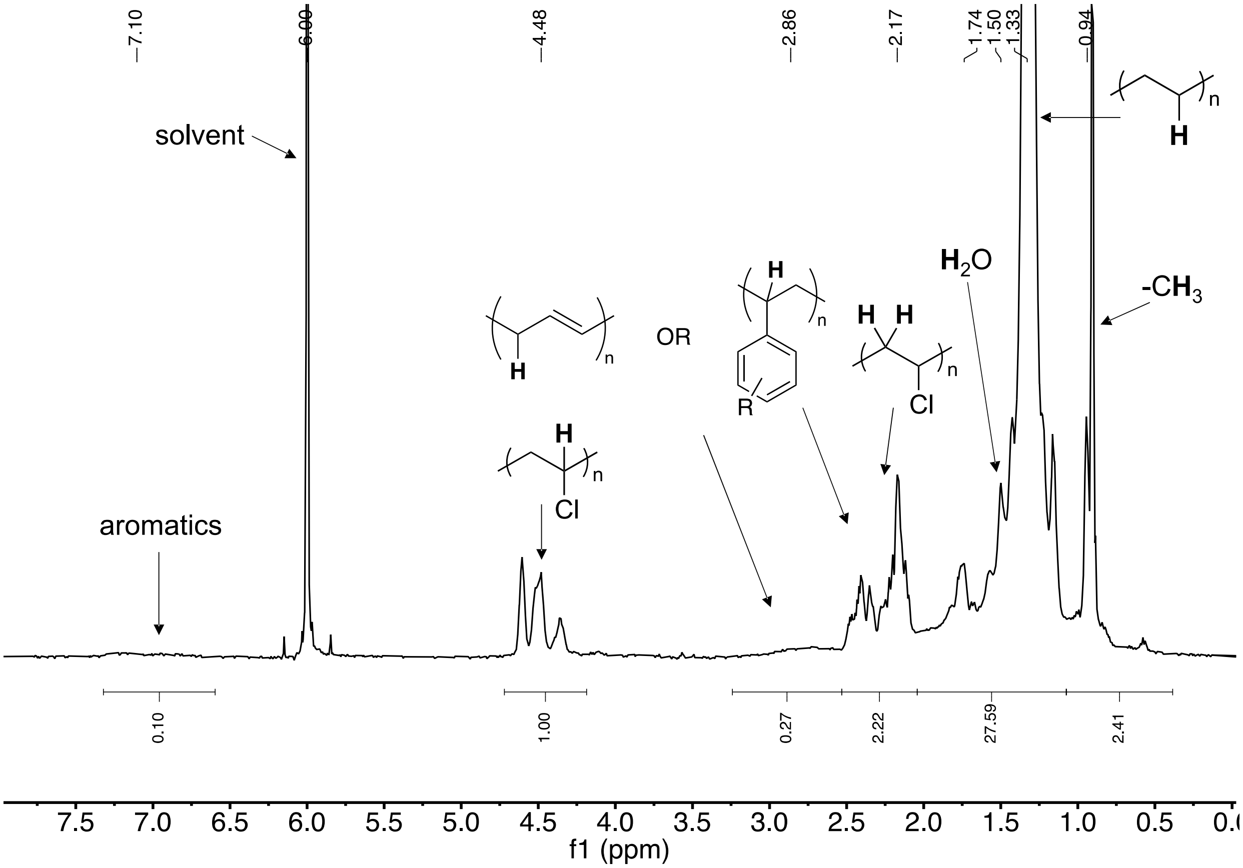

Fig. S73a Zoomed in ^{1}H NMR spectrum, in tetrachloroethane-d_2 (80 °C), of polyethylene product in Table S4, Entry 2b.

.

.

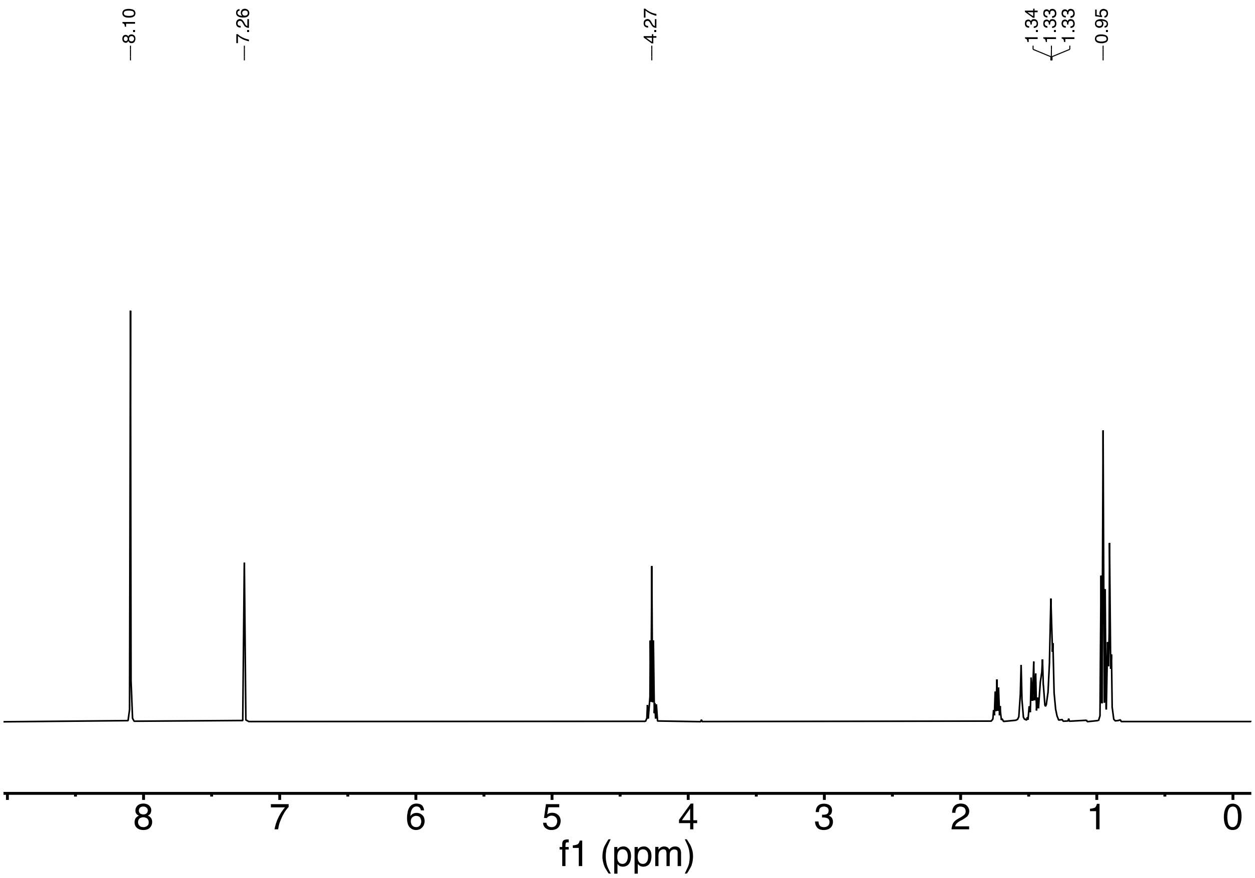

Fig. S74 ^{1}H NMR spectrum, in $CDCl_3$ (25 °C), of chloroform soluble fraction of flexible PVC tubing.

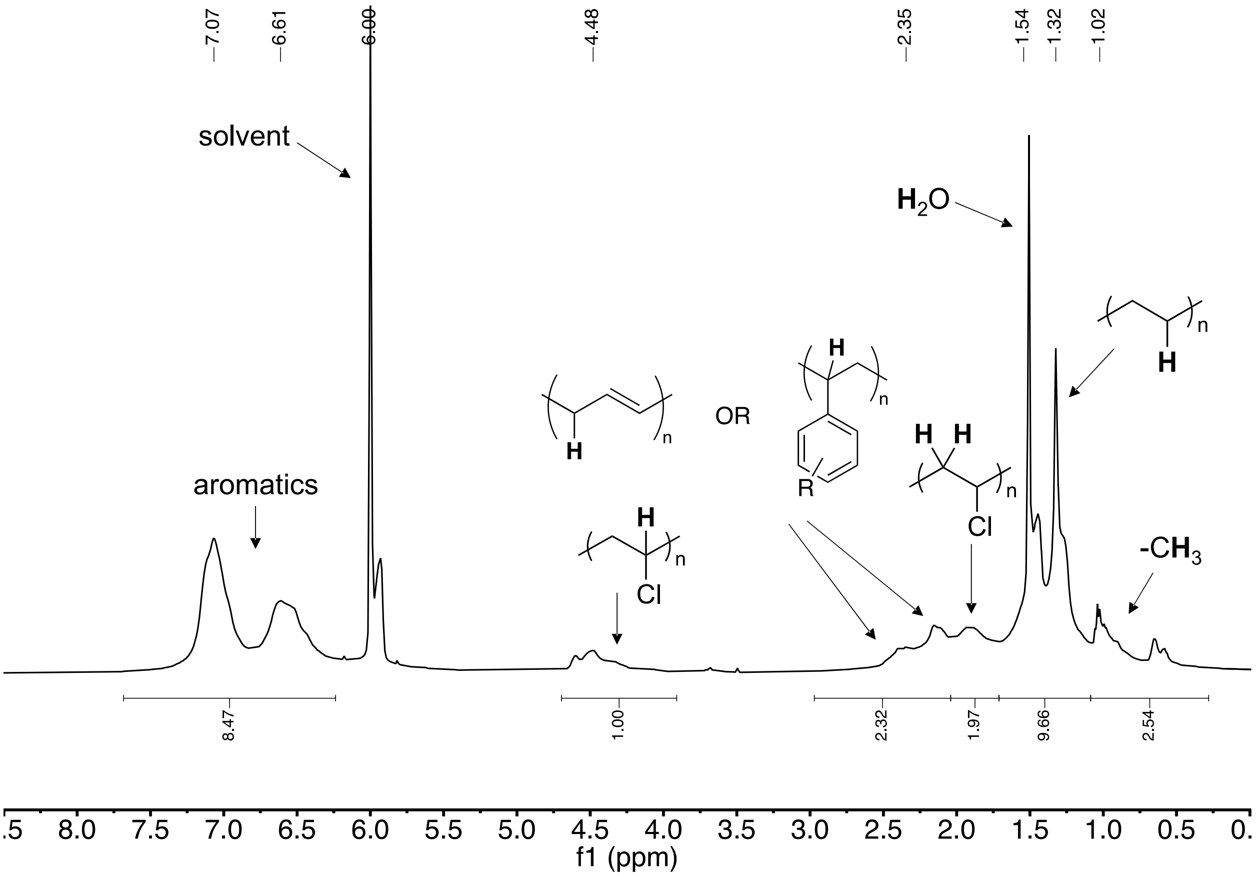

Fig. S75 ^{1}H NMR spectrum, in tetrachloroethane-d_2 (80 °C), of product from PS-PVC mixture.

3. Differential Scanning Calorimetry (DSC)

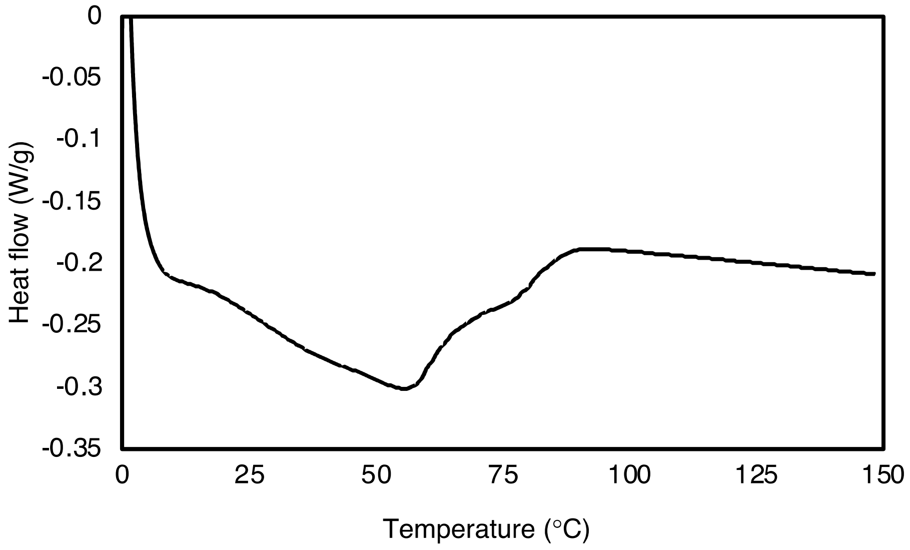

Fig. S76 DSC (2nd heating curve) of Table 1, Entry 1a.

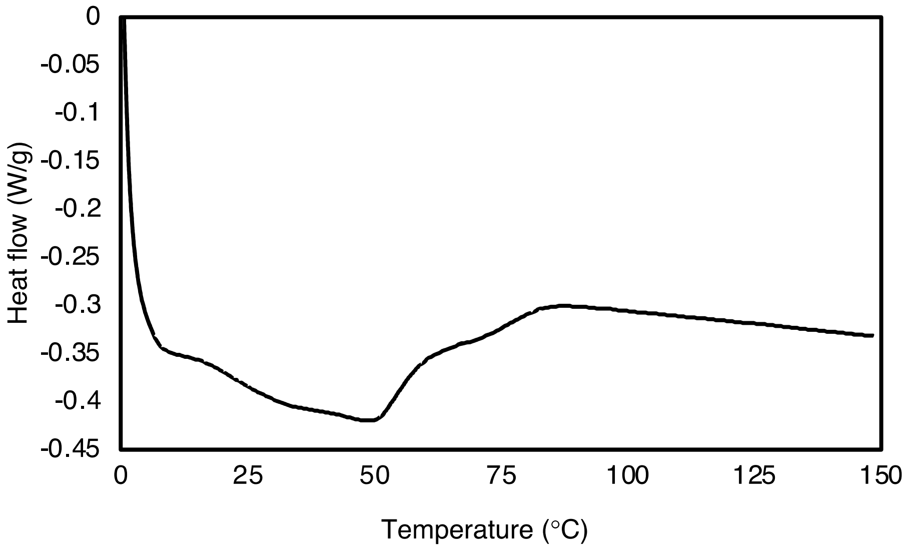

Fig. S77 DSC (2nd heating curve) of Table 1, Entry 1b.

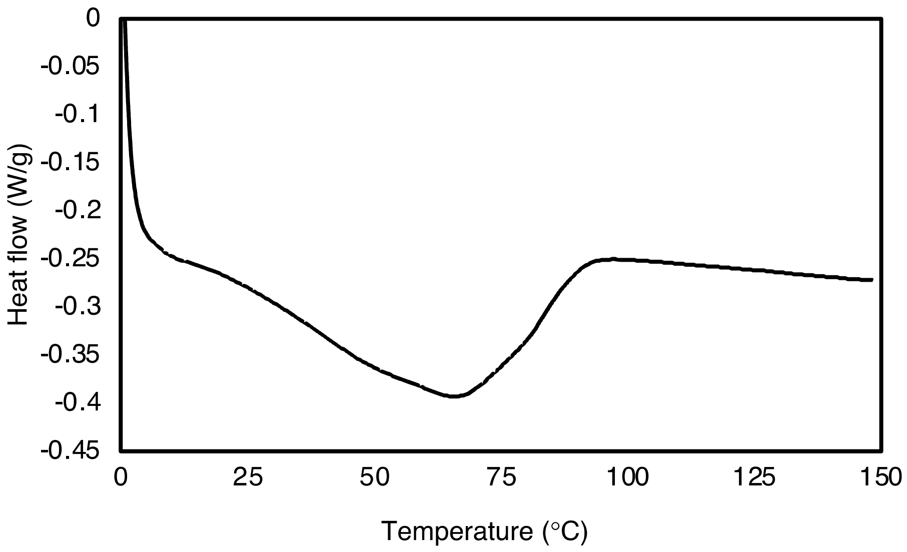

Fig. S78 DSC (2nd heating curve) of Table 1, Entry 2a.

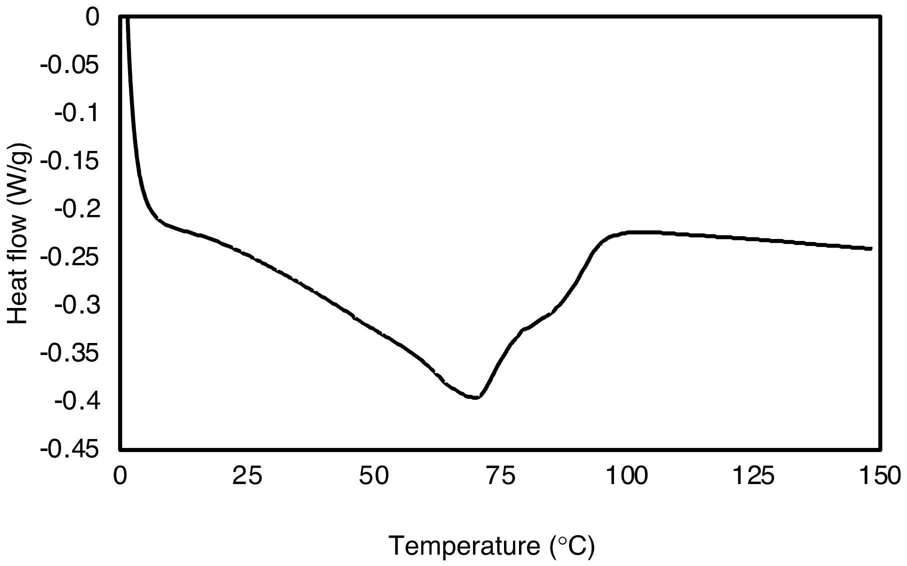

Fig. S79 DSC (2nd heating curve) of Table 1, Entry 2b.

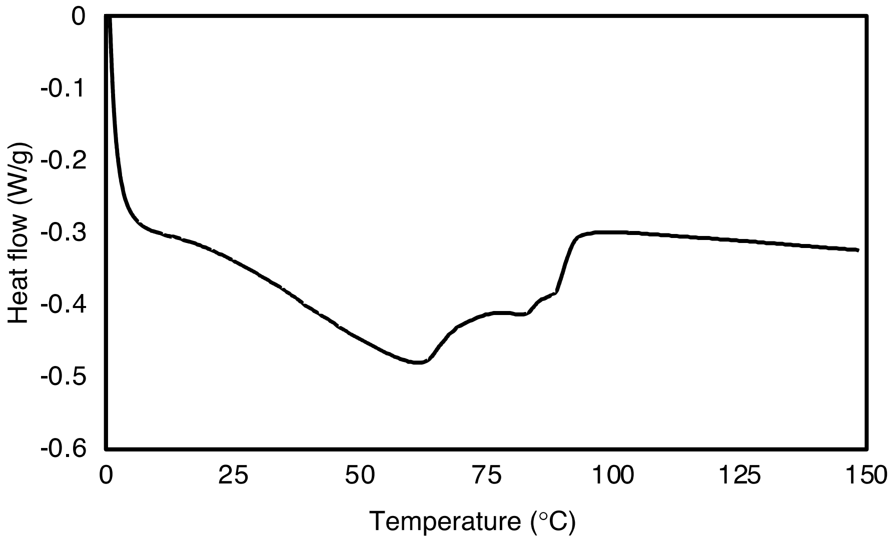

Fig. S80 DSC (2nd heating curve) of Table 1, Entry 3a.

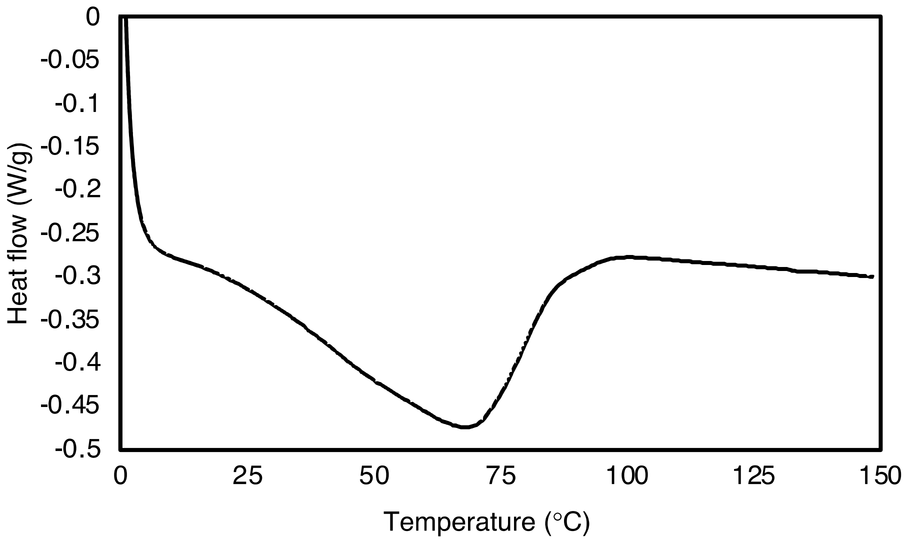

Fig. S81 DSC (2nd heating curve) of Table 1, Entry 3b.

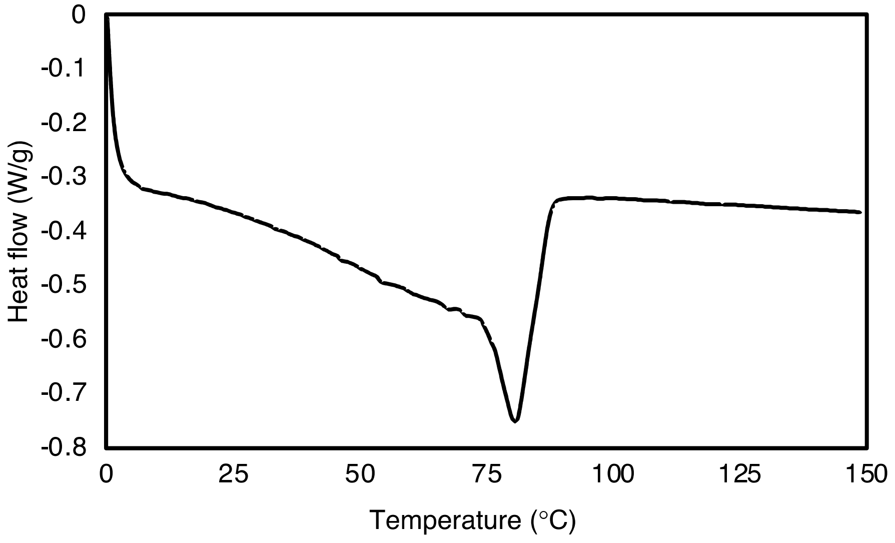

Fig. S82 DSC (2nd heating curve) of Table 1, Entry 4a.

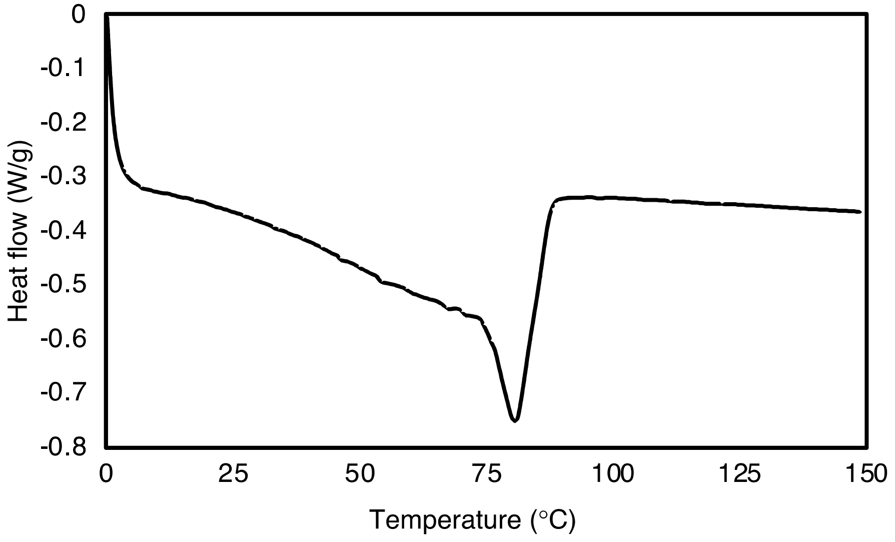

Fig. S83 DSC (2nd heating curve) of Table 1, Entry 4b.

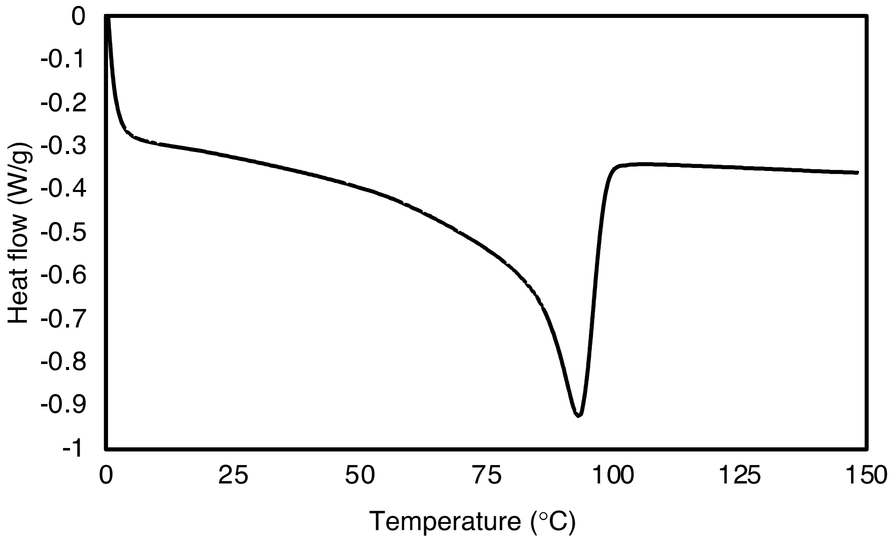

Fig. S84 DSC (2nd heating curve) of Table 1, Entry 5a.

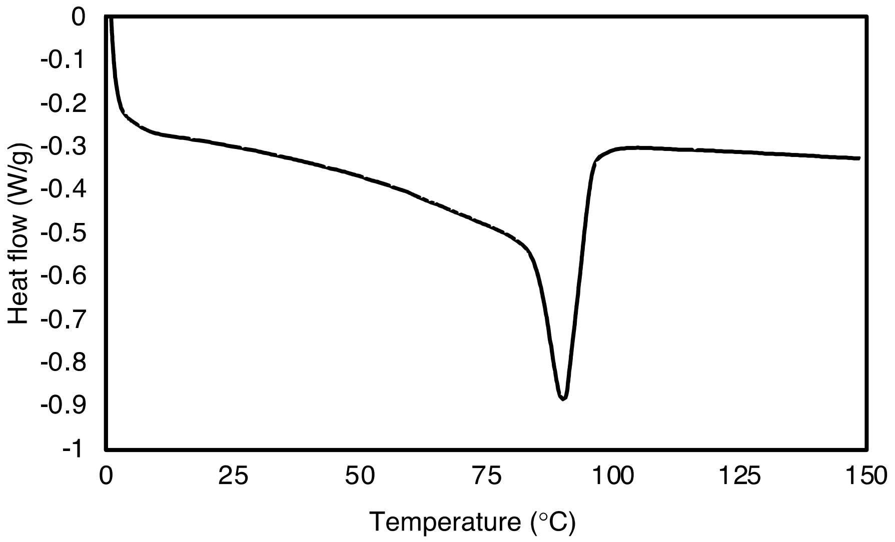

Fig. S85 DSC (2nd heating curve) of Table 1, Entry 5b.

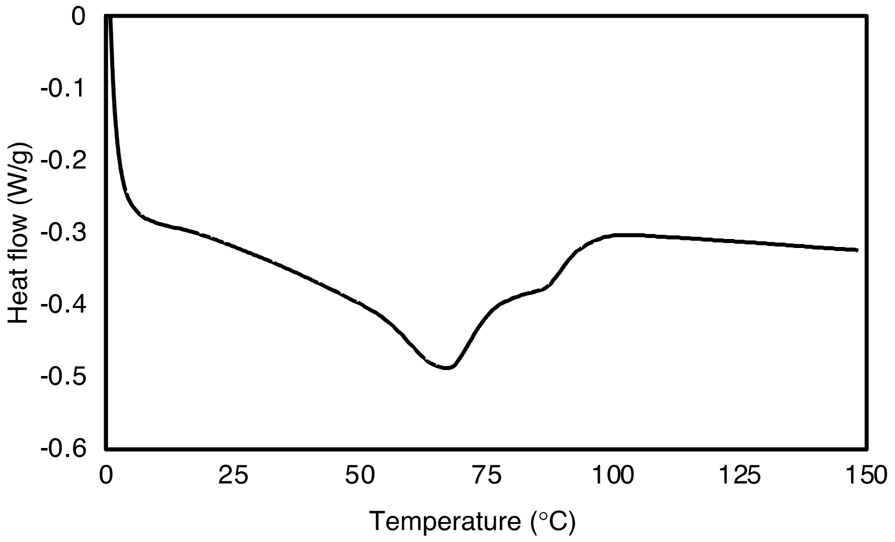

Fig. S86 DSC (2nd heating curve) of Table 1, Entry 6a.

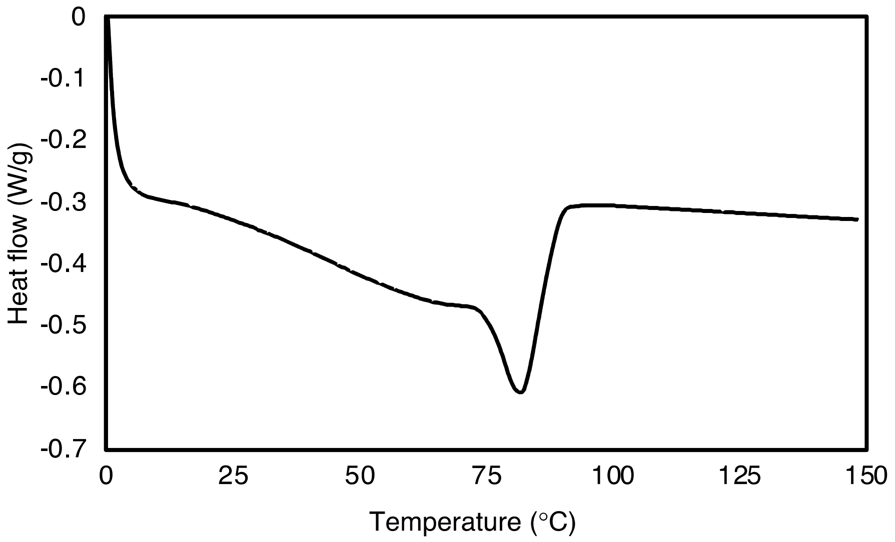

Fig. S87 DSC (2nd heating curve) of Table 1, Entry 6b.

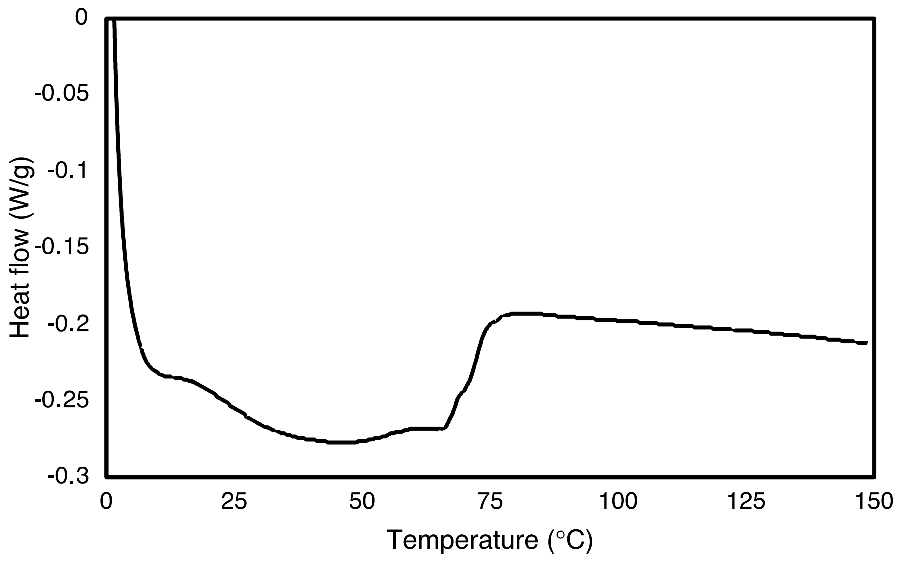

Fig. S88 DSC (2nd heating curve) of Table 2, toy lizard (product).

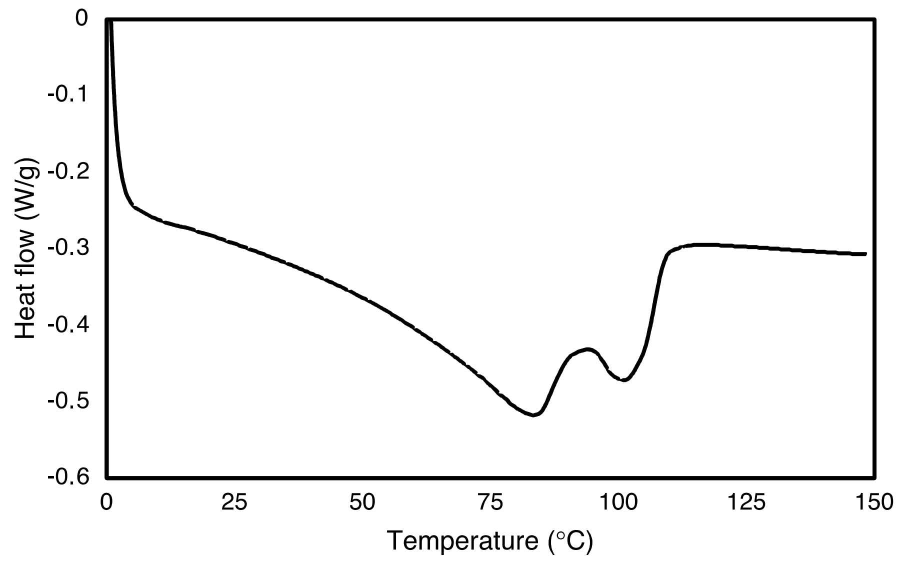

Fig. S89 DSC (2nd heating curve) of Table 2, extracted toy lizard (product).

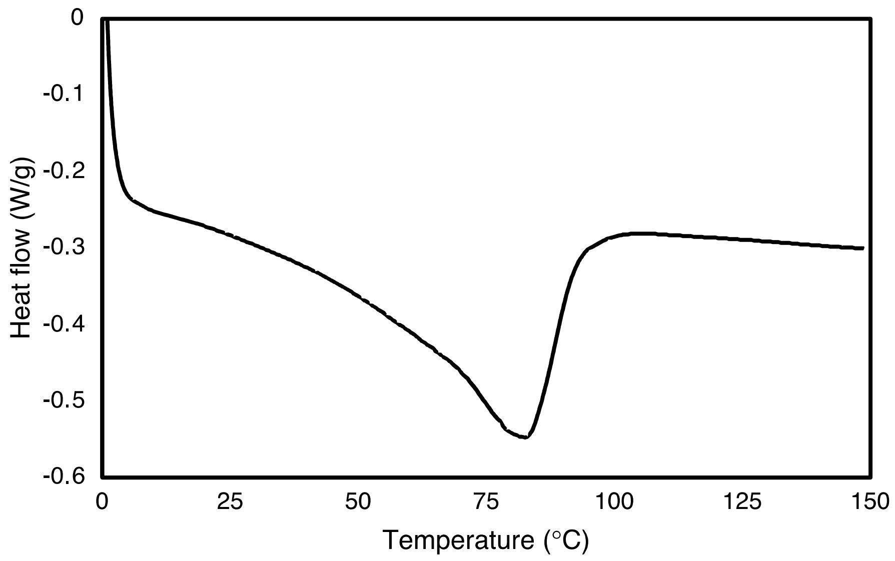

Fig. S90 DSC (2nd heating curve) of Table 2, rigid PVC pipe (product).

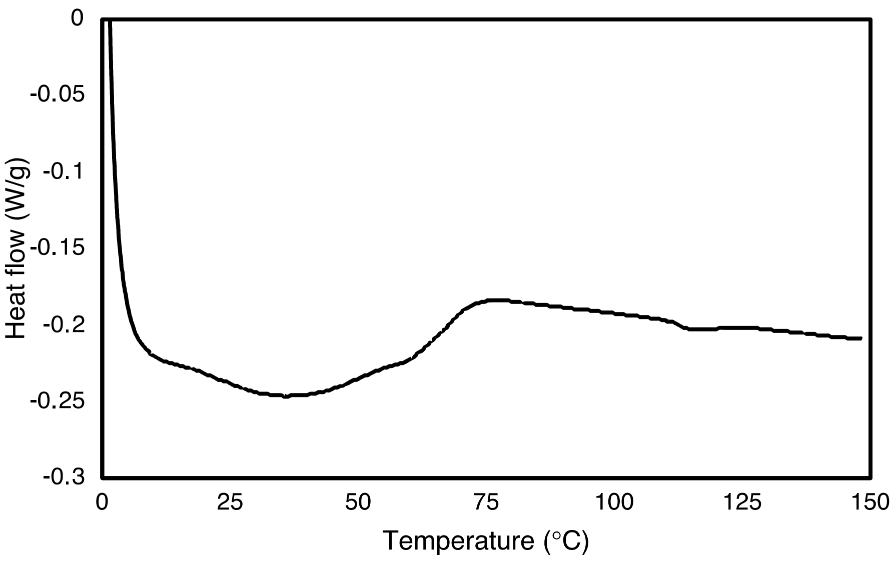

Fig. S91 DSC (2nd heating curve) of Table 2, soft PVC pipe (product).

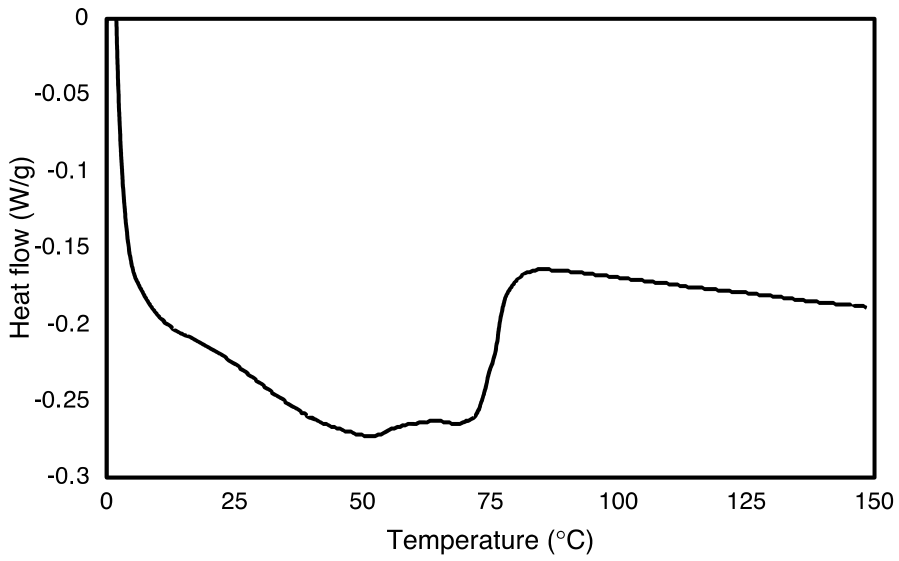

Fig. S92 DSC (2nd heating curve) of Table 2, vinyl record (product).

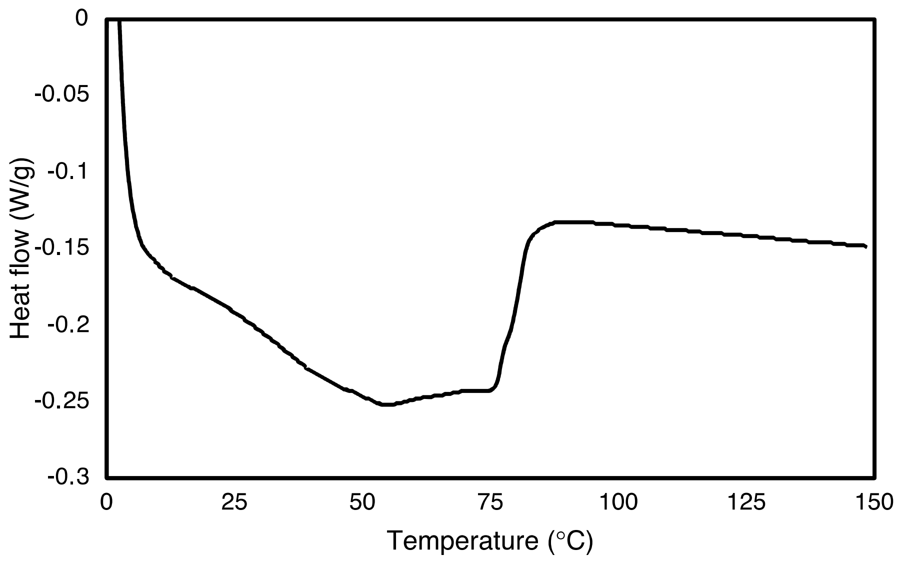

Fig. S93 DSC (2nd heating curve) of Table 2, extracted vinyl record (product).

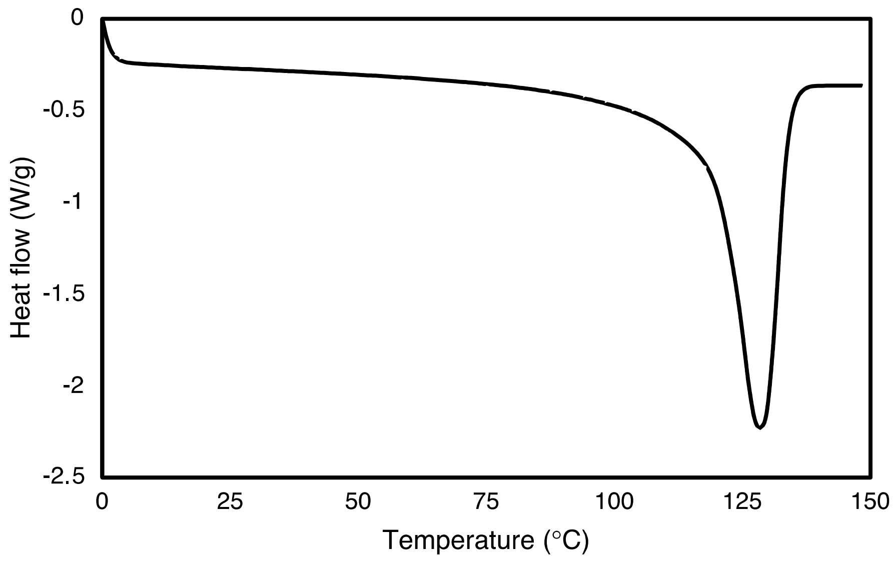

Fig. S94 DSC (2nd heating curve) of HDPE pellets (CAS: 9002-88-4, Sigma Aldrich).

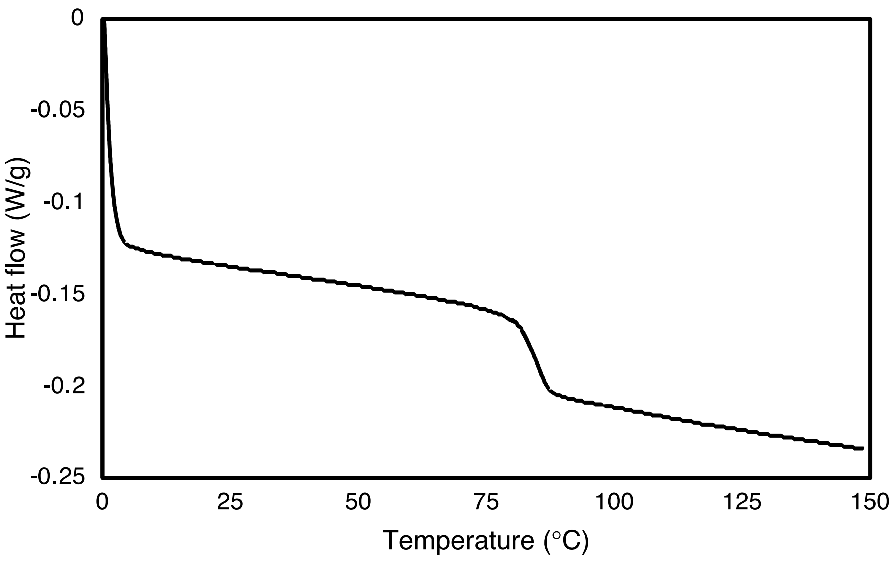

Fig. S95 DSC (2nd heating curve) of low molecular weight PVC (CAS: 9002-86-2, Product #: 81388, Sigma Aldrich).

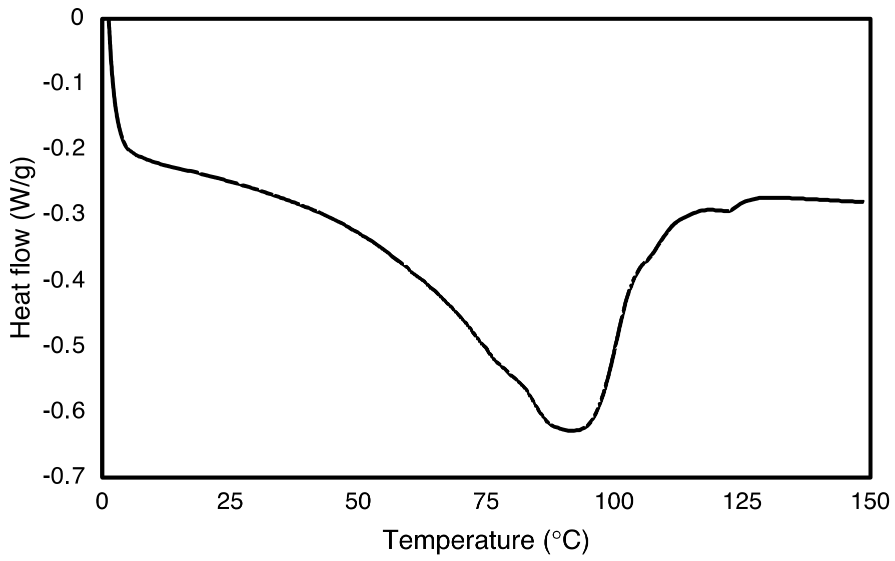

Fig. S96 DSC (2nd heating curve) of CH_2Cl_2 soluble fraction from the mixed PE/PVC reaction.

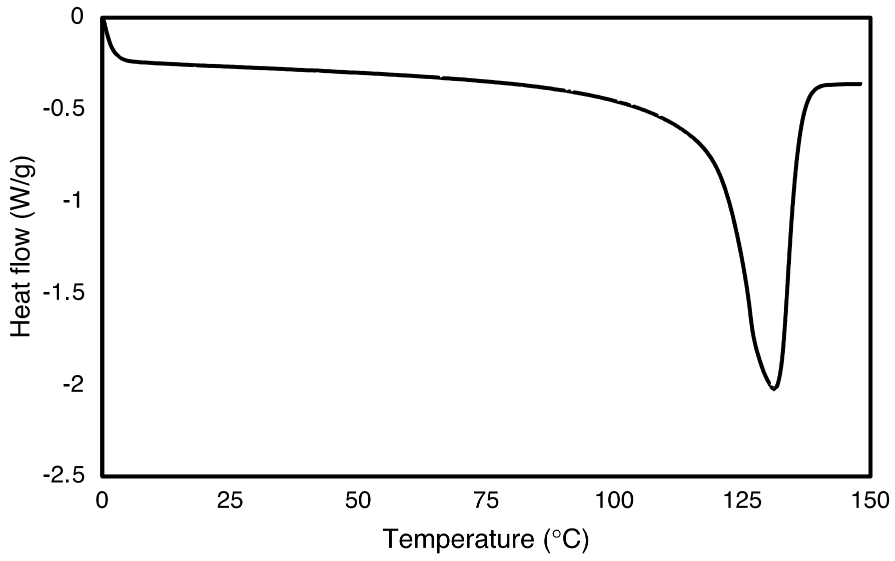

Fig. S97 DSC (2nd heating curve) of poly(ethylene) bead of the insoluble fraction from the mixed PE/PVC reaction.

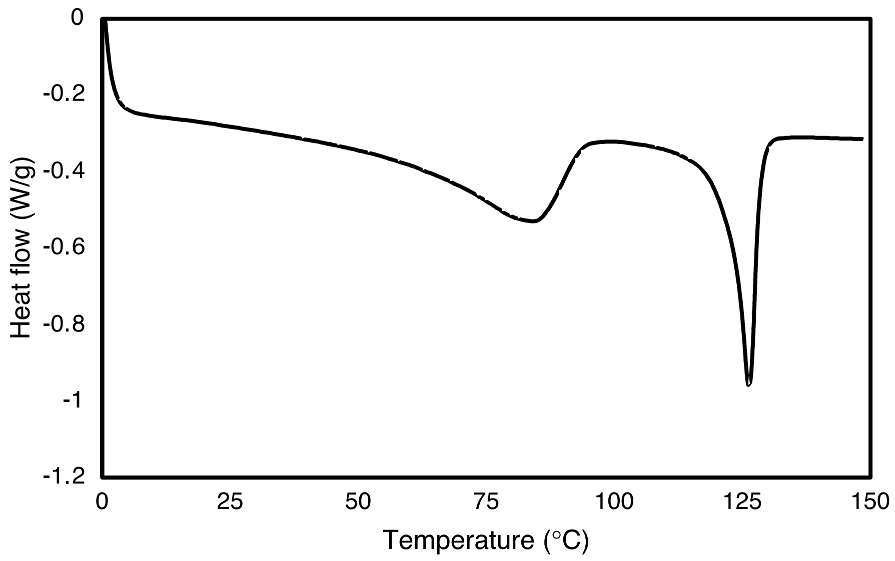

Fig. S98 DSC (2nd heating curve) of mixed Table 1, Entry 4a and HDPE (83/17 wt%).

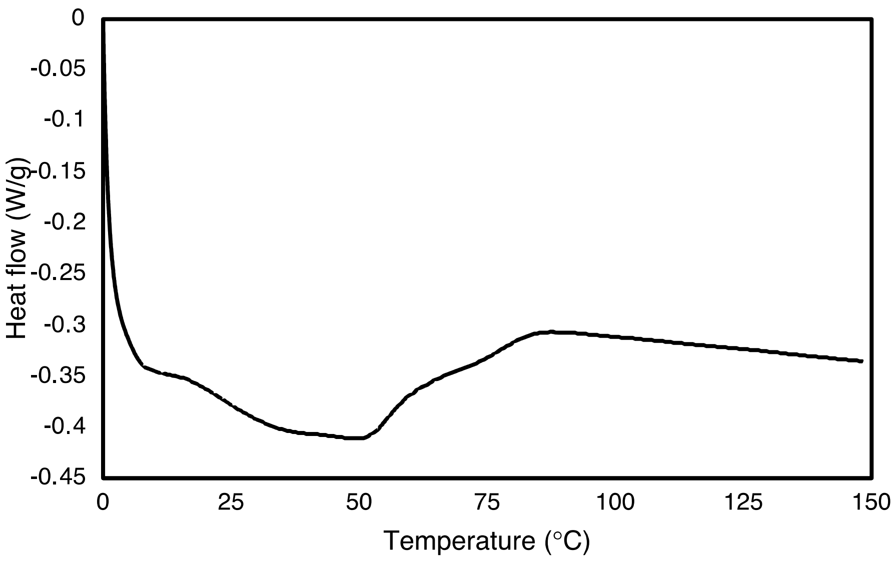

Fig. S99 DSC (2nd heating curve) of Table S1, Entry 1.

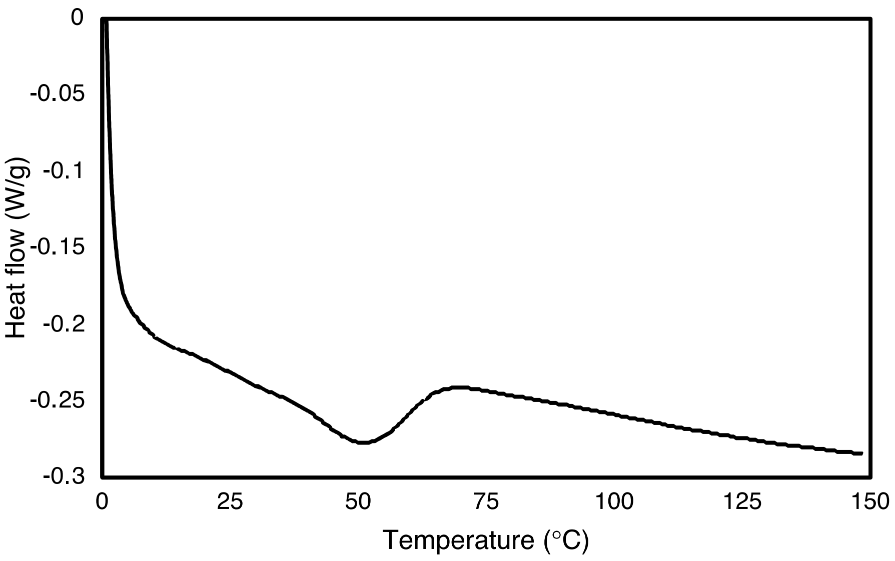

Fig. S100 DSC (2nd heating curve) of Table S2, Entry 1.

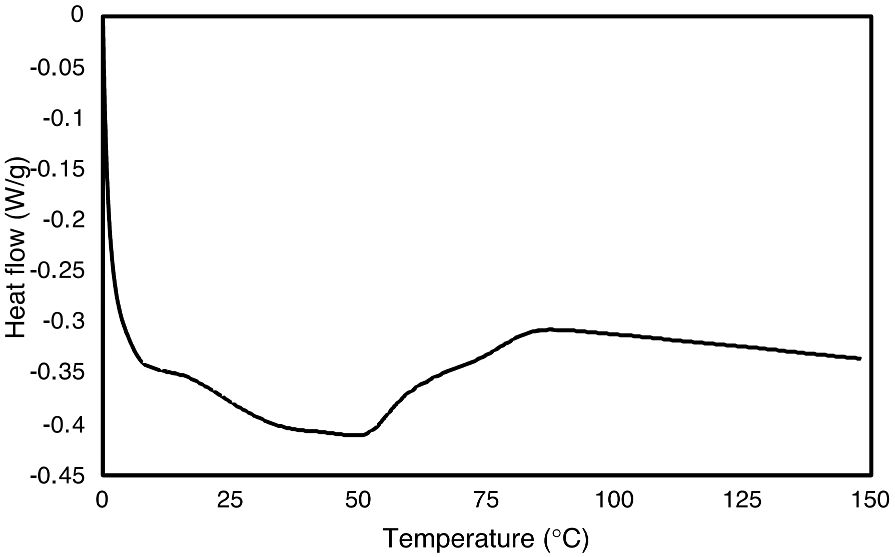

Fig. S101 DSC (2nd heating curve) of Table S3, Entry 1.

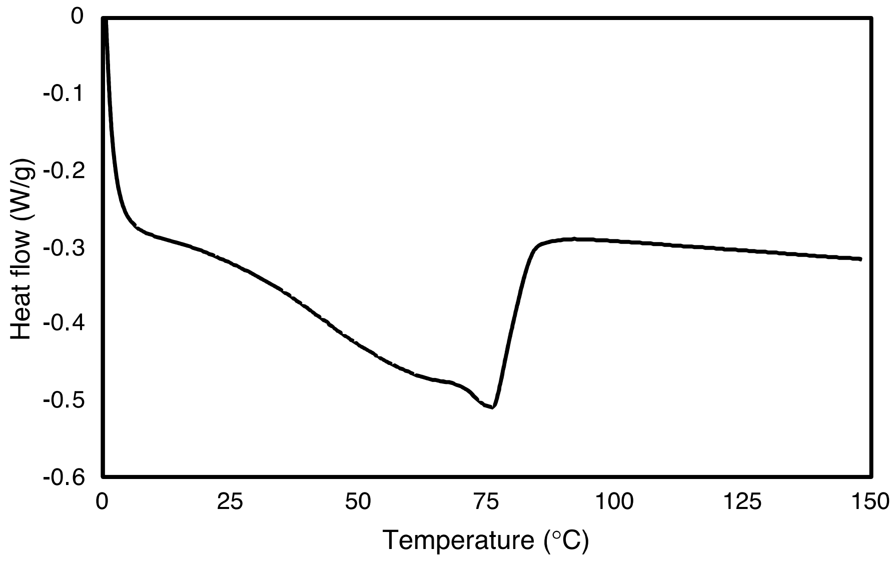

Fig. S102 DSC (2nd heating curve) of Table S3, Entry 2.

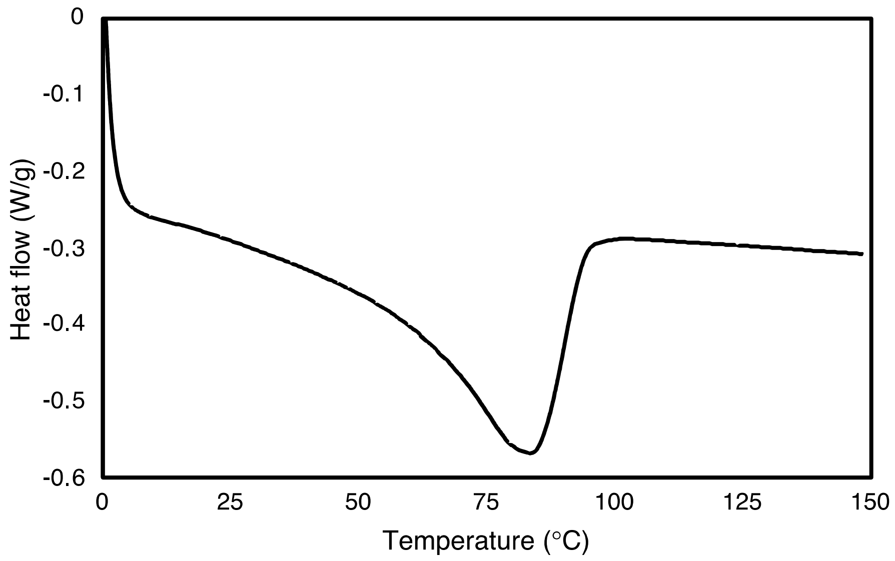

Fig. S103 DSC (2nd heating curve) of Table S3, Entry 3.

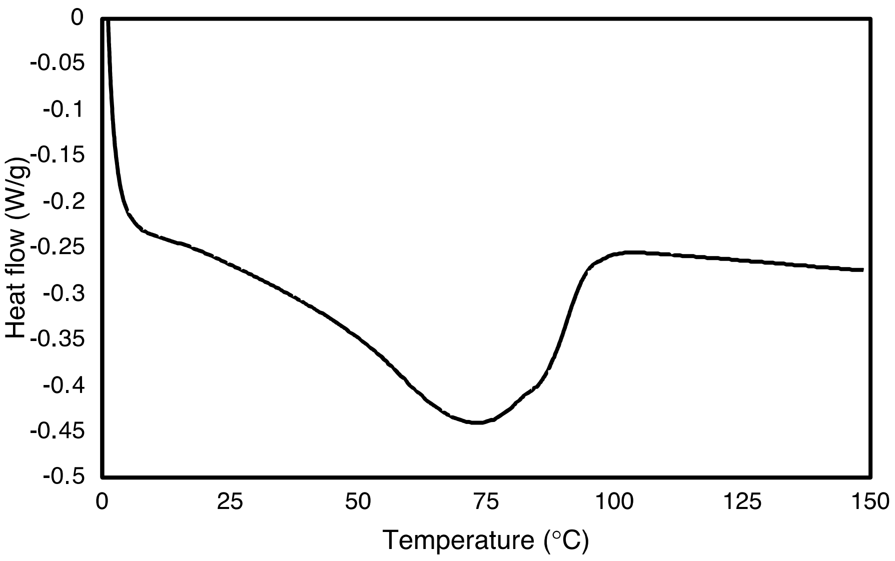

Fig. S104 DSC (2nd heating curve) of Table S3, Entry 4.

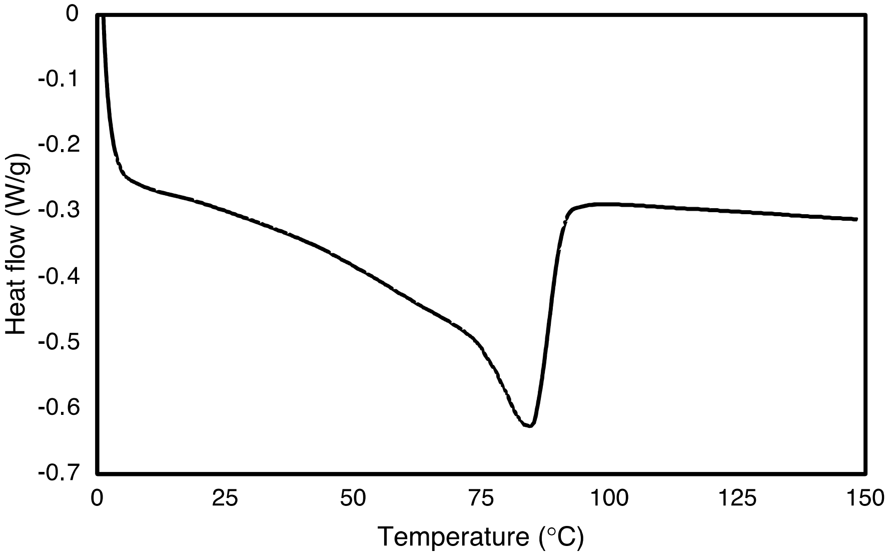

Fig. S105 DSC (2nd heating curve) of Table S3, Entry 5a.

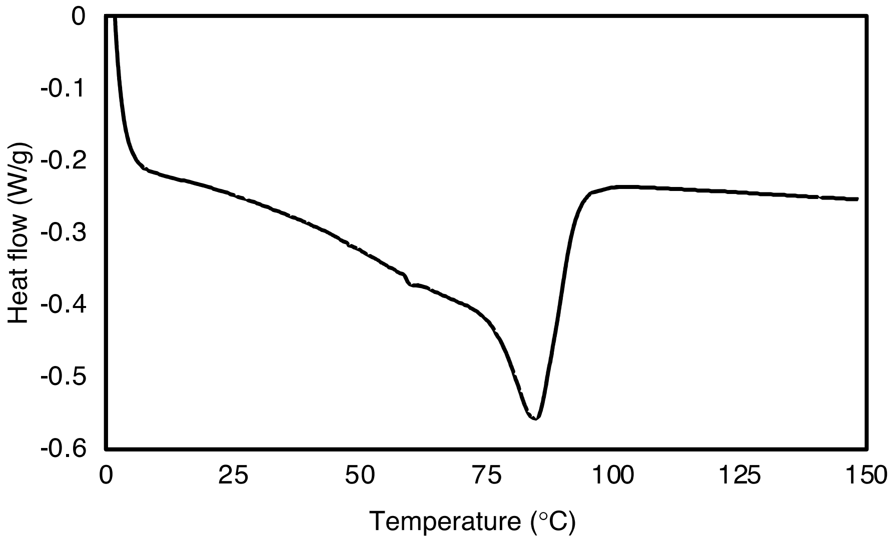

Fig. S106 DSC (2nd heating curve) of Table S3, Entry 5b.

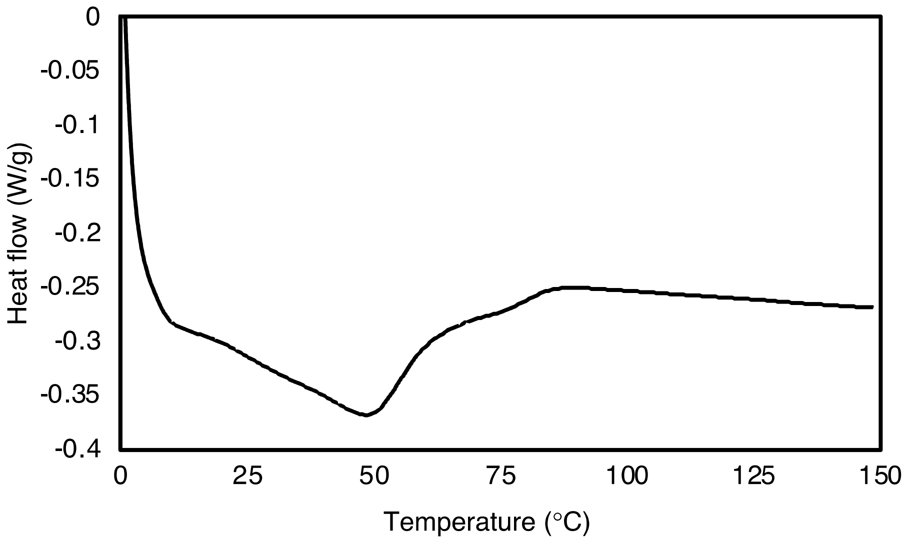

Fig. S107 DSC (2nd heating curve) of Table S4, Entry 1a.

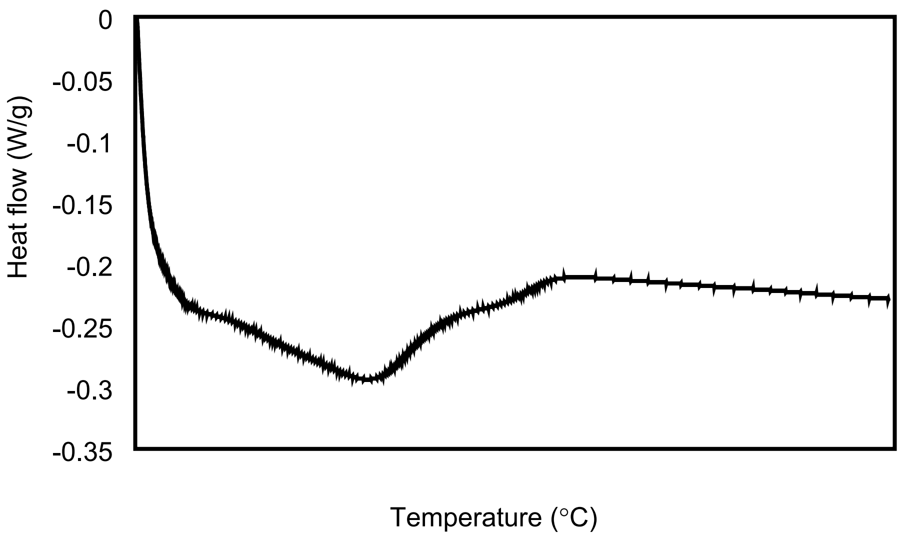

Fig. S108 DSC (2nd heating curve) of Table S4, Entry 1b.

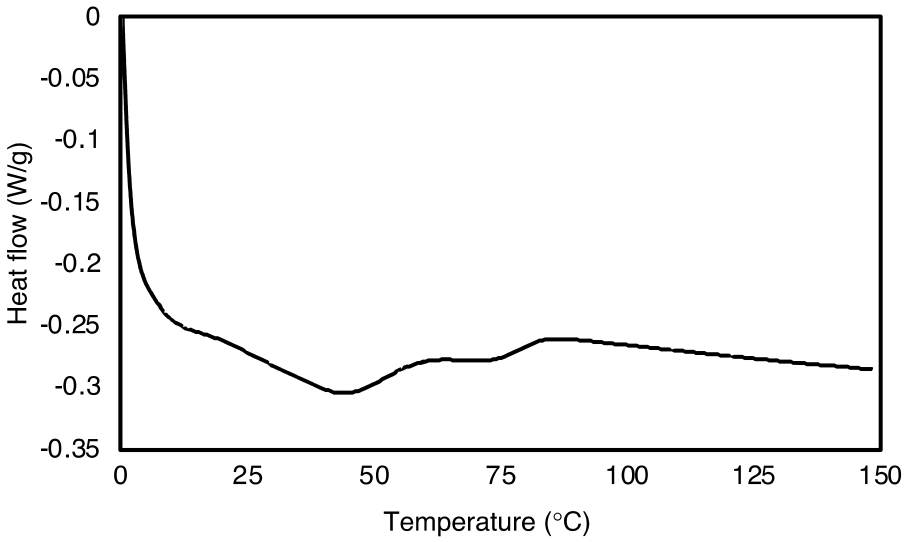

Fig. S109 DSC (2nd heating curve) of Table S4, Entry 1c.

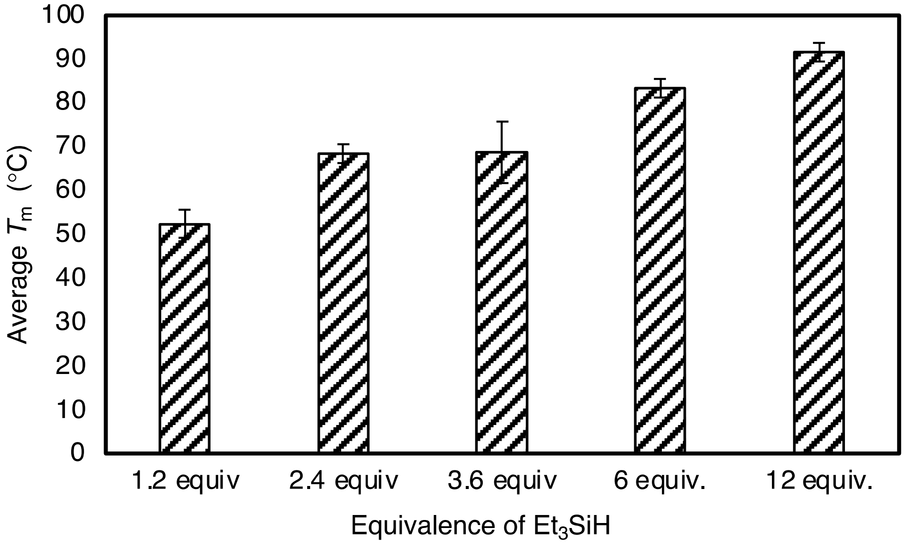

Fig. S110 Summary of melting point temperatures for branched PE products with varied branching.

4. Thermogravimetric Analysis (TGA)

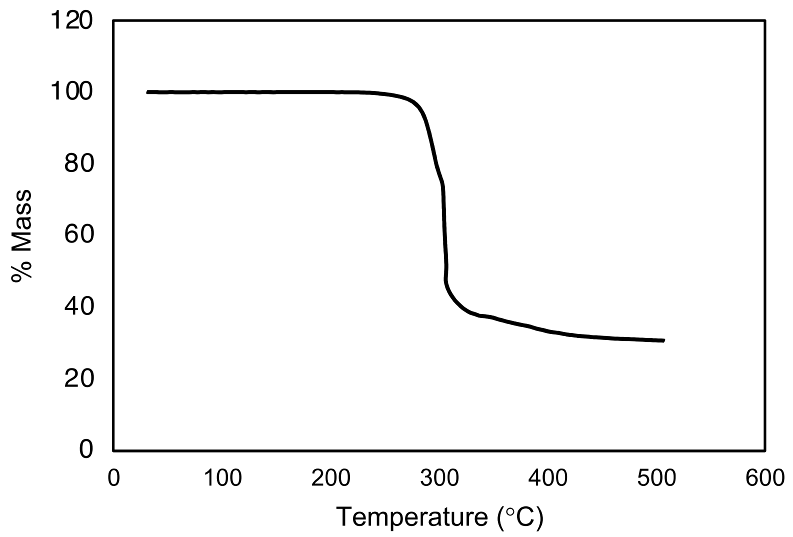

Fig. S111 TGA curve of low molecular weight PVC (CAS: 9002-86-2, Product #: 81388, Sigma Aldrich).

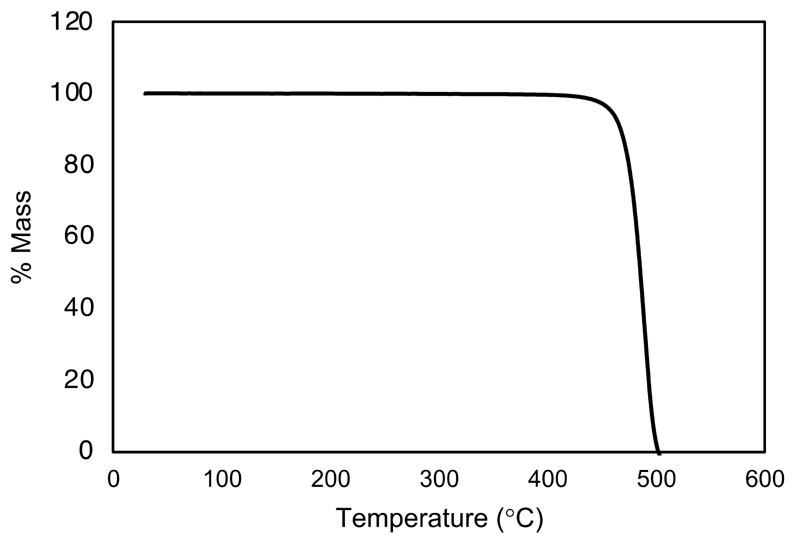

Fig. S112 TGA curve of HDPE pellets (CAS: 9002-88-4, Sigma Aldrich).

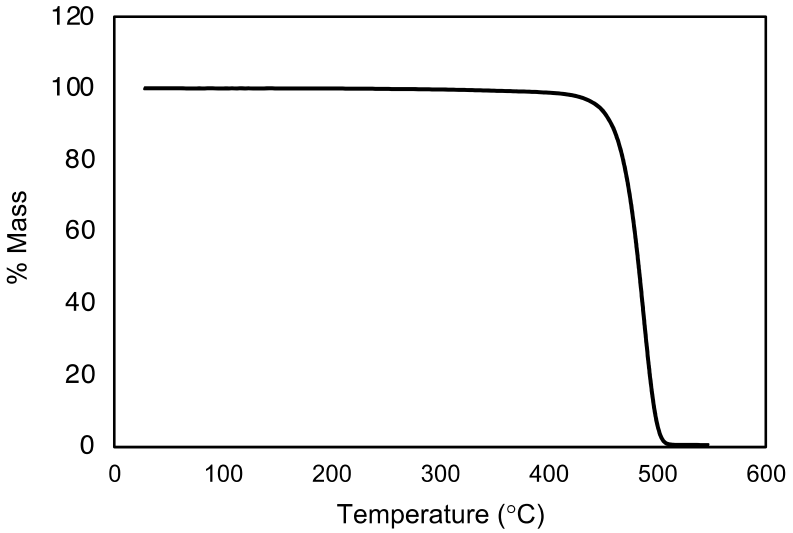

Fig. S113 TGA curve of Table 1, Entry 1a.

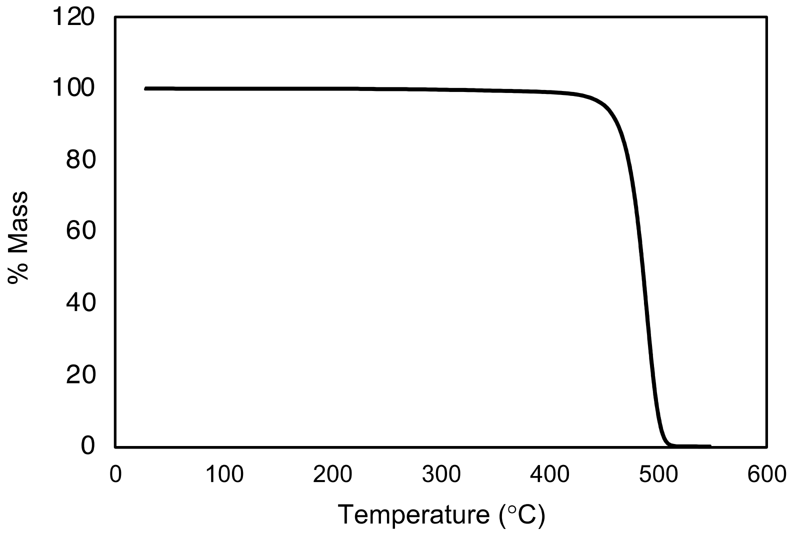

Fig. S114 TGA curve of Table 1, Entry 1b.

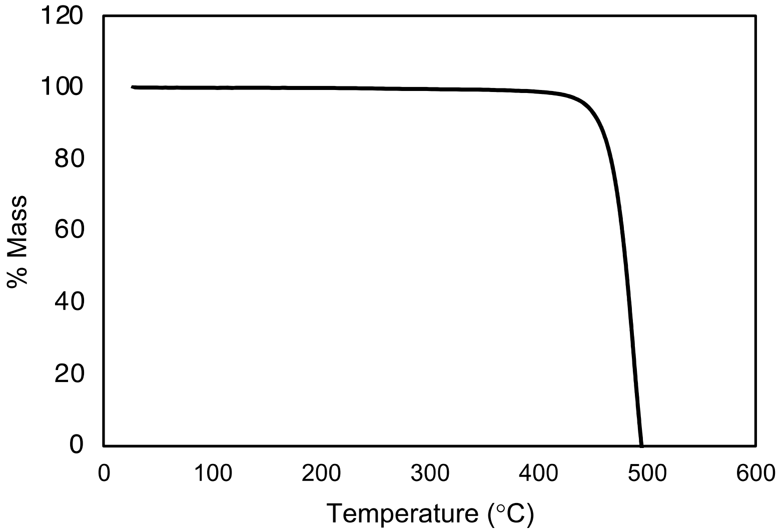

Fig. S115 TGA curve of Table 1, Entry 2a.

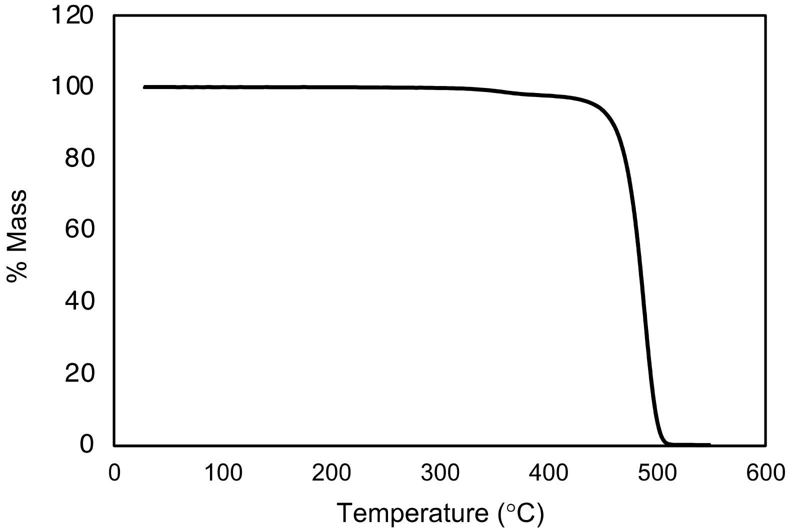

Fig. S116 TGA curve of Table 1, Entry 2b.

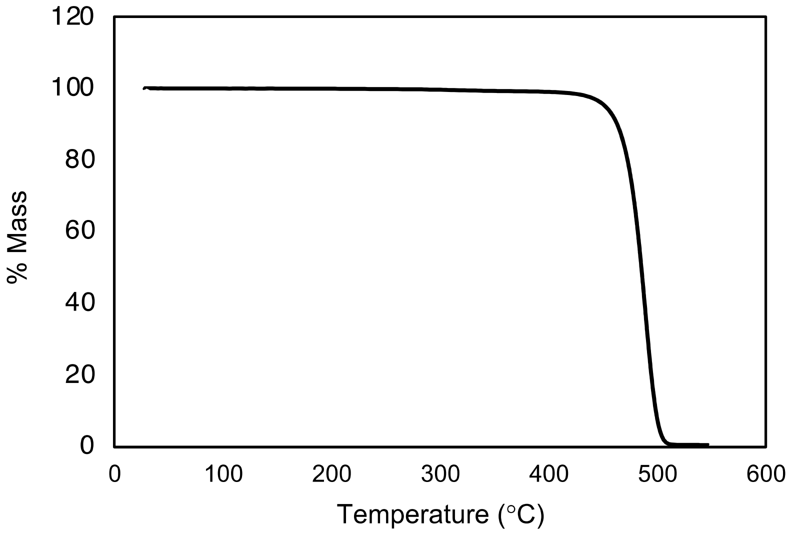

Fig. S117 TGA curve of Table 1, Entry 3a.

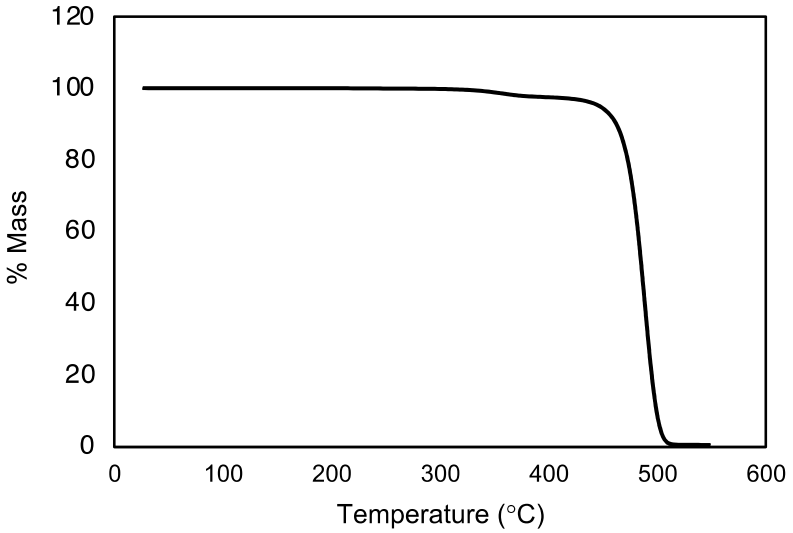

Fig. S118 TGA curve of Table 1, Entry 3b.

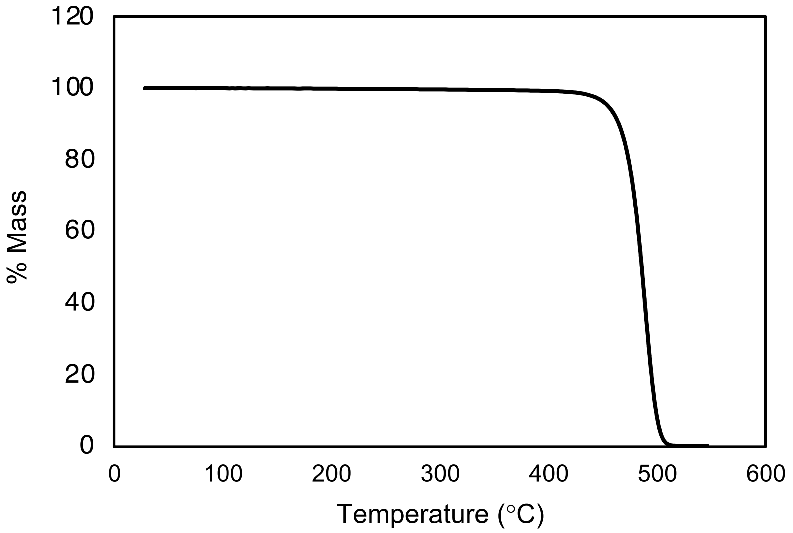

Fig. S119 TGA curve of Table 1, Entry 4a.

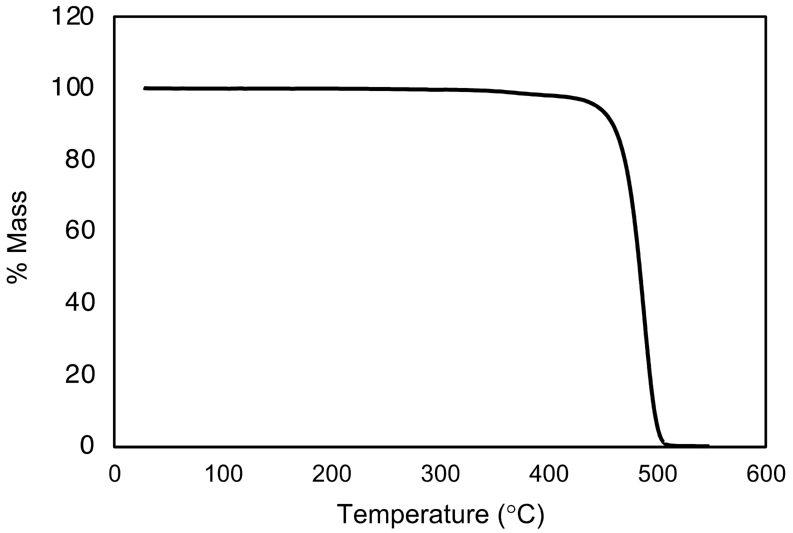

Fig. S120 TGA curve of Table 1, Entry 4b.

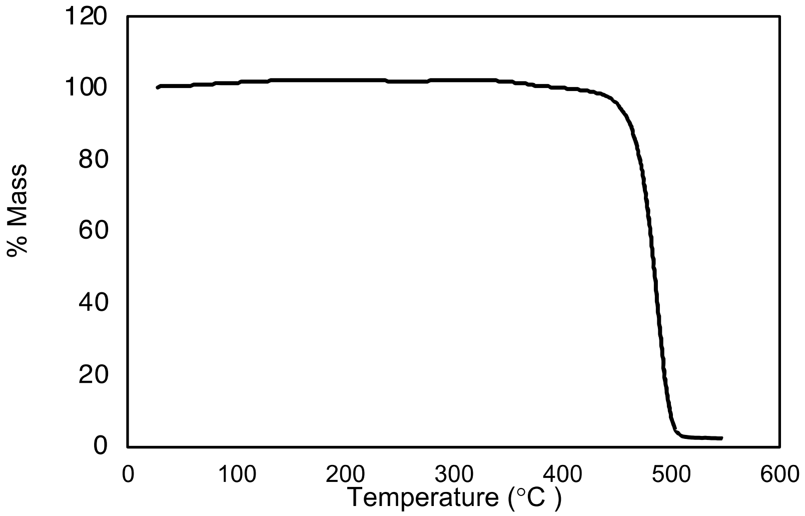

Fig. S121 TGA curve of Table 1, Entry 5a.

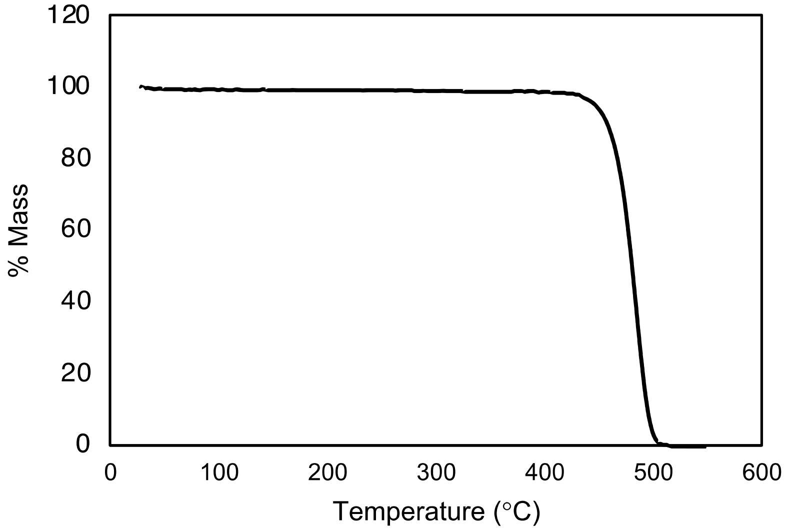

Fig. S122 TGA curve of Table 1, Entry 5b.

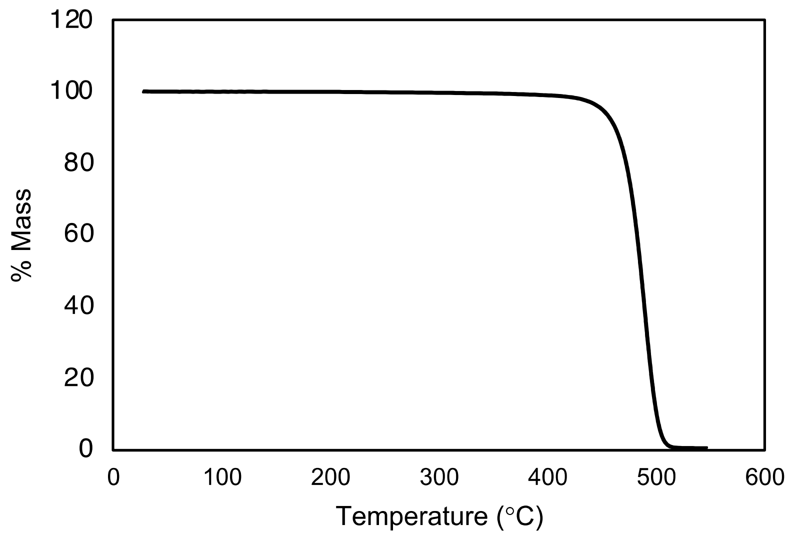

Fig. S123 TGA curve of Table 1, Entry 6a.

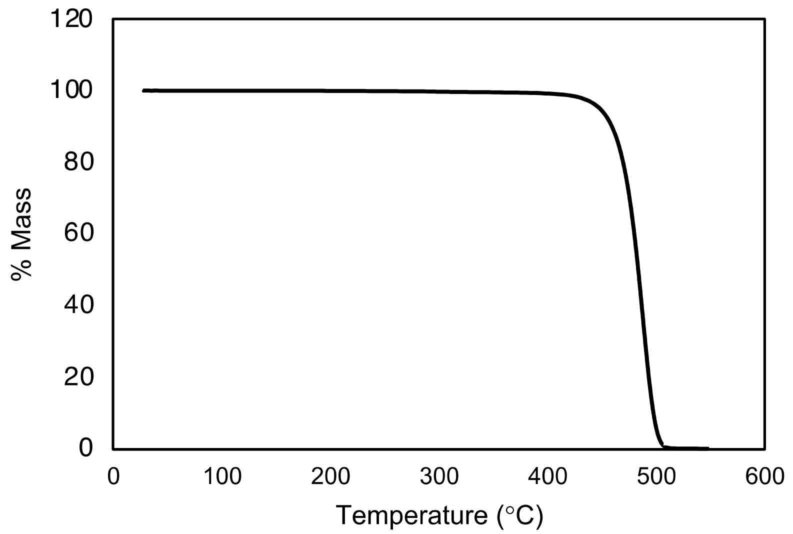

Fig. S124 TGA curve of Table 1, Entry 6b.

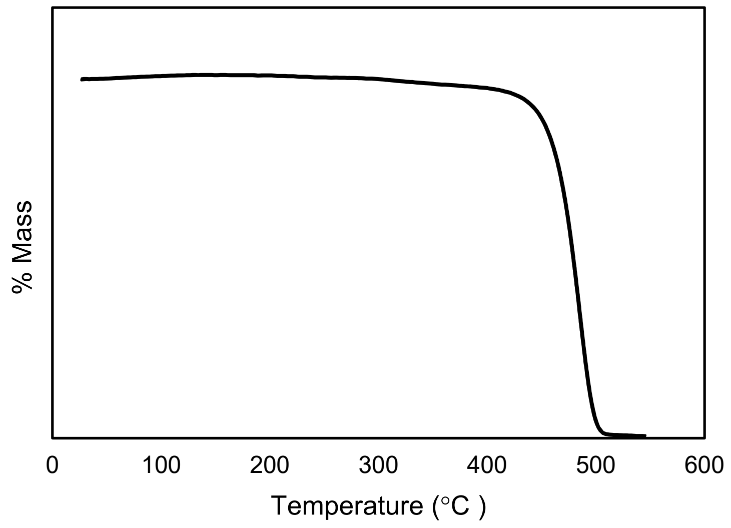

Fig. S125 TGA curve of Table 2, Entry 1.

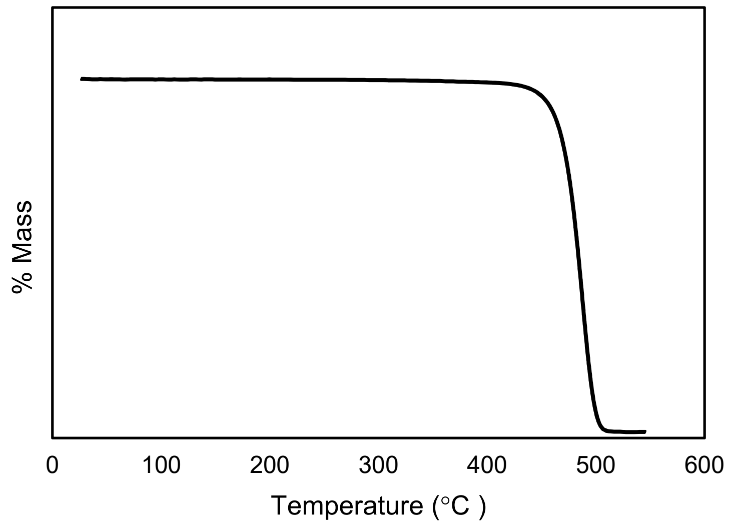

Fig. S126 TGA curve of Table 2, Entry 2.

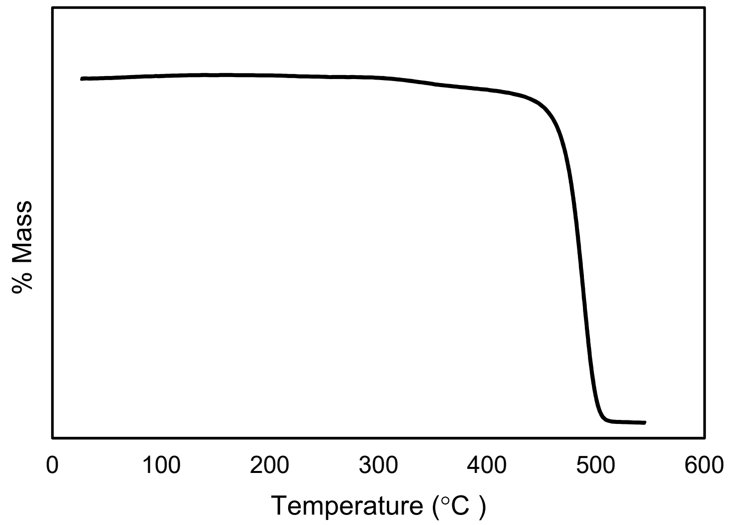

Fig. S127 TGA curve of Table 2, Entry 3.

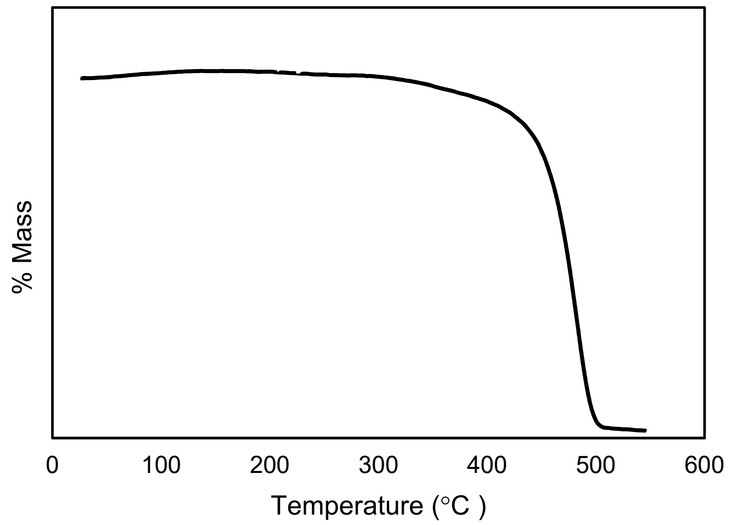

Fig. S128 TGA curve of Table 2, Entry 4.

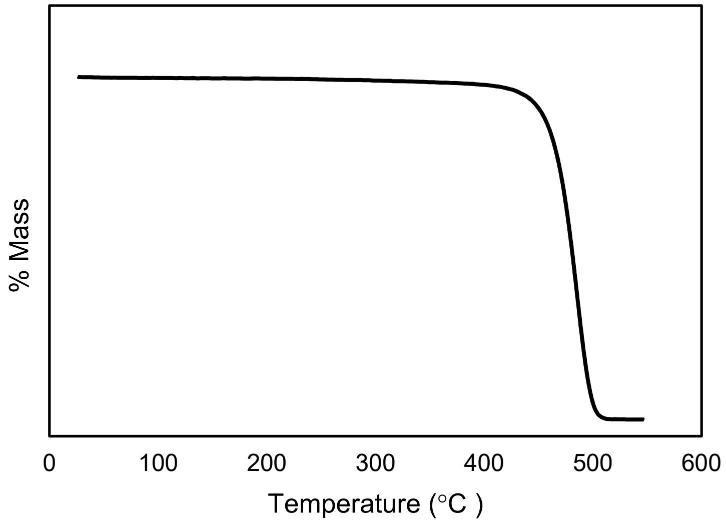

Fig. S129 TGA curve of Table 2, Entry 5.

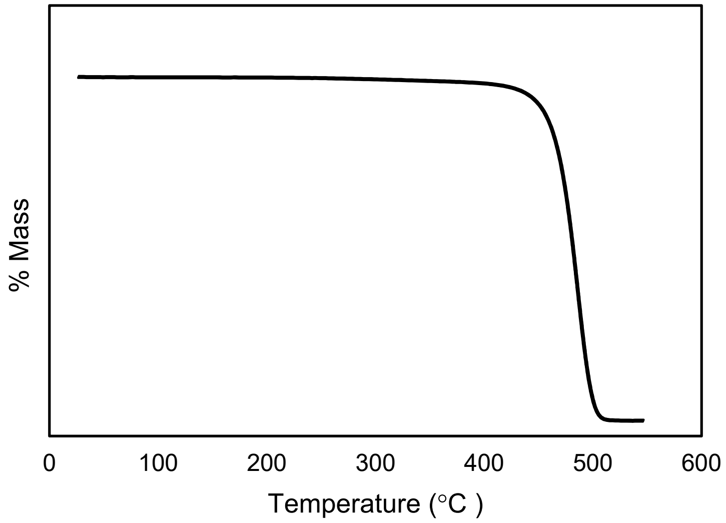

Fig. S130 TGA curve of Table 2, Entry 6.

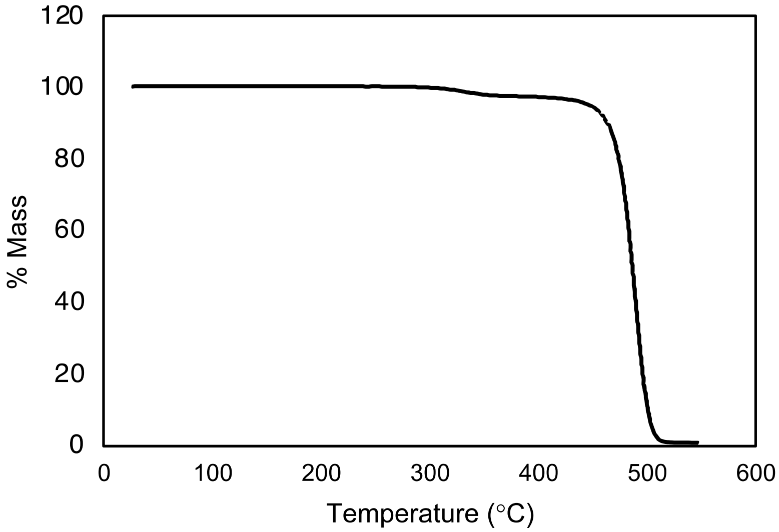

Fig. S131 TGA curve of Table S1, Entry 1.

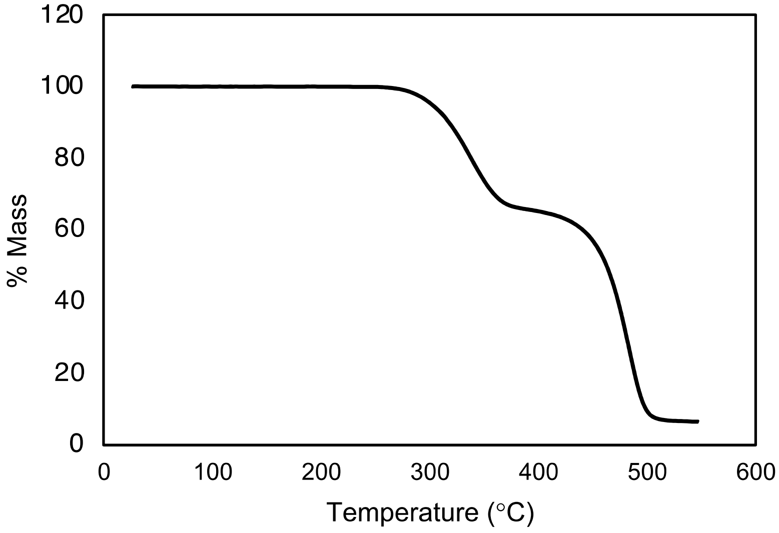

Fig. S132 TGA curve of Table S2, Entry 1.

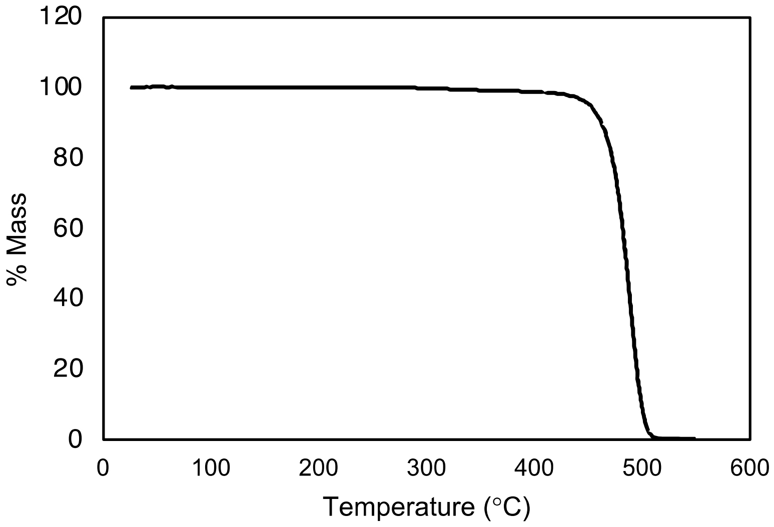

Fig. S133 TGA curve of Table S3, Entry 1.

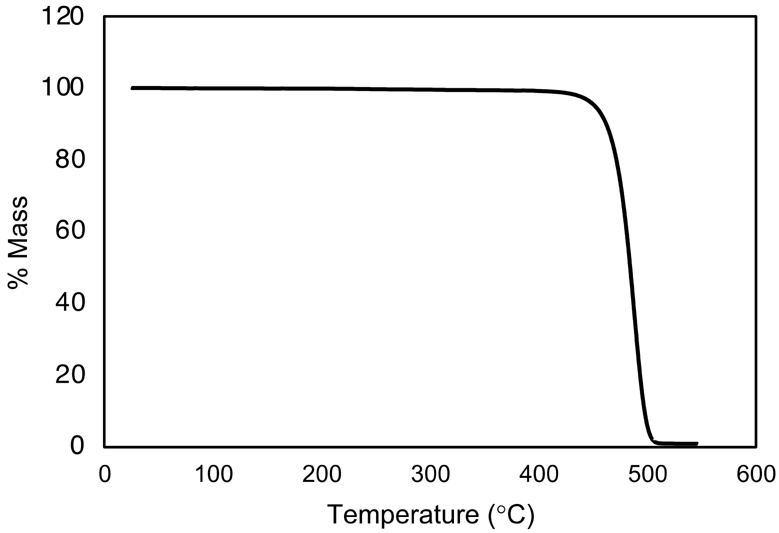

Fig. S134 TGA curve of Table S3, Entry 2.

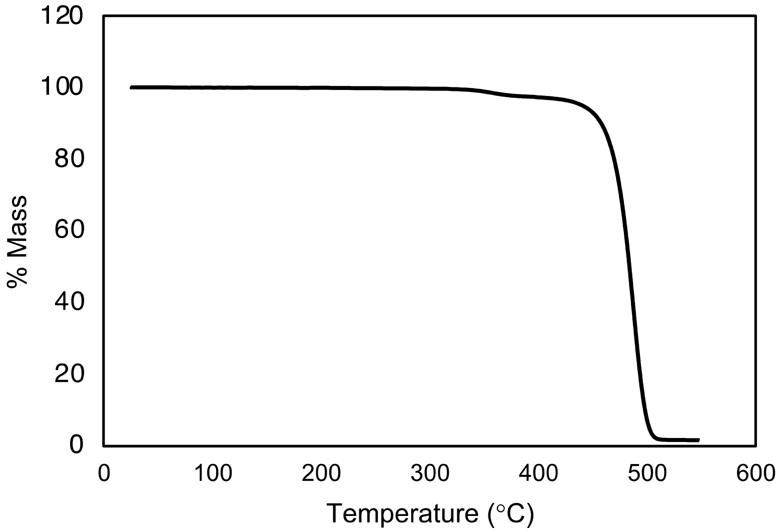

Fig. S135 TGA curve of Table S3, Entry 3.

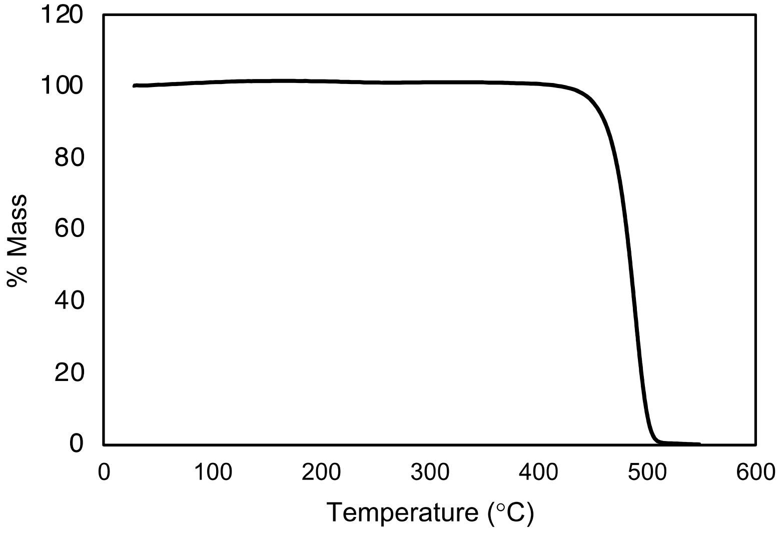

Fig. S136 TGA curve of Table S3, Entry 4.

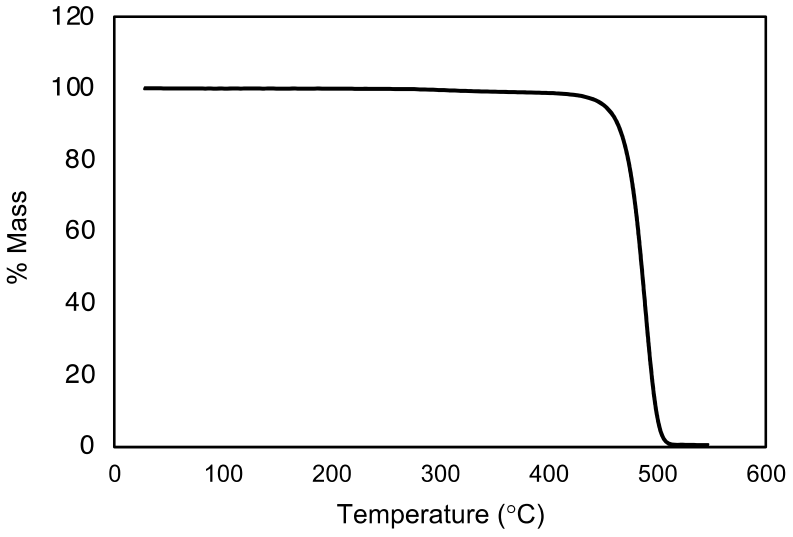

Fig. S137 TGA curve of Table S3, Entry 5a.

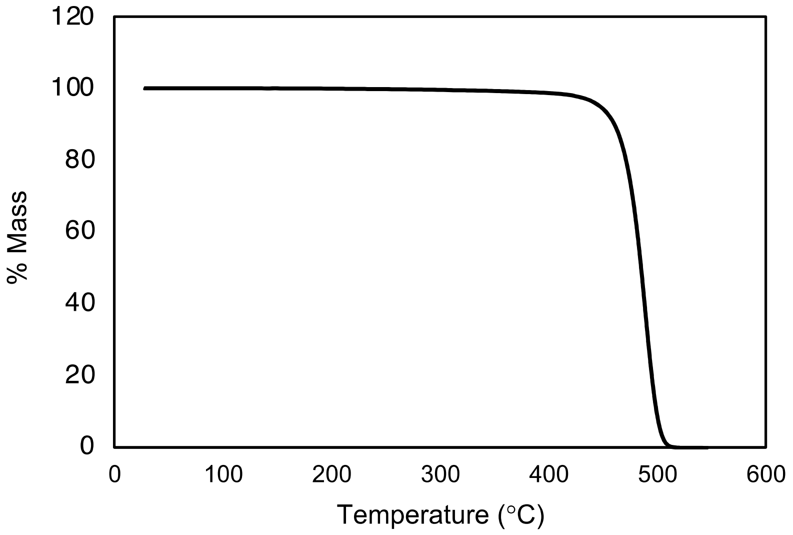

Fig. S138 TGA curve of Table S4, Entry 1a.

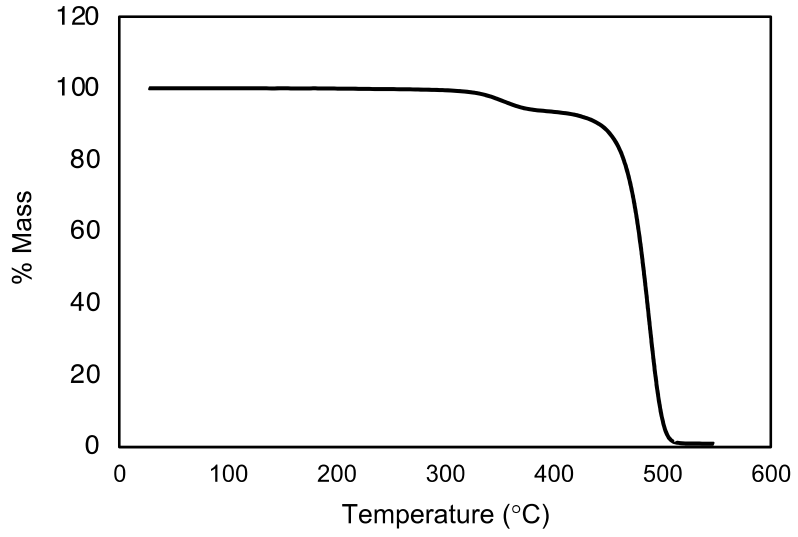

Fig. S139 TGA curve of Table S4, Entry 1b.

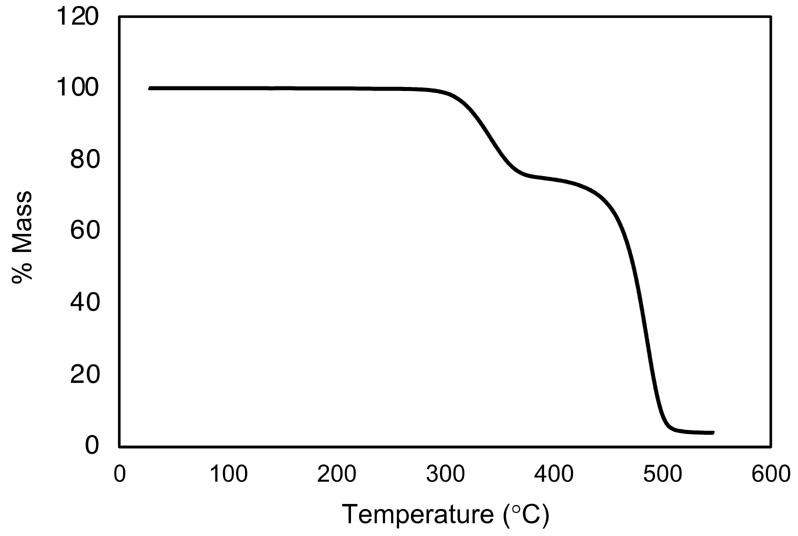

Fig. S140 TGA curve of Table S4, Entry 1c.

5. Gel Permeation Chromatography (GPC)

Table S7 Table of molar mass data for PVC items, analyzed via light scattering signal and eluted with THF.

Entry	Item	M_n (kDa)[c]	Đ[c]
1	PVC Low M_n	46.3	1.7
2	35 kDa	145.8	1.6
3	47 kDa	42.0	1.6
4	99 kDa	87.7	2.4
5	Extracted PVC lizard	50.8	2.1
6	Extracted Vinyl Record	74.4	1.5
7	Extracted Rigid PVC Pipe	72.7	1.8
8	Extracted Flexible PVC Pipe	67.9	1.7

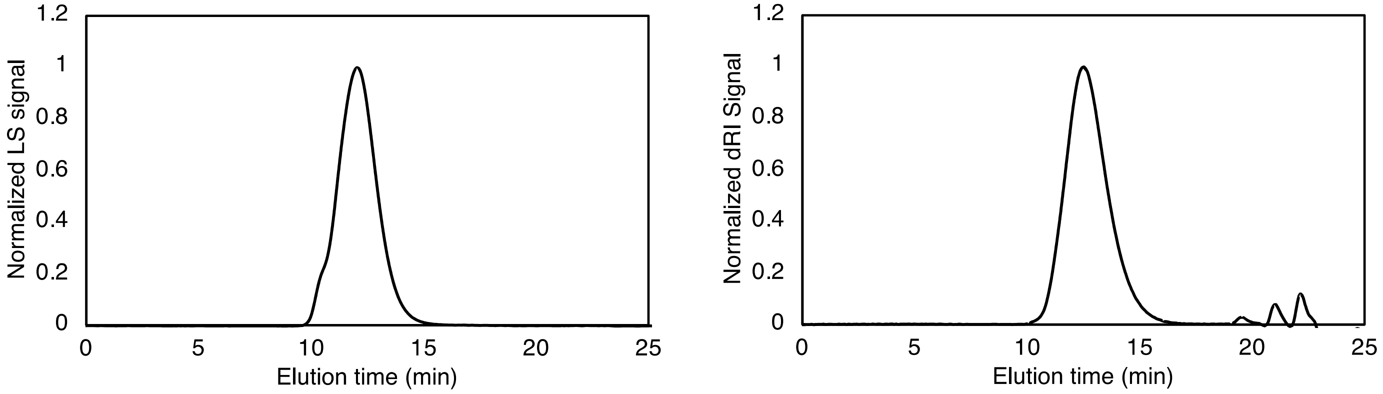

Fig. S141 GPC traces (MALS detector left, RI detector right) corresponding to low molecular weight PVC in Table S4, Entry 1. Collected at 30 ºC in THF (CAS: 9002-86-2, Product #: 81388, Sigma Aldrich).

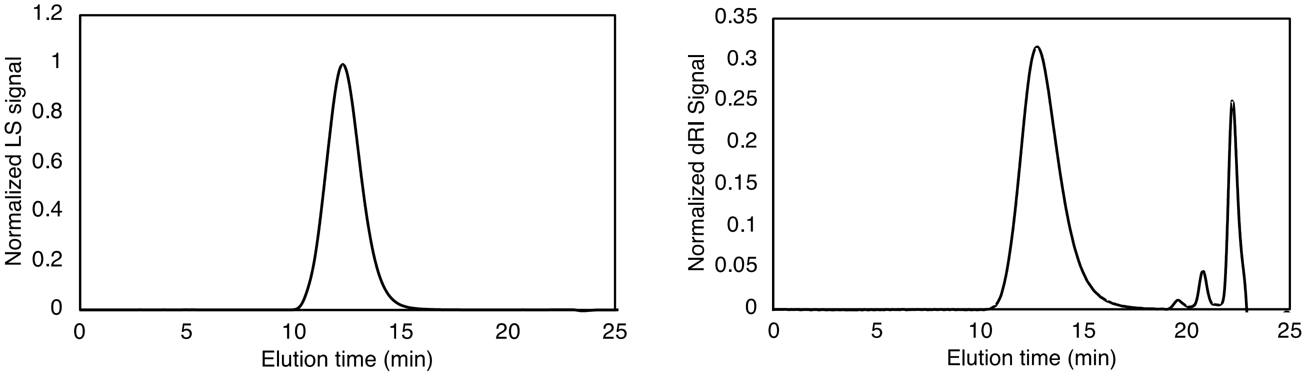

Fig. S142 GPC traces (MALS detector left, RI detector right) corresponding to PVC, M_n = 35 kDa in Table S4, Entry 2. Collected at 30 ºC in THF (Product #: 189588, Sigma Aldrich).

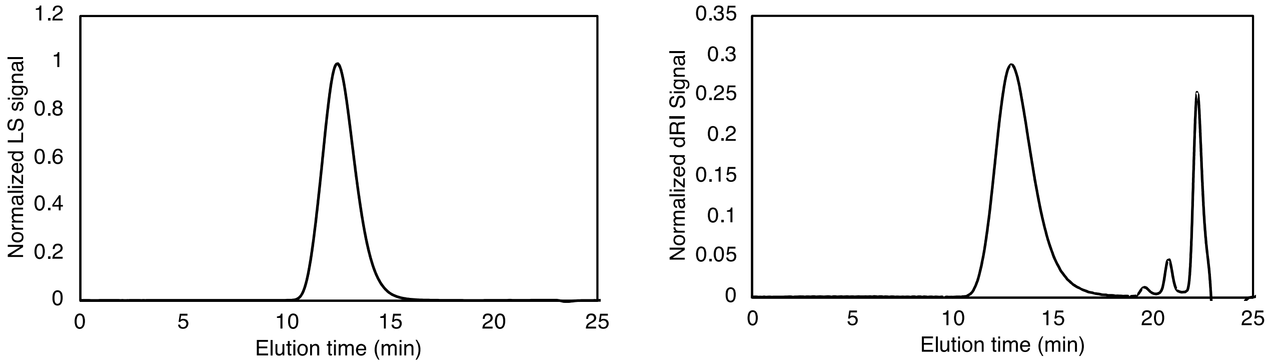

Fig. S143 GPC traces (MALS detector left, RI detector right) corresponding to PVC, M_n = 47 kDa in Table S4, Entry 3. Collected at 30 ºC in THF (Product #: 389232, Sigma Aldrich).

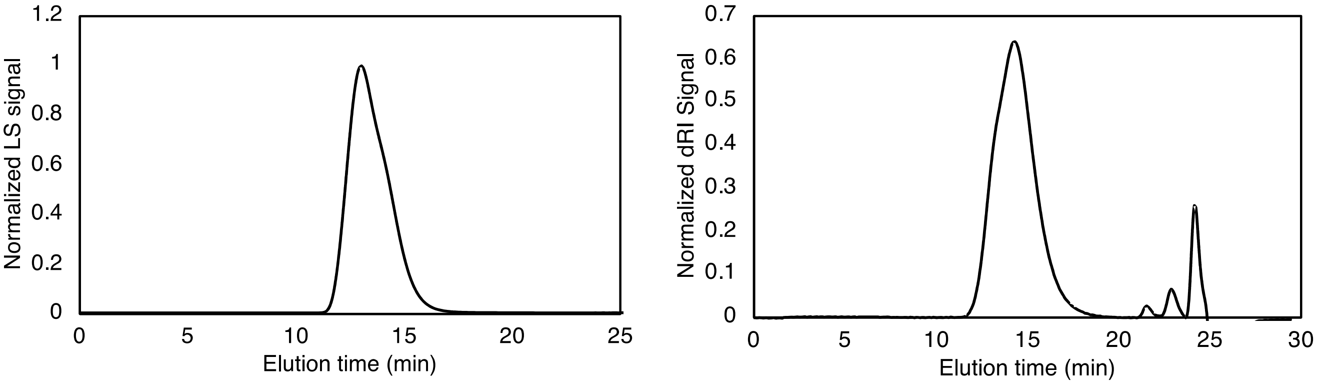

Fig. S144 GPC traces (MALS detector left, RI detector right) corresponding to PVC, M_n = 99 kDa in Table S4, Entry 4. Collected at 30 ºC in THF (Product #: 346764, Sigma Aldrich).

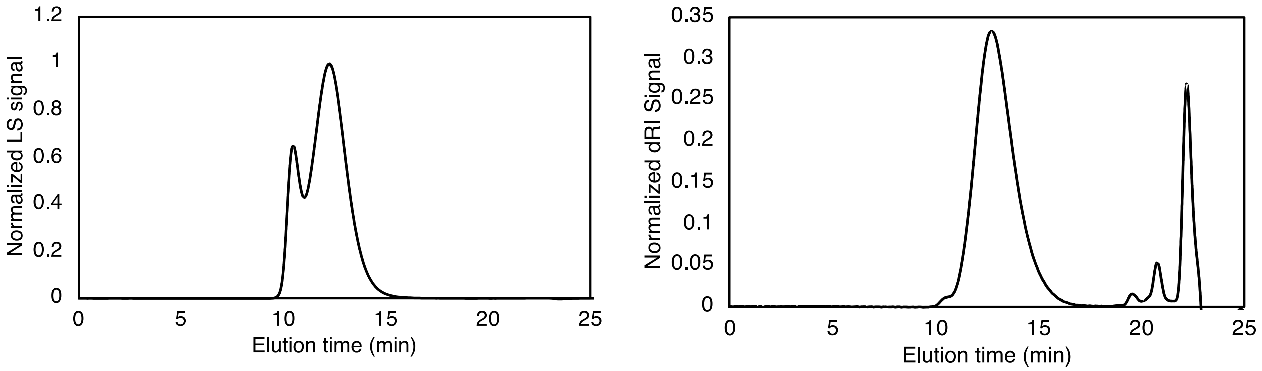

Fig. S145 GPC traces (MALS detector left, RI detector right) corresponding to PVC toy lizard item in Table S4, Entry 5. Product was purified by method described in **2.12**. Collected at 30 ºC in THF.

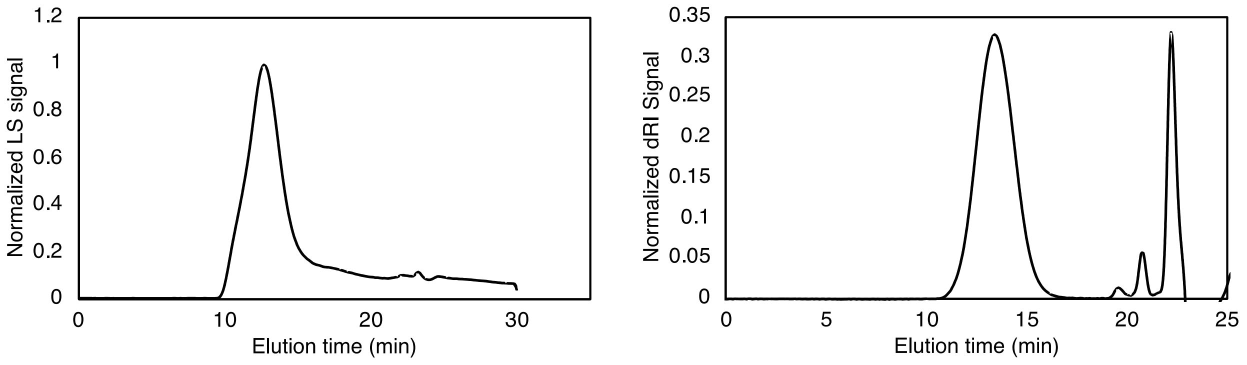

Fig. S146 GPC traces (MALS detector left, RI detector right) corresponding to PVC vinyl record item in Table S4, Entry 6. Product was purified by method described in **2.12**. Collected at 30 ºC in THF.

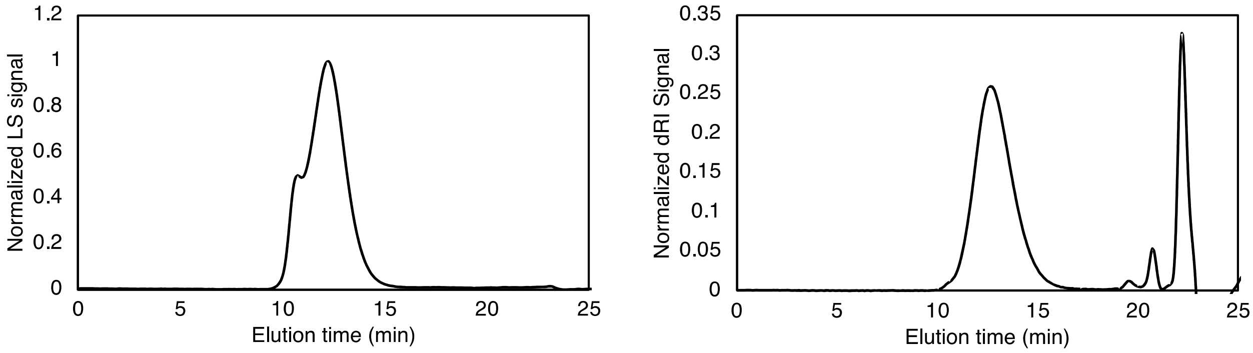

Fig. S147 GPC traces (MALS detector left, RI detector right) corresponding to rigid PVC pipe item in Table S4, Entry 7. Product was purified by method described in **2.12**. Collected at 30 ºC in THF.

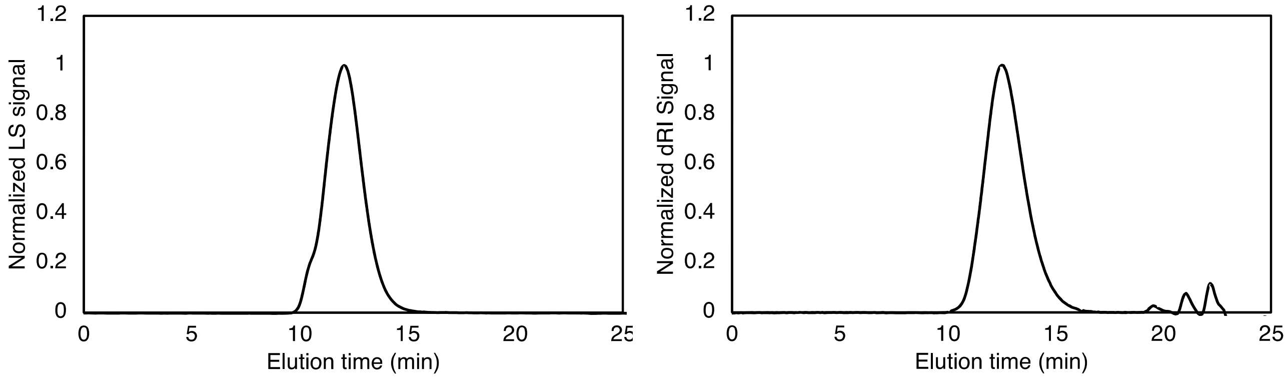

Fig. S148 GPC traces (MALS detector left, RI detector right) corresponding to flexible PVC pipe item in Table S4, Entry 8. Product was purified by method described in **2.12**. Collected at 30 ºC in THF.

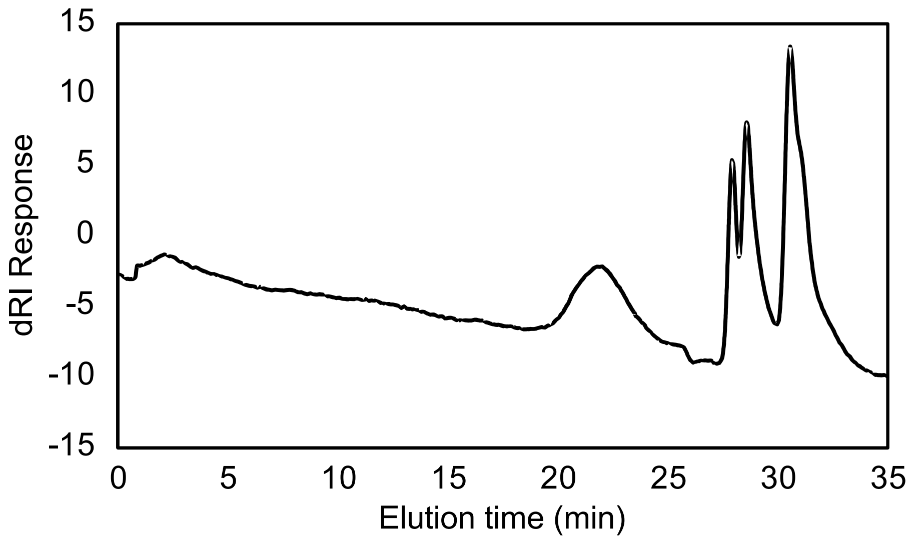

Fig. S149 GPC trace (collected at 140 ºC in TCB) corresponding to Table 1, Entry 1a.

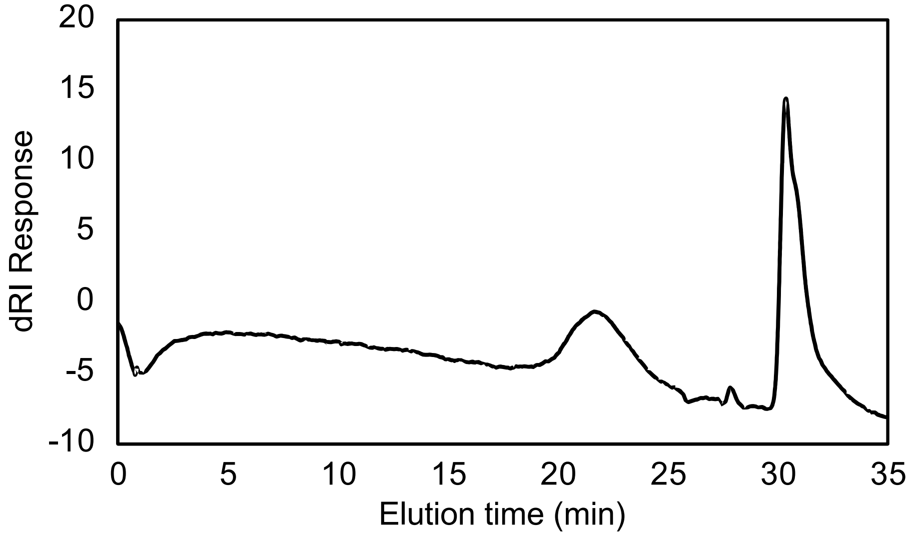

Fig. S150 GPC trace (collected at 140 ºC in TCB) corresponding to Table 1, Entry 1b.

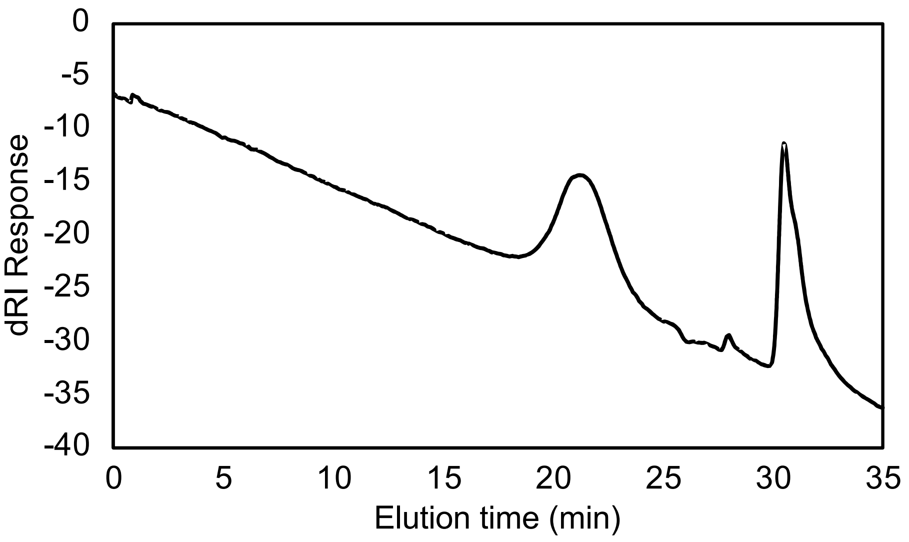

Fig. S151 GPC trace (collected at 140 ºC in TCB) corresponding to Table 1, Entry 2a.

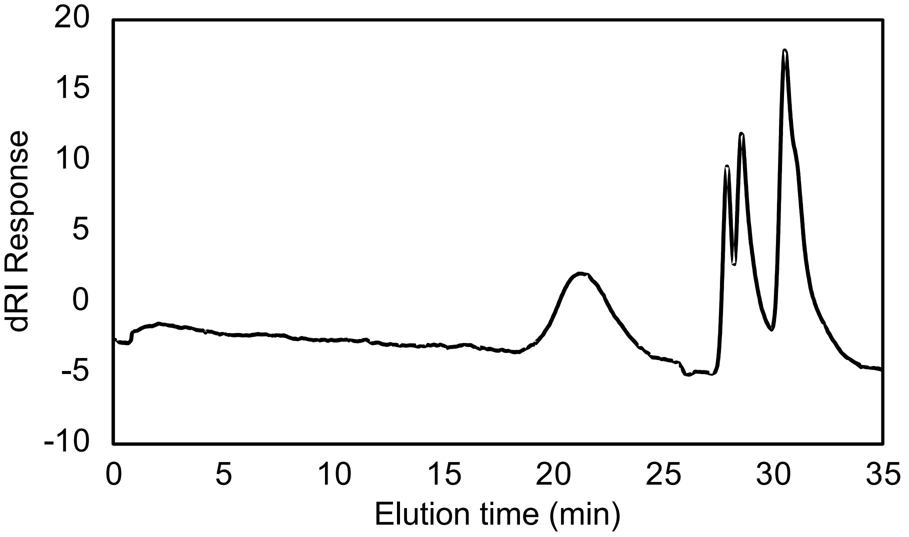

Fig. S152 GPC trace (collected at 140 ºC in TCB) corresponding to Table 1, Entry 2b.

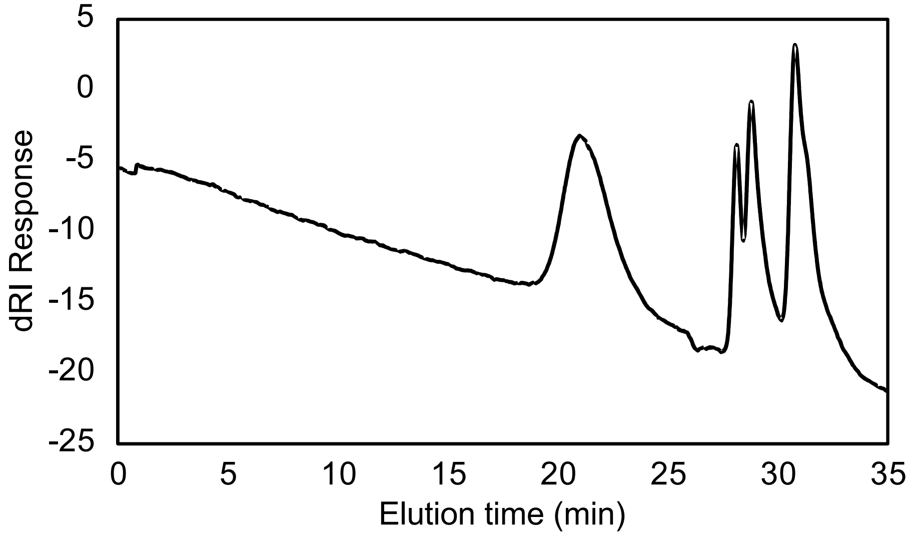

Fig. S153 GPC trace (collected at 140 ºC in TCB) corresponding to Table 1, Entry 3a.

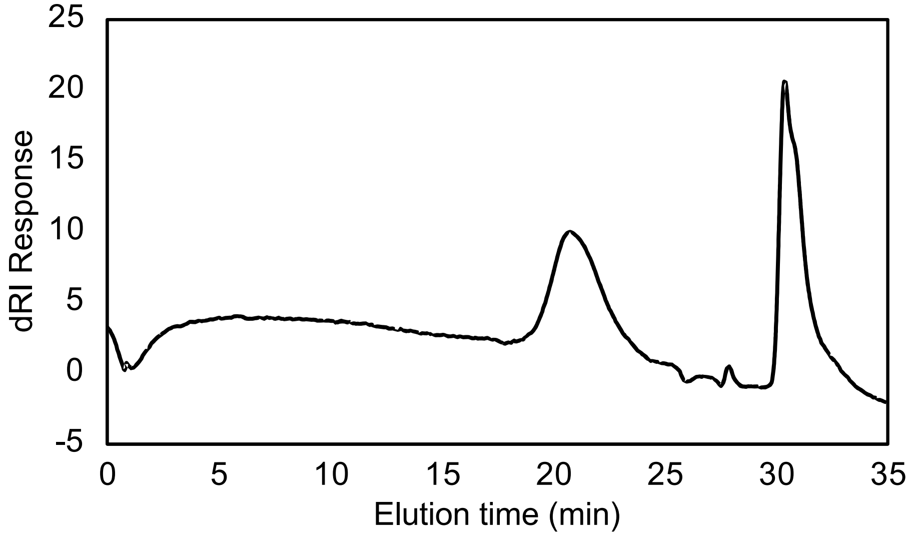

Fig. S154 GPC trace (collected at 140 ºC in TCB) corresponding to Table 1, Entry 3b.

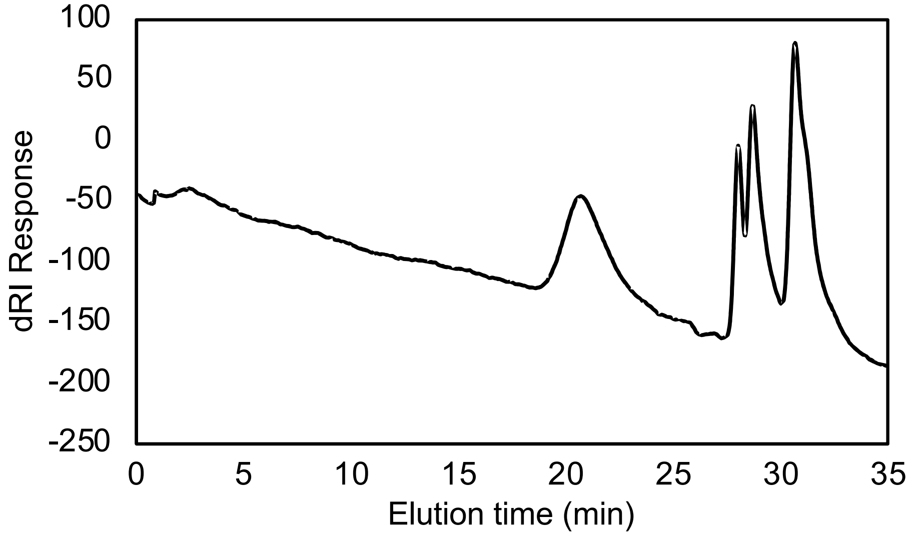

Fig. S155 GPC trace (collected at 140 ºC in TCB) corresponding to Table 1, Entry 4a.

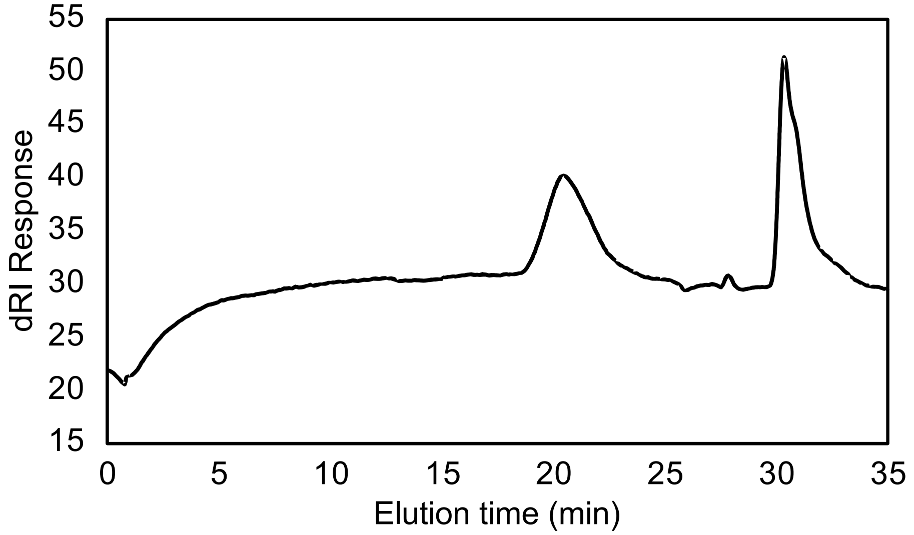

Fig. S156 GPC trace (collected at 140 ºC in TCB) corresponding to Table 1, Entry 4b.

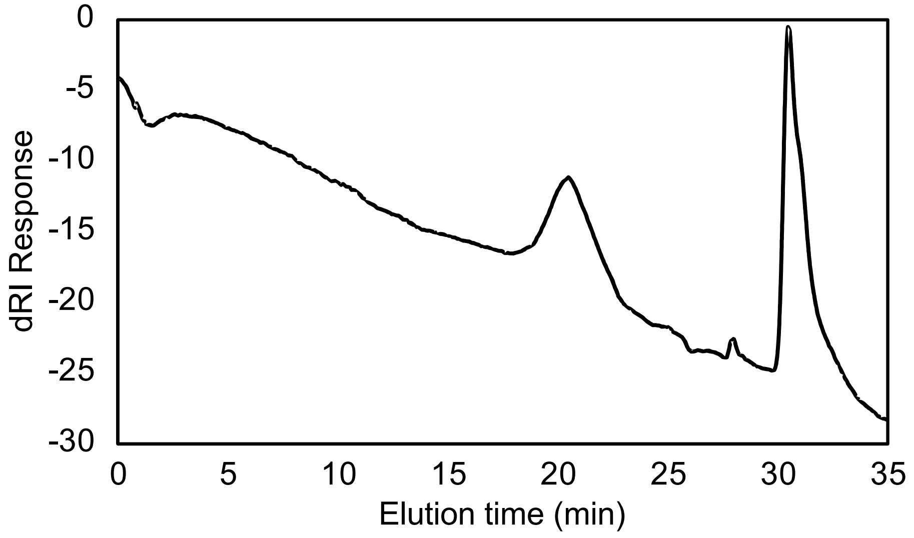

Fig. S157 GPC trace (collected at 140 ºC in TCB) corresponding to Table 1, Entry 5a.

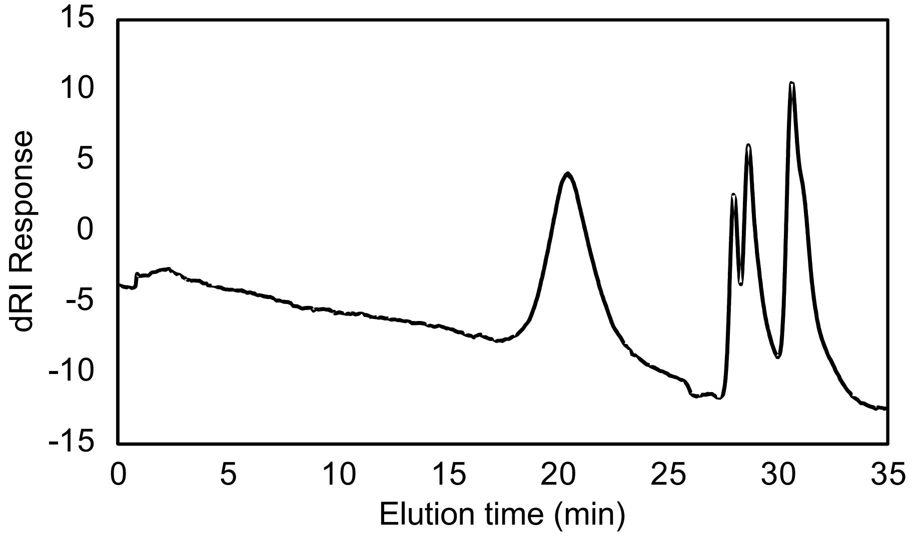

Fig. S158 GPC trace (collected at 140 ºC in TCB) corresponding to Table 1, Entry 5b.

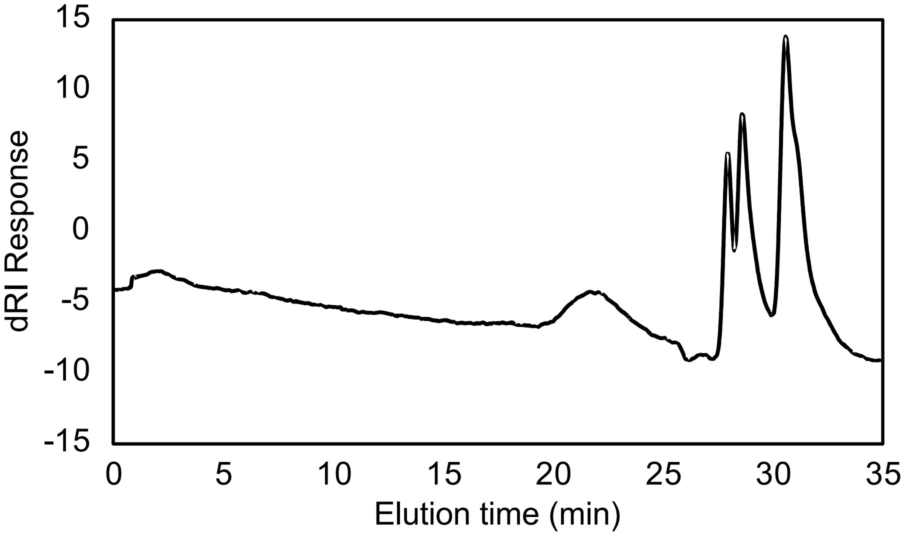

Fig. S159 GPC trace (collected at 140 ºC in TCB) corresponding to Table 1, Entry 6a.

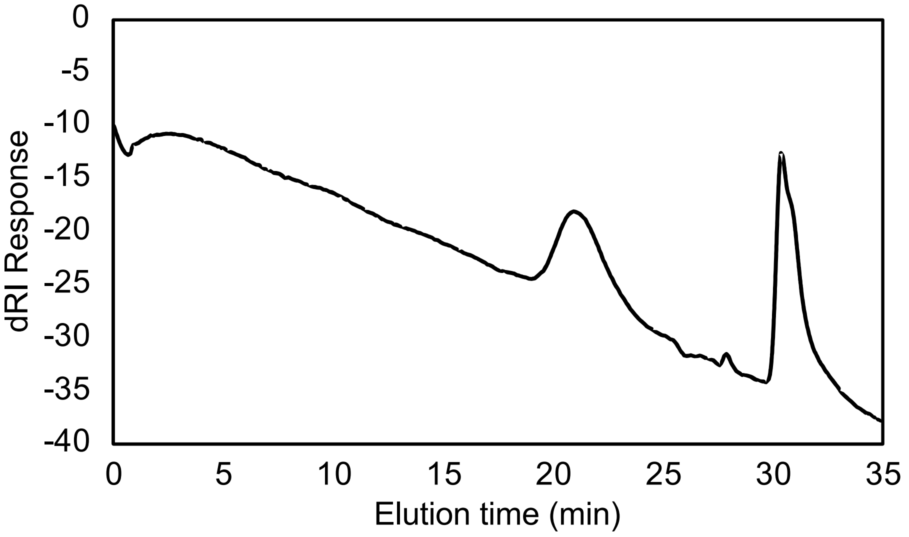

Fig. S160 GPC trace (collected at 140 ºC in TCB) corresponding to Table 1, Entry 6b.

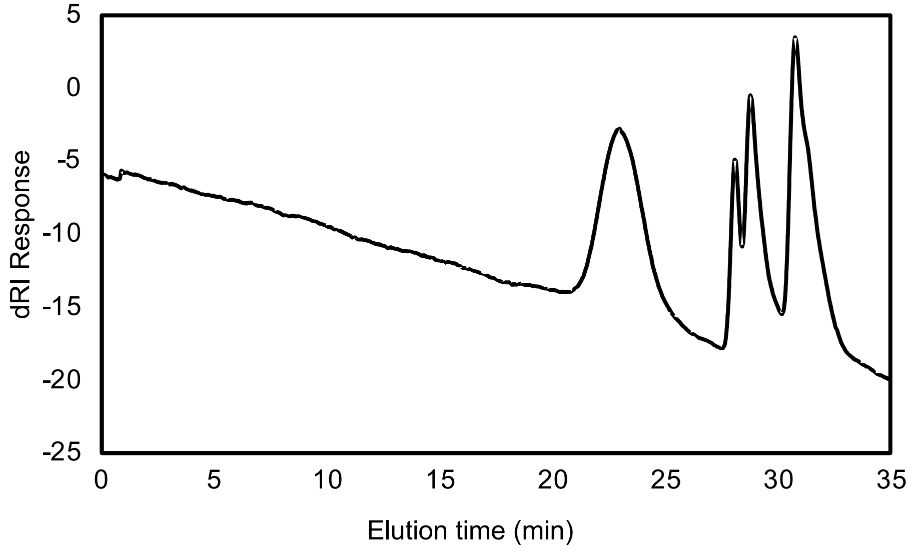

Fig. S161 GPC trace (collected at 140 ºC in TCB) corresponding to Table 2, Entry 1.

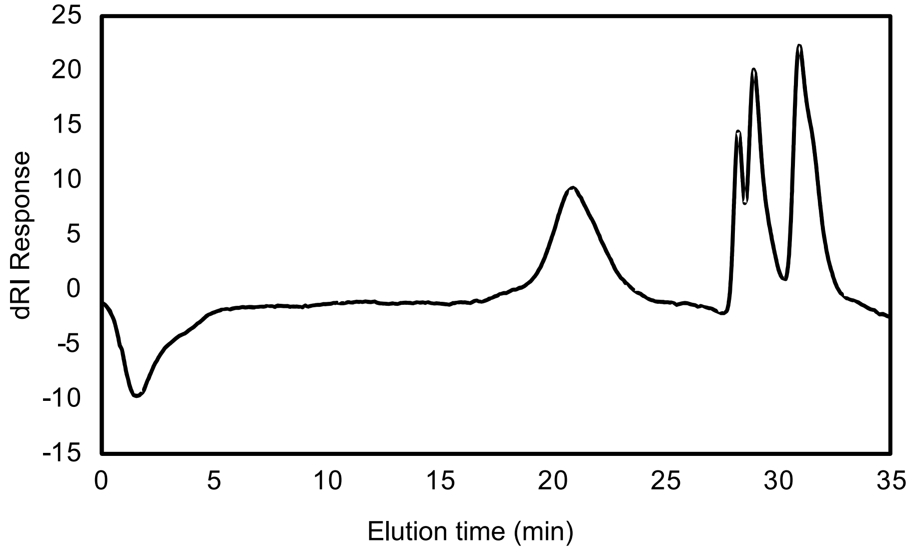

Fig. S162 GPC trace (collected at 140 ºC in TCB) corresponding to Table 2, Entry 2.

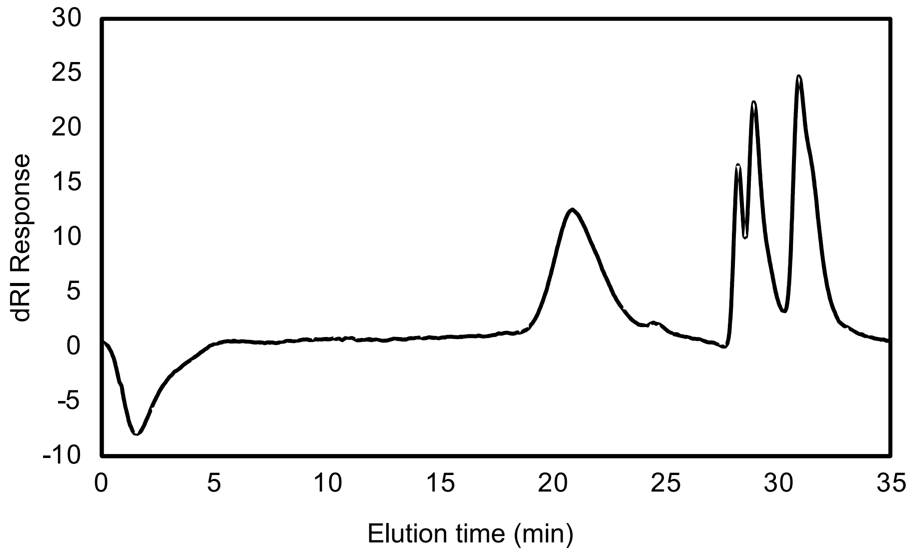

Fig. S163 GPC trace (collected at 140 ºC in TCB) corresponding to Table 2, Entry 3.

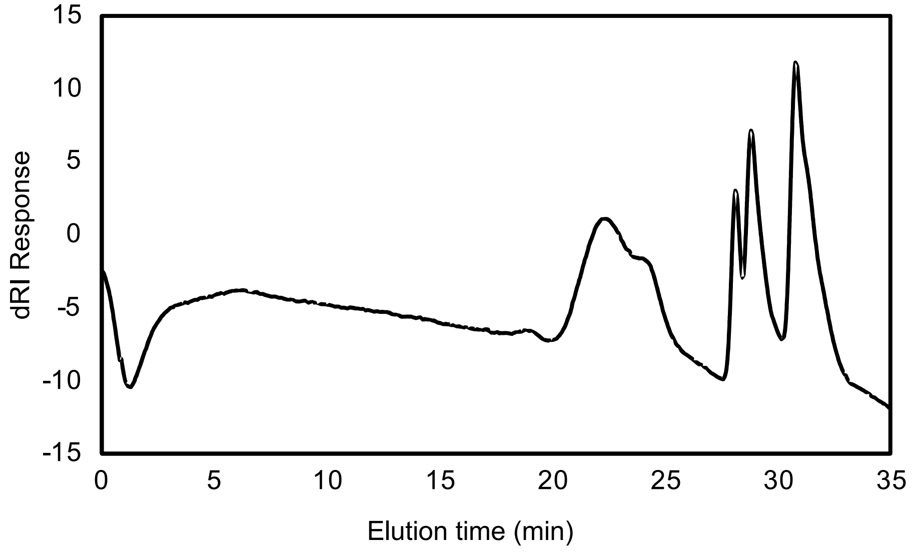

Fig. S164 GPC trace (collected at 140 ºC in TCB) corresponding to Table 2, Entry 4.

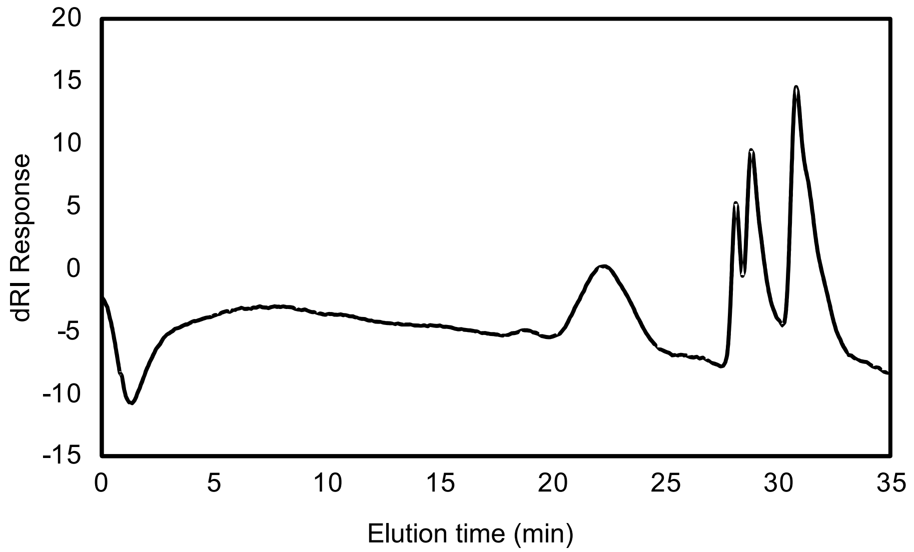

Fig. S165 GPC trace (collected at 140 ºC in TCB) corresponding to Table 2, Entry 5.

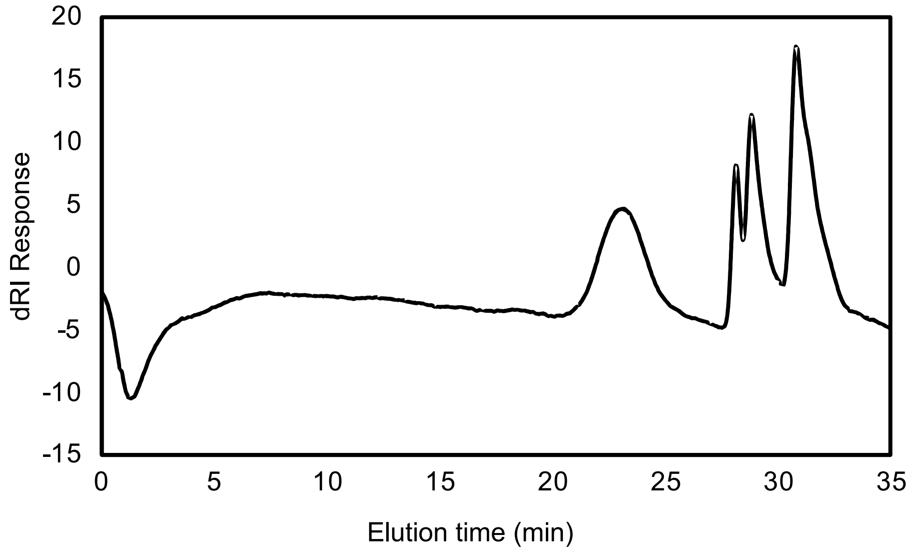

Fig. S166 GPC trace (collected at 140 ºC in TCB) corresponding to Table 2, Entry 6.

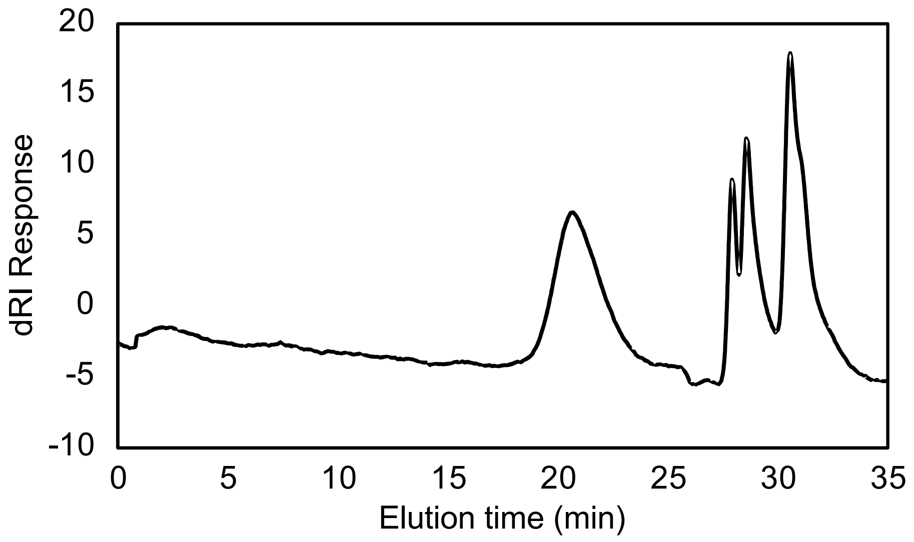

Fig. S167 GPC trace (collected at 140 ºC in TCB) corresponding to Table S3, Entry 5b (M_n = 32.6 kDa, Đ = 2.4).

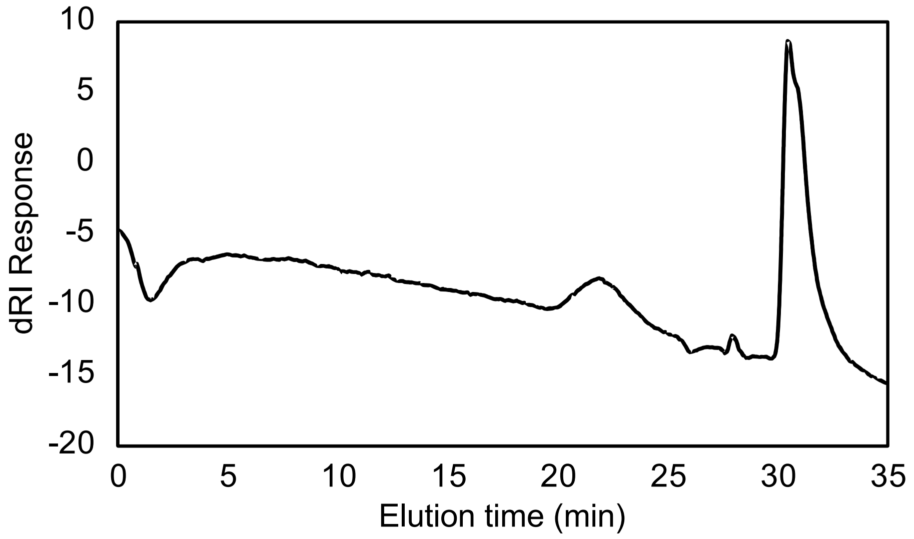

Fig. S168 GPC trace (collected at 140 ºC in TCB) corresponding to Table S4, Entry 1.

8. ^{13}C and ^{1}H NMR spectroscopy for calculating branch length

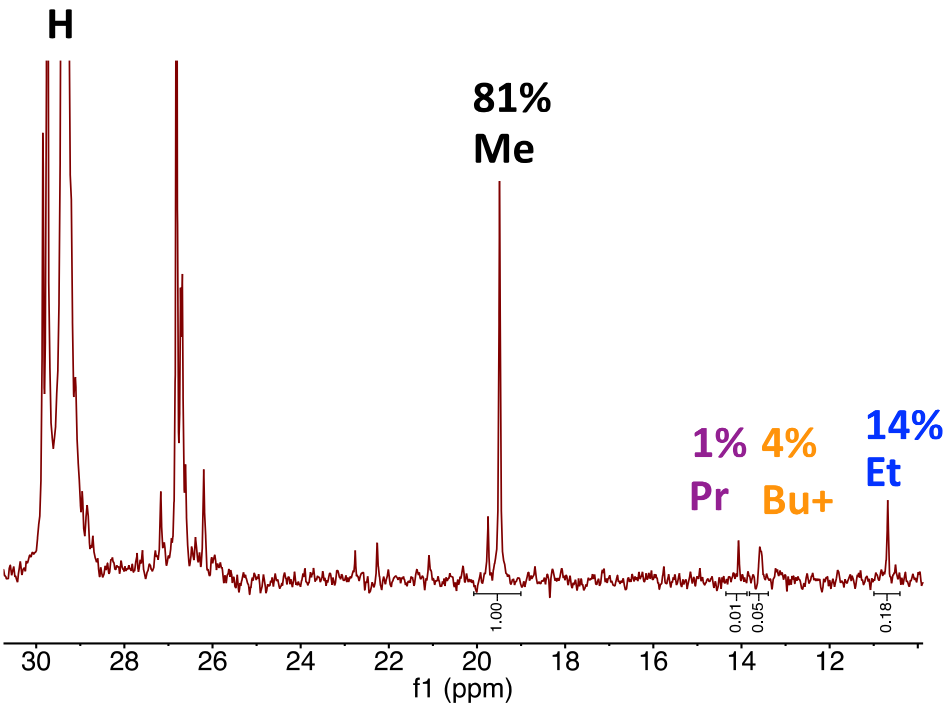

Fig. S169 $^{13}C\{^{1}H\}$ NMR spectrum of PVC to PE sample (in tetrachlorethane-d_2) using conditions from Table S1, Entry 4a. Reaction doubled in size, 20 mg of dried product used for NMR. Reaction reached 94% Cl loss by ^{1}H NMR.[3]

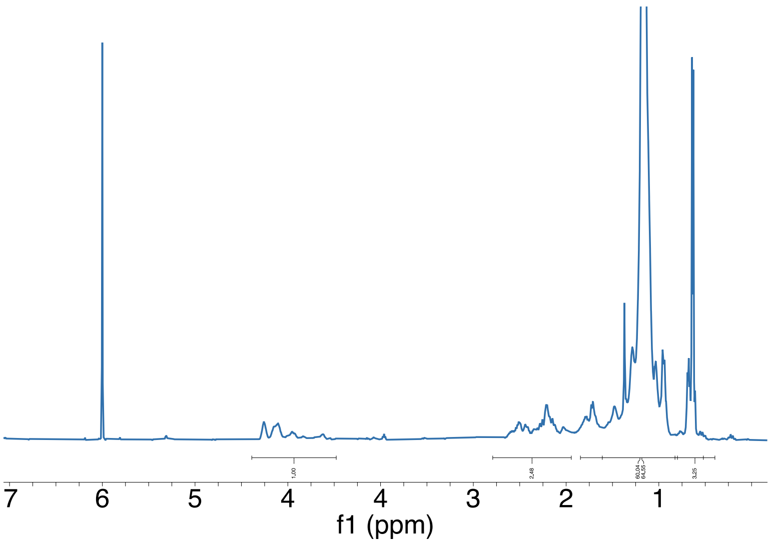

Fig. S170 ^{1}H NMR spectrum of PVC to PE sample (in tetrachlorethane-d_2) using conditions from Table S1, Entry 4a. Reaction doubled in size, 20 mg of dried product used for NMR. Reaction reached 94% Cl loss by ^{1}H NMR.

9. Control reactions for activation of CH_2Cl_2 by silylium ions

Control reactions were performed in Parr Reactors to determine if silylium ions can active CH_2Cl_2. 4 Parr Reactor vessels were loaded with different reagents, and the pressure of the reaction was monitored before, during, and after to determine if CH_4 is formed, which would be a result of CH_2Cl_2 activation. Similar pressures in all reaction vessels indicates no measurable activation of CH_2Cl_2.

General reaction conditions: The Parr Reactor vessels were loaded with reagents, and then allowed to stir and heat at 110 °C for 18 hours. The pressure of the reaction was recored before heating, 6 hours into heating, and after the reaction was cooled to back 25 °C after the reaction.

Entry 1: 9 mL of CH_2Cl_2.
Entry 2: 9 mL of CH_2Cl_2, 2.1 mL of Et_3SiH
Entry 3: 9 mL of CH_2Cl_2, 2.1 mL of Et_3SiH, 16.8 mg of $[Ph_3C][B(C_6F_5)_4]$
Entry 3: 9 mL of CH_2Cl_2, 2.1 mL of Et_3SiH, 16.8 mg of $[Ph_3C][B(C_6F_5)_4]$, 136.2 mg of low M_n PVC (Sigma-Aldrich)

Table S8 Pressure of Parr Reactor vessels before, during, and after reactions.

Entry	Pressure before (bar)	Pressure during (bar)	Pressure after (bar)
1	0.1	6.2	0.1
2	0.2	6.0	0.2
3	0.3	5.9	0.2
4	0.7	6.2	0.6

10. Conversion of Et_3SiH to Et_3SiCl monitored by ^1H NMR

10.1 Monitoring Et_3SiCl conversion from dechlorination of PVC at 110 °C:

([Ph_3C][$B(C_6F_5)_4$]) (16.6 mg, 0.018 mmol, 0.8 mol%), Et_3SiH (0.84 mL, 5.26 mmol, 2.4 equiv.) CH_2Cl_2 (3 mL) and a stir bar were charged to a 2 dram vial. The vial was then stirred for 5 minutes. PVC (135.8 mg, 2.176 mmol, 1 equiv.) was weighed into a 2 dram vial equipped with a Teflon-coated cap. The CH_2Cl_2 mixture was then added to the PVC, and the vial was sealed with Teflon tape along the inside of the cap, and electrical tape on the outside. The vial was then placed inside a Chemglass high-throughput tray that was preheated to 110 °C. After 2 hrs, an aliquot was taken and a ^1H NMR was recorded in $CDCl_3$ to determine the conversion of Et_3SiH to Et_3SiCl.

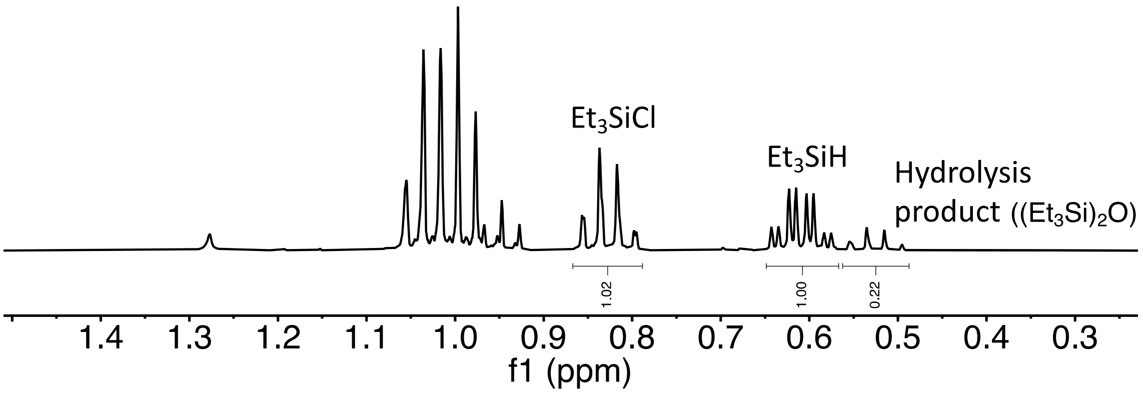

Fig. S171 ^1H NMR spectrum of aliquot for Et_3SiH to Et_3SiCl (in $CDCl_3$) using conditions from Table S1, Entry 2a-b.[4]

11. References

[1] L. Wild, R. Ranganath, T. Ryle, *J. Polym. Sci. A-2 Polym. Phys.* 1971, **9 (12)**, 2137–2150.

[2] F. A. Hicks, J. C. Jenkins, M. Brookhart, *Organometallics* 2003, **22 (17)**, 3533–3545.

[3] Y. Ge, Q. Cai, Y. Wang, J. Gao, Y. Chi, S. Dai, *Front. Chem.* 2022, **10**, 886888.

[4] N. Almenara, S. Azpeitia, M. A. Garralda, M. A. Huertos, *Dalton Trans.* 2018, **47**, 16225–16231.
